# Synergistic decatungstate and Brønsted acid catalysis enables direct C–H indolation of alkanes and aldehydes

**DOI:** 10.1039/d6sc01827k

**Published:** 2026-04-27

**Authors:** Gangqi Peng, Yangling Deng, Mengxuan Zhang, Yuxuan He, Hao Cheng, Xinbin Wang, Yuanyuan An, Guanyinsheng Qiu, Danqing Zheng

**Affiliations:** a State Key Laboratory of Materials-Oriented Chemical Engineering, College of Chemical Engineering, Nanjing Tech University Nanjing 211816 China zhengd@njtech.edu.cn; b College of Biological and Chemical Engineering, Jiaxing University Jiaxing 314001 China qiuguanyinsheng@mail.zjxu.edu.cn

## Abstract

Indole skeletons are ubiquitous in natural products, pharmaceuticals, and agricultural agents, making direct C–H indolation strategies highly significant. Direct radical alkylation of indoles, however, is often hampered by the inherent polarity mismatch between nucleophilic alkyl radicals and the electron-rich indole ring. Herein, we report that synergistic decatungstate and Brønsted acid catalysis enables the direct radical C–H indolation of alkanes and aldehydes with 2-indolylmethanols. The acid-mediated ionization of 2-indolylmethanols generates delocalized carbocations, which efficiently capture alkyl or acyl radicals derived from the decatungstate-catalyzed hydrogen atom transfer (HAT) process. This cascade delivers C3-functionalized indoles with high regioselectivity under mild conditions. Mechanistic studies suggest that the Brønsted acid plays a dual role by facilitating the formation of key carbocation intermediates and accelerating the HAT step. The late-stage C–H indolation of complex natural products and pharmaceutical agents further demonstrates the synthetic versatility of this protocol.

## Abstract 2

Synergistic decatungstate and Brønsted acid catalysis enables direct C–H indolation of alkanes/aldehydes with 2-indolylmethanols. This radical-polar crossover strategy overcomes polarity mismatch, offering broad substrate scope and high efficiency.

## Graphical abstract

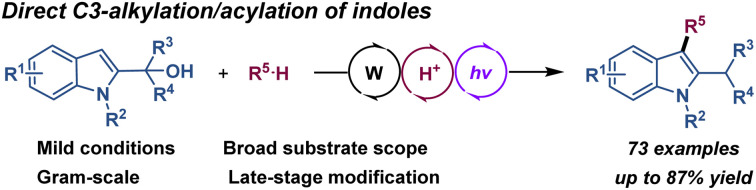

## Introduction

**Fig. 1:**
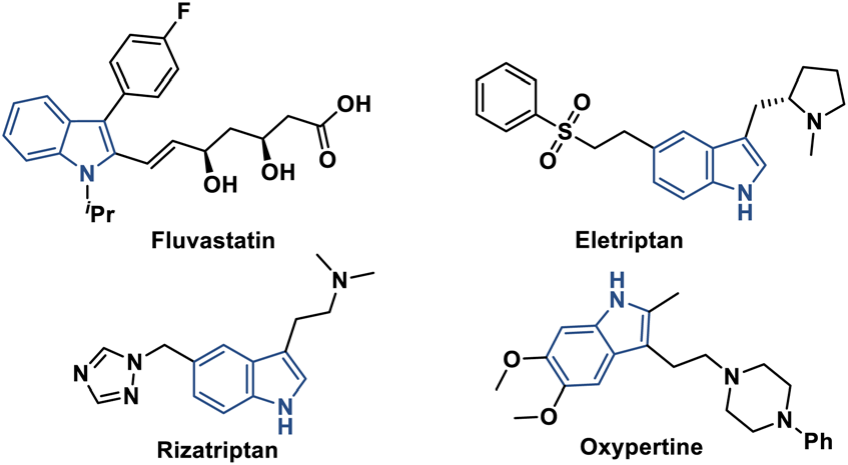
Examples of drugs containing the indole moiety.

The indole scaffold represents a highly adaptable and valuable heterocyclic system that is extensively distributed across various natural products, medicinal compounds, and agricultural chemicals (Fig. 1).^1^ This prevalence stems from its remarkable structural versatility and diverse biological functionalities. In organic synthesis, significant research efforts have been consistently directed toward the exploration and advancement of innovative strategies for both the assembly and modification of indole-based structures.^2^

**Scheme 1:**
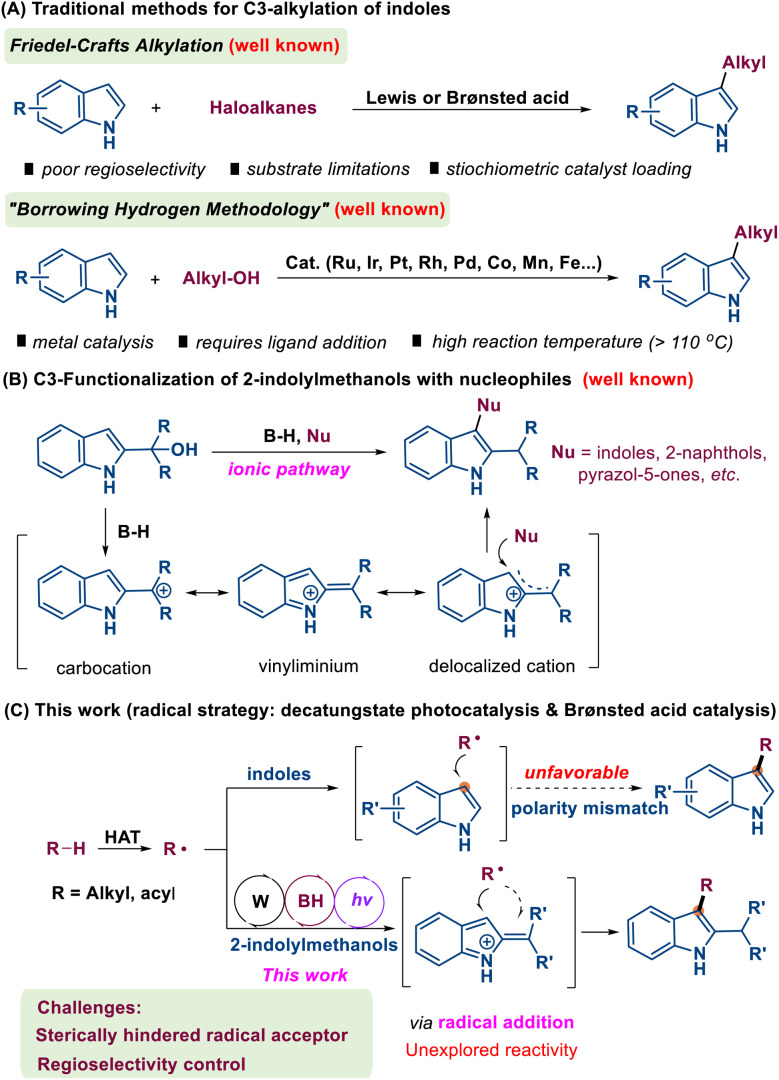
Strategies for the C-3 functionalization of indoles.

Among the various established methodologies, C3-alkylation of indoles has emerged as a particularly promising approach, attracting significant research attention due to its effectiveness in generating C3-substituted indole derivatives (Scheme 1A).^3^ Traditional C3-alkylation methods mainly rely on Friedel–Crafts reactions using Lewis or Brønsted acids. However, these approaches face several limitations, including poor compatibility with green chemistry principles, the toxicity of alkyl halides and issues such as over-alkylation.^4^ In addition, the borrowing hydrogen strategy provides an alternative approach for the C3-alkylation of indoles. This methodology is advantageous due to its utilization of non-toxic and readily accessible alcohols as starting materials, with water being the only byproduct, thus aligning with green chemistry principles. Nevertheless, this method presents certain limitations, such as the use of metal catalysts (*e.g.*, Ru, Ir, Rh, Pd, Co, Mn, Fe, and Cu complexes) and the requirement for additional ligands. Moreover, the process typically requires a higher reaction temperature, which may limit its practical applications.^5,6^

Over the last decade, Shi and coworkers have disclosed a remarkable polarity reversal phenomenon that the C3-position of 2-indolemethanol exhibits electrophilic characteristics rather than its conventional nucleophilic behavior when exposed to Brønsted or Lewis acids.^7^ This intriguing transformation can be rationalized by the *in situ* formation of a delocalized cation intermediate derived from 2-indolemethanol (Scheme 1B). On this foundation, numerous reactions involving 2-indolylmethanol have subsequently been reported, encompassing diverse nucleophilic substitutions^8^ and various intermolecular cycloadditions, including [3 + 2],^9^ [3 + 3],^10^ and [4 + 3]^11^ variants. These groundbreaking findings have not only challenged the traditional paradigm of C3-nucleophilicity but have also significantly expanded the scope of substrate compatibility. Despite these advancements, it is noteworthy that both conventional and newly established methods predominantly proceed through ionic mechanisms. Crucially, such ionic pathways are inherently biased towards ‘soft’ nucleophiles, rendering them ineffective for the introduction of ‘hard’ alkyl groups derived from unactivated alkanes. Consequently, the development of a complementary radical-based strategy to overcome this limitation and achieve direct C3-alkylation remains a significant, yet largely unexplored frontier.

However, due to the intrinsic chemical properties of the C3 position of indoles, it is difficult for them to undergo attack by alkyl radicals due to polarity mismatch, making direct radical C3-alkylation of indoles unfavorable. To address this limitation, herein we report a synergistic catalytic strategy that combines hydrogen atom transfer (HAT) photocatalysis with Brønsted acid catalysis, achieving C3-alkylation of indoles through a radical process (Scheme 1C). This methodology provides a versatile platform for the synthesis of diverse C3-substituted indole derivatives, offering significant improvements in both efficiency and substrate scope.

## Results and discussion

In recent years, decatungstate-catalyzed hydrogen atom transfer (HAT) of alkanes has represented a powerful strategy for direct functionalization of inert C(sp3)-H bonds.^12^ Drawing on this established reactivity, we employed tetrabutylammonium decatungstate (TBADT) as the photocatalyst to generate alkyl radicals from alkanes for this investigation. To optimize the reaction conditions, 2-indolemethanol 1a (0.1 mmol) and cyclohexane 2a (0.1 mmol) were used as model substrates (Table 1). When the reaction was carried out in the presence of 2 mol% TBADT in a mixture of MeCN/0.25 M HCl (0.85 : 0.15, 0.1 M) under nitrogen and irradiated with a Kessil 40 W 370 nm lamp for 1 hour, the desired product 3a was isolated in 81% yield (entry 1). Replacement of aqueous HCl with organic Brønsted acids such as phosphonic acid A–B, benzoic acid C and *p*-toluenesulfonic acid (TsOH) D resulted in lower yields of 3a and the generation of regioisomer 3a′ (entries 2–5). Varying the concentration of HCl led to a slight decrease in yield (Table 1, entries 6–7). When an aqueous solution of TsOH, H_2_SO_4_ and H_3_PO_4_ was used instead of aqueous HCl, the target product 3a was obtained in 61–69% yield (Table 1, entries 8–10). Comparing the results of entries 5 and 8, it can be concluded that H_2_O played a significant role in this reaction system. The use of other solvents such as DCE, EA, and DMSO all led to negative outcomes (Table 1, entries 11–13). Replacement of TBADT with other HAT photocatalysts such as Eosin Y, FeCl_3_, and benzophenone resulted in trace yields of 3a (entries 14–16). Reducing the amount of TBADT from 2 mol% to 1 mol% resulted in a slight decrease of yield (Table 1, entry 17). Altering the amount of 2a to 0.1 mmol and 1a to 0.15 mmol afforded product 3a in 50% yield (Table 1, entry 18). Additionally, the results from controlled experiments demonstrated the necessity of TBADT, HCl and light for this transformation (Table 1, entries 19–21).

**Table 1:** Reaction optimization^a^

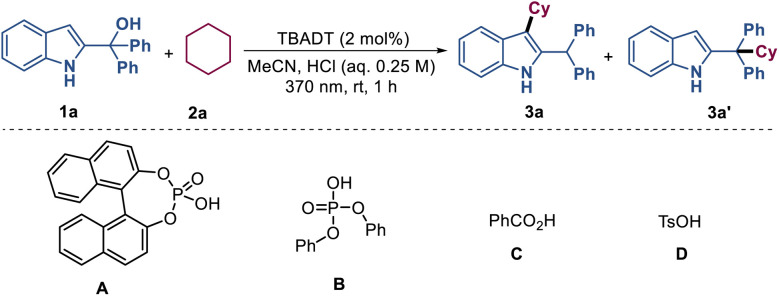			
Entry	Variations from standard conditions	3a [%]^b^	3a′ [%]^b^
1	None	87(81)^c^	n.d.
2	A (10 mol%) instead of HCl	44	12
3	B (10 mol%) instead of HCl	19	n.d.
4	C (10 mol%) instead of HCl	n.d.	n.d.
5	D (10 mol%) instead of HCl	13	n.d.
6	HCl (0.1 M)	72	10
7	HCl (0.5 M)	81	n.d.
8	0.25 M TsOH instead of HCl	63	11
9	0.125 M H_2_SO_4_ instead of HCl	69	10
10	0.08 M H_3_PO_4_ instead of HCl	61	6
11	DCE as solvent	Trace	n.d.
12	EA as solvent	n.d.	n.d.
13	DMSO as solvent	n.d.	n.d.
14	Eosin Y instead of TBADT	Trace	n.d.
15	FeCl_3_ instead of TBADT	Trace	n.d.
16^c^	Benzophenone instead of TBADT	n.d.	n.d.
17	TBADT (1 mol%)	76	n.d.
18	1.0 eq. of 2a, 1.5 eq. of 1a	50	n.d.
19^c^	No TBADT	n.d.	n.d.
20	No HCl	n.r.	n.r.
21	In the dark	n.r.	n.r.

a Reaction conditions: 1a (0.1 mmol), 2a (1.0 mmol), TBADT (2 mol%), 0.25 M HCl (0.15 mL), MeCN (0.85 mL), Kessil 370 nm, 1 h, rt. M = mol L^−1^. n.d. = not detected. n.r. = no reaction.

b Yields were determined by GC.

c Isolated yields based on 1a.

With the optimized conditions identified, we then explored the substrate scope and site selectivity of this transformation. As illustrated in Table 2, a broad range of hydrocarbon compounds R^1^–H 2 including cycloalkanes, chain alkanes, olefins, benzylic substrates and aldehydes were demonstrated to be suitable as radical precursors in this reaction. It was observed that cycloalkanes with varying ring sizes and substituents all yielded the corresponding products 3a–3h in 58–81% yields. Different alkyl- or aryl-substituted cyclohexanes were also compatible, delivering 3e–3h in good yields. Thioethers and cyclic ketones were also viable substrates, providing the desired products 3i–3k with yields of 46–63%. Linear and branched alkanes could be converted into products 3l–3p in moderate yields. Notably, the reaction with linear alkanes produced a mixture of positional isomers, indicating that indolylation of the secondary C(sp^3^)−H position was more favorable than other positions (3l and 3m). Additionally, exclusive site-selectivity on the tertiary C–H bond was observed in the reaction with branched alkanes (3n–3p). The divergent regioselectivity observed between cyclic and acyclic alkanes is characteristic of decatungstate-mediated HAT. For acyclic precursors (3n–3p), the hydrogen abstraction is driven by bond dissociation energy (BDE), favoring the weaker tertiary C–H bonds. However, the excited state [W_10_O_32_]^4−^ is highly sensitive to steric hindrance. In rigid, substituted cyclic alkanes (3e–3h), the tertiary C–H bonds are sterically shielded, leading the bulky photocatalyst to preferentially abstract hydrogen from the more accessible secondary C–H positions. Moreover, olefins furnished indolated products 3q–3t at the allylic position with yields ranging from 60% to 86%. The reactivity of a variety of substrates containing benzylic C(sp^3^)−H bonds was investigated. It was found that electron-neutral or electron-poor alkyl benzenes, diphenylmethane, indane, tetralin, 9H-xanthene, isoindolin-1-one and even heterocyclic substrates all reacted smoothly with 2-indolemethanol 1a to yield the corresponding products (3u–3ap) in moderate to good yields. It should be noted that the reaction resulted in two regioisomers when there were two benzylic reactive sites in the substrate (3ae–3af).

**Table 2:** Reaction of 1a with various alkanes and aldehydes^a^

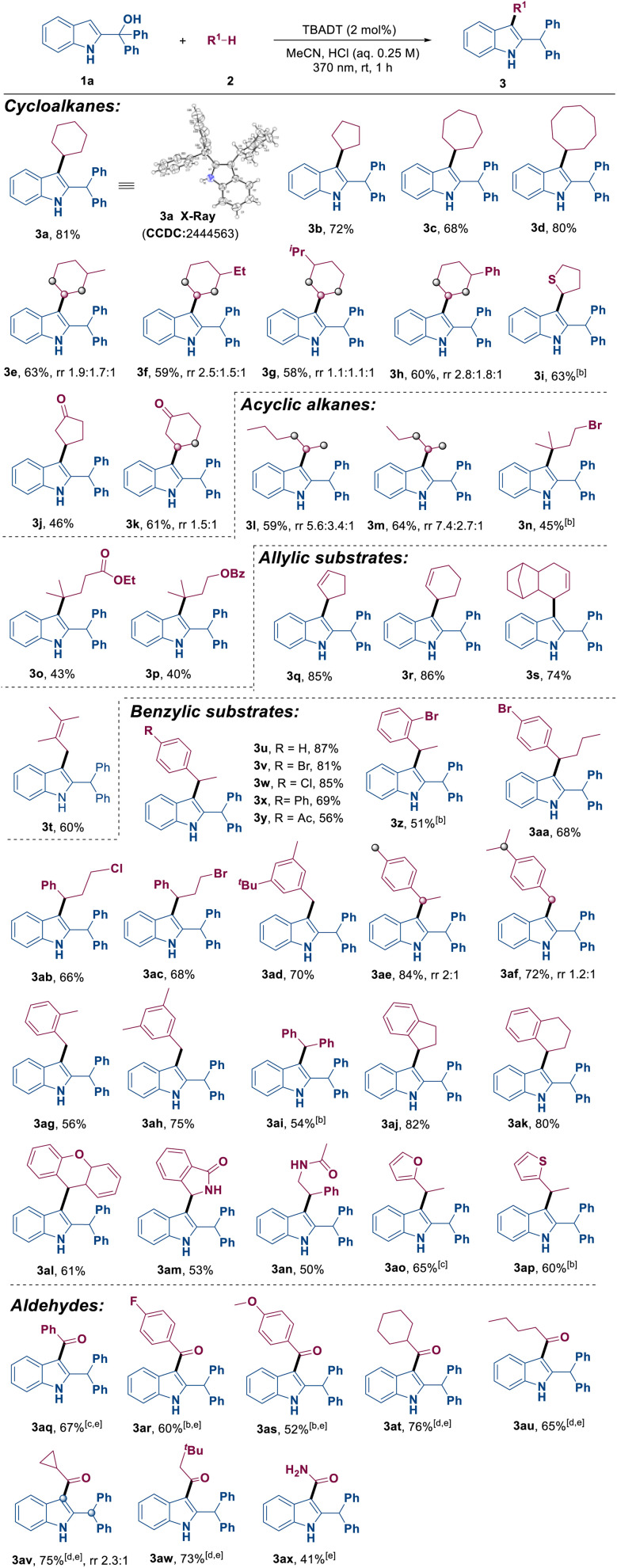

a Reaction conditions: 1a (0.1 mmol), 2 (1.0 mmol), TBADT (2 mol%), MeCN/HCl (0.85 mL: 0.15 mL, 0.25 M), 370 nm, 1 h, rt, isolated yields based on 1a. rr = regioisometric ratio.

b HCl (0.1 M).

c 4 h.

d TsOH (0.25 M).

e TsOH (0.1 M).

f 1a (0.1 mmol), 2 (0.3 mmol), 12 h.

Besides that, to our delight, both aromatic and aliphatic aldehydes (3aq–3ax) were found to be compatible with this transformation. This compatibility was achieved by extending the reaction time and varying the type and concentration of Brønsted acid.

Subsequently, we assessed the reactivity of 2-indolemethanol derivatives 1 with cyclohexane 2a, and a series of indolated products 3ay–3bo were obtained in moderate to excellent yields (Table 3). 2-Indolemethanols with different electronic properties, either due to different Ar groups or due to electron-donating or electron-withdrawing substituents on the indole ring, were all compatible with this reaction. The reaction produced the corresponding products 3ay–3bl with yields ranging from moderate to good. One should note that when the substrates contain F, Cl, Br, and OMe groups, this reaction may often lead to the formation of alkylated by-products at the aryl benzylic position (3ba–3bg and 3bk–3bl). A 2-indolemethanol derivative with an N–CH_3_ substituent was found to be a suitable substrate, yielding the expected product 3bm in 53% yield. Notably, when the reaction was performed using ethylbenzene and a 2-indolylmethanol derivative with isopropyl groups replacing the aryl groups, product 3bn was obtained with a yield of 56% and a rr (regioisomeric ratio) value of 1 : 1. Most importantly, 2-indolylmethanol bearing two distinct aryl groups efficiently afforded the desired product 3bo in 50% yield.

**Table 3:** Reaction of 2a with various 2-indolemethanols^a^

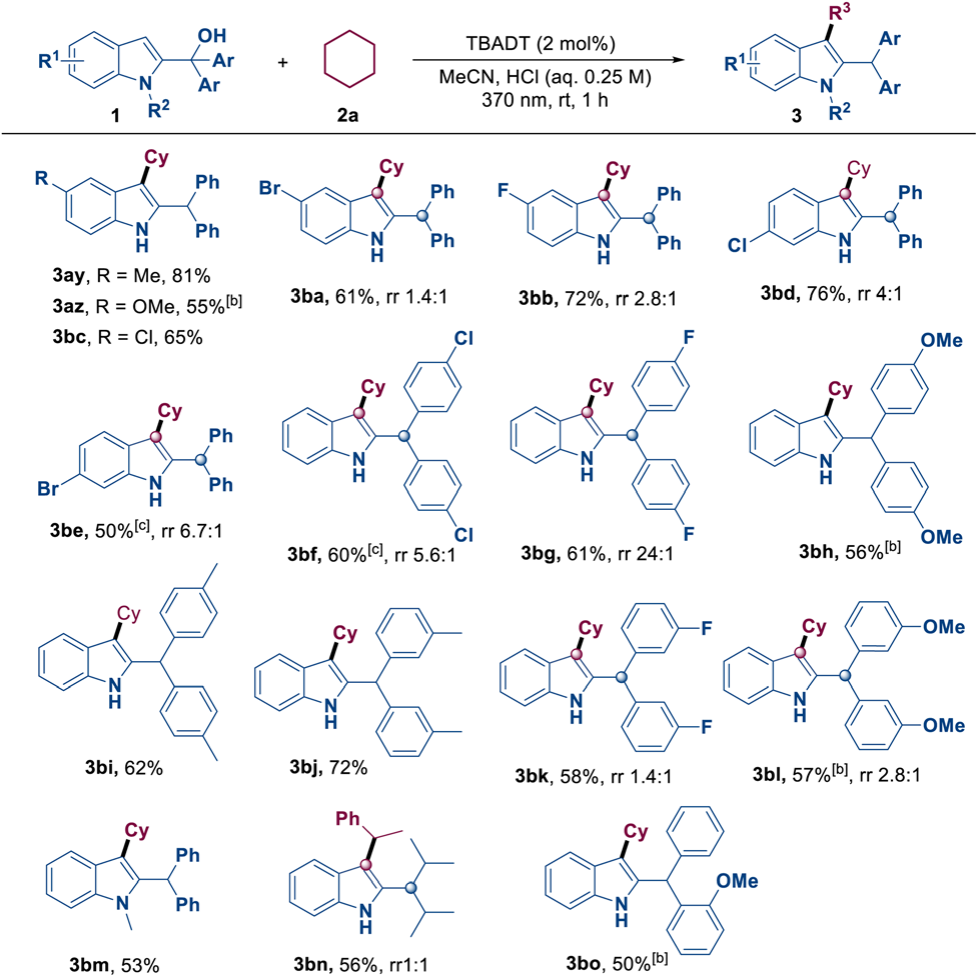

a Reaction conditions: 1 (0.1 mmol), 2a (1.0 mmol), TBADT (2 mol%), MeCN/HCl (0.85 mL: 0.15 mL, 0.25 M), 370 nm, 1 h, rt, isolated yields based on 1. rr = regioisometric ratio.

b MeCN/TsOH (0.85 mL: 0.15 mL, 0.25 M).

c MeCN/HCl (0.9 mL: 0.1 mL, 1.0 M).

d 4 h.

Furthermore, the reaction proceeded smoothly with natural products and drug molecules, including sclareolide, artemisinin and lurasidone, yielding the corresponding products 3bp–3bu in moderate yields (Table 4). For late-stage functionalizations, the C–H substrate was reduced to 3.0 equivalents; thus, a higher TBADT loading (5 mol%) was employed to offset the slower HAT kinetics and maintain efficient turnover. The successful indolylation of these substrates demonstrates the high robustness and reliability of the reaction and its broad applicability to a diverse array of chemical structures, particularly those found in biologically active compounds. The observed regioselectivity is highly consistent with established empirical trends for decatungstate-mediated HAT processes, where hydrogen abstraction preferentially occurs at the most sterically accessible and thermodynamically favored C–H bonds within the complex molecular frameworks.^12^

**Table 4:** Late-stage indolation of natural products and derivatives^a^

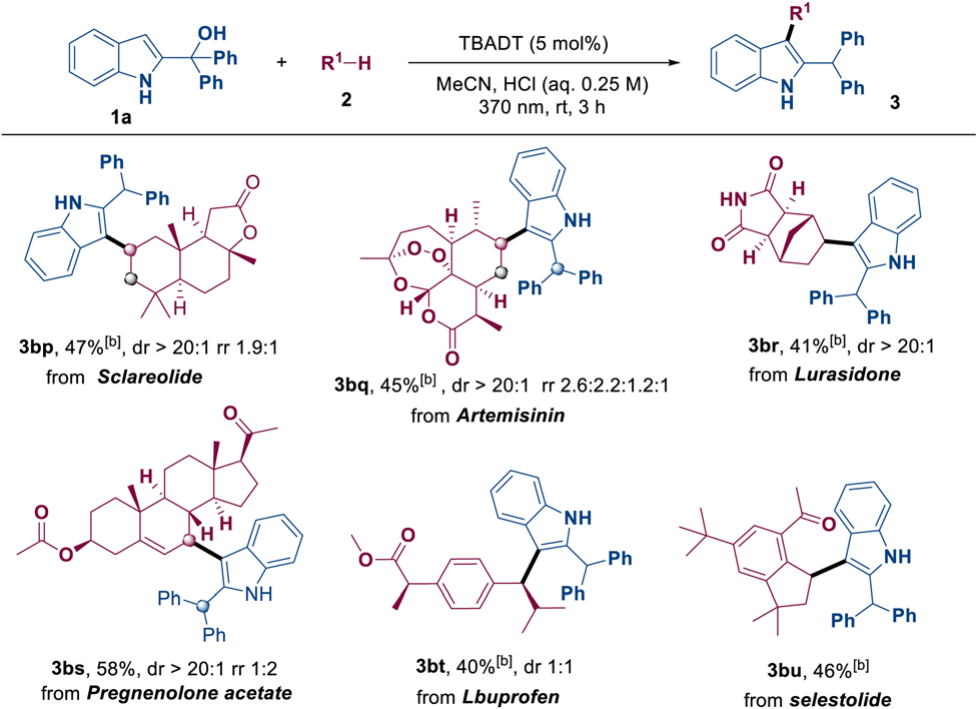

a Reaction conditions: 1a (0.1 mmol), 2 (0.3 mmol), TBADT (5 mol%), MeCN/TsOH (0.85 mL: 0.15 mL, 0.25 M), 370 nm, 3 h, rt, isolated yields based on 1a. dr = diastereomeric ratio.

b HCl (0.25 M).

c 6 h.

**Scheme 2:**
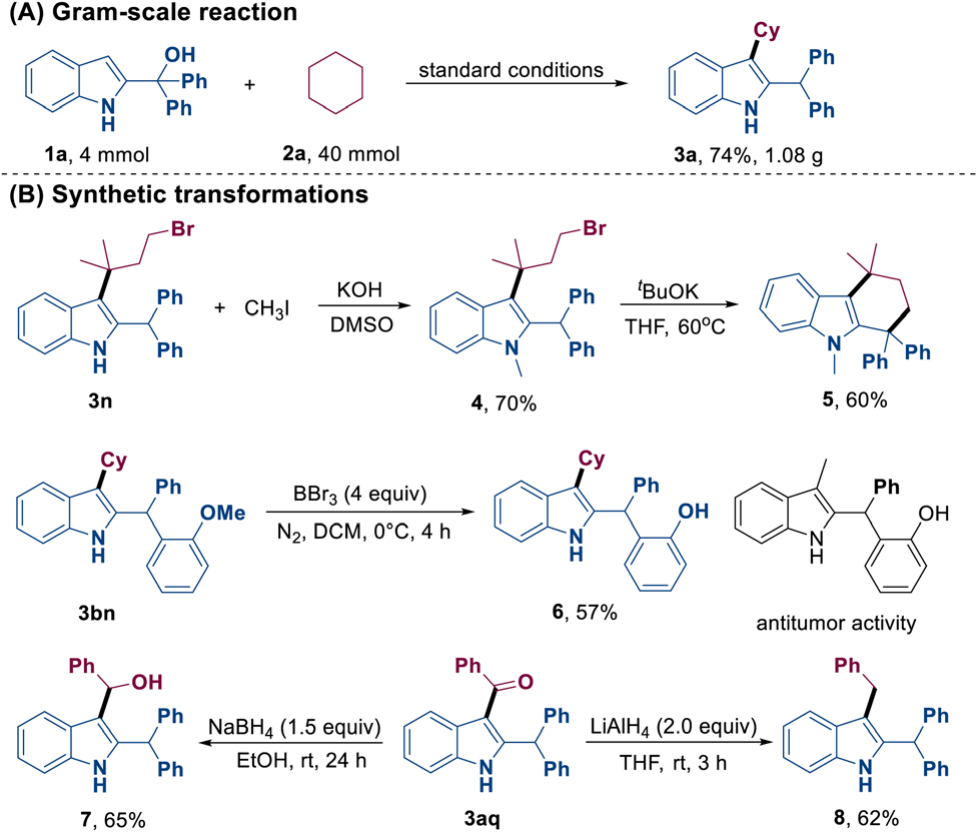
Gram-scale reaction.

A gram-scale reaction involving 4.0 mmol of 2-indolemethanol 1a with cyclohexane 2a was successfully conducted, which resulted in the formation of product 3a in 74% yield. This result demonstrated the feasibility of this protocol (Scheme 2A). Subsequently, straightforward transformations of the obtained products were studied (Scheme 2B). For instance, compound 5 could be synthesized from 3n through successive *N*-methylation and intramolecular cyclization. The demethylation of 3bn could lead to the formation of 6, which is presumably a bioactive compound. Moreover, treatment of 3aq with NaBH_4_ or LiAlH_4_ as the reducing agent afforded compounds 7 and 8, respectively.

**Scheme 3:**
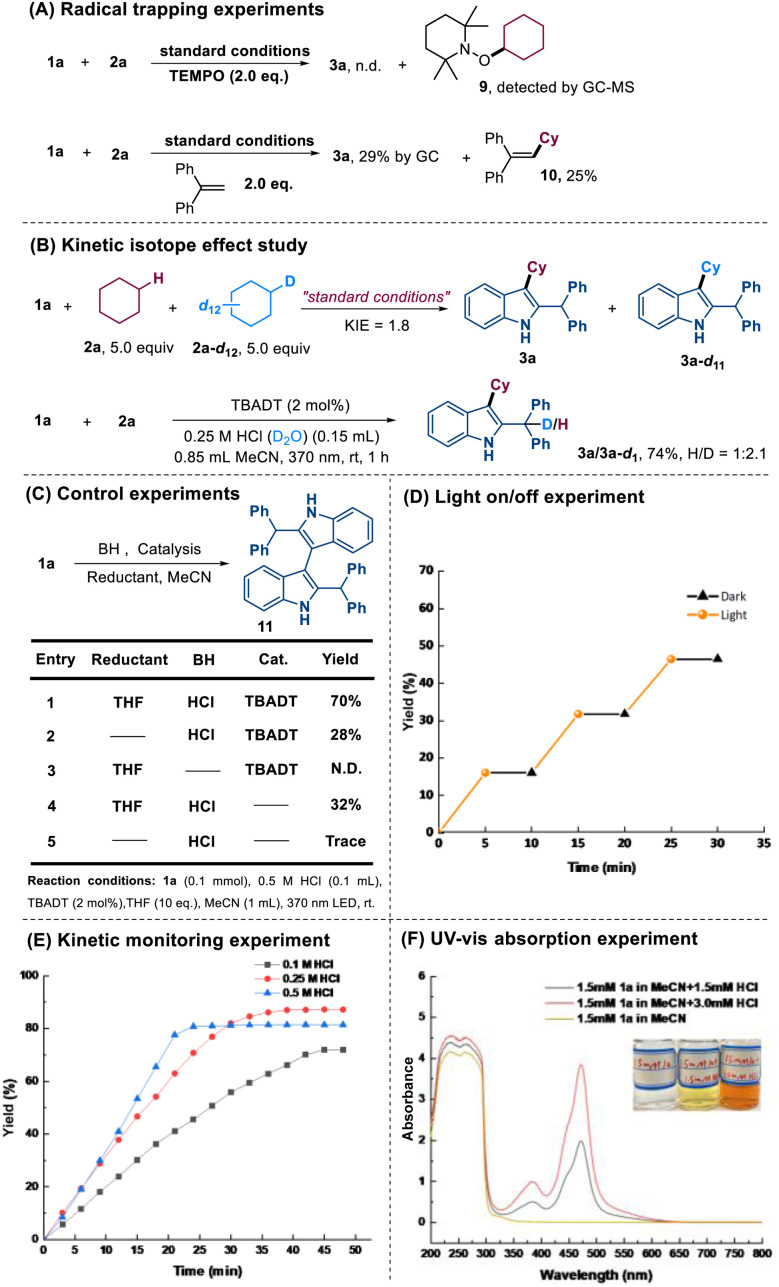
Control experiments.

To gain insights into the reaction mechanism, a series of mechanistic experiments were carried out (Scheme 3). Adding 2 equivalents of 2,2,6,6-tetramethyl-1-piperidinyloxy (TEMPO) as a radical scavenger completely inhibited the reaction. While product 3a was undetectable, GC-MS analysis revealed the formation of TEMPO-Cy 9. Using 1,1-diphenylethylene as a radical trapper afforded product 3a in only 29% yield, along with the trapped cyclohexyl radical adduct 10 (25% yield) (Scheme 3A). Based on these results, a cyclohexyl radical-involved pathway was confirmed. Next, we examined the kinetic isotope effect (KIE) *via* an intermolecular competition experiment. The observed KIE value of 1.8 indicated that C–H bond homolysis plays a non-negligible role in the rate-determining step. Further mechanistic insights were gained by employing an aqueous solution of HCl (0.25 M in D_2_O); product 3a/3a–d1 was isolated in 74% yield with an H/D ratio of 1 : 2.1 (Scheme 3B). This prominent solvent isotope effect suggested water as the predominant proton donor for the benzylic position of product 3a. As shown in Scheme 3C, a range of control experiments were conducted. Notably, the formation of a dimer product 11 was significantly enhanced when the reaction was conducted in the presence of THF (acting as a hydrogen atom donor/reductant), HCl, and photocatalyst TBADT. This observation suggests that the reduced photocatalyst species [W_10_O_32_]^5−^H^+^, generated *via* HAT from THF, engages in a single electron transfer (SET) with the acid-generated cationic intermediate. This reduction yields a persistent benzylic radical, which subsequently undergoes homocoupling to furnish the dimer 11. A light on–off experiment revealed that the reaction could only occur under illumination conditions (Scheme 3D). Kinetic studies revealed a noticeable acceleration of the reaction rate with increasing HCl concentration (Scheme 3E). These measurements were completed within 0.5–1.0 hours, demonstrating significantly faster kinetics than most reported HAT-mediated transformations, which generally require more than 10 hours for completion. According to the reported literature, this phenomenon can be explained by the dual catalytic role of HCl in this system, both facilitating the formation of carbocation intermediates and synergistically promoting the HAT process with TBADT.^13^ Furthermore, UV-vis spectroscopic analysis was performed to investigate the electronic properties of 1a under acidic conditions (Scheme 3F). While compound 1a itself exhibited no detectable absorption in the visible light region, the addition of HCl induced obvious absorption bands in this spectral region. Notably, the absorption intensity showed a clear concentration-dependent enhancement with increasing HCl concentration.

**Scheme 4:**
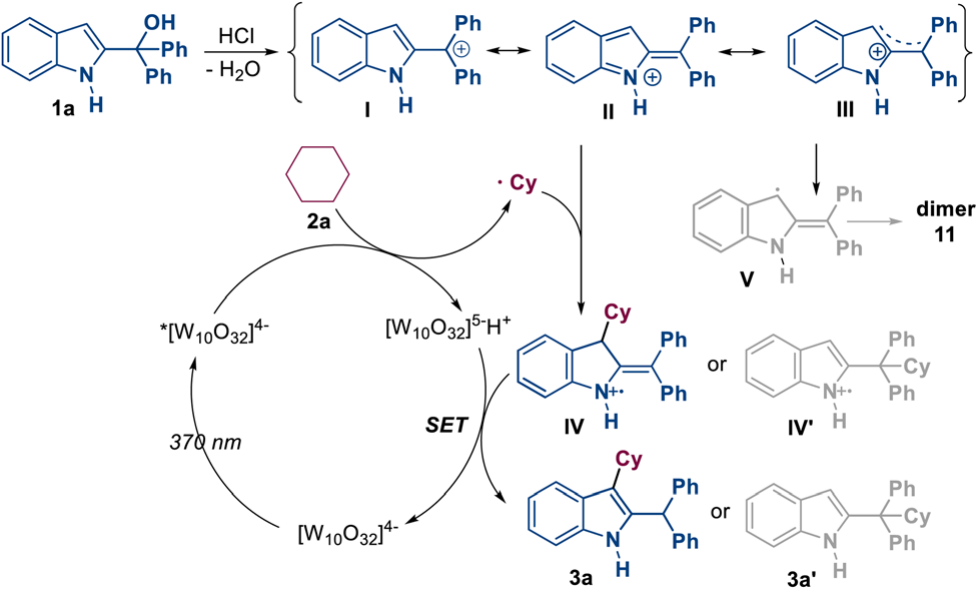
Proposed mechanism.

Based on the mechanistic verification presented above and previous reports,^13,14^ a plausible mechanism for the photoredox and Brønsted acid dual-catalyzed C3-alkylation of indoles is proposed in Scheme 4. On the one hand, in the presence of HCl, 2-indolemethanol 1 transforms into resonant intermediates, including carbocation I, vinyliminium II and delocalized cation III. On the other hand, photoexcitation of [W_10_O_32_]^4−^ generates its excited state *[W_10_O_32_]^4−^, which can directly abstract a hydrogen atom from R^5^–H 2, leading to the formation of alkyl radical R^5^˙ and [W_10_O_32_]^5−^H^+^. The alkyl radical R^5^˙ can be trapped by the vinyliminium intermediate II, yielding intermediates IV or IV′. Following the SET process between [W_10_O_32_]^5−^H^+^ and intermediates IV or IV′, two distinct products are generated. 3a′ is produced directly from the SET process, whereas 3a is obtained after an intramolecular rearrangement of the intermediate formed in the SET process. Additionally, the delocalized cation III can be transformed into a radical intermediate V*via* an SET process with [W_10_O_32_]^5−^H^+^, and a dimer product 11 is further produced from intermediate V.

## Conclusions

In summary, we report a synergistic catalytic protocol that merges decatungstate photocatalysis and Brønsted acid catalysis to achieve the direct C–H indolation of alkanes and aldehydes with 2-indolylmethanols. This method effectively overcomes the inherent polarity mismatch through a radical-polar crossover strategy, enabling the direct coupling of 2-indolylmethanols with a broad range of radical precursors with high regioselectivity. This protocol was applied to 73 examples, notably involving the late-stage functionalization of complex natural products and pharmaceuticals. Mechanistic investigations reveal the critical dual role of the Brønsted acid: it not only facilitates the dehydration of 2-indolylmethanols to generate the electrophilic cationic acceptor but also synergistically accelerates the decatungstate-mediated HAT process. Given the ubiquity of indole scaffolds in pharmaceutically important compounds, we anticipate that this methodology will find widespread application in medicinal chemistry and synthesis.

## Author contributions

D. Z. conceptualized the project. G. P. and Y. D. performed the experiments and analyzed the results. M. Z., Y. H., H. C. and X. W. assisted in performing the experiments. G. Q., Y. A., and D. Z. supervised and directed the project. Y. A. and D. Z. wrote the manuscript.

## Conflicts of interest

There are no conflicts to declare.

## Supplementary Material

SC-OLF-D6SC01827K-s001

SC-OLF-D6SC01827K-s002

## Data Availability

The data supporting this article have been included as part of the supplementary information (SI). Supplementary information: further details of the experimental procedures, ^1^H and ^13^C NMR spectra, HRMS data, and X-ray crystallographic data for 3a. See DOI: https://doi.org/10.1039/d6sc01827k. CCDC 2444563 contains the supplementary crystallographic data for this paper.^15^
