# Supplementary material for: Synergistic decatungstate and Brønsted acid catalysis enables direct C–H indolation of alkanes and aldehydes
Source: Chem Sci. 2026 Apr 27;17(23):11528–37. doi: 10.1039/d6sc01827k (PMC13133923; doi:10.1039/d6sc01827k)
Supplement: SC-017-D6SC01827K-s001 [file SC-017-D6SC01827K-s001.pdf]

Supplementary Information (SI) for Chemical Science.
This journal is © The Royal Society of Chemistry 2026

Supporting Information

Synergistic Decatungstate and Brønsted Acid Catalysis Enables Direct C－H Indolation of Alkanes and Aldehydes

Gangqi Peng,[+,a] Yangling Deng,[+,a,b] Mengxuan Zhang,[a] Yuxuan He,[a] Hao Cheng,[a] Xinbin Wang,[a] Yuanyuan An,*[,a] Guanyinsheng Qiu,*[,b] and Danqing Zheng*[,a]

[a] *State Key Laboratory of Materials-Oriented Chemical Engineering, College of Chemical Engineering, Nanjing Tech University, Nanjing 211816, China. Email: zhengd@njtech.edu.cn*
[b] *College of Biological, Chemical Sciences and Engineering, Jiaxing University, Jiaxing 314001, Zhejiang, China. E-mail: qiuguanyinsheng@mail.zjxu.edu.cn*

Table of Contents

1. **General experimental methods** **S2**
2. **General experimental procedure** **S3**
3. **Gram-scale experiment** **S4**
4. **Synthetic transformations** **S5**
5. **Mechanistic studies** **S6**
6. **X-Ray crystallographic data of product 3a** **S13**
7. **Unsuccessful Substrates** **S16**
8. **Analytical data for products** **S16**
9. **References** **S56**
10. **NMR spectra for all compounds** **S57**

1. General experimental methods

All experiments were carried out under nitrogen atmosphere using anhydrous solvents. Unless otherwise noted, all commercially available reagents and solvents were purchased and used as received without further purification. All reactions were monitored by thin layer chromatography (TLC) carried out on silica gel plates, visualisation was by ultraviolet fluorescence. Column chromatography was performed on silica gel (300-400 mesh). ^{1}H NMR, ^{13}C NMR and ^{19}F NMR spectra were recorded on a Bruker Avance-400 spectrometer in $CDCl_3$ as solvents at room temperature. Chemical shifts are expressed in parts per million (ppm) and referenced to the residual solvent peak. Splitting patterns are indicated as follows: br, broad; s, singlet; d, doublet; m, multiplet, q, quartet, with all coupling constants (J) reported in Hertz (Hz). The high-resolution mass spectra (HRMS) were recorded on an IonSpec FT-ICR mass spectrometer with ESI resource. GC-MS analysis was analyzed with GC-MS (Agilent 7890B-5977A, USA). All reactions irradiated with a 40 W Kessil UV light lamp (PR160L-370nm). Substrates **1** were synthesized according to the literature methods [1-5]. The photoreactions were set up as below:

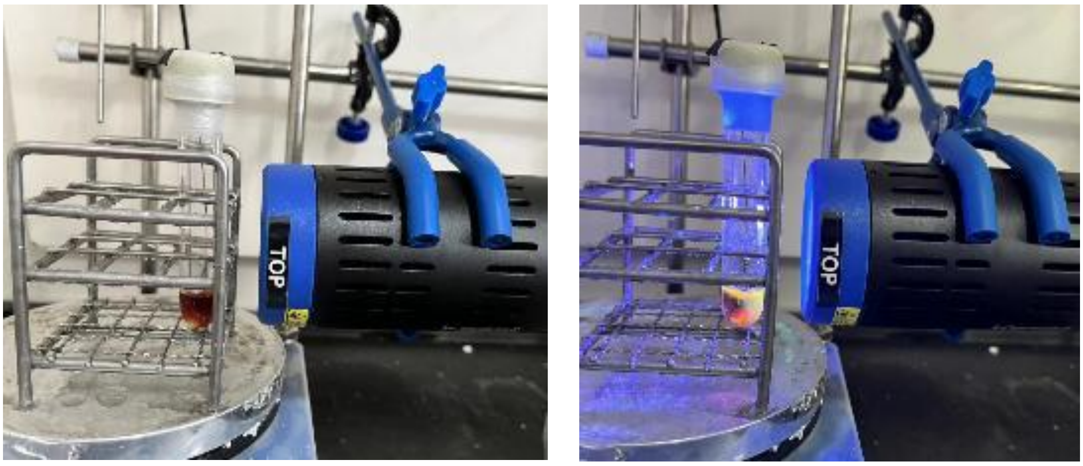

Supplementary Figure 1. Setup for the photoreactions

2. General experimental procedure

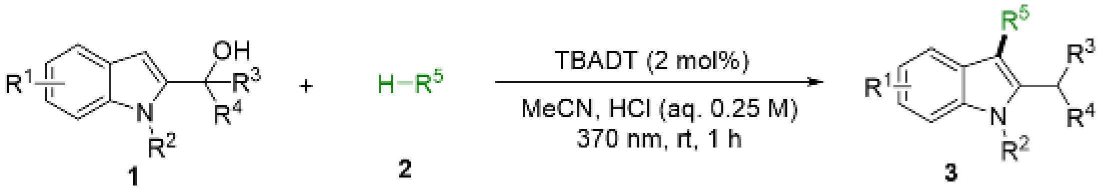

Procedures for the C–H indolation of alkanes followed **General Procedure A**. A 10 mL quartz reaction tube equipped with a magnetic stirrer was charged with 2-indolylmethanol **1** (0.1 mmol, 1 equiv.), alkane substrates **2** (1 mmol, 10 equiv.) and TBADT (2 mol%, 6.6 mg). The tube was then carefully evacuated and backfilled with N_2 for three times. Subsequently, anhydrous MeCN (0.85 mL) and 0.25 mol/L HCl (0.15 mL) were added *via* syringe. The reaction mixture was stirred under the irradiation of a 40W Kessil 370 nm lamp for 1 h. Upon completion of the reaction as indicated by TLC, the reaction mixture was directly purified by flash column chromatography.

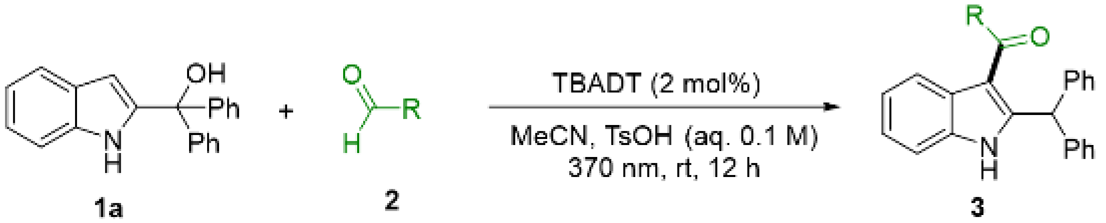

Procedures for the C–H indolation of aldehydes followed **General Procedure B**. A 10 mL quartz reaction tube equipped with a magnetic stirrer was charged with 2-indolylmethanol **1a** (0.1 mmol, 1 equiv.), aldehyde substrates **2** (0.3 mmol, 3 equiv.) and TBADT (2 mol%, 6.6 mg). The tube was then carefully evacuated and backfilled with N_2 for three times. Subsequently, anhydrous MeCN (0.85 mL) and 0.1 mol/L TsOH(0.15 mL) were added *via* syringe. The reaction mixture was stirred under the irradiation of a 40W Kessil 370 nm lamp for 12 h. Upon completion of the reaction as indicated by TLC, the reaction mixture was directly purified by flash column chromatography.

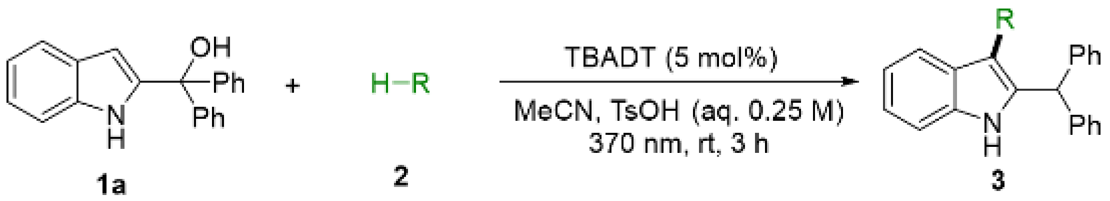

Procedures for the late-stage indolation of natural products and derivatives followed **General Procedure C**. A 10 mL quartz reaction tube equipped with a magnetic stirrer was charged with 2-indolylmethanol **1a** (0.1 mmol, 1 equiv.), natural product derivatives **2** (0.3 mmol, 3 equiv.) and TBADT (5 mol%, 16.5 mg). The tube was then carefully evacuated and backfilled with N_2 for three times. Subsequently, anhydrous MeCN (0.85 mL) and 0.25 mol/L TsOH (0.15 mL) were added *via* syringe. The reaction mixture was stirred under the irradiation of a 40W Kessil 370 nm lamp for 3 h. Upon completion of the reaction as indicated by TLC, the reaction mixture was directly purified by flash column chromatography.

3. Gram-scale experiment

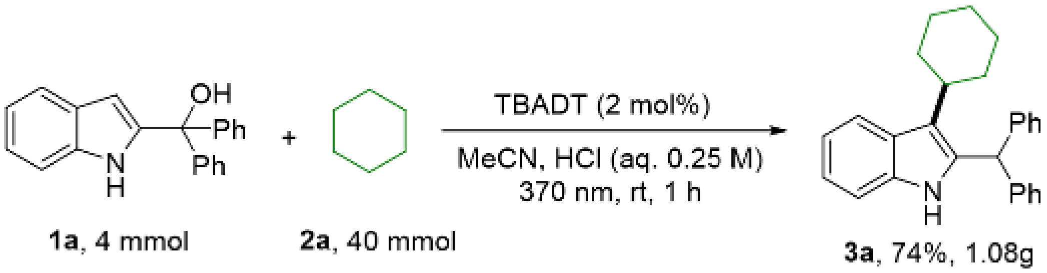

A 100 mL round bottom flask equipped with a magnetic stirrer was charged with 2-indolylmethanol **1a** (4 mmol, 1.19 g), cyclohexane **2a** (40 mmol, 4 mL) and TBADT (0.08 mmol, 264.0 mg). The tube was then carefully evacuated and backfilled with N_2 for three times. Subsequently, anhydrous MeCN (34.0 mL) and 0.25mol/L HCl (6.0 mL) were added via syringe. The reaction mixture was stirred under the irradiation of a 40W Kessil 370 nm lamp for 1 h. Upon completion of the reaction as indicated by TLC, the reaction mixture was concentrated *in vacuo*. The residue was purified by flash chromatography on silica gel to give the desired product **3a** as a white solid in 74% yield (1.08 g).

4. Synthetic transformations

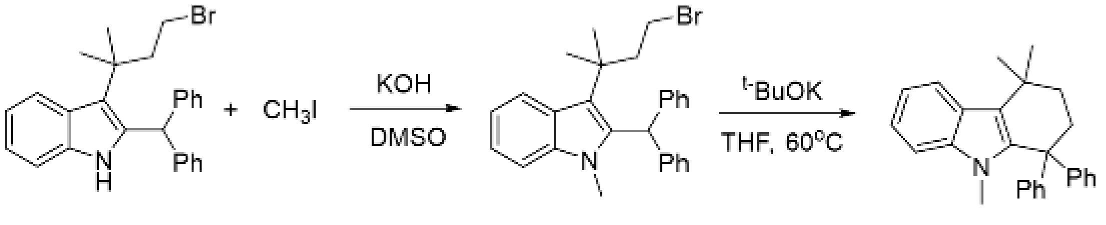

3n **4**, 70% **5**, 60%

A mixture of potassium hydroxide (0.2 mmol, 11.2 mg), **3n** (0.1 mmol, 43.2 mg), iodomethane (0.2 mmol, 12.6 μL) and DMSO (1.0 mL) was stirred in a 10 mL glass tube at room temperature for 12 h. The mixture was then extracted with H_2O and EtOAc (3×2 mL). The combined organic extracts were dried over anhydrous Na_2SO_4. After concentration under reduced pressure, the crude residue was purified by flash column chromatography (EtOAc: Petroleum = 1:50) on silica gel to afford the pure compound **4**.

The potassium *tert*-butoxide (0.2 mmol, 22.4 mg), **4** (0.1 mmol, 44.6 mg) and THF (1.0 mL) were added to a 10 mL glass tube. Then, the reaction mixture was stirred at 60 °C for 12 h. Upon completion of the reaction as indicated by TLC, the reaction mixture was directly purified by flash chromatography (EtOAc: Petroleum = 1:100) on silica gel to afford pure compound **5**.

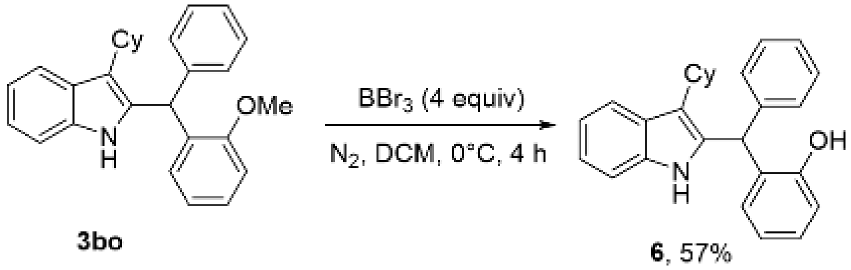

Under N_2 atmosphere, boron tribromide (0.4mmol, 38.5 μL) was added dropwise to a solution of compound **3bo** (0.1 mmol, 39.5 mg) in DCM (1 mL) at 0 °C. Then, the reaction mixture was allowed to warm up to room temperature and stirred for 4 h. After the reaction completed, the mixture was then extracted with H_2O and EtOAc (3×2 mL). The combined organic extracts were dried over anhydrous Na_2SO_4 and concentrated

under reduced pressure. The crude residue was purified by flash column chromatography (EtOAc: Petroleum = 1:8) on silica gel to afford the pure compound **6**.

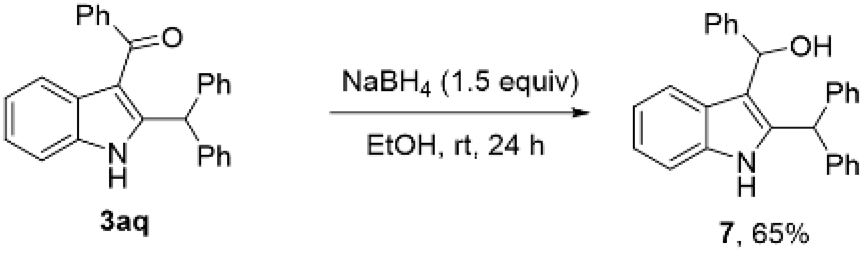

The sodium borohydride (0.3 mmol, 11.3 mg), **3aq** (0.2 mmol, 77.4 mg) and EtOH (1.0 mL) were added to a 10 mL glass tube. Then, the reaction mixture was stirred at room temperature for 24 h. After the reaction completed, the mixture was extracted with H_2O and EtOAc (3×2 mL). The combined organic extracts were dried over anhydrous Na_2SO_4 and concentrated under reduced pressure. The residue was purified by flash column chromatography (EtOAc: Petroleum = 1:8) on silica gel to afford pure compound **7**.

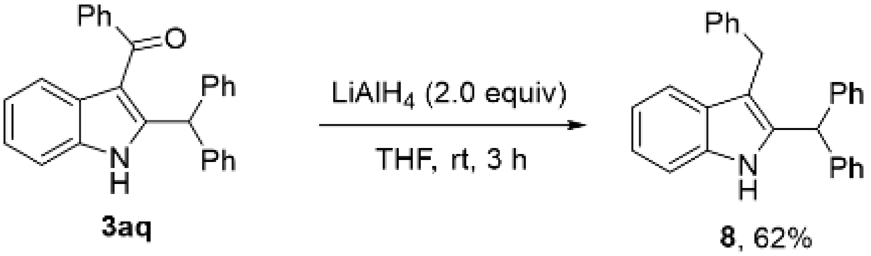

The lithium aluminum hydride (0.4 mmol, 15.2 mg), **3aq** (0.2 mmol, 77.4 mg) and THF (1.0 mL) were added to a 10 mL glass tube. Then, the reaction mixture was stirred at room temperature for 3 h. After the reaction completed, the mixture was extracted with H_2O and EtOAc (3×2 mL). The combined organic extracts were dried over anhydrous Na_2SO_4 and concentrated under reduced pressure. The residue was purified by flash column chromatography (EtOAc: Petroleum = 1:50) on silica gel to afford pure compound **8**.

5. Mechanistic studies

5.1 Radical trap experiments

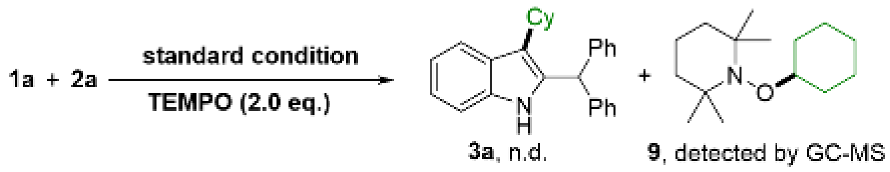

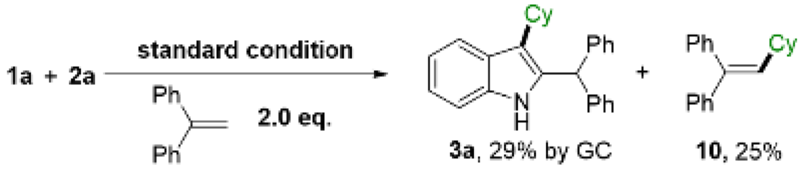

A 10 mL quartz reaction tube equipped with a magnetic stirrer was charged with 2-indolylmethanol **1a** (0.1 mmol, 29.9 mg), cyclohexane **2a** (1 mmol, 0.1 mL), TEMPO (2.0 equiv.) and TBADT (2 mol%, 6.6 mg). Then, the tube was carefully evacuated and backfilled with N_2 for three times. Afterwards, anhydrous MeCN (1.0 mL) and 0.25mol/L HCl (0.15 mL) were added *via* syringe. The reaction mixture was stirred under the irradiation of a 40W Kessil 370 nm lamp for 0.5 h. After the reaction was completed, product **3a** could not be detected, and TEMPO-Cy **9** was detected by GC-MS.

A 10 mL quartz reaction tube equipped with a magnetic stirrer was charged with 2-indolylmethanol **1a** (0.1 mmol, 29.9 mg), cyclohexane **2a** (1 mmol, 0.1 mL), 1,1-Diphenylethylene (2.0 equiv.) and TBADT (2 mol%, 6.6 mg). Then, the tube was carefully evacuated and backfilled with N_2 for three times. Afterwards, anhydrous MeCN (1.0 mL) and 0.25mol/L HCl (0.15 mL) were added via syringe. The reaction mixture was stirred under the irradiation of a 40W Kessil 370 nm lamp for 0.5 h. After the reaction was completed, product **3a** was monitored by GC and the remaining material was purified by flash chromatography on silica gel to give product **10**.

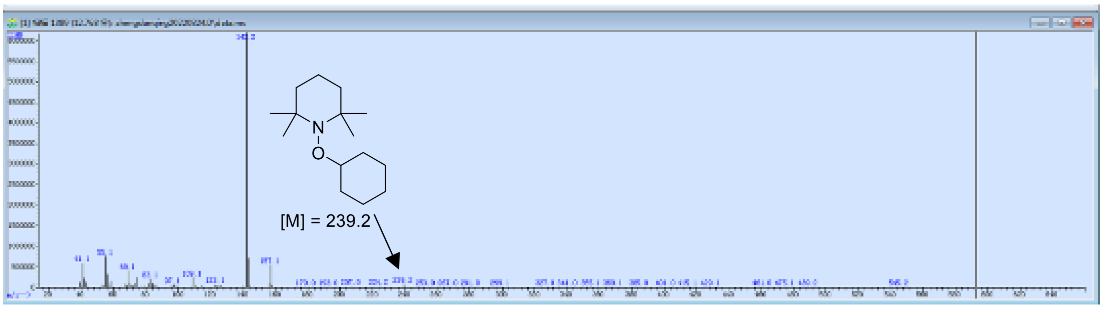

Supplementary Figure 2. Radical trapping with TEMPO.

5.2 Kinetic isotope effect study

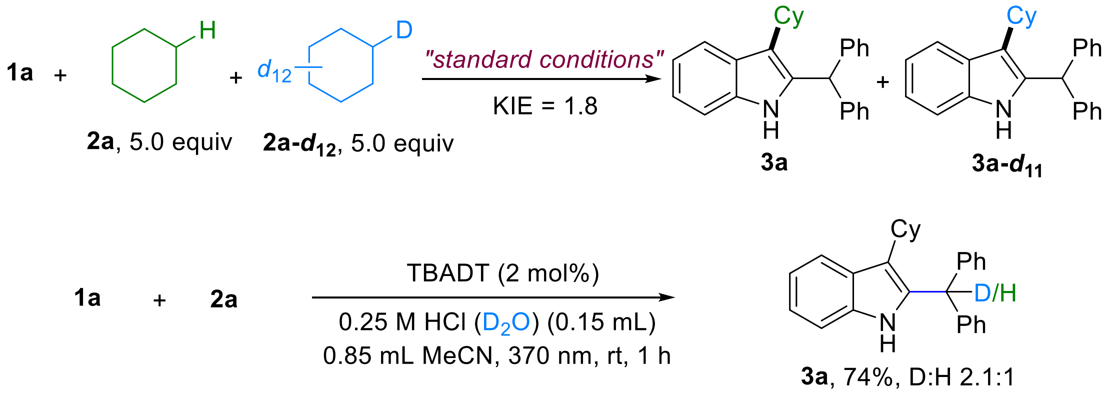

A 10 mL quartz reaction tube equipped with a magnetic stirrer was charged with 2-indolylmethanol **1a** (0.1 mmol, 29.9 mg), cyclohexane **2a** (0.5 mmol, 0.05 mL), cyclohexane **2a-*d*12** (0.5 mmol, 0.05 mL) and TBADT (2 mol%, 6.6 mg). Then, the tube was carefully evacuated and backfilled with N_2 for three times. Afterwards, anhydrous MeCN (1.0 mL) and 0.25mol/L HCl (0.15 mL) were added *via* syringe. The reaction mixture was stirred under the irradiation of a 40W Kessil 370 nm lamp for 1 h. Upon completion of the reaction as indicated by TLC, the reaction mixture was directly purified by flash column chromatography on silica gel (EtOAc: Petroleum = 1:50) to give the pure products **3a** and **3a-*d*11**.

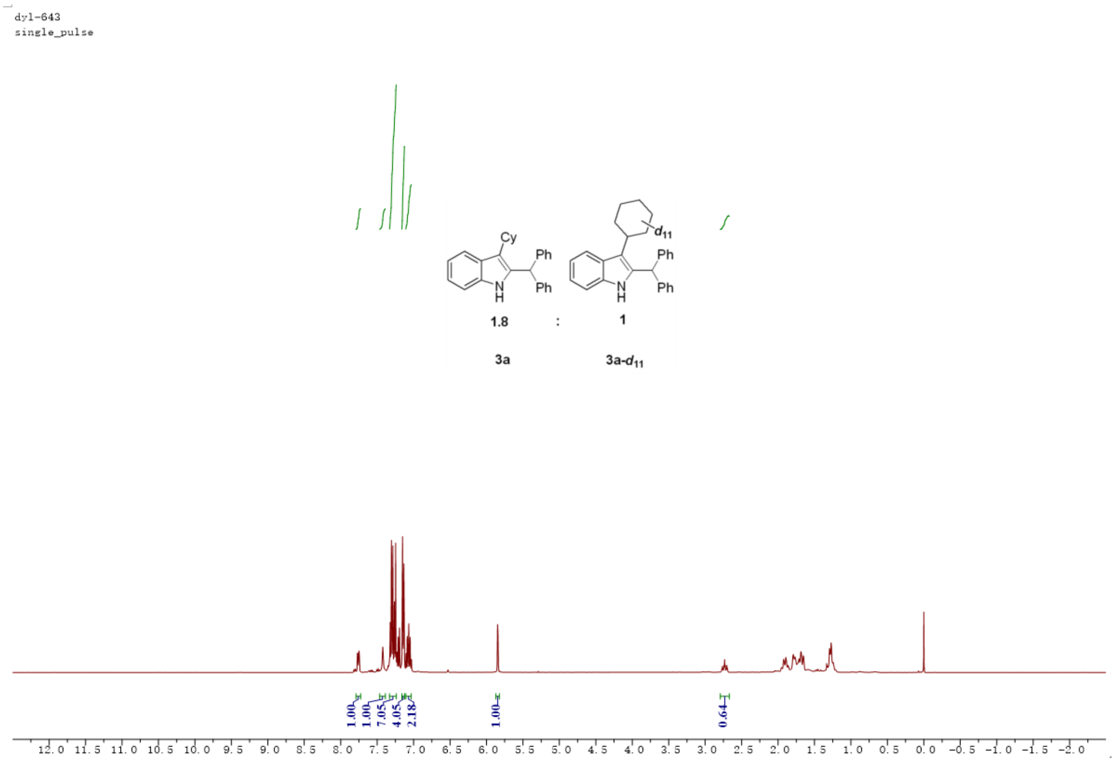

Supplementary Figure 3. KIE experiment involving 5 equivalents of cyclohexane

and 5 equivalents of d_{12} cyclohexane.

A 10 mL quartz reaction tube equipped with a magnetic stirrer was charged with 2-indolylmethanol **1a** (0.1 mmol, 29.9 mg), cyclohexane **2a** (1.0 mmol, 0.1 mL) and TBADT (2 mol%, 6.6 mg). Then, the tube was carefully evacuated and backfilled with N_2 for three times. Afterwards, anhydrous MeCN (1.0 mL) and 0.25 mol/L HCl (D_2O) (0.15 mL) were added via syringe. The reaction mixture was stirred under the irradiation of a 40W Kessil 370 nm lamp for 0.5 h. Upon completion of the reaction as indicated by TLC, the reaction mixture was directly purified by flash column chromatography on silica gel (EtOAc: Petroleum = 1:50) to give the pure products **3a**.

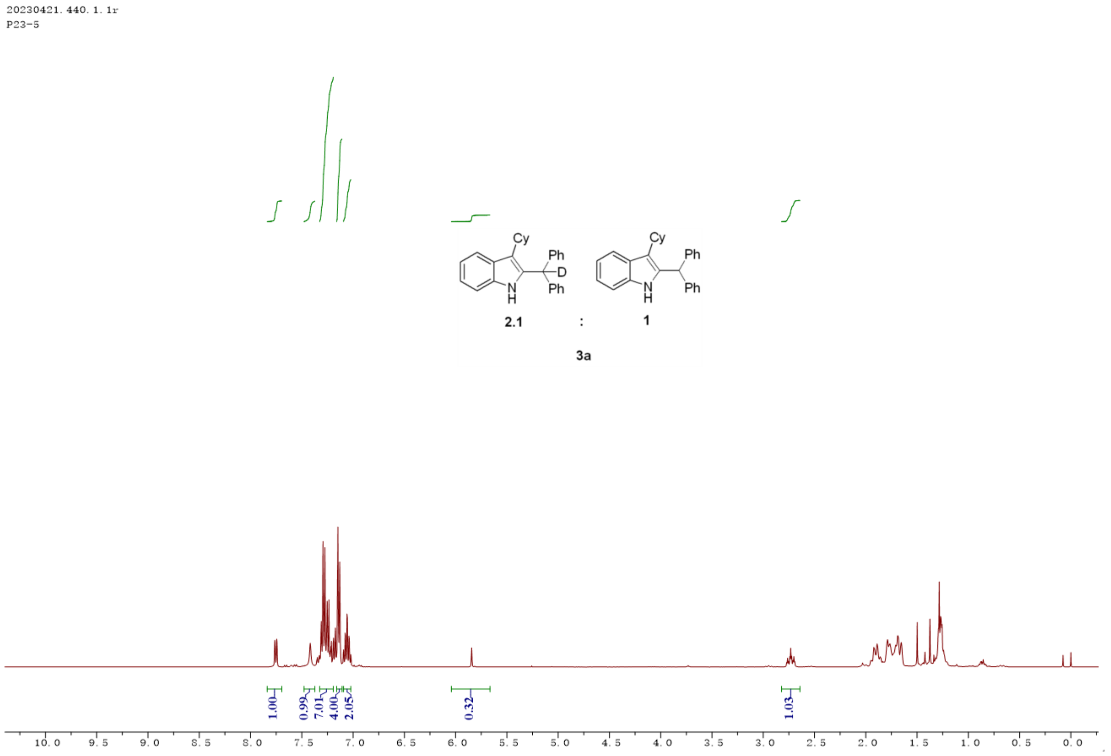

Supplementary Figure 4.

5.3 Light On/Off Experiments

Following the general procedure (0.3 mmol scale, 3 mL solvent, N_2), the reaction mixture was stirred and irradiated cyclically with a 40W Kessil 370 nm lamp (5 min on / 5 min off) for a total 30 minutes. The reaction progress was monitored by GC analysis every 5 minutes.

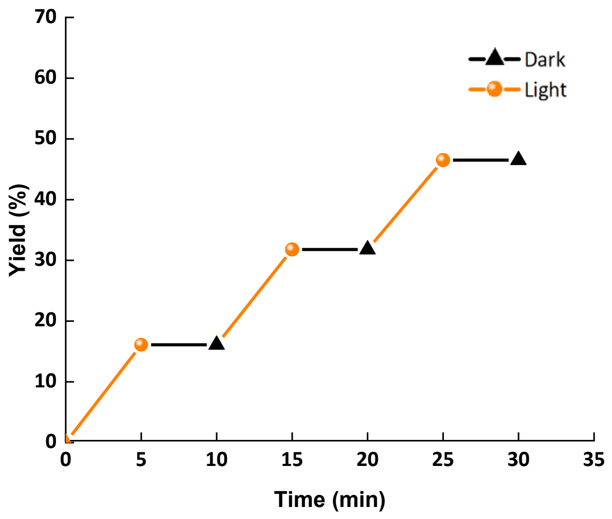

Supplementary Figure 5. Light on/off experiment.

5.4 Determination of the Reaction Order in HCl

Three reactions were set up identically with 2-indolylmethanol **1a** (0.1 mmol, 29.9 mg), cyclohexane **2a** (1.0 mmol, 0.1 mL) and TBADT (2 mol%, 6.6 mg) in MeCN (1.0 mL), differing only in the concentration of HCl (0.15 mL) added (0.1 M, 0.25 M, or 0.5 M). The reaction rate was observed to increase with [HCl]; however, no positive correlation was observed between the acid concentration and the yield.

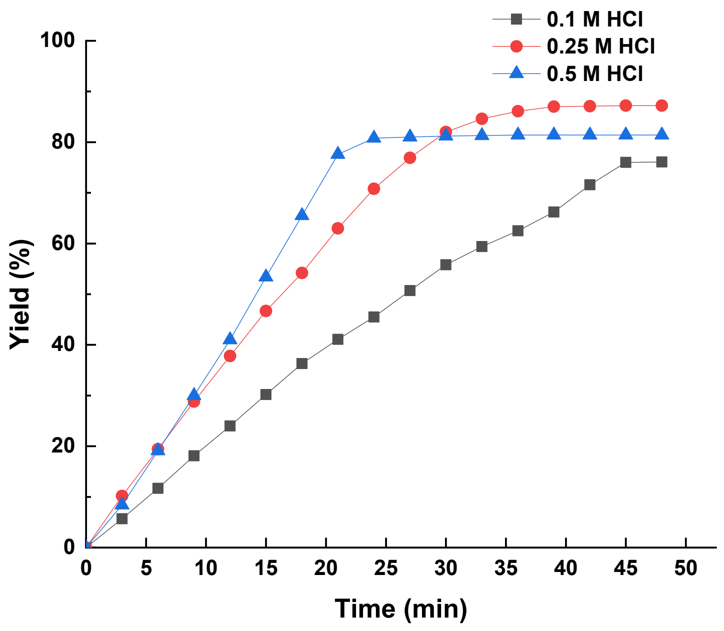

Supplementary Figure 6. Kinetic experiment.

5.5 UV/Vis Absorption Spectroscopy

As outlined in **Supplementary Figure 7**, the UV/Vis absorption spectra of **1a** were collected at different HCl concentrations, showing a significant enhancement in absorption with increasing [HCl].

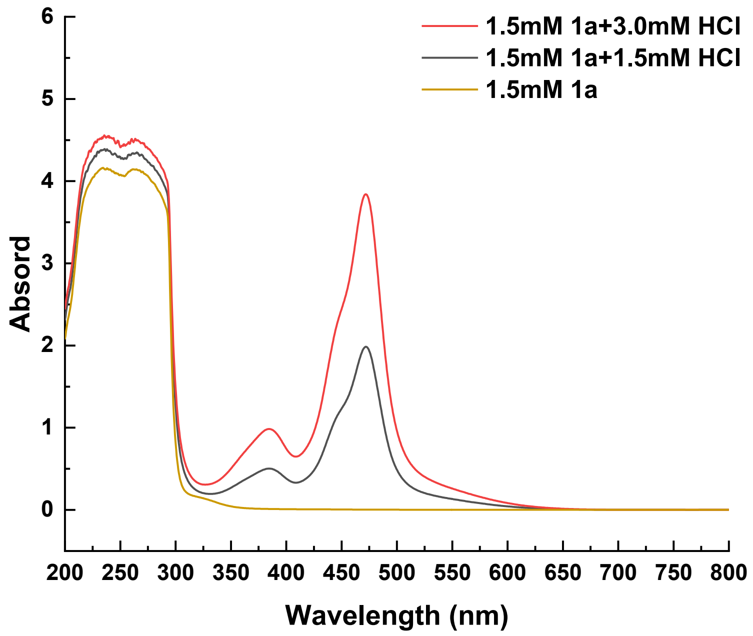

Supplementary Figure 7. UV/Vis absorption spectra of **1a** (1.5 mM) in MeCN recorded at varying concentrations of HCl.

5.6 Emission Quenching Experiments (Stern–Volmer Studies)

Stern-Volmer luminescence quenching analysis was conducted using a PerkinElmer FL 6500 Fluorescence Spectrophotometer at 25 °C. The following parameters were employed: Excitation Slit (nm) = 5, Emission Slit (nm) = 20, Emission Scan Speed (nm/min) = 240, Response Width (nm) = 10. The acetonitrile solution of TBADT (0.3 mM) were excited at λ_{ex} = 324 nm and the emission was recorded at 380 nm. For each quenching experiment, 10 μL of the quenching reagent were titrated to a solution (2.0 mL) of TBADT in a screw-top 10.0 mm quartz cuvette, $\mathbf{I_0}$ is the luminescence intensity without the quencher, **I** is the intensity in the presence of the quencher. The results are listed below:

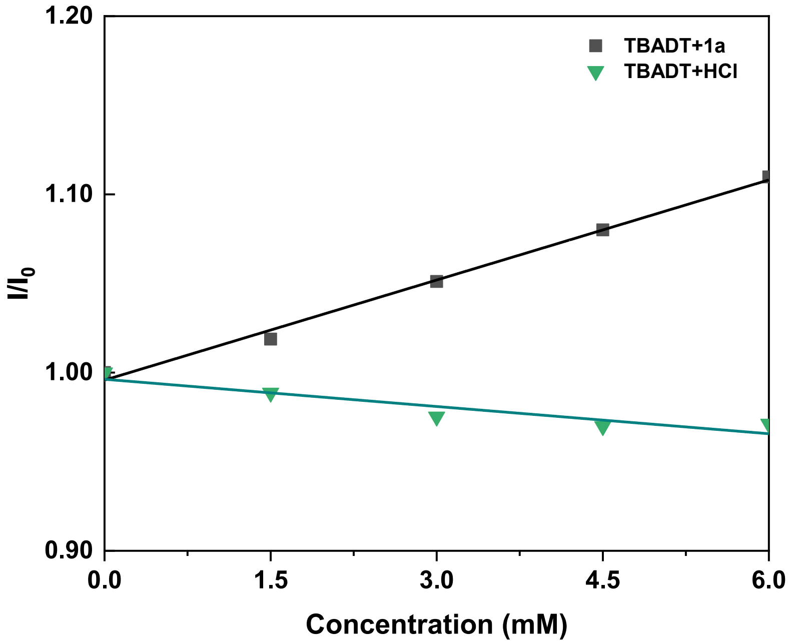

Supplementary Figure 8. Stern-Volmer plot of TBADT (0.3 mM) in MeCN recorded at varying concentrations of **1a** and HCl.

5.7 Fluorescence Emission

As outlined in **Supplementary Figure 9**, the fluorescent emission spectra of TBADT were collected at varying concentrations of **1a**, showing a significant enhancement in emission intensity with increasing concentration of **1a**.

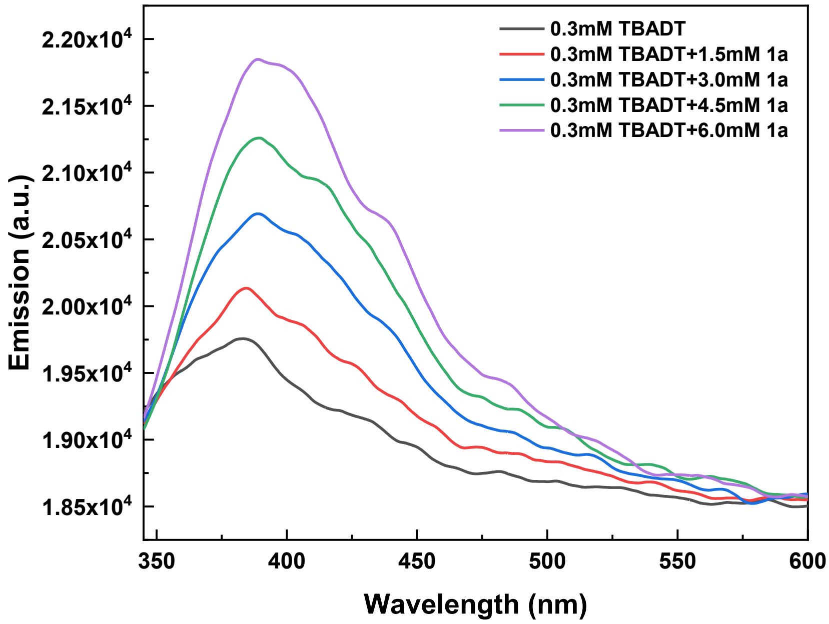

Supplementary Figure 9. Fluorescence Emission of TBADT (0.3 mM) in MeCN recorded at varying concentrations of **1a**.

6. X-Ray crystallographic data of product 3a

The crystal structure **3a** has been deposited at the Cambridge Crystallographic Data Centre and allocated the deposition number: CCDC 2444563. (The method for crystal growth: Dissolve 26 mg product in a small amount of dichloromethane, not more than 0.2 mL, then add about 5 mL of n-hexane. Not completely sealed, placed in a cabinet at room temperature, after about one week to get one cuboid single crystal.)

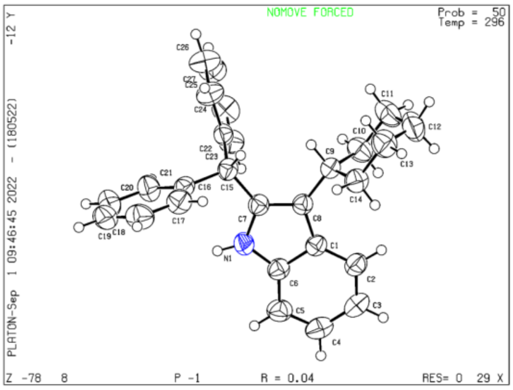

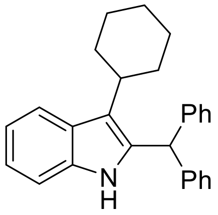

Bond precision: C-C = 0.0023 Å Wavelength: 0.71073

Cell: a=8.588(2) b=11.038(3) c=11.493(3)

alpha=105.954(3) beta=91.575(3) gamma=92.728(3)

Temperature: 296 K

	Calculated	Reported
Volume	1046.1(5)	1046.1(5)
Space group	P -1	P -1
Hall group	-P 1	-P 1
Moiety formula	$C_{27}H_{27}N$	$C_{27}H_{27}N$
Sum formula	$C_{27}H_{27}N$	$C_{27}H_{27}N$
Mr	365.50	365.49
Dx, g cm^{-3}	1.161	1.161
Z	2	2
Mu (mm^{-1})	0.066	0.066
F000	392.0	392.0
F000'	392.13	
h, k, lmax	10, 13, 13	10, 13, 13
Nref	3676	3642
Tmin, Tmax	0.991, 0.993	0.991, 0.993
Tmin'	0.991	

Correction method = # Reported T Limits: Tmin=0.991 Tmax=0.993 AbsCorr = MULTI-SCAN

Data completeness = 0.991 Theta(max) = 25.000

R(reflections) = 0.0409(2925) wR2(reflections) = 0.1144(3642)

S = 1.075 Npar = 254

7. Unsuccessful substrates

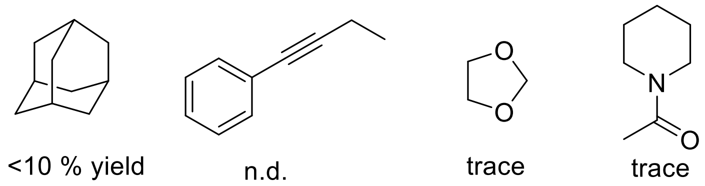

8. Analytical data for products

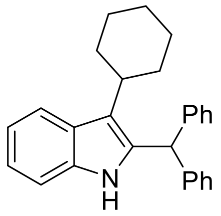

2-Benzhydryl-3-cyclohexyl-1*H*-indole (**3a**): According to the **General Procedure A**, **3a** (29.5 mg, 81%) was prepared as a white solid; m.p. 142-144 ºC; ^{1}H NMR (400 MHz, Chloroform-*d*) δ 7.76 (d, *J* = 7.4 Hz, 1H), 7.42 (s, 1H), 7.33 – 7.22 (m, 7H), 7.16 – 7.12 (m, 4H), 7.10 – 7.04 (m, 2H), 5.84 (s, 1H), 2.73 (tt, *J* = 12.3, 3.6 Hz, 1H), 1.97 – 1.64 (m, 8H), 1.30 – 1.27 (m, 2H). ^{13}C NMR (101 MHz, Chloroform-*d*) δ 142.5, 135.8, 134.2, 129.1, 128.7, 127.6, 126.9, 121.1, 120.5, 118.9, 118.3, 111.0, 48.5, 36.7, 33.0, 27.4, 26.4. HRMS (ESI): Calcd for $[C_{27}H_{27}N+H]^+$: 366.2222, found: 366.2220.

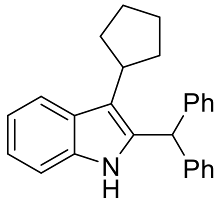

2-Benzhydryl-3-cyclopentyl-1*H*-indole (**3b**): According to the **General Procedure A**, **3b** (25.3 mg, 72%) was prepared as a white solid; m.p. 124-126 ºC; ^{1}H NMR (400 MHz, Chloroform-*d*) δ 7.66 (d, *J* = 7.8 Hz, 1H), 7.42 (s, 1H), 7.34 – 7.21 (m, 8H), 7.17 – 7.13 (m, 4H), 7.09 – 7.06 (m, 1H), 5.86 (s, 1H), 3.24 – 3.08 (m, 1H), 2.04 – 1.94 (m, 2H), 1.91 – 1.85 (m, 2H), 1.81 – 1.76 (m, 2H), 1.68 – 1.61 (m, 2H); ^{13}C NMR (101 MHz, Chloroform-*d*) δ 142.5, 135.9, 134.8, 129.1, 128.7, 127.2, 126.9, 121.1, 120.0, 118.9, 116.2, 111.1, 48.4, 37.2, 32.6, 26.5. HRMS (ESI): Calcd for $[C_{26}H_{25}N+H]^+$: 352.2065, found: 352.2056.

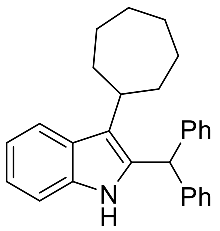

2-Benzhydryl-3-cycloheptyl-1*H*-indole (**3c**): According to the **General Procedure A**, **3c** (25.7 mg, 68%) was prepared as a white solid; m.p. 139-140 ºC; ^{1}H NMR (400 MHz, Chloroform-*d*) δ 7.68 (d, *J* = 7.5 Hz, 1H), 7.39 (s, 1H), 7.32 – 7.26 (m, 5H), 7.25 (s, 1H), 7.21 – 7.18 (m, 1H), 7.17 – 7.13 (m, 4H), 7.10 – 7.02 (m, 2H), 5.83 (s, 1H), 2.87 (tt, *J* = 11.2, 3.2 Hz, 1H), 2.06 – 1.97 (m, 2H), 1.77 – 1.70 (m, 4H), 1.65 – 1.56 (m, 4H), 1.42 – 1.33 (m, 2H); ^{13}C NMR (101 MHz, Chloroform-*d*) δ 142.5, 135.7, 133.3, 132.6, 130.2, 129.1, 128.7, 128.4, 127.4, 126.9, 121.1, 120.44, 120.37, 118.9, 110.9, 48.6, 38.4, 35.2, 28.2, 28.0. HRMS (ESI): Calcd for $[C_{28}H_{29}N+H]^+$: 380.2378, found: 380.2373.

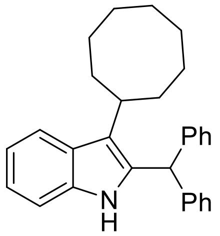

2-Benzhydryl-3-cyclooctyl-1*H*-indole (**3d**): According to the **General Procedure A**, **3d** (31.4 mg, 80%) was prepared as a white solid; m.p. 141-142 ºC; ^{1}H NMR (400 MHz, Chloroform-*d*) δ 7.64 (d, *J* = 7.7 Hz, 1H), 7.38 (s, 1H), 7.34 – 7.30 (m, 4H), 7.29 – 7.22 (m, 3H), 7.19 – 7.16 (m, 4H), 7.11 – 7.04 (m, 2H), 5.82 (s, 1H), 2.98 (tt, *J* = 10.3, 2.9 Hz, 1H), 2.09 – 2.01 (m, 2H), 1.75 – 1.62 (m, 6H), 1.56 – 1.47 (m, 6H); ^{13}C NMR (101 MHz, Chloroform-*d*) δ 142.4, 135.7, 133.4, 129.1, 128.8, 127.4, 126.9, 121.3, 121.1, 120.5, 118.9, 111.0, 48.8, 35.1, 34.4, 27.0, 26.7, 26.4. HRMS (ESI): Calcd for $[C_{29}H_{31}N+H]^+$: 394.2535, found: 394.2533.

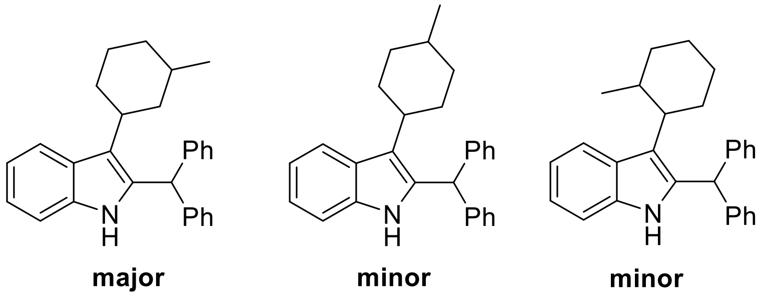

2-Benzhydryl-3-(3-methylcyclohexyl)-1*H*-indole (**3e, major**); 2-Benzhydryl-3-(4-methylcyclohexyl)-1*H*-indole (**3e, minor**); 2-Benzhydryl-3-(2-methylcyclohexyl)-1*H*-indole (**3e, minor**): According to the **General Procedure A**, **3e** (23.9 mg, 63%) was prepared as a white solid; m.p. 60-62 ºC; ^{1}H NMR (400 MHz, Chloroform-*d*) δ 7.78 – 7.70 (m, 1H), 7.45 and 7.42 (s, 1H), 7.33 – 7.27 (m, 4H), 7.27 – 7.24 (m, 2H), 7.23 – 7.16 (m, 2H), 7.16 – 7.12 (m, 3H), 7.11 – 7.04 (m, 2H), 5.83 and 5.82 and 5.80 (s, 1H), 3.13 – 2.88 and 2.81 – 2.65 and 2.40 – 2.33 (m, 1H), 2.14 – 1.58 (m, 6H), 1.49 – 1.27 (m, 3H), 1.12 and 0.92 and 0.62 (d, J = 6.5 Hz, 3H); ^{13}C NMR (101 MHz, Chloroform-*d*) δ 142.52, 142.46, 142.4, 142.2, 135.7, 135.6, 134.5, 134.2, 129.2, 129.1, 129.05, 129.03, 128.9, 128.72, 128.65, 128.6, 126.9, 126.8, 126.78, 126.72, 121.0, 120.9, 120.5, 120.4, 120.2, 118.9, 118.84, 118.81, 118.76, 118.0, 117.94, 117.91, 117.0, 110.9, 110.8, 48.8, 48.5, 48.4, 41.6, 38.0, 36.9, 36.4, 36.2, 36.2, 36.0, 35.1, 33.5, 33.3, 33.0, 32.7, 32.6, 32.4, 32.3, 31.4, 29.9, 29.7, 27.9, 27.2, 27.0, 26.9, 26.7, 26.6, 22.9, 22.9, 21.3, 21.3, 17.7, 17.4, 14.2. HRMS (ESI): Calcd for $[C_{28}H_{29}N+H]^{+}$: 380.2378, found: 380.2378.

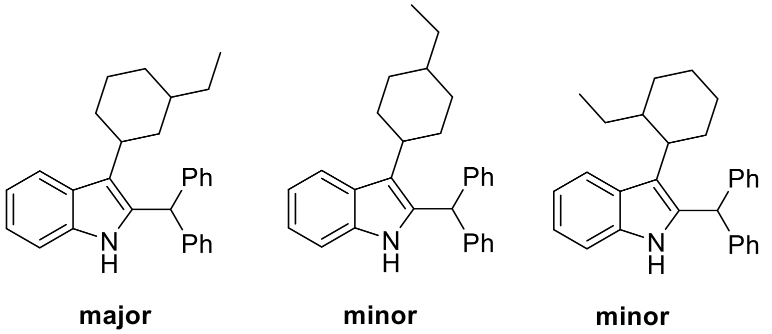

2-Benzhydryl-3-(3-ethylcyclohexyl)-1*H*-indole (**3f, major**); 2-Benzhydryl-3-(4-ethylcyclohexyl)-1*H*-indole (**3f, minor**); 2-Benzhydryl-3-(2-ethylcyclohexyl)-1*H*-

indole (**3f, minor**): According to the **General Procedure A**, **3f** (23.2 mg, 59%) was prepared as a yellowish oil; ^{1}H NMR (400 MHz, Chloroform-*d*) δ 7.81 – 7.68 (m, 1H), 7.43 (s, 1H), 7.33 – 7.26 (m, 5H), 7.23 – 7.03 (m, 8H), 5.84 and 5.82 and 5.81 (s, 1H), 2.96 – 2.88 and 2.81 – 2.60 and 2.48 – 2.45 (m, 1H), 2.12 – 1.82 (m, 2H), 1.73 – 1.61 (m, 2H), 1.55 – 1.44 (m, 2H), 1.31 – 1.17 (m, 3H), 0.99 – 0.86 (m, 2H), 0.83 – 0.49 (m, 3H); ^{13}C NMR (101 MHz, Chloroform-*d*) δ 142.5, 142.4, 142.3, 135.7, 135.6, 134.3, 134.2, 129.2, 129.13, 129.09, 129.06, 129.03, 129.01, 128.96, 128.92, 128.90, 128.71, 128.68, 128.6, 128.5, 126.89, 126.85, 126.8, 126.7, 121.0, 120.9, 120.5, 120.4, 120.2, 118.9, 118.85, 118.82, 118.1, 118.0, 110.9, 110.8, 48.7, 48.52, 48.48, 48.4, 42.6, 40.2, 39.3, 39.1, 36.8, 36.7, 36.4, 35.8, 35.5, 34.2, 33.6, 33.0, 32.8, 32.7, 31.9, 30.3, 30.2, 30.2, 30.1, 29.5, 27.3, 27.2, 27.0, 26.9, 26.8, 26.6, 23.8, 23.7, 21.65, 12.63, 12.5, 11.6, 11.4, 11.1. HRMS (ESI): Calcd for $[C_{29}H_{31}N+H]^+$: 394.2535, found: 394.2535.

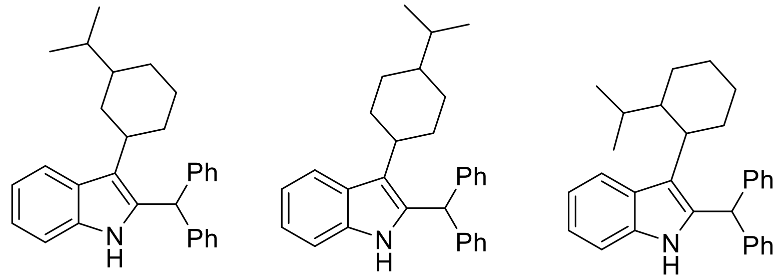

2-Benzhydryl-3-(3-isopropylcyclohexyl)-1*H*-indole (**3g, major**); 2-Benzhydryl-3-(4-isopropylcyclohexyl)-1*H*-indole (**3g, minor**); 2-Benzhydryl-3-(2-isopropylcyclohexyl)-1*H*-indole (**3g, minor**): According to the **General Procedure A**, **3g** (23.6 mg, 58%) was prepared as a yellowish solid; m.p. 52-54 ºC; ^{1}H NMR (400 MHz, Chloroform-*d*) δ 7.80 – 7.68 (m, 1H), 7.43 (s, 1H), 7.32 – 7.27 (m, 4H), 7.25 – 7.18 (m, 3H), 7.16 – 7.04 (m, 6H), 5.85 and 5.84 and 5.83 (s, 1H), 2.95 – 2.88 and 2.78 – 2.73 and 2.72 – 2.66 (m, 1H), 2.08 – 1.63 (m, 6H), 1.42 – 1.31 and 1.07 – 0.94 (m, 3H), 0.95 (d, *J* = 6.6 Hz, 1H), 0.87 (t, *J* = 6.7 Hz, 2H), 0.79 and 0.57 (d, *J* = 6.8 Hz, 4H); ^{13}C NMR (101 MHz, Chloroform-*d*) δ 142.5, 142.42, 142.36, 135.71, 135.66, 134.3, 134.2, 129.2, 129.1, 129.0, 128.9, 128.7, 128.7, 128.64, 128.58, 127.6, 126.9, 126.8, 126.72, 120.98, 120.95, 120.6, 120.4, 120.2, 118.9, 118.8, 118.2, 118.1, 110.9,

48.6, 48.5, 48.5, 44.8, 41.2, 39.9, 36.8, 36.7, 36.0, 34.6, 33.00, 32.96, 32.9, 32.6, 31.5, 30.4, 30.3, 30.2, 29.7, 29.0, 28.12, 28.07, 27.1, 25.8, 25.6, 21.7, 21.5, 21.1, 19.9, 19.7, 15.8. HRMS (ESI): Calcd for $[C_{27}H_{25}N+H]^+$: 364.2065, found: 364.2065.

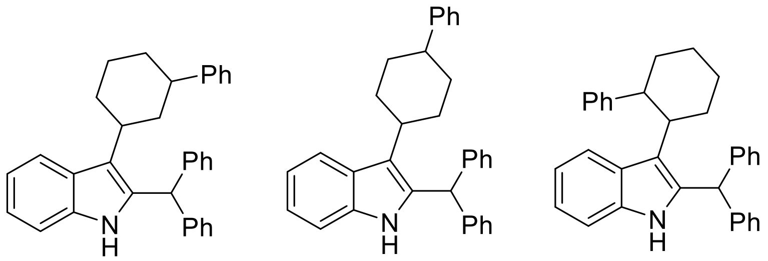

major **minor** **minor**

2-Benzhydryl-3-(3-phenylcyclohexyl)-1*H*-indole (**3i, major**); 2-Benzhydryl-3-(4-phenylcyclohexyl)-1*H*-indole (**3i, minor**); 2-Benzhydryl-3-(2-phenylcyclohexyl)-1*H*-indole (**3i, minor**): According to the **General Procedure A**, **3i** (26.5 mg, 60%) was prepared as a yellowish oil; ^{1}H NMR (400 MHz, Chloroform-*d*) δ 7.86 – 7.75 (m, 1H), 7.46 – 7.35 (m, 2H), 7.34 – 7.24 (m, 8H), 7.21 – 7.14 (m, 5H), 7.11 – 7.06 (m, 4H), 5.88 and 5.87 and 5.80 and 5.74 (s, 1H), 3.07 – 3.00 and 2.97 – 2.89 and 2.85 –2.80 (m, 1H), 2.72 – 2.36 (m, 1H), 2.35 – 2.20 (m, 1H), 2.19 – 1.69 (m, 6H), 1.67 –1.60 and 1.45 –1.44 (m, 1H); ^{13}C NMR (101 MHz, Chloroform-*d*) δ 147.8, 147.4, 144.7, 144.1, 142.54, 142.47, 142.4, 142.3, 135.8, 135.74, 135.68, 135.6, 134.5, 134.4, 134.3, 134.2, 130.1, 129.1, 129.05, 129.02, 128.9, 128.74, 128.71, 128.68, 128.6, 128.4, 128.33, 128.28, 127.9, 127.53, 127.50, 127.47, 126.9, 126.8, 125.9, 125.8, 125.3, 125.0, 121.1, 121.04, 121.01, 120.6, 120.4, 120.3, 120.0, 119.0, 118.94, 118.90, 118.1, 117.7, 117.6, 117.5, 111.1, 111.01, 110.97, 110.8, 48.6, 48.5, 48.3, 44.9, 44.3, 40.2, 36.9, 36.8, 36.2, 36.1, 35.8, 35.3, 34.9, 34.1, 32.9, 32.1, 30.8, 30.7, 30.1, 27.7, 27.3, 22.1. HRMS (ESI): Calcd for $[C_{33}H_{31}N+H]^+$: 442.2535, found: 442.2535.

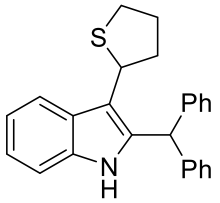

2-Benzhydryl-3-(tetrahydrothiophen-2-yl)-1*H*-indole (**3j**): According to the **General Procedure A,** 0.1 M HCl was employed in place of 0.25 M HCl, **3j** (23.3 mg, 63%) was prepared as a white solid; m.p. 62-63 ºC; ^{1}H NMR (400 MHz, Chloroform-*d*) δ 7.86 (d, *J* = 7.0 Hz, 1H), 7.48 (s, 1H), 7.34 – 7.25 (m, 6H), 7.22 – 7.20 (m, 1H), 7.16 – 7.08 (m, 6H), 5.97 (s, 1H), 4.79 (dd, *J* = 10.7, 6.1 Hz, 1H), 3.25 – 3.18 and 3.07 – 3.00 (m, 2H), 2.33 – 2.26 and 2.19 – 2.10 (m, 2H), 2.02 – 1.95 and 1.89 – 1.80 (m, 2H); ^{13}C NMR (101 MHz, Chloroform-*d*) δ 142.2, 141.7, 136.2, 135.6, 129.1, 128.9, 128.8, 128.7, 127.1, 126.93, 126.90, 121.5, 120.3, 119.3, 111.7, 111.1, 48.3, 45.0, 37.7, 33.6, 31.9. HRMS (ESI): Calcd for $[C_{25}H_{23}NS+H]^+$: 370.1629, found: 370.1623.

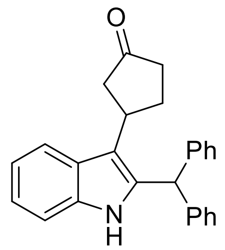

3-(2-Benzhydryl-1*H*-indol-3-yl)cyclopentan-1-one (**3k**): According to the **General Procedure A,** but with a reaction time of 4 h instead of 1 h, **3k** (16.8 mg, 46%) was prepared as a red solid; m.p. 196-198 ºC; ^{1}H NMR (400 MHz, Chloroform-*d*) δ 7.59 (d, *J* = 7.9 Hz, 1H), 7.53 (s, 1H), 7.36 – 7.33 (m, 2H), 7.32 – 7.29 (m, 3H), 7.27 (d, *J* = 1.7 Hz, 1H), 7.25 – 7.24 (m, 1H), 7.17 – 7.06 (m, 6H), 5.84 (s, 1H), 3.59 – 3.40 (m, 1H), 2.77 – 2.69 and 2.38 – 2.31 (m, 2H), 2.52 – 2.39 (m, 2H), 2.27 – 2.15 and 2.13 – 2.06 (m, 2H); ^{13}C NMR (101 MHz, Chloroform-*d*) δ 141.91, 141.85, 135.8, 135.6, 129.0, 128.91, 128.85, 127.2, 127.1, 126.7, 121.5, 119.5, 119.4, 112.8, 111.4, 48.7, 44.0, 39.5, 34.6, 29.4. HRMS (ESI): Calcd for $[C_{26}H_{23}NO+Na]^+$: 388.1677, found: 388.1680.

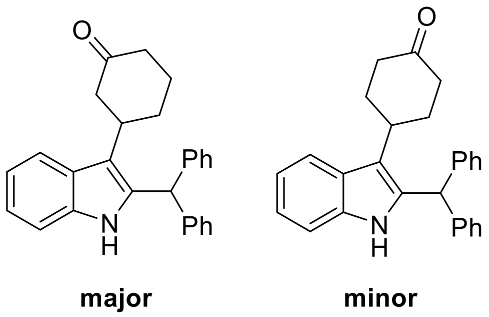

3-(2-Benzhydryl-1*H*-indol-3-yl)-cyclohexan-1-one (**3l, major**); 4-(2-Benzhydryl-1*H*-indol-3-yl)-cyclohexan-1-one (**3l, minor**): According to the **General Procedure A,** but with a reaction time of 4 h instead of 1 h, **3l** (23.2 mg, 61%) was prepared as a white solid; m.p. 149-151 ºC; ^{1}H NMR (400 MHz, Chloroform-*d*) δ 7.73 and 7.63 (d, *J* = 7.6 Hz, 1H), 7.56 (s, 1H), 7.34 – 7.23 (m, 7H), 7.18 – 7.08 (m, 6H), 5.88 and 5.79 (s, 1H), 3.17 (tt, *J* = 12.6, 3.8 Hz, 1H), 3.03 – 2.96 and 2.12 – 2.06 and 2.00 – 1.97 (m, 2H), 2.53 – 2.22 (m, 5H), , 1.81 – 1.77 and 1.64 – 1.61 (m, 1H); ^{13}C NMR (101 MHz, Chloroform-*d*) δ 211.9, 211.6, 142.0, 141.7, 135.6, 135.5, 135.0, 134.9, 129.0, 128.95, 128.91, 128.89, 128.8, 128.7, 127.2, 127.1, 127.0, 126.9, 121.41, 121.36, 119.9, 119.6, 119.4, 119.3, 115.0, 114.6, 111.3, 111.2, 48.8, 48.5, 47.8, 41.9, 41.4, 37.2, 35.3, 32.5, 31.2, 26.0. HRMS (ESI): Calcd for $[C_{27}H_{25}NO+Na]^+$: 402.1834, found: 402.1826.

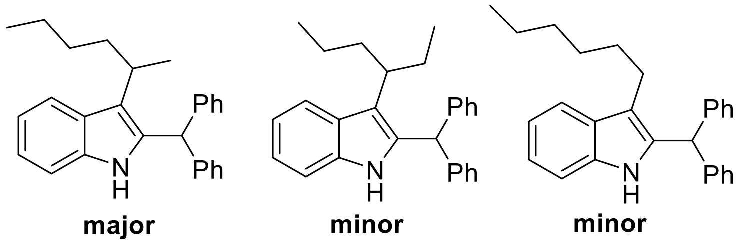

2-Benzhydryl-3-(hexan-2-yl)-1*H*-indole (**3l, major**); 2-Benzhydryl-3-(hexan-3-yl)-1*H*-indole (**3l, minor**); 2-Benzhydryl-3-hexyl-1*H*-indole (**3l, minor**): According to the **General Procedure A**, **3l** (21.7 mg, 59%) was prepared as a yellow oil; ^{1}H NMR (400 MHz, Chloroform-*d*) δ 7.70 and 7.67 (d, *J* = 7.6 Hz, 1H), 7.46 (s, 1H), 7.34 – 7.26 (m, 5H), 7.25 – 7.20 (m, 2H), 7.16 – 7.02 (m, 6H), 5.82 and 5.80 and 5.79 (s, 1H), 3.00 – 2.90 and 2.76 – 2.66(m, 1H), 1.87 –1.80 and 1.73 –1.66 (m, 2H), 1.31 (d, *J* = 7.1 Hz, 2H), 1.25 – 0.97 (m, 4H), 0.78 – 0.63 (m, 4H); ^{13}C NMR (101 MHz, Chloroform-*d*) δ 142.6, 142.5, 136.1, 136.0, 135.9, 134.7, 129.2, 129.14, 129.11, 129.07, 129.0, 128.8,

128.7, 127.5, 127.4, 127.0, 126.9, 121.1, 120.5, 120.4, 118.9, 118.8, 117.7, 115.8, 111.0, 110.9, 48.4, 48.3, 39.0, 37.9, 36.7, 31.5, 30.8, 28.6, 22.9, 21.53, 21.49, 14.3, 14.2, 13.1. HRMS (ESI): Calcd for $[C_{27}H_{29}N+H]^+$: 368.2378, found: 368.2382.

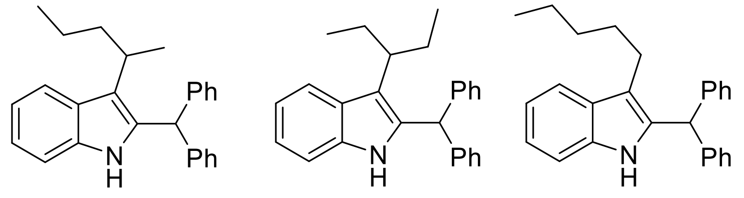

major **minor** **minor**

2-Benzhydryl-3-(pentan-2-yl)-1*H*-indole (**3m, major**); 2-Benzhydryl-3-(pentan-3-yl)-1*H*-indole (**3m, minor**); 2-Benzhydryl-3-pentyl-1*H*-indole (**3m, minor**): According to the **General Procedure A**, **3m** (22.6 mg, 64%) was prepared as a yellow oil; ^{1}H NMR (400 MHz, Chloroform-*d*) δ 7.69 (d, *J* = 7.8 Hz, 1H), 7.42 (s, 1H), 7.32 – 7.20 (m, 7H), 7.16 – 6.99 (m, 6H), 5.81 and 5.80 and 5.79(s, 1H), 3.03 – 2.91 and 2.69 – 2.58 (m, 1H), 1.89 – 1.60 and 1.20 – 1.11 (m, 4H), 1.30 – 1.28 and 0.78 – 0.74 and 0.68 – 0.64 (m, 6H); ^{13}C NMR (101 MHz, Chloroform-*d*) δ 142.5, 142.44, 142.41, 135.8, 134.6, 130.1, 129.10, 129.06, 129.0, 128.9, 128.68, 128.66, 128.6, 127.4, 126.9, 126.8, 121.0, 120.4, 120.3, 118.84, 118.76, 117.6, 115.4, 110.9, 48.3, 48.2, 41.2, 39.1, 31.2, 28.3, 21.5, 21.3, 14.2, 13.0. HRMS (ESI): Calcd for $[C_{26}H_{27}N+H]^+$: 354.2222, found: 354.2222.

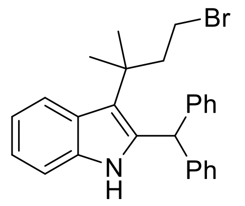

2-Benzhydryl-3-(4-bromo-2-methylbutan-2-yl)-1*H*-indole (**3n**): According to the **General Procedure A,** 0.1 M HCl was employed in place of 0.25 M HCl, with a reaction time of 4 h instead of 1 h, **3n** (19.4 mg, 45%) was prepared as a yellow solid; m.p. 49-50 ºC; ^{1}H NMR (400 MHz, Chloroform-*d*) δ 7.82 (d, *J* = 8.0 Hz, 1H), 7.44 (s, 1H), 7.35 – 7.25 (m, 6H), 7.20 – 7.17 (m, 1H), 7.14 – 7.09 (m, 1H), 7.09 – 7.04 (m,

5H), 6.17 (s, 1H), 3.08 – 2.97 (m, 2H), 2.56 – 2.42 (m, 2H), 1.54 (s, 6H); ^{13}C NMR (101 MHz, Chloroform-*d*) δ 142.8, 135.7, 134.8, 129.2, 128.9, 127.7, 127.1, 121.8, 121.5, 119.2, 116.8, 111.0, 49.5, 47.2, 38.3, 30.5, 30.2. HRMS (ESI): Calcd for $[C_{26}H_{26}NBr+H]^+$: 432.1327, found: 432.1338.

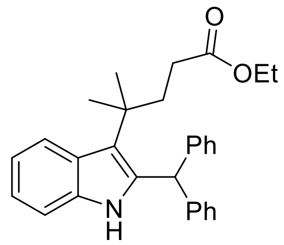

Ethyl 4-(2-benzhydryl-1*H*-indol-3-yl)-4-methylpentanoate (**3o**): According to the **General Procedure A,** but with a reaction time of 4 h instead of 1 h, **3o** (18.3 mg, 43%) was prepared as a colorless oil; ^{1}H NMR (400 MHz, Chloroform-*d*) δ 7.86 (d, *J* = 8.0 Hz, 1H), 7.42 (s, 1H), 7.33 – 7.24 (m, 7H), 7.18 – 7.15 (m, 1H), 7.09 – 7.05 (m, 5H), 6.20 (s, 1H), 3.98 (q, *J* = 7.1 Hz, 2H), 2.24 – 2.16 (m, 2H), 2.07 – 1.99 (m, 2H), 1.50 (s, 6H), 1.16 (t, *J* = 7.1 Hz, 3H); ^{13}C NMR (101 MHz, Chloroform-*d*) δ 174.3, 142.9, 135.7, 134.6, 129.1, 128.8, 127.8, 126.9, 122.0, 121.2, 118.9, 117.7, 110.8, 60.1, 49.5, 38.2, 36.8, 30.8, 30.0, 14.2. HRMS (ESI): Calcd for $[C_{29}H_{31}NO_2+H]^+$: 426.2433, found: 426.2433.

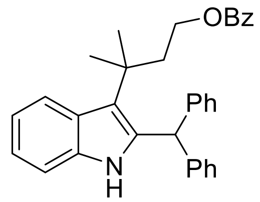

3-(2-Benzhydryl-1*H*-indol-3-yl)-3-methylbutyl benzoate (**3p**): According to the **General Procedure A,** but with a reaction time of 4 h instead of 1 h, **3p** (18.9 mg, 40%) was prepared as a white solid; m.p. 94-96 ºC; ^{1}H NMR (400 MHz, Chloroform-*d*) δ 7.88 (d, *J* = 7.9 Hz, 1H), 7.74 (d, *J* = 7.1 Hz, 2H), 7.49 –7.46 (m, 1H), 7.39 (s, 1H), 7.35 –7.31 (m, 2H), 7.28 –7.25 (m, 4H), 7.22 (d, *J* = 7.4 Hz, 1H), 7.20 (d, *J* = 7.3 Hz, 1H), 7.16 (d, *J* = 7.5 Hz, 1H), 7.10 – 7.05 (m, 6H), 6.24 (s, 1H), 4.11 (t, *J* = 6.9 Hz, 2H), 2.39 (t, *J* = 6.9 Hz, 2H), 1.61 (s, 6H); ^{13}C NMR (101 MHz, Chloroform-*d*) δ 166.5,

142.9, 135.8, 134.4, 132.6, 130.5, 129.5, 129.1, 128.8, 128.2, 127.9, 126.9, 122.0, 121.2, 119.0, 117.8, 111.0, 63.2, 49.5, 41.6, 36.3, 30.5. HRMS (ESI): Calcd for $[C_{33}H_{31}NO_2+H]^+$: 474.2433, found: 474.2433.

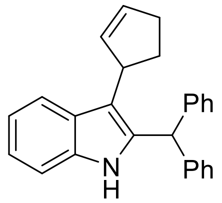

2-Benzhydryl-3-(cyclopent-2-en-1-yl)-1*H*-indole (**3q**): According to the **General Procedure A**, **3q** (29.7 mg, 85%) was prepared as a white solid; m.p. 80-81 ºC; ^{1}H NMR (400 MHz, Chloroform-*d*) δ 7.63 (d, J = 8.3 Hz, 1H), 7.48 (s, 1H), 7.35 – 7.28 (m, 5H), 7.27 – 7.21 (m, 2H), 7.18 – 7.14 (m, 4H), 7.14 – 7.09 (m, 1H), 7.07 – 7.02 (m, 1H), 5.92 (s, 1H), 5.92 – 5.89 (m, 1H), 5.82 – 5.76 (m, 1H), 4.22 – 4.10 (m, 1H), 2.63 – 2.50 and 2.47 – 2.37 (m, 2H), 2.22 – 2.09 and 1.94 – 1.84 (m, 2H); ^{13}C NMR (101 MHz, Chloroform-*d*) δ 142.5, 142.3, 135.6, 134.9, 134.7, 130.9, 129.1, 128.7, 128.7, 127.9, 126.9, 126.8, 121.2, 119.8, 119.0, 115.8, 110.8, 48.3, 42.3, 32.9, 31.3. HRMS (ESI): Calcd for $[C_{26}H_{23}N+Na]^+$: 372.1728, found: 372.1713.

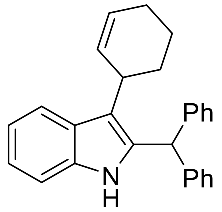

2-Benzhydryl-3-(cyclohex-2-en-1-yl)-1*H*-indole (**3r**): According to the **General Procedure A**, **3r** (31.2 mg, 86%) was prepared as a white solid; m.p. 55-56 ºC; ^{1}H NMR (400 MHz, Chloroform-*d*) δ 7.70 (d, *J* = 7.9 Hz, 1H), 7.51 (s, 1H), 7.36 – 7.30 (m, 4H), 7.30 – 7.26 (m, 2H), 7.25 – 7.21 (m, 1H), 7.19 – 7.15 (m, 4H), 7.15 – 7.04 (m, 2H), 5.94 (s, 1H), 5.92 – 5.85 (m, 1H), 5.82 – 5.75 (m, 1H), 3.75 – 3.65 (m, 1H), 2.27 – 2.11 (m, 2H), 1.87 – 1.77 (m, 3H), 1.69 – 1.58 (m, 1H); ^{13}C NMR (101 MHz, Chloroform-*d*) δ 142.6, 142.5, 135.6, 135.1, 131.7, 129.1, 129.1, 128.8, 128.0, 127.5,

126.9, 121.3, 120.0, 119.1, 116.5, 110.9, 48.2, 33.6, 30.6, 25.7, 22.9. HRMS (ESI): Calcd for $[C_{27}H_{25}N+H]^+$: 364.2065, found: 364.2084.

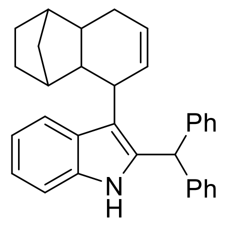

2-Benzhydryl-3-(1,2,3,4,4a,5,8,8a-octahydro-1,4-methanonaphthalen-5-yl)-1*H*-indole (**3s**): According to the **General Procedure A**, **3s** (31.8 mg, 74%) was prepared as a white solid; m.p. 64-66 ºC; ^{1}H NMR (400 MHz, Chloroform-*d*) δ 7.77 and 7.60 (d, *J* = 8.1 Hz, 1H), 7.48 (s, 1H), 7.36 – 7.27 (m, 4H), 7.27 – 7.23 (m, 3H), 7.20 – 7.14 (m, 3H), 7.12 – 6.99 (m, 3H), 5.97 – 5.93 (m, 1H), 5.89 – 5.76 (m, 2H), 3.54 – 3.50 and 3.14 – 3.12 and 3.01 – 2.97 (m, 1H), 2.36 – 2.14 (m, 1H), 2.12 – 1.83 (m, 3H), 1.79 – 1.62 (m, 3H), 1.59 – 1.54 (m, 1H), 1.41 – 1.38 (m, 1H), 1.23 – 1.09 and 1.02 – 0.77 (m, 3H); ^{13}C NMR (101 MHz, Chloroform-*d*) δ 142.54, 142.50, 142.41, 142.37, 142.2, 142.1, 135.5, 135.3, 135.0, 134.8, 132.9, 132.1, 129.2, 129.1, 129.05, 128.99, 128.9, 128.8, 128.74, 128.69, 128.65, 128.6, 128.3, 127.9, 126.94, 126.89, 126.8, 126.7, 121.3, 121.2, 120.81, 120.79, 119.9, 119.11, 119.07, 119.0, 117.1, 116.6, 111.0, 110.84, 110.78, 50.7, 49.6, 48.9, 48.4, 48.3, 44.4, 44.2, 43.7, 43.5, 43.4, 43.2, 42.7, 41.4, 41.2, 40.0, 37.9, 35.2, 35.1, 34.1, 33.5, 32.9, 31.8, 31.6, 31.5, 30.4, 30.2, 30.1, 29.8, 28.8, 28.1, 27.9, 27.7. HRMS (ESI): Calcd for $[C_{31}H_{31}N+H]^+$: 430.2535, found: 430.2535.

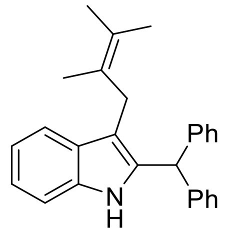

2-Benzhydryl-3-(2,3-dimethylbut-2-en-1-yl)-1*H*-indole (**3t**): According to the **General Procedure A,** but with a reaction time of 4 h instead of 1 h, **3t** (21.9 mg, 60%) was prepared as a white solid; m.p. 124-125 ºC; ^{1}H NMR (400 MHz, Chloroform-*d*) δ 7.89

and 7.56 (d, J = 7.5 Hz, 1H), 7.52 and 7.46 (s, 1H), 7.34 – 7.27 (m, 6H), 7.25 – 7.21 (m, 1H), 7.15 – 7.08 (m, 6H), 6.27 and 5.83 (s, 1H), 5.08 and 4.92 and 3.51 (s, 2H), 1.84 and 1.62 and 1.45 (s, 9H); ^{13}C NMR (101 MHz, Chloroform-*d*) δ 154.2, 143.1, 142.4, 135.9, 135.5, 134.7, 129.5, 129.3, 129.1, 128.8, 128.7, 127.7, 126.9, 126.6, 124.3, 121.7, 121.3, 121.2, 119.3, 119.1, 118.9, 117.7, 111.5, 110.73, 110.66, 108.5, 48.8, 48.2, 42.4, 29.5, 28.8, 20.8, 20.8, 17.7. HRMS (ESI): Calcd for $[C_{27}H_{27}N+H]^+$: 366.2222, found: 366.2212.

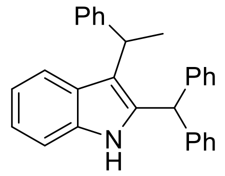

2-Benzhydryl-3-(1-phenylethyl)-1*H*-indole (**3u**): According to the **General Procedure A**, **3u** (33.7 mg, 87%) was prepared as a white solid; m.p. 40-42 ºC; ^{1}H NMR (400 MHz, Chloroform-*d*) δ 7.52 (s, 1H), 7.42 (d, J = 7.9 Hz, 1H), 7.33 – 7.22 (m, 12H), 7.19 – 7.15 (m, 1H), 7.13 – 7.10 (m, 2H), 7.10 – 7.07 (m, 2H), 7.01 – 6.95 (m, 1H), 5.74 (s, 1H), 4.47 (q, J = 7.2 Hz, 1H), 1.64 (d, J = 7.3 Hz, 3H); ^{13}C NMR (101 MHz, Chloroform-*d*) δ 145.6, 142.3, 142.3, 135.7, 135.3, 129.2, 129.0, 128.82, 128.80, 128.2, 127.7, 127.6, 127.02, 126.99, 125.8, 121.4, 120.1, 119.3, 117.3, 110.9, 48.3, 35.2, 20.2. HRMS (ESI): Calcd for $[C_{29}H_{25}N+H]^+$: 388.2065, found: 388.2069.

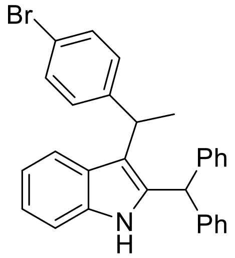

2-Benzhydryl-3-(1-(4-bromophenyl)-ethyl)-1*H*-indole (**3v**): According to the **General Procedure A**, **3v** (37.7 mg, 81%) was prepared as a white solid; m.p. 143-144 ºC; ^{1}H NMR (400 MHz, Chloroform-*d*) δ 7.54 (s, 1H), 7.38 (d, J = 8.0 Hz, 1H), 7.31 (t, J = 7.5 Hz, 6H), 7.28 – 7.26 (m, 2H), 7.24 (d, J = 3.5 Hz, 1H), 7.15 – 7.09 (m, 5H), 7.09 –

7.06 (m, 2H), 7.00 (t, *J* = 7.5 Hz, 1H), 5.74 (s, 1H), 4.39 (q, *J* = 7.3 Hz, 1H), 1.65 (d, *J* = 7.2 Hz, 3H); ^{13}C NMR (101 MHz, Chloroform-*d*) δ 144.6, 142.0, 141.9, 135.6, 135.4, 131.1, 129.3, 129.0, 128.9, 128.8, 128.8, 127.4, 127.03, 126.98, 121.4, 119.9, 119.5, 119.4, 116.5, 110.9, 48.3, 34.8, 20.1. HRMS (ESI): Calcd for $[C_{29}H_{24}NBr+H]^+$: 466.1770, found: 466.1779.

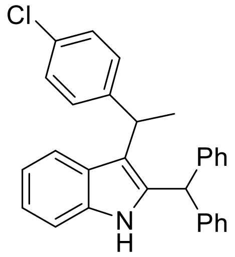

2-Benzhydryl-3-(1-(4-chlorophenyl)ethyl)-1*H*-indole (**3w**): According to the **General Procedure A**, **3w** (35.8 mg, 85%) was prepared as a white solid; m.p. 136-137 ºC; ^{1}H NMR (400 MHz, Chloroform-*d*) δ 7.52 (s, 1H), 7.36 (d, *J* = 7.9 Hz, 1H), 7.28 (p, *J* = 6.7 Hz, 6H), 7.21 (d, *J* = 8.1 Hz, 1H), 7.15 (s, 4H), 7.07 (dd, *J* = 14.2, 7.0 Hz, 5H), 6.97 (t, *J* = 7.5 Hz, 1H), 5.72 (s, 1H), 4.39 (q, *J* = 7.3 Hz, 1H), 1.63 (d, *J* = 7.3 Hz, 3H); ^{13}C NMR (101 MHz, Chloroform-*d*) δ 144.1, 142.0, 141.9, 135.6, 135.4, 131.4, 129.0, 128.9, 128.9, 128.8, 128.7, 128.3, 127.4, 127.01, 126.98, 121.4, 119.9, 119.4, 116.6, 110.9, 48.3, 34.7, 20.2. HRMS (ESI): Calcd for $[C_{29}H_{24}NCl+H]^+$: 422.1676, found: 422.1675.

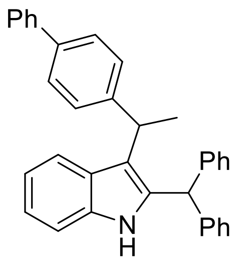

3-(1-([1,1'-Biphenyl]-4-yl)ethyl)-2-benzhydryl-1*H*-indole (**3x**): According to the **General Procedure A**, **3x** (31.9 mg, 69%) was prepared as a white solid; m.p. 68-70 ºC; ^{1}H NMR (400 MHz, Chloroform-*d*) δ 7.60 – 7.57 (m, 2H), 7.55 (s, 1H), 7.50 – 7.40 (m, 5H), 7.35 (dd, *J* = 6.8, 4.9 Hz, 3H), 7.31 (dd, *J* = 5.1, 2.1 Hz, 3H), 7.29 – 7.23 (m,

4H), 7.17 – 7.11 (m, 3H), 7.08 (d, J = 6.9 Hz, 2H), 7.01 (t, J = 7.2 Hz, 1H), 5.79 (s, 1H), 4.52 (q, J = 7.1 Hz, 1H), 1.71 (d, J = 7.3 Hz, 3H); ^{13}C NMR (101 MHz, Chloroform-d) δ 144.8, 142.3, 142.2, 141.2, 138.6, 135.7, 135.4, 129.2, 129.0, 128.82, 128.79, 128.0, 127.7, 127.13, 127.09, 127.03, 126.98, 126.9, 121.4, 120.1, 119.4, 117.2, 111.0, 48.3, 35.0, 20.3. HRMS (ESI): Calcd for $[C_{35}H_{29}N+H]^+$: 464.2378, found: 464.2388.

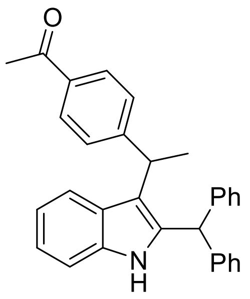

1-(4-(1-(2-Benzhydryl-1*H*-indol-3-yl)ethyl)phenyl)ethan-1-one (**3y**): According to the **General Procedure A**, but with a reaction time of 4 h instead of 1 h, **3y** (24.1 mg, 56%) was prepared as a red solid; m.p. 141-143 ºC; ^{1}H NMR (400 MHz, Chloroform-d) δ 7.80 (d, J = 8.4 Hz, 2H), 7.58 (s, 1H), 7.37 – 7.24 (m, 10H), 7.12 – 7.05 (m, 5H), 6.98 (t, J = 7.2 Hz, 1H), 5.74 (s, 1H), 4.48 (q, J = 7.3 Hz, 1H), 2.55 (s, 3H), 1.69 (d, J = 7.2 Hz, 3H); ^{13}C NMR (101 MHz, Chloroform-d) δ 198.1, 151.5, 142.0, 141.9, 135.7, 135.6, 134.8, 129.1, 129.0, 128.9, 128.8, 128.4, 127.8, 127.4, 127.13, 127.07, 121.5, 119.9, 119.5, 116.3, 111.1, 48.4, 35.5, 26.7, 20.0. HRMS (ESI): Calcd for $[C_{31}H_{27}NO+H]^+$: 430.2187, found: 430.2171.

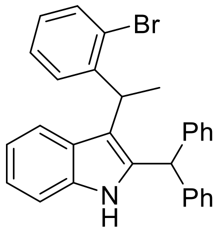

2-Benzhydryl-3-(1-(2-bromophenyl)ethyl)-1*H*-indole (**3z**): According to the **General Procedure A**, 0.1 M HCl was employed in place of 0.25 M HCl, with a reaction time of 4 h instead of 1 h, **3z** (31.1 mg, 67%) was prepared as a yellowish oil; ^{1}H NMR (400 MHz, Chloroform-d) δ 7.66 (d, J = 7.9 Hz, 1H), 7.53 (s, 1H), 7.47 – 7.44 (m, 2H), 7.32

– 7.18 (m, 9H), 7.15 – 7.06 (m, 4H), 7.00 – 6.97 (m, 2H), 5.72 (s, 1H), 4.71 (q, *J* = 7.3 Hz, 1H), 1.60 (d, *J* = 7.2 Hz, 3H); ^{13}C NMR (101 MHz, Chloroform-*d*) δ 145.1, 142.0, 141.8, 136.0, 135.4, 132.8, 129.1, 129.0, 128.6, 128.5, 128.3, 127.5, 127.3, 126.8, 126.7, 124.6, 121.2, 119.9, 119.4, 115.0, 110.9, 48.3, 36.6, 20.5. HRMS (ESI): Calcd for $[C_{29}H_{24}NBr+H]^+$: 466.1170, found: 466.1170.

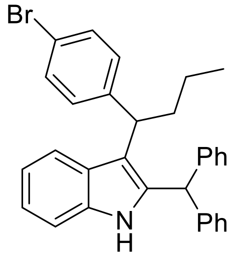

2-Benzhydryl-3-(1-(4-bromophenyl)-butyl)-1*H*-indole (**3aa**): According to the **General Procedure A**, **3aa** (33.5 mg, 68%) was prepared as a white solid; m.p. 42-43 ºC; ^{1}H NMR (400 MHz, Chloroform-*d*) δ 7.57 – 7.51 (m, 2H), 7.30 – 7.25 (m, 6H), 7.24 – 7.21 (m, 4H), 7.11 – 7.07 (m, 2H), 7.05 – 7.02 (m, 5H), 5.72 (s, 1H), 4.15 (dd, *J* = 9.6, 6.1 Hz, 1H), 2.16 – 2.05 (m, 2H), 1.26 – 1.17 (m, 2H), 0.78 (t, *J* = 7.3 Hz, 3H); ^{13}C NMR (101 MHz, Chloroform-*d*) δ 144.5, 142.0, 141.9, 136.2, 135.6, 131.3, 131.1, 130.2, 129.7, 129.0, 128.84, 128.78, 127.8, 127.1, 127.0, 121.4, 120.0, 119.5, 115.0, 111.0, 48.4, 41.1, 36.4, 21.6, 14.2. HRMS (ESI): Calcd for $[C_{31}H_{28}NBr+H]^+$: 494.1483, found: 494.1473.

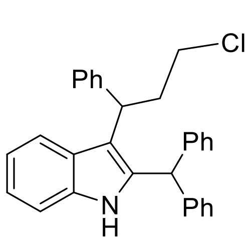

2-Benzhydryl-3-(3-chloro-1-phenylpropyl)-1*H*-indole (**3ab**): According to the **General Procedure A**, **3ab** (28.7 mg, 66%) was prepared as a colorless oil; ^{1}H NMR (400 MHz, Chloroform-*d*) δ 7.61 (s, 1H), 7.54 (d, *J* = 8.4 Hz, 1H), 7.36 – 7.29 (m, 3H), 7.29 – 7.25 (m, 2H), 7.25 – 7.21 (m, 3H), 7.20 – 7.17 (m, 1H), 7.16 – 7.13 (m, 5H),

7.11 – 7.07 (m, 3H), 7.05 – 7.00 (m, 1H), 5.91 (s, 1H), 4.56 (dd, *J* = 10.5, 5.1 Hz, 1H), 3.57 – 3.45 and 3.38 – 3.29 (m, 2H), 2.83 – 2.67 and 2.63 – 2.54 (m, 2H); ^{13}C NMR (101 MHz, Chloroform-*d*) δ 143.8, 142.1, 142.0, 137.2, 135.7, 129.09, 129.06, 129.0, 128.8, 128.3, 127.8, 127.6, 127.2, 127.0, 126.0, 121.5, 120.1, 119.6, 113.3, 111.1, 48.2, 44.0, 38.3, 36.4. HRMS (ESI): Calcd for $[C_{30}H_{26}NCl+H]^+$: 436.1832, found: 436.1840.

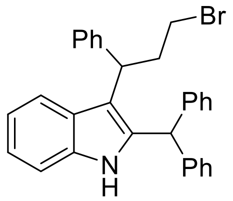

2-Benzhydryl-3-(3-bromo-1-phenylpropyl)-1*H*-indole (**3ac**): According to the **General Procedure A**, **3ac** (32.6 mg, 68%) was prepared as a colorless oil; ^{1}H NMR (400 MHz, Chloroform-*d*) δ 7.59 (s, 1H), 7.55 (d, *J* = 8.0 Hz, 1H), 7.35 – 7.30 (m, 2H), 7.29 – 7.20 (m, 5H), 7.17 – 7.05 (m, 10H), 7.04 – 7.00 (m, 1H), 5.92 (s, 1H), 4.52 (dd, *J* = 10.3, 5.2 Hz, 1H), 3.40 – 3.13 (m, 2H), 2.84 – 2.61 (m, 2H); ^{13}C NMR (101 MHz, Chloroform-*d*) δ 143.6, 142.0, 141.9, 137.1, 135.6, 129.04, 128.98, 128.9, 128.7, 128.2, 127.7, 127.5, 127.1, 126.9, 126.0, 121.5, 120.0, 119.6, 113.2, 111.0, 48.2, 39.6, 36.8, 33.0. HRMS (ESI): Calcd for $[C_{30}H_{26}NBr+H]^+$: 480.1327, found: 480.1327.

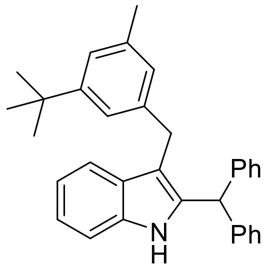

2-Benzhydryl-3-(3-(tert-butyl)-5-methylbenzyl)-1*H*-indole (**3ad**): According to the **General Procedure A**, but with a reaction time of 4 h instead of 1 h, **3ad** (31.1 mg, 70%) was prepared as a colorless oil; ^{1}H NMR (400 MHz, Chloroform-*d*) δ 7.56 (s, 1H), 7.48 (d, *J* = 7.8 Hz, 1H), 7.31 – 7.22 (m, 7H), 7.13 – 7.06 (m, 5H), 7.06 – 7.00 (m, 2H), 6.96 (s, 1H), 6.69 (s, 1H), 5.73 (s, 1H), 4.01 (s, 2H), 2.18 (s, 3H), 1.23 (s, 9H); ^{13}C NMR (101 MHz, Chloroform-*d*) δ 150.9, 142.1, 140.7, 137.3, 136.0, 135.4, 129.1, 129.0,

128.7, 126.8, 126.5, 123.6, 122.7, 121.4, 119.4, 119.1, 111.9, 110.6, 48.2, 34.5, 31.4, 30.4, 21.6. HRMS (ESI): Calcd for $[C_{28}H_{23}N+H]^+$: 374.1909, found: 374.1909.

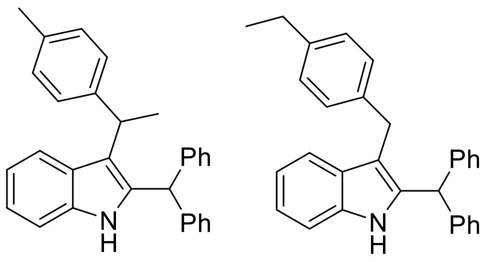

major **minor**

2-Benzhydryl-3-(1-(p-tolyl)ethyl)-1*H*-indole (**3ae, major**); 2-Benzhydryl-3-(4-ethylbenzyl)-1*H*-indole (**3ae, minor**): According to the **General Procedure A**, **3ae** (33.7 mg, 84%) was prepared as a colorless oil; ^{1}H NMR (400 MHz, Chloroform-*d*) δ 7.55 and 7.48 (s, 1H), 7.41 (d, *J* = 8.3 Hz, 1H), 7.30 – 7.22 (m, 6H), 7.20 – 7.13 (m, 2H), 7.12 – 6.94 (m, 9H), 5.75 and 5.73 (s, 1H), 4.44 – 4.38 and 4.00 and 2.60 – 2.55 (m, 2H), 2.29 (s, 2H), 1.62 –1.60 and 1.21 – 1.17 (m, 3H); ^{13}C NMR (101 MHz, Chloroform-*d*) δ 142.5, 142.3, 142.2, 142.1, 141.6, 138.4, 136.2, 135.7, 135.5, 135.2, 135.1, 129.5, 129.1, 129.02, 128.97, 128.8, 128.72, 128.67, 128.4, 127.9, 127.8, 127.7, 127.4, 126.9, 126.8, 121.5, 121.2, 120.1, 119.5, 119.2, 119.1, 117.4, 111.8, 110.8, 110.7, 48.3, 48.2, 34.7, 29.7, 28.5, 21.0, 20.2, 15.8. HRMS (ESI): Calcd for $[C_{30}H_{27}N+H]^+$: 402.2222, found: 402.2222.

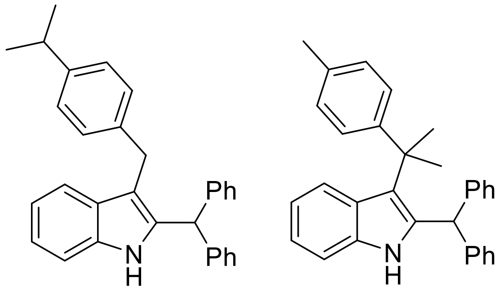

major **minor**

2-Benzhydryl-3-(4-isopropylbenzyl)-1*H*-indole (**3af, major**); 2-Benzhydryl-3-(2-(p-tolyl)propan-2-yl)-1*H*-indole (**3af, minor**): According to the **General Procedure A**, **3af** (29.8 mg, 72%) was prepared as a colorless oil; ^{1}H NMR (400 MHz, Chloroform-

d) δ 7.55 and 7.47 – 7.44 and 7.39 – 7.37 (m, 2H), 7.30 – 7.28 and 7.25 – 7.16 (m, 8H), 7.12 – 7.07 (m, 3H), 7.06 – 7.02 (m, 4H), 6.97 – 6.92 (m, 2H), 5.77 and 5.75 (s, 1H), 4.01 and 2.86 – 2.80 and 2.30 (m, 3H), 1.75 and 1.21 – 1.20 (m, 6H); ^{13}C NMR (101 MHz, Chloroform-*d*) δ 149.0, 146.2, 142.9, 142.1, 138.5, 136.2, 135.50, 135.45, 135.0, 134.9, 131.0, 129.5, 129.1, 129.04, 129.01, 128.8, 128.7, 128.6, 128.4, 128.2, 127.9, 126.9, 126.74, 126.71, 126.3, 125.8, 122.0, 121.5, 121.0, 120.6, 119.5, 119.1, 118.9, 111.9, 110.7, 110.6, 48.8, 48.3, 40.9, 33.7, 31.7, 29.7, 24.1, 24.0, 21.0. HRMS (ESI): Calcd for $[C_{31}H_{29}N+Na]^+$: 438.2198, found: 438.2204.

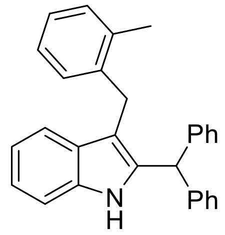

2-Benzhydryl-3-(2-methylbenzyl)-1*H*-indole (**3ag**): According to the **General Procedure A**, but with a reaction time of 4 h instead of 1 h, **3ag** (21.7 mg, 56%) was prepared as a white solid; m.p. 112-114 ºC; ^{1}H NMR (400 MHz, Chloroform-*d*) δ 7.60 (s, 1H), 7.33 (d, *J* = 7.9 Hz, 1H), 7.31 – 7.27 (m, 2H), 7.27 – 7.23 (m, 5H), 7.15 – 7.11 (m, 2H), 7.10 – 7.05 (m, 5H), 7.04 – 6.99 (m, 2H), 6.98 – 6.94 m, 1H), 5.61 (s, 1H), 3.96 (s, 2H), 2.27 (s, 3H); ^{13}C NMR (101 MHz, Chloroform-*d*) δ 142.0, 138.9, 136.5, 136.4, 135.5, 129.9, 129.2, 129.0, 128.8, 128.6, 126.9, 126.0, 125.9, 121.5, 119.5, 119.1, 110.8, 110.5, 48.3, 27.7, 19.9. HRMS (ESI): Calcd for $[C_{29}H_{25}N+H]^+$: 388.2065, found: 388.2069.

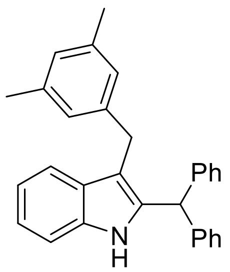

2-Benzhydryl-3-(3,5-dimethylbenzyl)-1*H*-indole (**3ah**): According to the **General**

Procedure A, but with a reaction time of 4 h instead of 1 h, **3ah** (30.0 mg, 75%) was prepared as a colorless oil; ^{1}H NMR (400 MHz, Chloroform-*d*) δ 7.59 (s, 1H), 7.48 (d, *J* = 8.0 Hz, 1H), 7.33 – 7.24 (m, 7H), 7.14 – 7.10 (m, 5H), 7.07 – 7.03 (m, 1H), 6.76 (s, 1H), 6.72 (s, 2H), 5.75 (s, 1H), 3.99 (s, 2H), 2.18 (s, 6H); ^{13}C NMR (101 MHz, Chloroform-*d*) δ 142.2, 141.1, 137.7, 136.2, 135.5, 129.1, 128.8, 127.5, 127.0, 126.4, 121.5, 119.5, 119.1, 111.8, 110.7, 48.3, 30.1, 21.4. HRMS (ESI): Calcd for $[C_{30}H_{27}N+H]^+$: 402.2222, found: 402.2229.

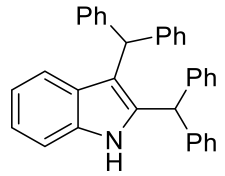

2,3-Bibenzhydryl-1*H*-indole (**3ai**): According to the **General Procedure A**, 0.1 M HCl was employed in place of 0.25 M HCl, with a reaction time of 4 h instead of 1 h, **3ai** (24.2 mg, 54%) was prepared as a colorless oil; ^{1}H NMR (400 MHz, Chloroform-*d*) δ 7.59 (s, 1H), 7.28 – 7.24 (m, 5H), 7.23 – 7.18 (m, 8H), 7.12 – 7.08 m, 4H), 7.08 –7.02 (m, 5H), 6.95 – 6.82 (m, 2H), 5.70 (s, 1H), 5.58 (s, 1H); ^{13}C NMR (101 MHz, Chloroform-*d*) δ 143.6, 141.9, 136.8, 135.5, 131.2, 130.9, 129.3, 129.1, 128.8, 128.4, 128.2, 127.9, 127.7, 127.0, 126.2, 121.3, 120.6, 119.5, 115.0, 110.8, 48.3, 47.8. HRMS (ESI): Calcd for $[C_{34}H_{27}N+H]^+$: 450.2222, found: 450.2241.

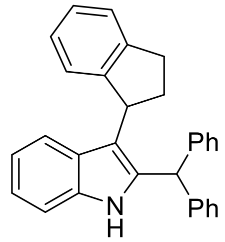

2-Benzhydryl-3-(2,3-dihydro-1*H*-inden-1-yl)-1*H*-indole (**3aj**): According to the **General Procedure A**, **3aj** (32.7 mg, 82%) was prepared as a white solid; m.p. 71-72 ºC; ^{1}H NMR (400 MHz, Chloroform-*d*) δ 7.55 (s, 1H), 7.33 – 7.29 (m, 4H), 7.27 – 7.25 (m, 2H), 7.23 – 7.11 (m, 7H), 7.07 – 7.02 (m, 2H), 6.96 – 6.84 (m, 3H), 5.80 (s, 1H), 4.62 (t, *J* = 9.0 Hz, 1H), 3.03 – 2.88 (m, 2H), 2.29 – 2.15 (m, 2H); ^{13}C NMR (101 MHz,

Chloroform-*d*) δ 146.2, 143.9, 142.4, 136.2, 135.8, 129.0, 129.0, 128.8, 128.7, 127.4, 127.0, 126.9, 126.34, 126.26, 124.9, 124.4, 121.3, 120.2, 119.0, 115.0, 110.8, 48.3, 42.2, 33.9, 32.0. HRMS (ESI): Calcd for $[C_{30}H_{25}N+H]^+$: 400.2065, found: 400.2065.

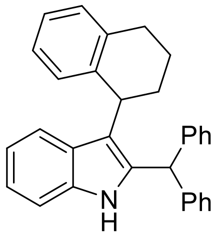

2-Benzhydryl-3-(1,2,3,4-tetrahydronaphthalen-1-yl)-1*H*-indole (**3ak**): According to the **General Procedure A**, **3ak** (33.0 mg, 80%) was prepared as a white solid; m.p. 76-77 ºC; ^{1}H NMR (400 MHz, Chloroform-*d*) δ 7.57 (s, 1H), 7.36 – 7.28 (m, 5H), 7.27 – 7.22 (m, 2H), 7.22 – 7.18 (m, 2H), 7.17 – 7.14 (m, 1H), 7.13 – 7.06 (m, 5H), 6.97 – 6.90 (m, 3H), 5.71 (s, 1H), 4.42 (dd, *J* = 9.9, 5.8 Hz, 1H), 3.00 – 2.82 (m, 2H), 1.99 – 1.70 (m, 4H); ^{13}C NMR (101 MHz, Chloroform-*d*) δ 142.44, 142.39, 139.5, 137.4, 136.1, 135.8, 129.6, 129.1, 129.0, 128.9, 128.8, 128.7, 127.6, 127.0, 126.9, 125.9, 125.7, 121.3, 120.1, 119.2, 117.4, 110.8, 48.2, 36.6, 31.6, 30.3, 23.1. HRMS (ESI): Calcd for $[C_{31}H_{27}N+Na]^+$: 436.2041, found: 436.2035.

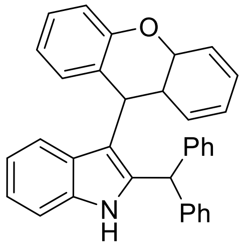

2-Benzhydryl-3-(4a,9a-dihydro-9*H*-xanthen-9-yl)-1*H*-indole (**3al**): According to the **General Procedure A**, but with a reaction time of 4 h instead of 1 h, **3al** (28.4 mg, 61%) was prepared as a white solid; m.p. 150-151 ºC; ^{1}H NMR (400 MHz, Chloroform-*d*) δ 7.65 (s, 1H), 7.29 – 7.21 (m, 9H), 7.10 – 7.03 (m, 8H), 6.91 (t, *J* = 7.5 Hz, 1H), 6.84 – 6.73 (m, 4H), 5.82 (s, 1H), 5.71 (s, 1H); ^{13}C NMR (101 MHz, Chloroform-*d*) δ 151.0, 141.8, 136.8, 135.6, 129.6, 128.9, 128.74, 128.66, 127.7, 127.5, 127.01, 126.96, 123.5, 123.0, 121.7, 119.8, 119.5, 116.8, 116.1, 110.8, 48.2, 33.6. HRMS (ESI): Calcd for

$[C_{34}H_{27}NO+H]^+$: 466.2171, found: 466.2171.

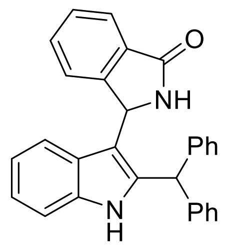

3-(2-Benzhydryl-1*H*-indol-3-yl)isoindolin-1-one (**3am**): According to the **General Procedure A**, but with a reaction time of 4 h instead of 1 h, **3am** (21.9 mg, 53%) was prepared as a white solid; m.p. 196-197 ºC; ^{1}H NMR (400 MHz, Chloroform-*d*) δ 7.89 (d, *J* = 7.8 Hz, 2H), 7.44 (t, *J* = 7.4 Hz, 1H), 7.38 (t, *J* = 7.6 Hz, 3H), 7.35 – 7.27 (m, 4H), 7.22 (d, *J* = 8.0 Hz, 3H), 7.16 – 7.03 (m, 3H), 6.97 (d, *J* = 7.5 Hz, 1H), 6.85 (s, 1H), 6.13 (s, 1H), 5.93 (s, 2H); ^{13}C NMR (101 MHz, Chloroform-*d*) δ 170.3, 147.4, 141.5, 141.3, 135.2, 132.1, 131.7, 129.1, 129.03, 128.98, 128.8, 128.2, 127.5, 127.4, 123.6, 123.5, 122.1, 120.2, 111.1, 107.7, 53.2, 48.5. HRMS (ESI): Calcd for $[C_{29}H_{22}N_2O+H]^+$: 415.1810, found: 415.1822.

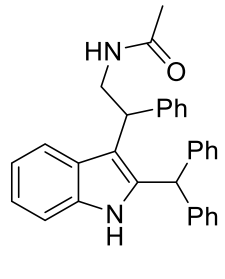

N-(2-(2-benzhydryl-1*H*-indol-3-yl)-2-phenylethyl)acetamide (**3an**): According to the **General Procedure A**, but with a reaction time of 4 h instead of 1 h, **3an** (22.2 mg, 50%) was prepared as a white solid; m.p. 196-197 ºC; ^{1}H NMR (400 MHz, Chloroform-*d*) δ 7.79 (s, 1H), 7.46 (d, *J* = 6.2 Hz, 1H), 7.32 (d, *J* = 7.5 Hz, 2H), 7.29 – 7.22 (m, 10H), 7.16 (d, *J* = 8.5 Hz, 3H), 7.07 – 7.00 (m, 3H), 5.75 (s, 1H), 5.15 (s, 1H), 4.51 (dd, *J* = 10.2, 5.7 Hz, 1H), 4.34 – 4.23 (m, 1H), 3.76 – 3.70 (m, 1H), 1.54 (s, 3H); ^{13}C NMR (101 MHz, Chloroform-*d*) δ 170.1, 142.1, 141.9, 141.6, 137.7, 135.9, 129.1, 128.9, 128.5, 127.8, 127.4, 127.3, 127.2, 126.5, 121.9, 120.1, 119.9, 112.1, 111.2, 48.1, 42.6,

41.2, 23.1. HRMS (ESI): Calcd for $[C_{31}H_{28}N_2O+Na]^+$: 467.2099, found: 467.2097.

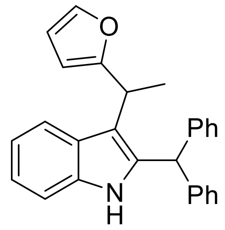

2-Benzhydryl-3-(1-(furan-2-yl)ethyl)-1*H*-indole (**3ao**): According to the **General Procedure A**, 0.25 M TsOH was employed in place of 0.25 M HCl, **3ao** (24.5 mg, 65%) was prepared as a yellow oil; ^{1}H NMR (400 MHz, Chloroform-*d*) δ 8.70 (s, 1H), 7.52 (d, *J* = 7.8 Hz, 1H), 7.33 – 7.25 (m, 7H), 7.15 (td, *J* = 8.2, 7.6, 1.3 Hz, 1H), 7.10 – 7.00 (m, 5H), 6.10 (dd, *J* = 2.2, 0.9 Hz, 1H), 5.93 –5.92 (m, 1H), 5.86 (d, *J* = 3.2 Hz, 1H), 2.69 (q, *J* = 7.5 Hz, 2H), 1.24 (t, *J* = 7.5 Hz, 3H); ^{13}C NMR (101 MHz, Chloroform-*d*) δ 158.1, 155.6, 143.6, 142.1, 136.2, 129.6, 127.9, 127.4, 127.3, 121.8, 120.5, 119.7, 111.8, 110.8, 104.6, 104.3, 56.9, 21.6, 12.3. HRMS (ESI): Calcd for $[C_{27}H_{23}NO+H]^+$: 378.1858, found: 378.1858.

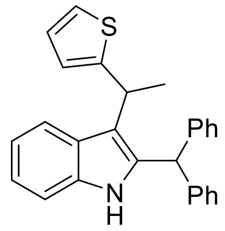

2-Benzhydryl-3-(1-(thiophen-2-yl)ethyl)-1*H*-indole (**3ap**): According to the **General Procedure A**, 0.1 M HCl was employed in place of 0.25 M HCl, **3ap** (23.6 mg, 60%) was prepared as a yellow oil; ^{1}H NMR (400 MHz, Chloroform-*d*) δ 7.51 (s, 1H), 7.41 (d, *J* = 8.0 Hz, 1H), 7.33 – 7.25 (m, 7H), 7.17 – 7.09 (m, 6H), 6.99 (t, *J* = 7.5 Hz, 1H), 6.90 (dd, *J* = 5.1, 3.5 Hz, 1H), 6.83 – 6.80 (m, 1H), 5.79 (s, 1H), 4.58 (q, *J* = 6.9 Hz, 1H), 1.67 (d, *J* = 7.2 Hz, 3H); ^{13}C NMR (101 MHz, Chloroform-*d*) δ 150.9, 142.2, 135.7, 135.2, 129.2, 129.1, 129.0, 128.8, 127.2, 127.1, 127.0, 126.5, 123.59, 123.56, 121.5, 120.1, 119.4, 116.8, 111.0, 48.3, 31.8, 21.4. HRMS (ESI): Calcd for $[C_{27}H_{23}NS+H]^+$: 394.1629, found: 394.1629.

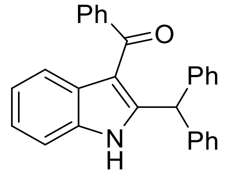

(2-Benzhydryl-1*H*-indol-3-yl)(phenyl)methanone (**3aq**): According to the **General Procedure B**, 0.25 M TsOH was employed in place of 0.1 M TsOH, **3aq** (25.9 mg, 67%) was prepared as a red solid; m.p. 124-126 ºC; ^{1}H NMR (400 MHz, Chloroform-*d*) δ 8.25 (s, 1H), 7.72 – 7.65 (m, 2H), 7.52 (t, J = 7.4 Hz, 1H), 7.39 (t, J = 7.6 Hz, 2H), 7.34 – 7.24 (m, 8H), 7.21 – 7.11 (m, 5H), 7.06 (t, J = 7.6 Hz, 1H), 6.37 (s, 1H); ^{13}C NMR (101 MHz, Chloroform-*d*) δ 192.8, 147.0, 141.4, 141.0, 134.6, 131.6, 129.0, 128.9, 128.8, 128.2, 127.5, 127.1, 122.7, 121.6, 121.4, 114.1, 111.1, 48.9. HRMS (ESI): Calcd for $[C_{28}H_{21}NO+H]^+$: 388.1701, found: 388.1701.

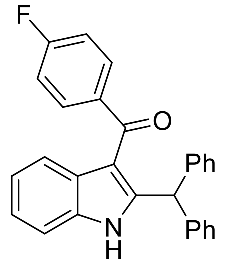

(2-Benzhydryl-1*H*-indol-3-yl)(4-fluorophenyl)methanone (**3ar**): According to the **General Procedure B**, 0.1 M HCl was employed in place of 0.1 M TsOH, **3ar** (24.3 mg, 60%) was prepared as a red solid; m.p. 116-118 ºC; ^{1}H NMR (400 MHz, Chloroform-*d*) δ 8.39 (s, 1H), 7.71 (dd, J = 8.7, 5.5 Hz, 2H), 7.31 – 7.22 (m, 8H), 7.18 – 7.11 (m, 5H), 7.09 – 7.02 (m, 3H), 6.36 (s, 1H); ^{13}C NMR (101 MHz, Chloroform-*d*) δ 191.3, 164.9 (d, $J_{C\text{-}F}$ = 253.2 Hz), 147.2, 141.4, 137.1 (d, $J_{C\text{-}F}$ = 2.9 Hz), 134.7, 131,5 (d, $J_{C\text{-}F}$ = 8.9 Hz), 129.0, 128.9, 127.4, 127.2, 122.8, 121.7, 121.2, 115.3 (d, $J_{C\text{-}F}$ = 21.7 Hz), 113.8, 111.4, 48.9; ^{19}F NMR (376 MHz, Chloroform-*d*) δ -107.52. HRMS (ESI): Calcd for $[C_{28}H_{20}NOF+H]^+$: 406.1607, found: 406.1607.

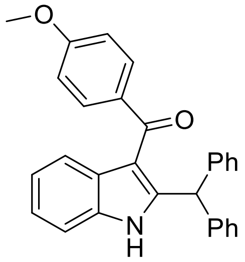

(2-Benzhydryl-1*H*-indol-3-yl)(4-methoxyphenyl)methanone (**3as**): According to the **General Procedure B**, 0.1 M HCl was employed in place of 0.1 M TsOH, **3as** (21.7 mg, 52%) was prepared as a red solid; m.p. 121-122 ºC; ^{1}H NMR (400 MHz, Chloroform-*d*) δ 8.35 (s, 1H), 7.73 (d, *J* = 8.8 Hz, 2H), 7.33 – 7.22 (m, 8H), 7.17 – 7.12 (m, 5H), 7.06 (t, *J* = 7.6 Hz, 1H), 6.89 – 6.85 (m, 2H), 6.38 (s, 1H), 3.85 (s, 3H); ^{13}C NMR (101 MHz, Chloroform-*d*) δ 191.7, 162.7, 146.3, 141.6, 134.7, 133.4, 131.5, 129.0, 128.8, 127.5, 127.0, 122.6, 121.40, 121.39, 114.4, 113.4, 111.2, 55.4, 48.7. HRMS (ESI): Calcd for $[C_{29}H_{23}NO_2+H]^+$: 418.1807, found: 418.1807.

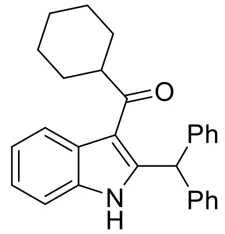

(2-Benzhydryl-1*H*-indol-3-yl)(cyclohexyl)methanone (**3at**): According to the **General Procedure B**, **3at** (29.9 mg, 76%) was prepared as a red solid; m.p. 123-125 ºC; ^{1}H NMR (400 MHz, Chloroform-*d*) δ 8.21 (s, 1H), 7.89 (d, *J* = 8.5 Hz, 1H), 7.30 (dd, *J* = 8.1, 6.2 Hz, 5H), 7.27 – 7.22 (m, 4H), 7.14 – 7.11 (m, 4H), 6.66 (s, 1H), 3.19 (tt, *J* = 11.3, 3.2 Hz, 1H), 1.89 (dd, *J* = 13.0, 3.2 Hz, 2H), 1.80 (dt, *J* = 12.6, 3.2 Hz, 2H), 1.73 – 1.67 (m, 1H), 1.51 – 1.26 (m, 5H); ^{13}C NMR (101 MHz, Chloroform-*d*) δ 200.7, 147.4, 141.5, 134.7, 129.1, 128.8, 127.1, 126.3, 122.5, 122.2, 120.9, 113.7, 111.6, 49.5, 28.9, 26.1, 26.1. HRMS (ESI): Calcd for $[C_{28}H_{27}NO+H]^+$: 394.2171, found: 394.2171.

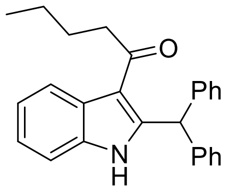

1-(2-Benzhydryl-1*H*-indol-3-yl)pentan-1-one (**3au**): According to the **General Procedure B**, **3au** (23.8 mg, 65%) was prepared as a red solid; m.p. 104-106 ºC; ^{1}H NMR (400 MHz, Chloroform-*d*) δ 8.22 (s, 1H), 7.99 (d, *J* = 7.9 Hz, 1H), 7.33 – 7.21 (m, 9H), 7.15 – 7.11 (m, 4H), 6.69 (s, 1H), 3.02 – 2.91 (m, 2H), 1.72 – 1.65 (m, 2H), 1.39 –1.32 (m, 2H), 0.90 (t, *J* = 7.3 Hz, 3H); ^{13}C NMR (101 MHz, Chloroform-*d*) δ 197.1, 147.2, 141.3, 134.6, 129.1, 128.8, 127.1, 126.6, 122.5, 122.1, 121.3, 114.3, 111.5, 49.5, 43.1, 26.2, 22.5, 14.1. HRMS (ESI): Calcd for $[C_{26}H_{25}NO+H]^+$: 368.2014, found: 368.2014.

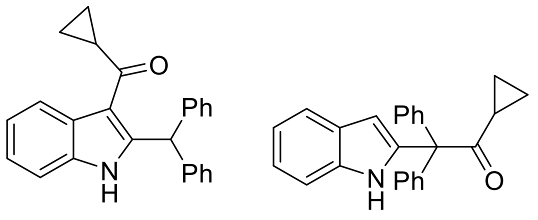

(2-Benzhydryl-1*H*-indol-3-yl)(cyclopropyl)methanone (**3av, major**); 1-Cyclopropyl-2-(1*H*-indol-2-yl)-2,2-diphenylethan-1-one (**3av, minor**): According to the **General Procedure B**, **3av** (26.3 mg, 75%) was prepared as a red solid; m.p. 115-116 ºC; (**3av, major**): ^{1}H NMR (400 MHz, Chloroform-*d*) δ 8.26 (s, 1H), 8.11 (d, *J* = 7.8 Hz, 1H), 7.31 – 7.20 (m, 9H), 7.15 – 7.11 (m, 4H), 6.58 (s, 1H), 2.59 (tt, *J* = 7.9, 4.7 Hz, 1H), 1.17 (dt, *J* = 4.8, 3.3 Hz, 2H), 0.92 (dt, *J* = 7.9, 3.4 Hz, 2H); ^{13}C NMR (101 MHz, Chloroform-*d*) δ 196.9, 145.6, 141.5, 134.9, 129.1, 128.9, 127.1, 126.9, 122.7, 122.0, 121.1, 115.5, 111.5, 49.2, 21.3, 10.1. (**3av, minor**): ^{1}H NMR (400 MHz, Chloroform-*d*) δ 9.10 (s, 1H), 7.50 (d, *J* = 7.8 Hz, 1H), 7.34 – 7.27 (m, 7H), 7.22 – 7.19 (m, 4H), 7.16 – 7.11 (m, 1H), 7.07 – 7.03 (m, 1H), 6.09 (s, 1H), 2.06 (tt, *J* = 7.7, 4.5 Hz, 1H), 1.23 – 1.19 (m, 2H), 0.95 –0.91 (m, 2H); ^{13}C NMR (101 MHz, Chloroform-*d*) δ 211.4, 141.9, 140.8, 136.4, 129.8, 129.0, 128.6, 128.3, 127.5, 127.2, 121.9, 120.4, 119.6, 111.1,

105.0, 68.0, 22.5, 14.3. HRMS (ESI): Calcd for $[C_{25}H_{21}NO+H]^+$: 352.1701, found: 352.1701.

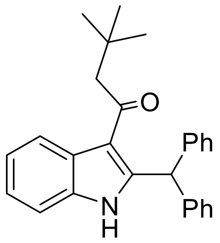

1-(2-Benzhydryl-1*H*-indol-3-yl)-3,3-dimethylbutan-1-one (**3aw**): According to the **General Procedure B**, **3aw** (27.8 mg, 73%) was prepared as a red solid; m.p. 108-109 ºC; ^{1}H NMR (400 MHz, Chloroform-*d*) δ 9.09 (s, 1H), 7.52 (d, *J* = 7.8 Hz, 1H), 7.32 – 7.29 (m, 7H), 7.24 – 7.21 (m, 4H), 7.16 – 7.12 (m, 1H), 7.08 – 7.04 (m, 1H), 6.23 (s, 1H), 2.48 (s, 2H), 0.94 (s, 9H); ^{13}C NMR (101 MHz, Chloroform-*d*) δ 210.7, 141.9, 140.4, 136.2, 129.8, 128.2, 127.5, 127.3, 121.9, 120.4, 119.6, 111.0, 105.0, 68.5, 54.3, 30.9, 29.5. HRMS (ESI): Calcd for $[C_{27}H_{27}NO+H]^+$: 382.2171, found: 382.2171.

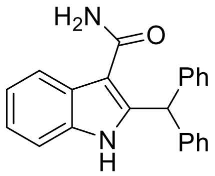

2-Benzhydryl-1*H*-indole-3-carboxamide (**3ax**): According to the **General Procedure B**, 0.25 M HCl was employed in place of 0.1 M TsOH, **3ax** (13.4 mg, 41%) was prepared as a red solid; m.p. 180-182 ºC; ^{1}H NMR (400 MHz, Chloroform-*d*) δ 8.15 (s, 1H), 7.89 (d, *J* = 7.3 Hz, 1H), 7.36 – 7.15 (m, 13H), 6.62 (s, 1H), 5.62 (s, 2H); ^{13}C NMR (101 MHz, Chloroform-*d*) δ 167.5, 144.3, 141.3, 134.8, 129.1, 129.0, 127.3, 126.4, 122.6, 121.7, 119.9, 111.5, 107.9, 48.6. HRMS (ESI): Calcd for $[C_{22}H_{18}NO_2+H]^+$: 327.1497, found: 327.1497.

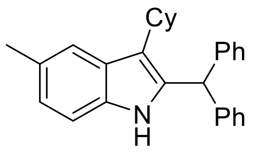

2-Benzhydryl-3-cyclohexyl-5-methyl-1*H*-indole (**3ay**): According to the **General Procedure A**, **3ay** (30.2 mg, 81%) was prepared as a white solid; m.p. 148-149 ºC; ^{1}H NMR (400 MHz, Chloroform-*d*) δ 7.52 (s, 1H), 7.31 – 7.26 (m, 4H), 7.26 – 7.21 (m, 3H), 7.15 – 7.11 (m, 4H), 7.10 – 7.07 (m, 1H), 6.94 – 6.87 (m, 1H), 5.82 (s, 1H), 2.70 (tt, *J* = 12.2, 3.6 Hz, 1H), 2.44 (s, 3H), 1.95 – 1.83 (m, 2H), 1.81 – 1.73 (m, 2H), 1.70 – 1.62 (m, 2H), 1.31 – 1.21 (m, 4H); ^{13}C NMR (101 MHz, Chloroform-*d*) δ 142.6, 134.4, 134.0, 129.1, 128.7, 128.1, 127.8, 126.8, 122.6, 120.1, 117.9, 110.7, 48.5, 36.7, 32.9, 27.4, 26.4, 21.8. HRMS (ESI): Calcd for $[C_{28}H_{29}N+H]^{+}$: 380.2378, found: 380.2356.

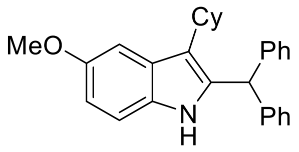

2-Benzhydryl-3-cyclohexyl-5-methoxy-1*H*-indole (**3az**): According to the General Procedure A, 0.25 M TsOH was employed in place of 0.25 M HCl, **3az** (21.7 mg, 55%) was prepared as a white solid; m.p. 156-158 ºC; ^{1}H NMR (400 MHz, Chloroform-*d*) δ 7.35 – 7.27 (m, 5H), 7.27 – 7.24 (m, 2H), 7.21 (d, *J* = 2.4 Hz, 1H), 7.17 – 7.07 (m, 5H), 6.76 (dd, *J* = 8.8, 2.4 Hz, 1H), 5.82 (s, 1H), 3.86 (s, 3H), 2.69 (tt, *J* = 12.3, 3.6 Hz, 1H), 1.91 – 1.85 (m, 2H), 1.80 – 1.74 (m, 2H), 1.68 –1.63 (m, 2H), 1.30 – 1.22 (m, 4H); ^{13}C NMR (101 MHz, Chloroform-*d*) δ 153.4, 142.4, 135.3, 131.0, 129.0, 128.6, 128.0, 126.8, 118.0, 111.4, 110.4, 103.3, 56.2, 48.6, 36.6, 32.7, 27.3, 26.4. HRMS (ESI): Calcd for $[C_{28}H_{29}NO+H]+$: 396.2327, found: 396.2327.

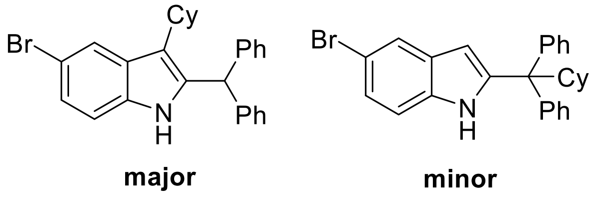

2-Benzhydryl-5-bromo-3-cyclohexyl-1*H*-indole (**3ba, major**); 5-Bromo-2-(cyclohexyldiphenylmethyl)-1*H*-indole (**3ba, minor**): According to the **General Procedure A**, **3ba** (27.1 mg, 61%) was prepared as a white solid; m.p. 84-86 ºC; ^{1}H NMR (400 MHz, Chloroform-*d*) δ 7.84 and 7.67 (s, 1H), 7.64 and 7.45 (s, 1H), 7.34 – 7.22 (m, 8H), 7.17 – 7.06 (m, 4H), 6.46 and 5.82 (s, 1H), 2.95 –2.89 and 2.71 – 2.63 (m, 1H), 1.86 – 1.60 (m, 6H), 1.53 – 1.38 (m, 1H), 1.32 – 1.21 (m, 2H), 0.92 – 0.56 (m, 1H); ^{13}C NMR (101 MHz, Chloroform-*d*) δ 142.1, 135.7, 134.3, 129.4, 129.0, 128.8, 128.0, 127.1, 126.4, 124.1, 123.8, 122.8, 122.7, 118.1, 112.4, 112.2, 112.0, 58.1, 48.5, 44.3, 36.5, 32.9, 29.8, 27.3, 26.6, 26.3. HRMS (ESI): Calcd for $[C_{27}H_{26}NBr+H]^+$: 444.1327, found: 444.1322.

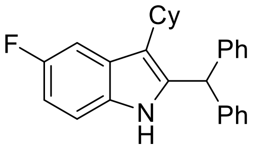

major

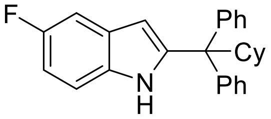

minor

2-Benzhydryl-3-cyclohexyl-5-fluoro-1*H*-indole (**3bb, major**); 2-(Cyclohexyldiphenylmethyl)-5-fluoro-1*H*-indole (**3bb, minor**): According to the **General Procedure A**, **3bb** (27.6 mg, 72%) was prepared as a white solid; m.p. 143-144 ºC; ^{1}H NMR (400 MHz, Chloroform-*d*) δ 7.41 – 7.38 (m, 2H), 7.33 – 7.24 (m, 7H), 7.16 – 7.12 (m, 4H), 6.87 – 6.80 (m, 1H), 6.50 and 5.83 (s, 1H), 2.98 – 2.91 and 2.72 – 2.63 (m, 1H), 1.85 – 1.63 (m, 7H), 1.34 – 1.20 (m, 3H); ^{13}C NMR (101 MHz, Chloroform-*d*) δ 157.3 (d, $J_{C\text{-}F}$ = 234.4 Hz), 142.3, 136.3, 132.2, 129.6, 129.1, 128.8, 128.4, 128.0, 127.8, 127.0, 126.4, 118.5 (d, $J_{C\text{-}F}$ = 4.3 Hz), 111.4 (d, $J_{C\text{-}F}$ = 9.8 Hz), 109.3, 109.1, 105.4 (d, $J_{C\text{-}F}$ = 24.2 Hz), 58.2, 48.6, 44.3, 36.5, 32.7, 29.8, 27.3, 26.7, 26.3; ^{19}F NMR (376 MHz, Chloroform-*d*) δ -124.9, -125.0. HRMS (ESI): Calcd for $[C_{27}H_{26}NF+H]^+$: 384.2128, found: 384.2118.

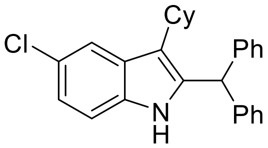

2-Benzhydryl-5-chloro-3-cyclohexyl-1*H*-indole (**3bc**): According to the **General Procedure A**, **3bc** (25.9 mg, 65%) was prepared as a white solid; m.p. 134-135 ºC; ^{1}H NMR (400 MHz, Chloroform-*d*) δ 7.69 (d, *J* = 1.9 Hz, 1H), 7.44 (s, 1H), 7.34 – 7.25 (m, 7H), 7.12 (dd, *J* = 6.9, 1.4 Hz, 4H), 7.02 (dd, *J* = 8.6, 2.0 Hz, 1H), 5.82 (s, 1H), 2.67 (tt, *J* = 12.2, 3.5 Hz, 1H), 1.87 – 1.71 (m, 5H), 1.63 (d, *J* = 12.5 Hz, 2H), 1.29 – 1.24 (m, 3H); ^{13}C NMR (101 MHz, Chloroform-*d*) δ 142.2, 135.9, 134.0, 129.0, 128.8, 127.0, 124.6, 121.2, 119.8, 118.2, 111.9, 48.5, 36.5, 32.9, 27.3, 26.3. HRMS (ESI): Calcd for $[C_{27}H_{26}NCl+H]^+$: 400.1832, found: 400.1846.

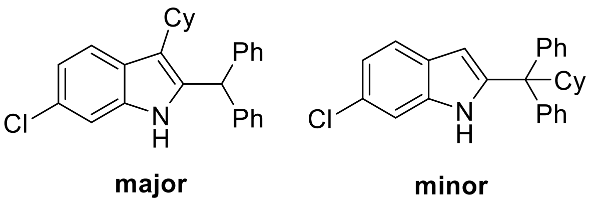

2-Benzhydryl-6-chloro-3-cyclohexyl-1*H*-indole (**3bd, major**); 6-Chloro-2-(cyclohexyldiphenylmethyl)-1*H*-indole (**3bd, minor**): According to the **General Procedure A**, **3bd** (30.3 mg, 76%) was prepared as a white solid; m.p. 170-171 ºC; ^{1}H NMR (400 MHz, Chloroform-*d*) δ 7.64 and 7.45 (d, *J* = 8.6 Hz, 1H), 7.61 and 7.42 (s, 1H), 7.34 – 7.24 (m, 7H), 7.18 (d, *J* = 1.9 Hz, 1H), 7.14 – 7.11 (m, 3H), 7.01 (dd, *J* = 8.5, 1.9 Hz, 1H), 6.51 and 5.83 (s, 1H), 2.96 – 2.90 and 2.74 – 2.66 (m, 1H), 1.89 – 1.70 (m, 5H), 1.69 – 1.59 (m, 2H), 1.30 – 1.23 (m, 3H); ^{13}C NMR (101 MHz, Chloroform-*d*) δ 142.2, 136.1, 134.9, 129.0, 128.8, 128.0, 127.04, 126.96, 126.4, 126.2, 121.3, 121.1, 120.3, 119.6, 118.4, 110.9, 110.6, 48.4, 44.3, 36.5, 33.0, 27.3, 26.4. HRMS (ESI): Calcd for $[C_{27}H_{26}NCl+H]^+$: 400.1832, found: 400.1822.

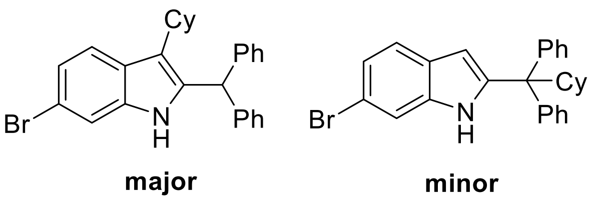

2-Benzhydryl-6-bromo-3-cyclohexyl-1*H*-indole (**3be, major**); 6-Bromo-2-(cyclohexyldiphenylmethyl)-1*H*-indole (**3be, minor**): According to the **General**

Procedure A, 0.1 ml 1 M HCl and 0.9 ml MeCN was employed in place of 0.15 ml 0.25 M HCl 0.85 ml MeCN**, 3be** (22.2 mg, 50%) was prepared as a white solid; m.p. 175-177 ºC; ^{1}H NMR (400 MHz, Chloroform-*d*) δ 7.60 (d, *J* = 8.5 Hz, 1H), 7.42 (s, 1H), 7.35 – 7.25 (m, 8H), 7.12 (d, *J* = 7.3 Hz, 4H), 6.50 and 5.82 (s, 1H), 2.96 – 2.89 and 2.74 – 2.66 (tt, *J* = 12.2, 3.4 Hz, 1H), 1.86 – 1.70 (m, 5H), 1.65 (d, *J* = 12.7 Hz, 2H), 1.29 – 1.22 (m, 3H); ^{13}C NMR (101 MHz, Chloroform-*d*) δ 142.2, 136.5, 134.9, 129.0, 128.8, 128.0, 127.0, 126.5, 122.1, 121.6, 118.5, 114.6, 113.9, 48.4, 36.5, 33.0, 27.3, 26.4. HRMS (ESI): Calcd for $[C_{27}H_{26}NBr+H]^+$: 444.1327, found: 444.1314.

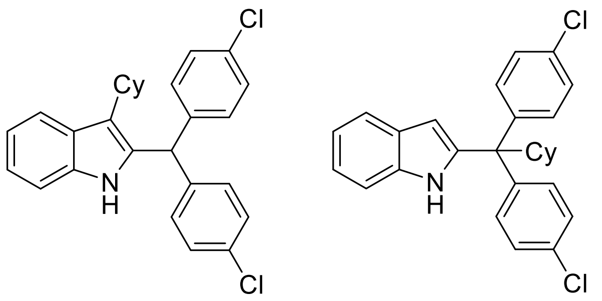

major **minor**

2-(Bis(4-chlorophenyl)-methyl)-3-cyclohexyl-1*H*-indole (**3bf, major**); 2-(Bis(4-chlorophenyl)(cyclohexyl)methyl)-1*H*-indole (**3bf, minor**): According to the **General Procedure A**, 0.1 ml 1 M HCl and 0.9 ml MeCN was employed in place of 0.15 ml 0.25 M HCl 0.85 ml MeCN, **3bf** (26.1 mg, 60%) was prepared as a white solid; m.p. 81-82 ºC; ^{1}H NMR (400 MHz, Chloroform-*d*) δ 7.76 (d, *J* = 7.8 Hz, 1H), 7.72 (d, *J* = 8.7 Hz, 1H), 7.47 (d, *J* = 8.7 Hz, 1H), 7.33 (s, 1H), 7.29 – 7.24 (m, 5H), 7.11 – 7.08 (m, 1H), 7.04 (d, *J* = 8.3 Hz, 3H), 6.49 and 5.79 (s, 1H), 2.87 and 2.68 (tt, *J* = 12.2, 3.6 Hz, 1H), 1.98 – 1.82 (m, 3H), 1.82 – 1.76 (m, 2H), 1.65 (d, *J* = 12.1 Hz, 2H), 1.31 – 1.23 (m, 3H); ^{13}C NMR (101 MHz, Chloroform-*d*) δ 140.6, 139.3, 135.8, 133.0, 132.9, 131.5, 130.3, 129.0, 128.9, 128.2, 127.5, 121.5, 120.6, 119.2, 118.8, 111.1, 47.2, 36.7, 33.0, 27.3, 26.4. HRMS (ESI): Calcd for $[C_{27}H_{25}NCl_2+H]^+$: 434.1442, found: 434.1463.

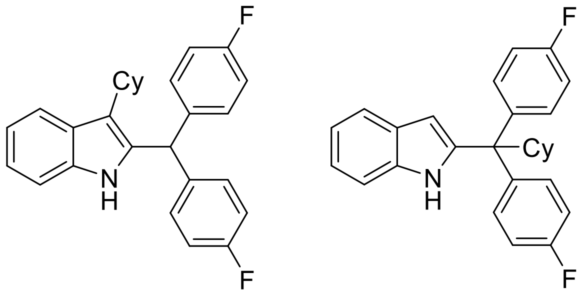

2-(Bis(4-fluorophenyl)-methyl)-3-cyclohexyl-1*H*-indole (**3bg, major**); 2-(Cyclohexylbis(4-fluorophenyl)methyl)-1*H*-indole (**3bg, minor**): According to the **General Procedure A**, **3bg** (24.5 mg, 61%) was prepared as a white solid; m.p. 63-64 ºC; ^{1}H NMR (400 MHz, Chloroform-*d*) δ 7.76 (d, *J* = 7.8 Hz, 1H), 7.35 (s, 1H), 7.26 – 7.21 (m, 1H), 7.12 – 7.05 (m, 6H), 7.00 (t, *J* = 8.6 Hz, 4H), 6.50 and 5.82 (s, 1H), 2.87 and 2.70 (tt, *J* = 12.2, 3.6 Hz, 1H), 1.94 – 1.71 (m, 6H), 1.65 (d, *J* = 13.7 Hz, 2H), 1.30 – 1.27 (m, 2H); ^{13}C NMR (101 MHz, Chloroform-*d*) δ 161.8 (d, $J_{C\text{-}F}$ = 247.5 Hz), 138.1 (d, $J_{C\text{-}F}$ = 2.9 Hz), 135.8, 133.7, 130.5 (d, $J_{C\text{-}F}$ = 8.1 Hz), 127.6, 121.4, 120.6, 119.1, 118.5, 115.7 (d, $J_{C\text{-}F}$ = 22.2 Hz), 111.1, 47.0, 36.7, 33.0, 27.3, 26.4; ^{19}F NMR (376 MHz, Chloroform-*d*) δ -105.53, -115.51. HRMS (ESI): Calcd for $[C_{27}H_{25}NF_2+H]^+$: 402.2033, found: 402.2036.

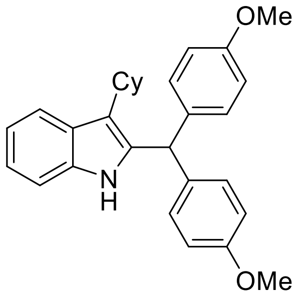

2-(Bis(4-methoxyphenyl)methyl)-3-cyclohexyl-1*H*-indole (**3bh**): According to the **General Procedure A**, 0.25 M TsOH was employed in place of 0.25 M HCl,, **3bh** (23.8 mg, 56%) was prepared as a white solid; m.p. 154-156 ºC; ^{1}H NMR (400 MHz, Chloroform-*d*) δ 7.75 (d, *J* = 7.4 Hz, 1H), 7.40 (s, 1H), 7.19 (d, *J* = 8.4 Hz, 1H), 7.12 – 6.98 (m, 6H), 6.82 (d, *J* = 8.7 Hz, 4H), 5.74 (s, 1H), 3.79 (s, 6H), 2.72 (tt, *J* = 12.2, 3.6 Hz, 1H), 1.95 – 1.86 (m, 2H), 1.78 (d, *J* = 9.2 Hz, 2H), 1.67 (d, *J* = 12.7 Hz, 2H), 1.28 (dd, *J* = 10.0, 6.3 Hz, 4H); ^{13}C NMR (101 MHz, Chloroform-*d*) δ 158.3, 135.6, 134.9,

129.9, 127.7, 120.8, 120.4, 118.8, 117.9, 113.9, 110.9, 55.3, 46.8, 36.6, 32.9, 27.3, 26.4. HRMS (ESI): Calcd for $[C_{29}H_{31}NO_2+H]^+$: 426.2433, found: 426.2433.

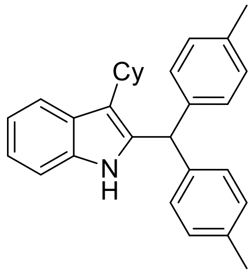

3-Cyclohexyl-2-(di-p-tolylmethyl)-1*H*-indole (**3bi**): According to the **General Procedure A**, **3bi** (24.4 mg, 62%) was prepared as a white solid; m.p. 57-58 ºC; ^{1}H NMR (400 MHz, Chloroform-*d*) δ 7.83 – 7.76 (m, 1H), 7.46 (s, 1H), 7.21 (dd, *J* = 6.9, 1.7 Hz, 1H), 7.13 – 7.10 (m, 5H), 7.07 – 7.02 (m, 5H), 5.79 (s, 1H), 2.76 (tt, *J* = 12.1, 3.4 Hz, 1H), 2.35 (s, 6H), 1.95 – 1.89 (m, 2H), 1.81 (d, *J* = 8.7 Hz, 2H), 1.71 (d, *J* = 14.8 Hz, 3H), 1.33 – 1.26 (m, 3H); ^{13}C NMR (101 MHz, Chloroform-*d*) δ 139.8, 136.4, 135.7, 134.7, 130.4, 129.4, 128.9, 120.9, 120.5, 118.8, 118.1, 111.0, 47.7, 36.7, 33.0, 27.4, 26.5, 21.2. HRMS (ESI): Calcd for $[C_{29}H_{31}N+H]^+$: 394.2535, found: 394.2538.

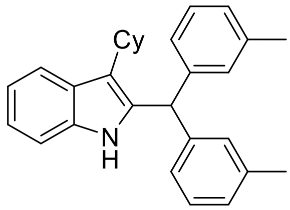

3-Cyclohexyl-2-(di-m-tolylmethyl)-1*H*-indole (**3bj**): According to the **General Procedure A, 3bj** (28.3 mg, 72%) was prepared as a white solid; m.p. 60-61 ºC; ^{1}H NMR (400 MHz, Chloroform-*d*) δ 7.80 (d, *J* = 8.8 Hz, 1H), 7.47 (s, 1H), 7.26 – 7.19 (m, 3H), 7.14 – 7.06 (m, 4H), 7.00 (s, 2H), 6.95 (d, *J* = 7.7 Hz, 2H), 5.80 (s, 1H), 2.76 (tt, *J* = 12.3, 3.6 Hz, 1H), 2.32 (s, 6H), 2.00 – 1.89 (m, 2H), 1.85 – 1.79 (m, 2H), 1.76 – 1.67 (m, 3H), 1.35 – 1.27 (m, 3H); ^{13}C NMR (101 MHz, Chloroform-*d*) δ 142.6, 138.3, 135.7, 134.5, 129.8, 128.6, 127.7, 127.6, 126.2, 120.9, 120.5, 118.8, 118.2, 111.0,

48.4, 36.7, 33.0, 27.4, 26.5, 21.6. HRMS (ESI): Calcd for $[C_{29}H_{31}N+H]^+$: 394.2535, found: 394.2539.

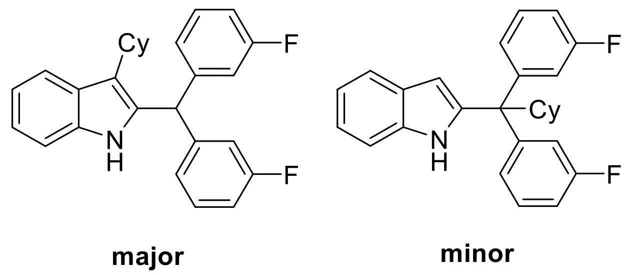

2-(Bis(3-fluorophenyl)-methyl)-3-cyclohexyl-1*H*-indole (**3bk, major**); 2-(Cyclohexylbis(3-fluorophenyl)-methyl)-1*H*-indole (**3bk, minor**): According to the **General Procedure A**, **3bk** (23.3 mg, 58%) was prepared as a white solid; m.p. 66-67 ºC; ^{1}H NMR (400 MHz, Chloroform-*d*) δ 7.77 and 7.31 (d, *J* = 7.8 Hz, 1H), 7.60 and 7.40 (s, 1H), 7.30 – 7.21 (m, 3H), 7.14 – 7.06 (m, 3H), 7.01 – 6.82 (m, 5H), 6.53 and 5.84 (s, 1H), 2.87 and 2.71 (tt, *J* = 12.3, 3.6 Hz, 1H), 2.00 – 1.65 (m, 7H), 1.46 – 1.23 (m, 3H); ^{13}C NMR (101 MHz, Chloroform-*d*) δ 163.2 (d, J_{C-F} = 248.1 Hz), 144.5, 144.4, 135.9, 132.6, 130.3 (d, J_{C-F} = 8.4 Hz), 129.4 (d, J_{C-F} = 8.8 Hz), 127.8, 127.5, 125.2, 124.7, 124.7, 121.8, 121.5, 120.7, 120.5, 119.9, 119.2, 118.9, 116.0 (d, J_{C-F} = 22.2 Hz), 114.2 (d, J_{C-F} = 22.2 Hz), 113.7, 113.4, 111.1, 110.7, 58.1, 47.9, 44.5, 36.8, 33.0, 29.7, 27.3, 27.2, 26.6, 26.4; ^{19}F NMR (376 MHz, Chloroform-*d*) δ -112.11, -112.14, -112.15, -112.18. HRMS (ESI): Calcd for $[C_{27}H_{25}NF_2+H]^+$: 402.2033, found: 402.2035.

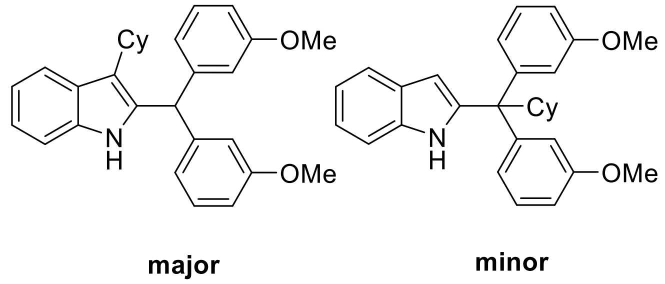

2-(Bis(3-methoxyphenyl)methyl)-3-cyclohexyl-1*H*-indole (**3bl, major**); 2-(Cyclohexylbis(3-methoxyphenyl)methyl)-1*H*-indole (**3bl, minor**): According to the **General Procedure A**, 0.25 M TsOH was employed in place of 0.25 M HCl,, **3bl** (24.2 mg, 57%) was prepared as a white solid; m.p. 138-140 ºC; ^{1}H NMR (400 MHz,

Chloroform-*d*) δ 7.75 and 7.56 (d, *J* = 7.6 Hz, 1H), 7.64 and 7.47 (s, 1H), 7.19 (dt, *J* = 13.6, 8.1 Hz, 3H), 7.10 – 7.02 (m, 2H), 6.93 (s, 1H), 6.83 – 6.67 (m, 5H), 6.52 and 5.77 (s, 1H), 3.74 (s, 6H), 2.89 and 2.73 (tt, *J* = 12.2, 3.5 Hz, 1H), 2.03 – 1.84 (m, 2H), 1.83 – 1.73 (m, 2H), 1.72 – 1.66 (m, 2H), 1.34 – 1.22 (m, 4H); ^{13}C NMR (101 MHz, Chloroform-*d*) δ 159.8, 159.0, 144.0, 135.7, 133.8, 129.6, 128.7, 127.6, 121.5, 121.3, 121.0, 120.4, 120.2, 119.5, 118.8, 118.3, 115.1, 111.8, 110.9, 110.7, 110.5, 58.1, 55.2, 48.4, 44.6, 36.6, 32.9, 27.3, 27.2, 26.6, 26.4. HRMS (ESI): Calcd for $[C_{29}H_{31}NO_2+H]^+$: 426.2433, found: 426.2447.

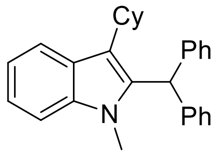

2-Benzhydryl-3-cyclohexyl-1-methyl-1*H*-indole (**3bm**): According to the **General Procedure A**, **3bm** (20.0 mg, 53%) was prepared as a white solid; m.p. 43-45 ºC; ^{1}H NMR (400 MHz, Chloroform-*d*) δ 7.81 (d, *J* = 8.0 Hz, 1H), 7.29 (dd, *J* = 8.0, 6.4 Hz, 4H), 7.26 – 7.22 (m, 3H), 7.16 (d, *J* = 7.1 Hz, 5H), 7.06 (t, *J* = 7.0 Hz, 1H), 6.02 (s, 1H), 3.41 (s, 3H), 2.42 (tt, *J* = 12.2, 3.5 Hz, 1H), 1.97 – 1.86 (m, 2H), 1.72 – 1.61 (m, 3H), 1.32 – 0.96 (m, 5H); ^{13}C NMR (101 MHz, Chloroform-*d*) δ 141.4, 137.3, 135.7, 129.3, 128.4, 126.6, 126.6, 120.9, 120.8, 119.2, 118.3, 109.0, 47.7, 36.4, 32.9, 30.8, 27.2, 26.4. HRMS (ESI): Calcd for $[C_{28}H_{29}N+H]^+$: 380.2378, found: 380.2356.

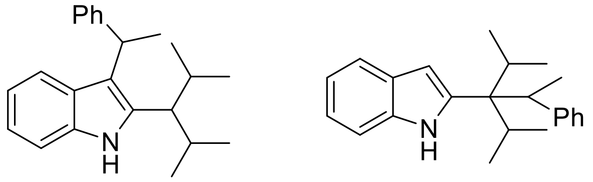

major **minor**

2-(2,4-Dimethylpentan-3-yl)-3-(1-phenylethyl)-1*H*-indole (**3bn, major**); 2-(3-Isopropyl-2-methyl-4-phenylpentan-3-yl)-1*H*-indole (**3bn, minor**): According to the **General Procedure A**, **3bn** (17.9 mg, 56%) was prepared as a yellow oil; ^{1}H NMR (400 MHz, Chloroform-*d*) δ 7.79 and 7.67 (s, 1H), 7.59 and 7.55 (d, *J* = 7.4 Hz, 1H),

7.36 (d, J = 7.8 Hz, 1H), 7.32 – 7.26 (m, 2H), 7.25 – 6.81 (m, 5H), 6.19 and 4.40 –4.35 (m, 1H), 2.61 and 2.25 (t, J = 7.5 Hz, 1H), 2.16 – 2.06 (m, 2H), 1.78 and 0.98 (d, J = 7.3 Hz, 2H), 0.93 –0.91 (m, 5H), 0.88 –0.79 (m, 7H); ^{13}C NMR (101 MHz, Chloroform-*d*) δ 146.0, 140.0, 135.7, 135.6, 135.3, 128.7, 128.0, 127.6, 126.8, 125.4, 120.7, 120.6, 120.5, 119.7, 119.4, 118.6, 118.2, 110.32, 110.29, 101.3, 53.5, 49.1, 35.2, 29.5, 29.4, 29.1, 21.9, 21.8, 21.7, 21.1, 20.3, 19.6, 19.4. HRMS (ESI): Calcd for [$C_{23}H_{29}N$+H]+: 320.2378, found: 320.2378.

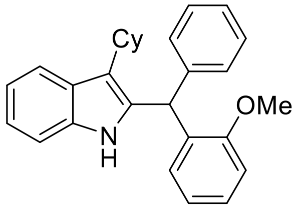

3-Cyclohexyl-2-((2-methoxyphenyl)(phenyl)methyl)-1*H*-indole (**3bo**): According to the **General Procedure A**, 0.25 M TsOH was employed in place of 0.25 M HCl, **3bo** (19.8 mg, 50%) was prepared as a white solid; m.p. 146-148 ºC; ^{1}H NMR (400 MHz, Chloroform-*d*) δ 7.75 (d, J = 7.4 Hz, 1H), 7.64 (s, 1H), 7.27 – 7.15 (m, 5H), 7.10 – 6.96 (m, 5H), 6.88 (dd, J = 7.7, 5.2 Hz, 2H), 6.12 (s, 1H), 3.70 (s, 3H), 2.76 (tt, J = 12.3, 2.9 Hz, 1H), 2.03 – 1.84 (m, 2H), 1.82 – 1.75 (m, 2H), 1.73 – 1.67 (m, 2H), 1.33 – 1.21 (m, 4H); ^{13}C NMR (101 MHz, Chloroform-*d*) δ 157.0, 142.7, 135.6, 134.3, 131.3, 130.1, 128.6, 128.4, 128.2, 127.7, 126.4, 120.8, 120.7, 120.3, 118.6, 118.1, 111.2, 110.9, 55.7, 42.4, 36.6, 32.93, 32.88, 27.42, 27.40, 26.5. HRMS (ESI): Calcd for [$C_{28}H_{29}NO$+H]+: 396.2327, found: 396.2327.

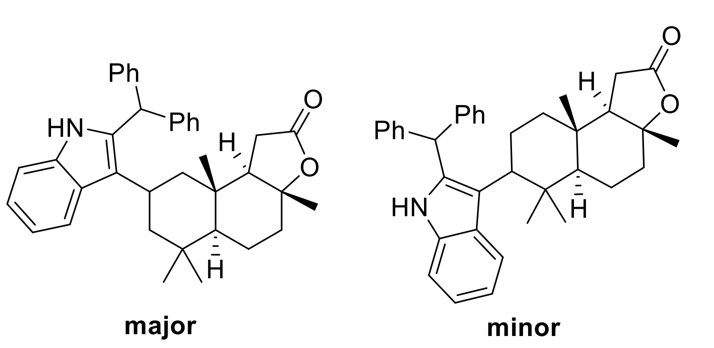

(3a*R*,5a*S*,9a*S*,9b*R*)-8-(2-Benzhydryl-1*H*-indol-3-yl)-3a,6,6,9a-

tetramethyldecahydronaphtho[2,1-b]furan-2(1*H*)-one (**3bp, major**); (3a*R*,5a*S*,9a*S*,9b*R*)-7-(2-Benzhydryl-1*H*-indol-3-yl)-3a,6,6,9a-tetramethyldecahydronaphtho[2,1-b]furan-2(1*H*)-one (**3bp, minor**): According to the **General Procedure C**, **3bp** (25.0 mg, 47%) was prepared as a white solid; m.p. 101-102 ºC; ^{1}H NMR (400 MHz, Chloroform-*d*) δ 7.69 (d, *J* = 7.6 Hz, 1H), 7.56 and 7.48 (s, 1H), 7.38 – 7.33 (m, 2H), 7.32 – 7.27 (m, 3H), 7.22 – 7.19 (m, 2H), 7.18 – 7.14 (m, 3H), 7.10 – 7.00 (m, 3H), 5.85 and 5.77 (s, 1H), 3.27 – 2.96 (m, 1H), 2.58 – 1.82 (m, 8H), 1.81 – 1.63 (m, 2H), 1.48 – 1.35 (m, 3H), 1.24 – 1.13 (m, 2H), 1.08 – 0.94 and 0.90 – 0.89 and 0.72 (m, 9H); ^{13}C NMR (101 MHz, Chloroform-*d*) δ 176.9, 176.8, 142.5, 141.95, 141.89, 141.6, 136.7, 135.6, 135.4, 135.1, 129.4, 129.32, 129.29, 129.27, 129.08, 129.06, 129.01, 128.97, 128.84, 128.78, 128.7, 128.62, 128.58, 128.55, 128.4, 128.2, 127.5, 127.3, 127.2, 127.10, 127.06, 126.9, 126.8, 122.3, 121.1, 120.8, 120.1, 119.12, 119.07, 115.7, 111.2, 86.33, 86.28, 59.7, 59.2, 59.1, 56.5, 51.3, 49.3, 49.2, 47.8, 45.0, 40.2, 39.6, 38.7, 38.6, 38.3, 37.3, 36.7, 36.4, 35.4, 34.0, 33.2, 30.2, 29.7, 28.8, 28.6, 27.2, 27.0, 24.2, 24.0, 22.3, 21.7, 21.6, 21.0, 20.6, 20.2, 15.7, 15.2. HRMS (ESI): Calcd for $[C_{37}H_{41}NO_2+Na]^+$: 554.3035, found: 554.3041.

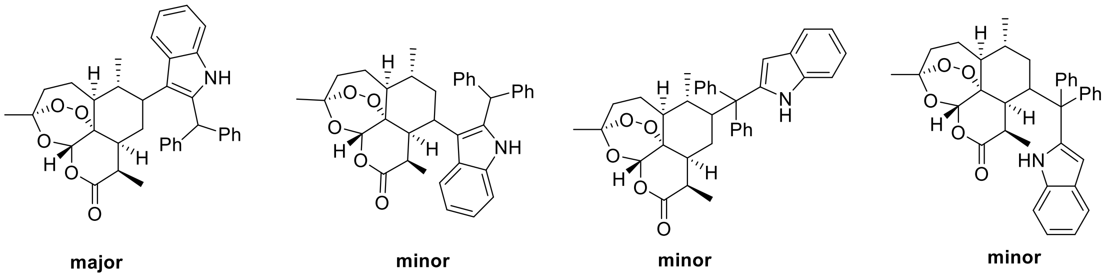

(3*R*,5a*S*,6*R*,8a*S*,9*R*,12*S*,12a*R*)-7-(2-Benzhydryl-1*H*-indol-3-yl)-3,6,9-trimethyloctahydro-12*H*-3,12-epoxy[1,2]dioxepino[4,3-i]isochromen-10(3*H*)-one (**3bq, major**); (3*R*,5a*S*,6*R*,8a*S*,9*R*,12*S*,12a*R*)-8-(2-Benzhydryl-1*H*-indol-3-yl)-3,6,9-trimethyloctahydro-12*H*-3,12-epoxy[1,2]dioxepino[4,3-i]isochromen-10(3*H*)-one (**3bq, minor**); (3R,5aS,6R,8aS,9R,12S,12aR)-7-((1H-indol-2-yl)diphenylmethyl)-3,6,9-trimethyloctahydro-12H-3,12-epoxy[1,2]dioxepino[4,3-i]isochromen-10(3H)-one (**3bq, minor**); (3R,5aS,6R,8aS,9R,12S,12aR)-8-((1H-indol-2-yl)diphenylmethyl)-3,6,9-trimethyloctahydro-12H-3,12-epoxy[1,2]dioxepino[4,3-i]isochromen-10(3H)-

one (**3bq, minor**): According to the **General Procedure C**, but with a reaction time of 6 h instead of 3 h, **3bq** (25.3 mg, 45%) was prepared as a white solid; m.p. 112-114 ºC; ^{1}H NMR (400 MHz, Chloroform-*d*) δ 7.61 – 7.52 (m, 1H), 7.39 – 7.28 (m, 8H), 7.27 (s, 1H), 7.23 – 7.17 (m, 4H), 7.15 – 7.08 (m, 2H), 6.34 and 5.86 and 5.87 (s, 1H), 3.65 – 3.64 and 3.42 – 3.38 (m, 1H), 2.50 – 2.38 (m, 1H), 2.10 – 1.98 (m, 2H), 1.92 – 1.74 (m, 3H), 1.45 (d, *J* = 2.6 Hz, 2H), 1.38 – 1.37 and 1.34 – 1.33 and 1.29 – 1.28 and 1.22 and 1.20 (m, 9H), 1.00 (dd, *J* = 6.0, 2.6 Hz, 2H); ^{13}C NMR (101 MHz, Chloroform-*d*) δ 184.8, 182.9, 172.2, 162.4, 158.1, 153.6, 141.2, 140.6, 135.6, 134.8, 129.2, 129.0, 128.9, 128.8, 128.2, 127.9, 127.7, 126.4, 123.7, 123.1, 121.3, 111.2, 105.4, 93.8, 79.5, 77.4, 77.3, 77.1, 76.7, 50.1, 48.6, 45.0, 41.8, 37.6, 35.9, 33.6, 32.9, 31.5, 30.2, 29.7, 25.2, 24.9, 23.4, 19.9, 12.6. HRMS (ESI): Calcd for [$C_{36}H_{37}NO_5$+H]+: 564.2750, found: 564.2750.

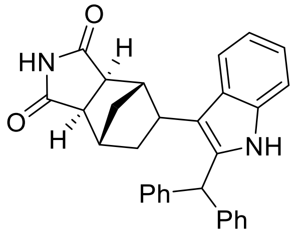

(3a*S*,4*S*,7*R*,7a*R*)-5-(2-Benzhydryl-1*H*-indol-3-yl)hexahydro-1*H*-4,7-methanoisoindole-1,3(2*H*)-dione (**3br**): According to the **General Procedure C**, but with a reaction time of 6 h instead of 3 h, **3br** (18.2 mg, 41%) was prepared as a white solid; m.p. 131-133 ºC; ^{1}H NMR (400 MHz, Chloroform-*d*) δ 7.91 (s, 1H), 7.66 (d, *J* = 7.8 Hz, 1H), 7.59 (s, 1H), 7.38 – 7.29 (m, 6H), 7.23 (d, *J* = 7.5 Hz, 1H), 7.16 – 7.06 (m, 6H), 5.79 (s, 1H), 3.06 – 2.94 (m, 1H), 2.85 (d, *J* = 3.7 Hz, 1H), 2.77 (s, 1H), 2.71 (d, *J* = 7.1 Hz, 1H), 2.61 (d, *J* = 7.1 Hz, 1H), 2.18 (dt, *J* = 13.0, 4.9 Hz, 1H), 2.00 (dt, *J* = 11.5, 1.5 Hz, 1H), 1.80 – 1.70 (m, 1H), 0.99 – 0.80 (m, 1H); ^{13}C NMR (101 MHz, Chloroform-*d*) δ 178.7, 178.0, 141.7, 141.6, 135.9, 135.4, 129.01, 128.98, 128.9, 127.3, 127.2, 126.8, 121.2, 120.1, 119.6, 114.7, 111.2, 77.4, 77.2, 77.0, 76.7, 51.1, 49.9, 49.0, 45.5, 40.1, 39.3, 36.0, 32.6. HRMS (ESI): Calcd for [$C_{30}H_{26}N_2O_2$+H]$^+$: 447.2073, found: 447.2078.

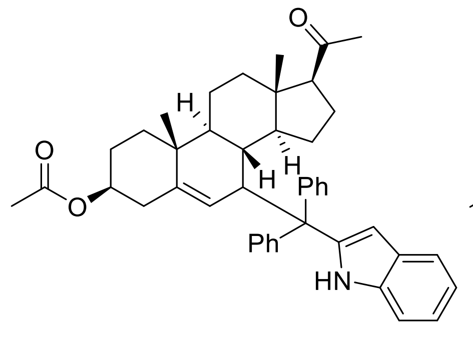

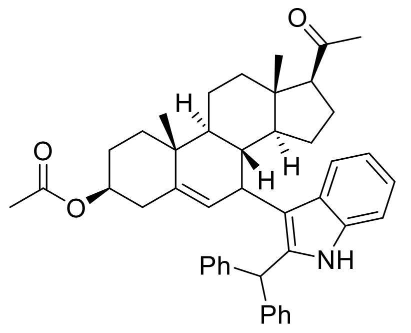

(3S,8S,9S,10R,13S,14S,17S)-7-((1H-indol-2-yl)diphenylmethyl)-17-acetyl-10,13-dimethyl-2,3,4,7,8,9,10,11,12,13,14,15,16,17-tetradecahydro-1H-cyclopenta[a]phenanthren-3-yl acetate (**3bs, major**); (3*S*,8*S*,9*S*,10*R*,13*S*,14*S*,17*S*)-17-Acetyl-7-(2-benzhydryl-1*H*-indol-3-yl)-10,13-dimethyl-2,3,4,7,8,9,10,11,12,13,14,15,16,17-tetradecahydro-1*H*-cyclopenta[a]phenanthren-3-yl acetate (**3bs, minor**): According to the **General Procedure C**, 0.25 M HCl was employed in place of 0.25 M TsOH, with a reaction time of 6 h instead of 3 h, **3bs** (37.1 mg, 58%) was prepared as a yellow solid; m.p. 133-134 ºC; ^{1}H NMR (400 MHz, Chloroform-*d*) δ 7.70 and 7.56 (d, *J* = 8.1 Hz, 1H), 7.64 and 7.61 (s, 1H), 7.40 – 7.28 (m, 5H), 7.24 – 7.17 (m, 3H), 7.15 – 7.06 (m, 4H), 7.05 –7.03 and 6.97 –6.95 (m, 1H) 6.10 and 5.86 (s, 1H), 5.55 and 4.80 (d, *J* = 4.6 Hz, 1H), 4.80 – 4.69 and 4.66 – 4.56 (m, 1H), 4.00 – 3.92 and 3.53 – 3.45 (m, 1H), 2.56 and 2.25 (d, *J* = 11.8 Hz, 1H), 2.48 –2.42 and 2.34 –2.30 (m, 1H), 2.19 – 2.08 (m, 1H), 2.05 and 2.04 (s, 4H), 1.99 and 1.94 (s, 3H), 1.94 – 1.81 (m, 3H), 1.81 – 1.58 (m, 2H), 1.55 – 1.46 (m, 2H), 1.45 – 1.09 (m, 6H), 1.09 and 1.07 (s, 5H), 0.90 – 0.81 (m, 1H), 0.67 and 0.52 (s, 3H); ^{13}C NMR (101 MHz, Chloroform-*d*) δ ^{13}C NMR (101 MHz, $CDCl_3$) δ 209.9, 209.8, 170.4, 143.5, 142.8, 142.7, 141.7, 138.03, 137.97, 136.9, 136.7, 135.6, 135.3, 130.0, 129.6, 129.2, 129.1, 128.8, 128.7, 128.53, 128.49, 127.3, 127.1, 126.8, 126.7, 125.9, 121.6, 121.4, 121.0, 119.2, 112.70, 112.67, 110.9, 110.6, 73.7, 62.9, 62.7, 52.5, 51.7, 48.9, 48.8, 46.3, 44.7, 44.2, 44.0, 38.3, 37.9, 36.73, 36.67, 36.6, 36.51, 36.46, 35.9, 34.8, 31.7, 31.6, 27.83, 27.78, 25.5, 25.2, 23.0, 22.8, 21.45, 21.41, 21.3, 20.6, 19.2, 18.9, 13.5, 13.0; HRMS (ESI): Calcd for $[C_{44}H_{49}NO_3+H]^+$: 640.3791, found: 640.3775.

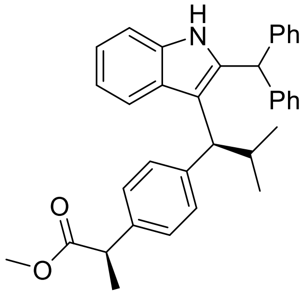

Methyl (*R*)-2-(4-((*S*)-1-(2-benzhydryl-1*H*-indol-3-yl)-2-methylpropyl)phenyl)propanoate (**3bt**): According to the **General Procedure C**, **3bt** (20.0 mg, 40%) was prepared as a white solid; m.p. 134-136 ºC; ^{1}H NMR (400 MHz, Chloroform-*d*) δ 7.90 – 7.79 (m, 1H), 7.46 (s, 1H), 7.30 – 7.19 (m, 6H), 7.15 (dd, *J* = 10.1, 7.7 Hz, 3H), 7.10 – 6.97 (m, 8H), 5.88 (s, 1H), 3.66 (d, *J* = 11.9 Hz, 1H), 3.620 and 3.616 (s, 3H), 2.87 – 2.73 (m, 1H), 1.41 (d, *J* = 7.2 Hz, 3H), 0.90 (d, *J* = 6.4 Hz, 3H), 0.87 – 0.82 (m, 1H), 0.79 (d, *J* = 6.5 Hz, 3H); ^{13}C NMR (101 MHz, Chloroform-*d*) δ 175.3, 143.7, 142.2, 141.9, 137.5, 137.4, 135.8, 135.6, 129.1, 128.7, 128.6, 128.5, 127.6, 127.1, 126.9, 126.8, 121.2, 120.5, 119.4, 116.0, 110.9, 52.0, 51.4, 48.3, 45.0, 44.9, 30.7, 22.5, 22.1, 18.7, 18.6. HRMS (ESI): Calcd for $[C_{35}H_{35}NO_2+H]^+$: 502.2746, found: 502.2769.

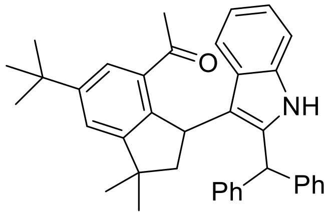

1-(3-(2-Benzhydryl-1*H*-indol-3-yl)-6-(tert-butyl)-1,1-dimethyl-2,3-dihydro-1*H*-inden-4-yl)-ethan-1-one (**3bu**): According to the **General Procedure C**, **3bu** (24.2 mg, 46%) was prepared as a white solid; m.p. 121-123 ºC; ^{1}H NMR (400 MHz, Chloroform-*d*) δ 7.45 – 7.38 (m, 5H), 7.33 – 7.27 (m, 4H), 7.24 (s, 1H), 7.19 – 7.11 (m, 4H), 6.95 (t, *J* = 7.6 Hz, 1H), 6.73 (t, *J* = 7.5 Hz, 1H), 6.38 (d, *J* = 8.0 Hz, 1H), 6.05 (s, 1H), 4.81 (t, *J* = 8.8 Hz, 1H), 1.99 – 1.96 (m, 1H), 1.77 (s, 3H), 1.76 – 1.71 (m, 1H), 1.38 (s, 3H), 1.36 (s, 9H), 1.09 (s, 3H); ^{13}C NMR (101 MHz, Chloroform-*d*) δ 203.1, 153.9, 150.2, 143.0,

142.1, 140.8, 137.9, 136.8, 135.5, 129.6, 129.1, 128.9, 128.4, 127.0, 126.60, 126.56, 122.2, 121.2, 120.5, 119.6, 118.8, 114.8, 110.8, 77.4, 77.0, 76.7, 49.3, 48.3, 42.9, 38.6, 34.8, 31.6, 30.2, 28.8, 28.6; HRMS (ESI): Calcd for [$C_{38}H_{39}NO$+H]+: 526.3110, found: 526.3137.

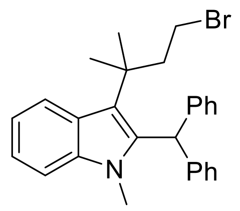

2-Benzhydryl-3-(4-bromo-2-methylbutan-2-yl)-1-methyl-1*H*-indole (**4**), **4** (31.2 mg, 70%) was prepared as a yellowish oil; ^{1}H NMR (400 MHz, Chloroform-*d*) δ 7.93 (d, *J* = 8.2 Hz, 1H), 7.31 (t, *J* = 7.5 Hz, 5H), 7.25 – 7.21 (m, 3H), 7.13 (d, *J* = 7.4 Hz, 5H), 6.55 (s, 1H), 3.11 (s, 3H), 2.89 – 2.78 (m, 2H), 2.53 – 2.46 (m, 2H), 1.62 (s, 6H); ^{13}C NMR (101 MHz, Chloroform-*d*) δ 141.1, 137.7, 136.9, 129.0, 128.7, 126.9, 126.7, 121.9, 121.1, 119.1, 117.3, 109.1, 49.7, 47.1, 40.2, 32.7, 31.3, 31.0. HRMS (ESI): Calcd for [$C_{27}H_{28}NBr$+H]+: 446.1483, found: 446.1483.

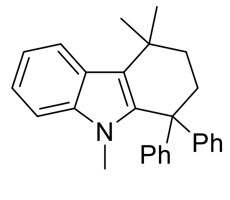

4,4,9-Trimethyl-1,1-diphenyl-2,3,4,9-tetrahydro-1*H*-carbazole (**5**): **5** (21.9 mg, 60%) was prepared as a yellowish oil; ^{1}H NMR (400 MHz, Chloroform-*d*) δ 7.84 (d, *J* = 7.9 Hz, 1H), 7.32 – 7.27 (m, 4H), 7.26 – 7.20 (m, 8H), 7.16 – 7.10 (m, 1H), 2.84 (s, 3H), 2.62 – 2.54 (m, 2H), 1.50 (s, 6H), 1.46 – 1.38 (m, 2H); ^{13}C NMR (101 MHz, Chloroform-*d*) δ 145.2, 138.6, 137.7, 129.3, 128.2, 126.5, 125.5, 120.9, 120.7, 120.2, 118.6, 109.1, 51.9, 41.8, 37.1, 32.4, 31.6, 29.8. HRMS (ESI): Calcd for [$C_{27}H_{27}N$+H]$^{+}$: 366.2222, found: 366.2235.

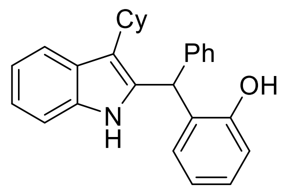

2-((3-Cyclohexyl-1*H*-indol-2-yl)(phenyl)methyl)phenol (**6**): **6** (21.7 mg, 57%) was prepared as a yellow oil; ^{1}H NMR (400 MHz, Chloroform-*d*) δ 7.81 – 7.72 (m, 2H), 7.33 – 7.26 (m, 3H), 7.23 – 7.14 (m, 4H), 7.12 – 7.04 (m, 2H), 6.95 (dd, *J* = 7.7, 1.8 Hz, 1H), 6.88 (td, *J* = 7.5, 1.2 Hz, 1H), 6.81 (dd, *J* = 8.0, 1.2 Hz, 1H), 5.97 (s, 1H), 4.93 (s, 1H), 2.78 (tt, *J* = 12.3, 3.6 Hz, 1H), 1.96 – 1.89 (m, 2H), 1.82 – 1.72 (m, 4H), 1.36 – 1.24 (m, 4H); ^{13}C NMR (101 MHz, Chloroform-*d*) δ 153.4, 141.3, 135.8, 133.0, 130.4, 129.1, 128.8, 128.6, 128.5, 127.5, 127.0, 121.3, 121.2, 120.5, 118.9, 118.8, 116.6, 111.0, 43.5, 36.6, 33.0, 32.9, 27.3, 26.3. HRMS (ESI): Calcd for [$C_{27}H_{27}NO$+H]+: 382.2171, found: 382.2171.

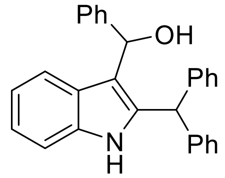

(2-Benzhydryl-1*H*-indol-3-yl)(phenyl)methanol (**7**): **7** (25.3 mg, 65%) was prepared as a yellowish solid; m.p. 68-70 ºC; ^{1}H NMR (400 MHz, Chloroform-*d*) δ 7.69 (s, 1H), 7.44 – 7.40 (m, 1H), 7.38 – 7.35 (m, 2H), 7.34 – 7.26 (m, 6H), 7.24 – 7.19 (m, 3H), 7.17 – 7.08 (m, 6H), 7.02 –6.98 (m, 1H), 6.21 and 6.20 (s, 1H), 5.90 (s, 1H); ^{13}C NMR (101 MHz, Chloroform-*d*) δ 143.4, 142.0, 141.9, 137.4, 135.5, 129.03, 128.97, 128.9, 128.2, 127.2, 126.9, 126.1, 121.9, 120.1, 115.1, 111.1, 68.9, 48.4. HRMS (ESI): Calcd for [$C_{28}H_{23}NO$+H]+: 390.1858, found: 390.1858.

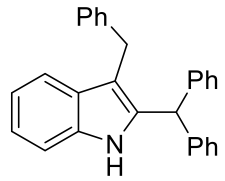

2-Benzhydryl-3-benzyl-1*H*-indole (**8**): **8** (23.1 mg, 62%) was prepared as a colorless oil; ^{1}H NMR (400 MHz, Chloroform-*d*) δ 7.58 (s, 1H), 7.42 (d, *J* = 7.8 Hz, 1H), 7.32 –

7.23 (m, 7H), 7.23 – 7.16 (m, 3H), 7.15 – 7.12 (m, 3H), 7.11 – 7.08 (m, 4H), 7.05 – 7.01 (m, 1H), 5.74 (s, 1H), 4.03 (s, 2H); ^{13}C NMR (101 MHz, Chloroform-*d*) δ 142.0, 141.2, 136.3, 135.4, 129.0, 128.7, 128.4, 128.2, 126.9, 125.7, 121.5, 119.5, 119.0, 111.5, 110.7, 48.3, 30.1. HRMS (ESI): Calcd for [$C_{28}H_{23}N$+H]+: 374.1909, found: 374.1909.

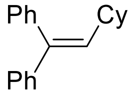

(2-Cyclohexylethene-1,1-diyl)dibenzene (**10**): According to the radical trapping experiment with 1,1-diphenylethylene, **10** (13.1 mg, 25%) was prepared as a colorless oil; ^{1}H NMR (400 MHz, Chloroform-*d*) δ 7.39 – 7.34 (m, 2H), 7.33 – 7.29 (m, 1H), 7.26 – 7.23 (m, 2H), 7.21 – 7.20 (m, 2H), 7.19 – 7.17 (m, 2H), 7.17 – 7.16 (m, 1H), 5.90 (d, *J* = 10.0 Hz, 1H), 2.22 – 2.03 (m, 1H), 1.74 – 1.60 (m, 5H), 1.21 – 1.11 (m, 5H); ^{13}C NMR (101 MHz, Chloroform-*d*) δ 143.0, 140.7, 139.7, 136.1, 129.9, 128.2, 128.1, 127.3, 126.9, 126.8, 38.4, 33.4, 26.1, 25.7.

8. References

[1] T.-Z. Li, S.-J. Liu, Y.-W. Sun, S. Deng, W. Tan, Y. Jiao, Y.-C. Zhang, F. Shi, *Angew. Chem. Int. Ed.* **2021**, *60*, 2355–2363.

[2] Y.-C. Shi, X.-Y. Yan, P. Wu, S. Jiang, R. Xu, W. Tan, F. Shi, *Chin. J. Chem.* **2023,** *41*, 27–36.

[3] S. C. Zhan, J. Sun, Q. Sun, Han, Y. Han, C. G. Yan, *J. Org. Chem.* **2023**, *88*, 9, 5440–5456.

[4] M. Sun, C. Ma, S.-J. Zhou, S.-F. Lou, J. Xiao, Y. Jiao, F. Shi, *Angew. Chem. Int. Ed.* **2019**, *58*, 8703–8708.

[5] Z. Han, H. Zhuang, L. Tang, Y. Zang, W. Guo, H. Huang, J. Sun, *Org. Lett.* **2022,** *24*, 4246-4251.

9. NMR spectra for all compounds

11.

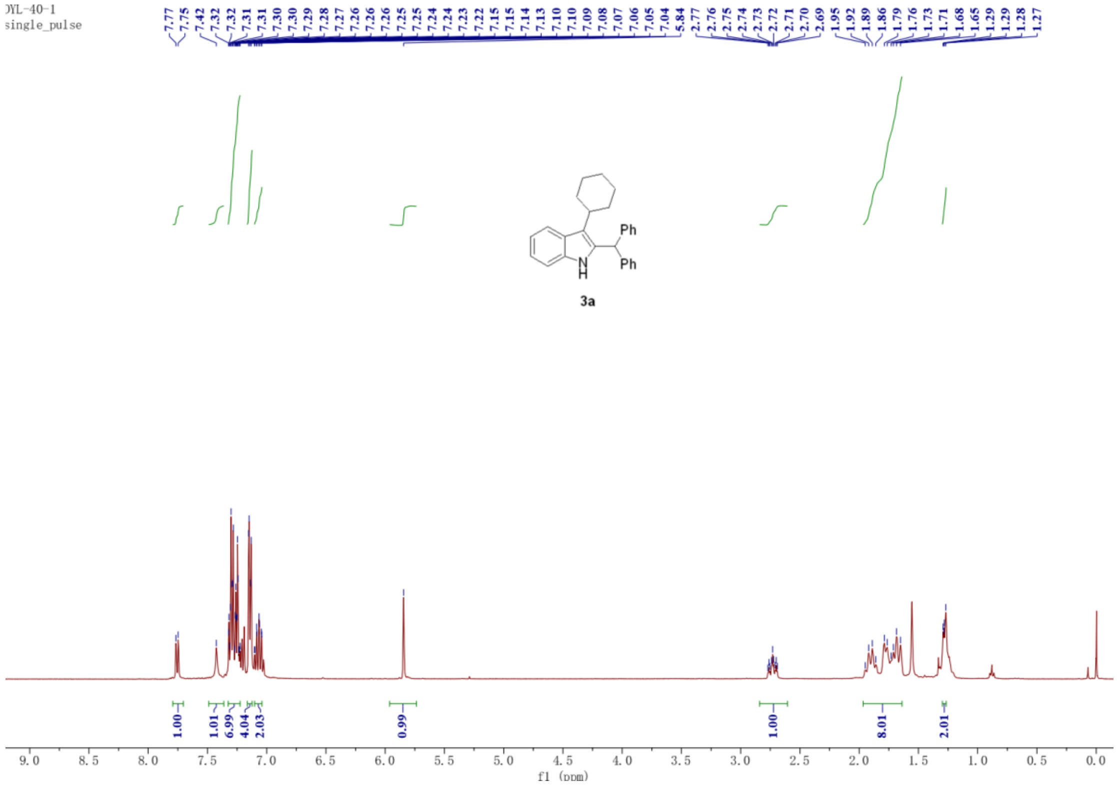

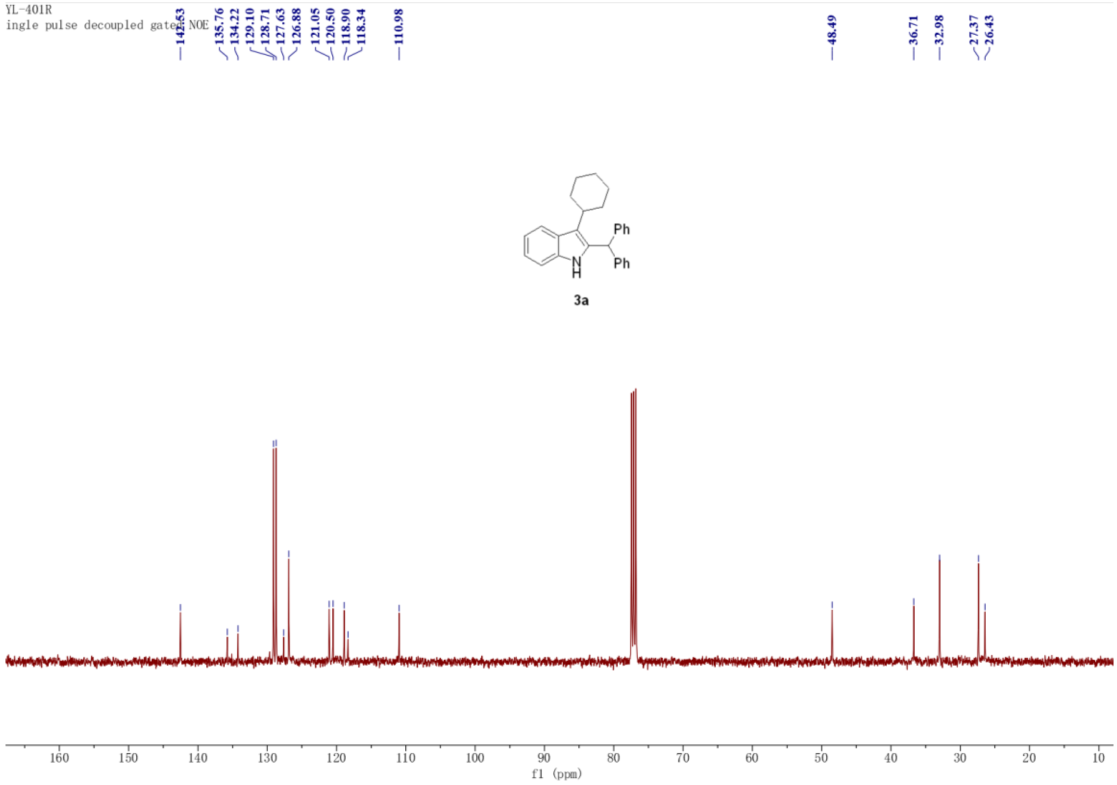

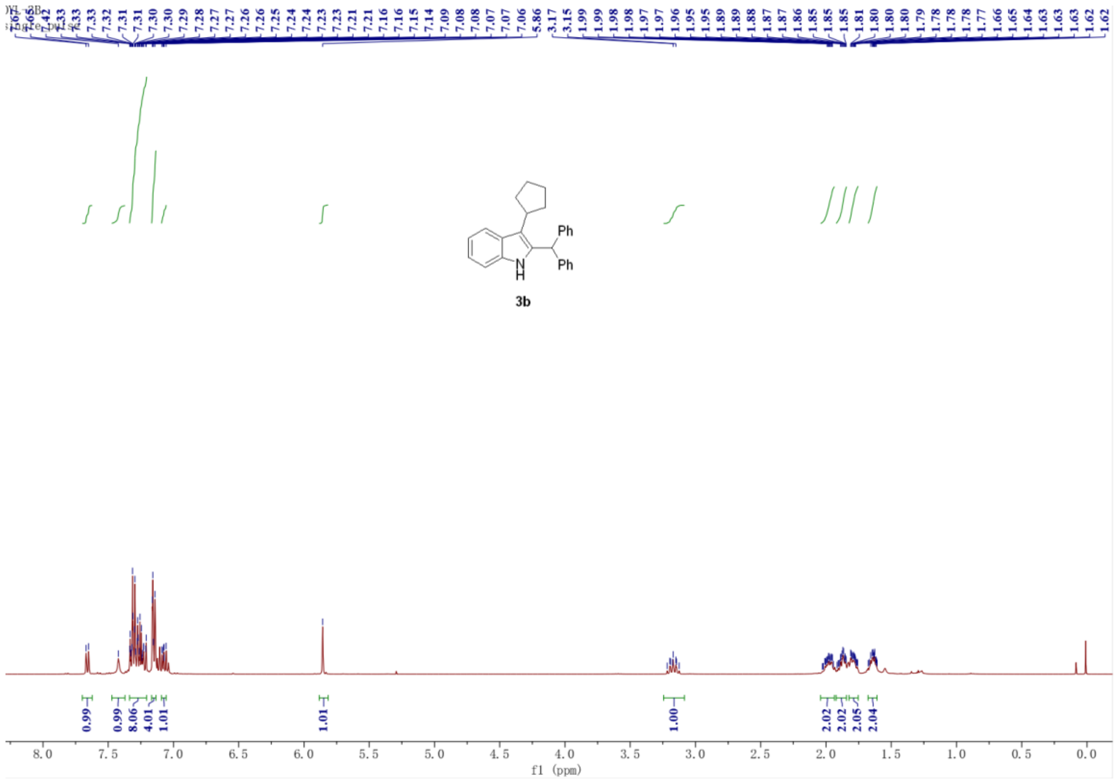
DYL-3B
Ph
Ph
N
H
3b
f1 (ppm)

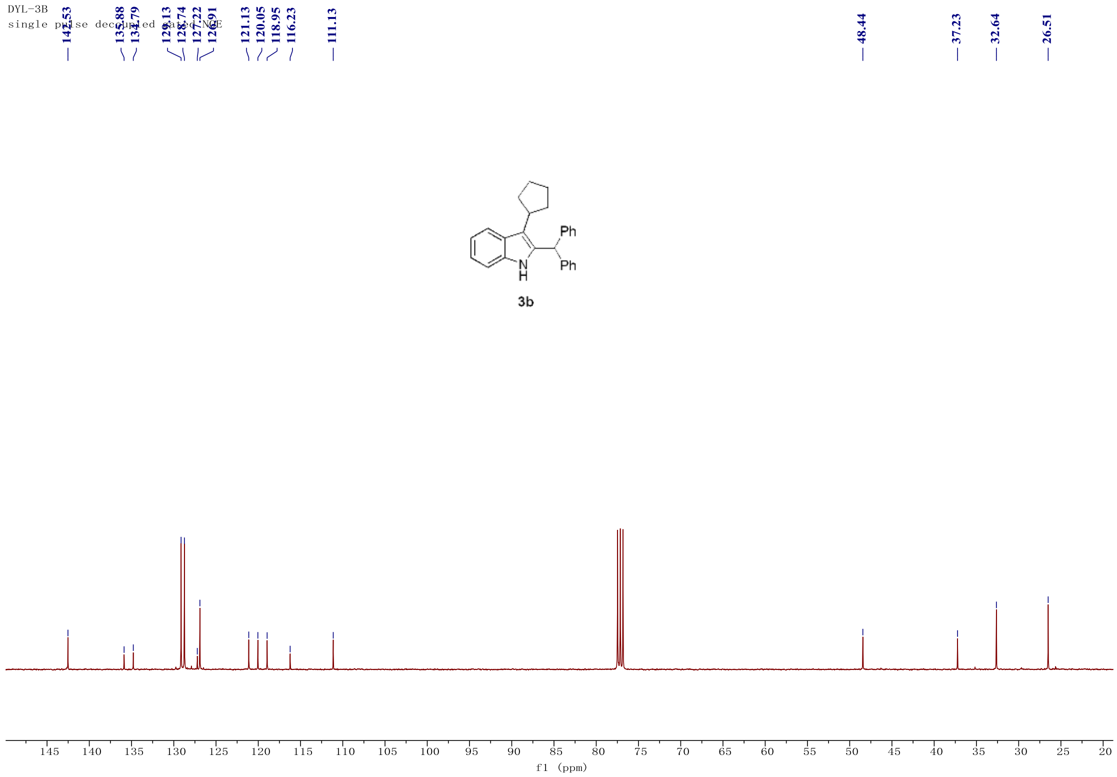
DYL-3B
Ph
Ph
N
H
3b
f1 (ppm)

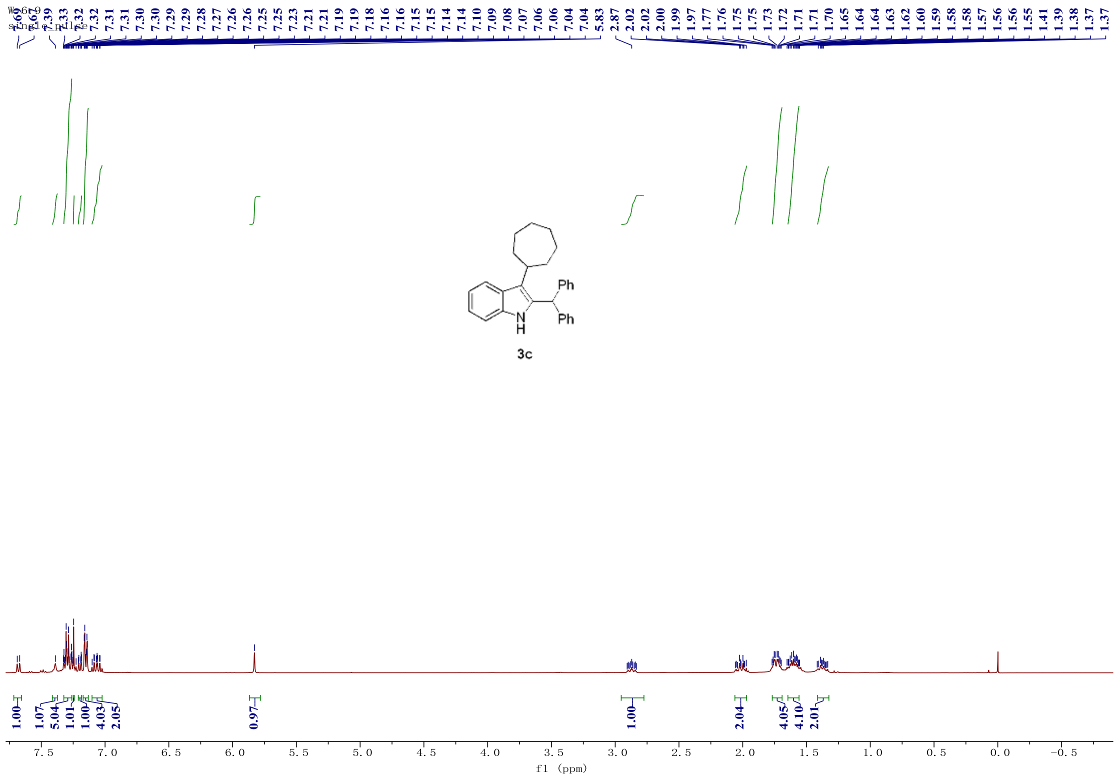

7.33
7.32
7.32
7.31
7.30
7.29
7.28
7.27
7.26
7.25
7.25
7.23
7.21
7.19
7.19
7.18
7.16
7.16
7.15
7.15
7.14
7.14
7.10
7.09
7.08
7.07
7.06
7.06
7.04
7.04
5.83
2.87
2.02
2.02
2.00
1.99
1.97
1.77
1.76
1.75
1.75
1.73
1.72
1.71
1.71
1.70
1.65
1.64
1.64
1.63
1.62
1.60
1.59
1.58
1.58
1.57
1.56
1.56
1.55
1.41
1.39
1.38
1.37
1.37
Ph
Ph
N
H
3c
1.00
1.07
5.04
1.01
1.00
4.03
2.05
0.97
1.00
2.04
4.05
4.10
2.01
f1 (ppm)

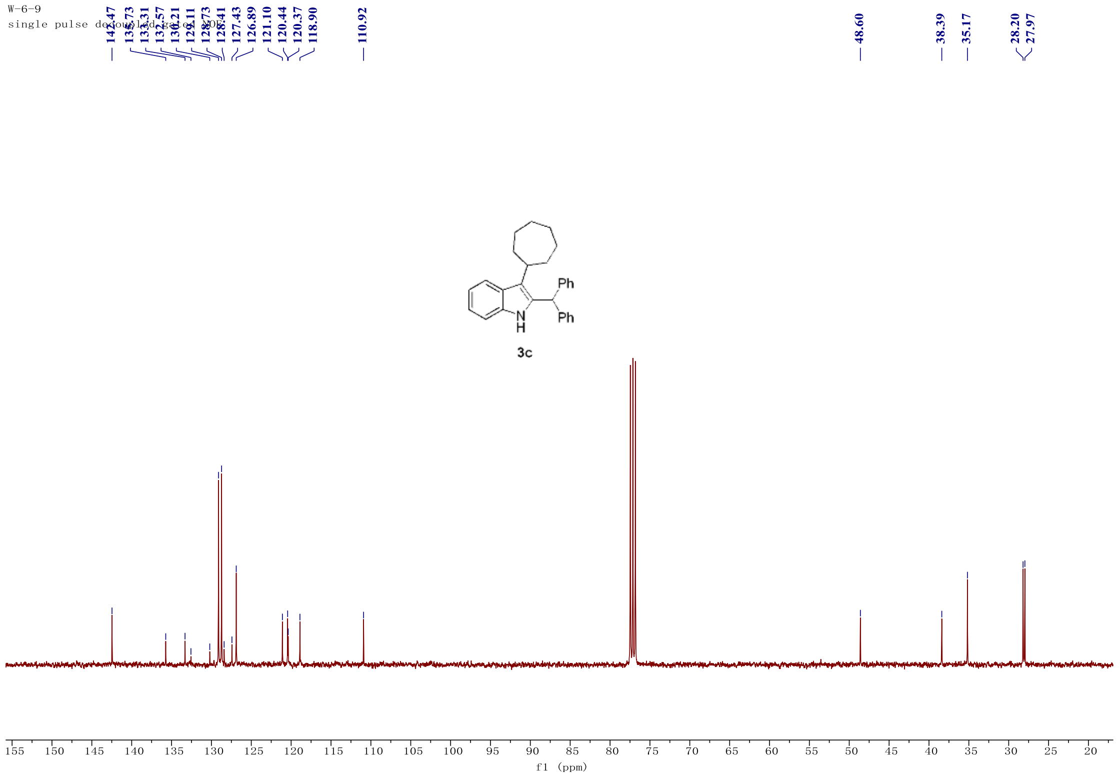

W-6-9
142.47
135.73
133.31
132.57
130.21
129.11
128.73
128.41
127.43
126.89
121.10
120.44
120.37
118.90
110.92
48.60
38.39
35.17
28.20
27.97
Ph
Ph
N
H
3c
f1 (ppm)

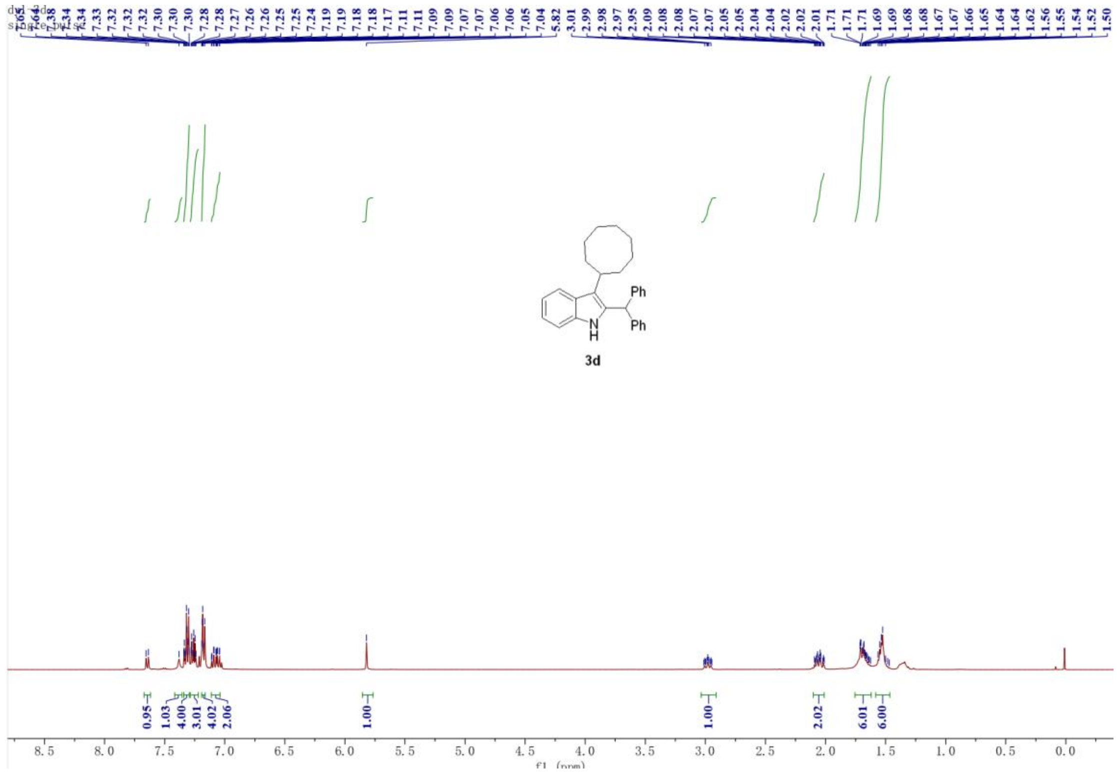

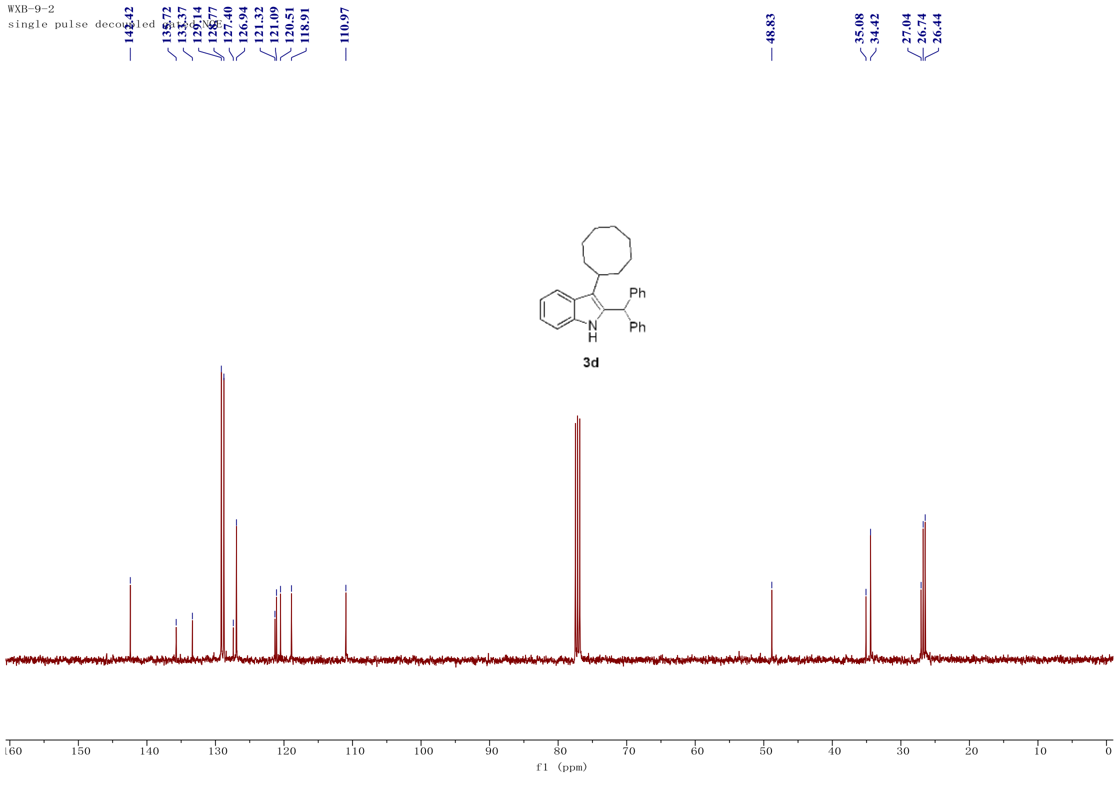

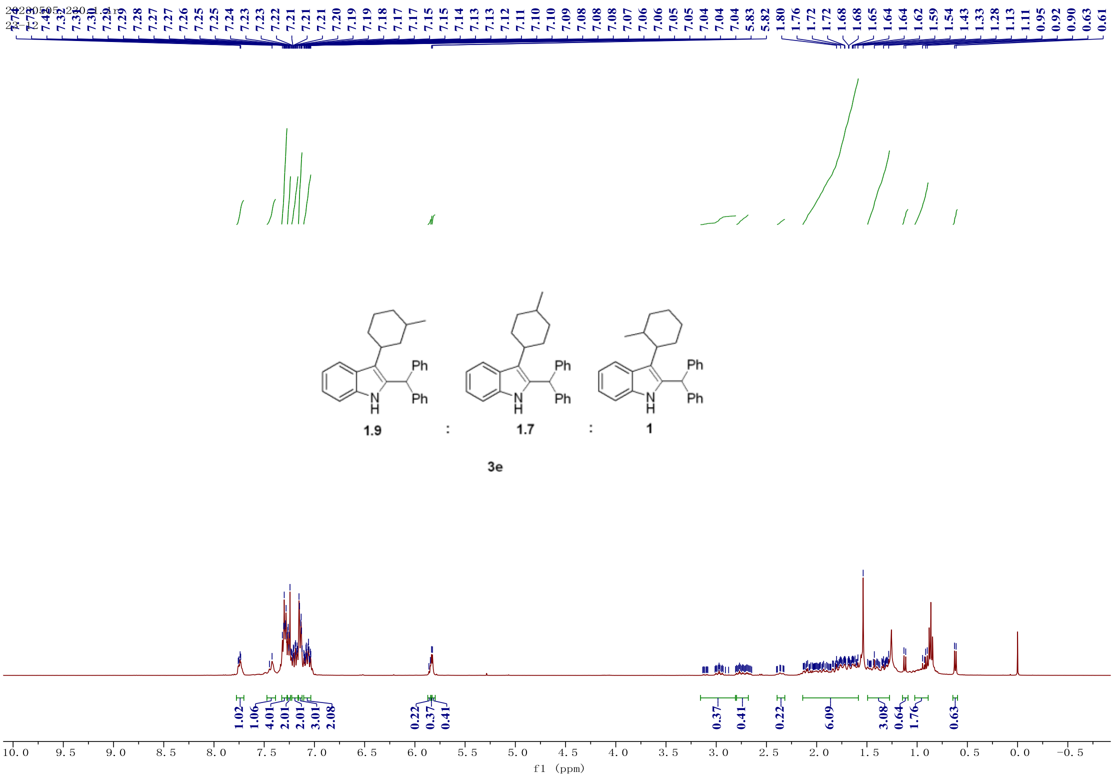
1.9
:
1.7
:
1
3e
f1 (ppm)

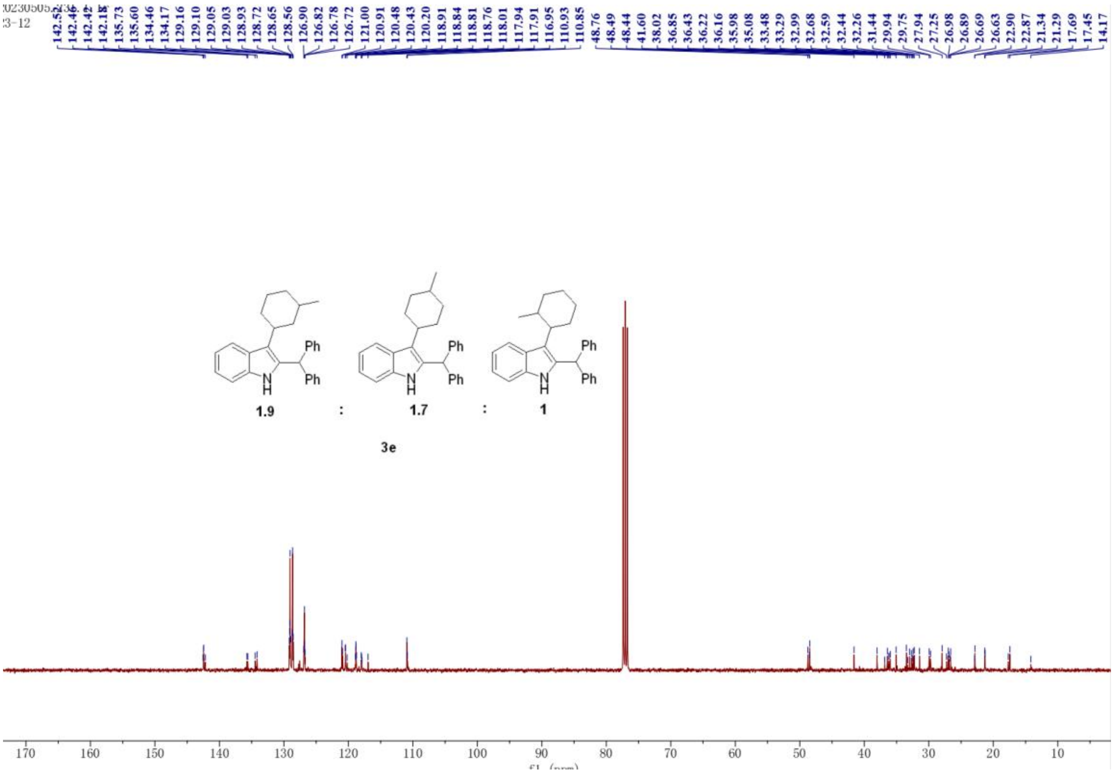
1.9
:
1.7
:
1
3e

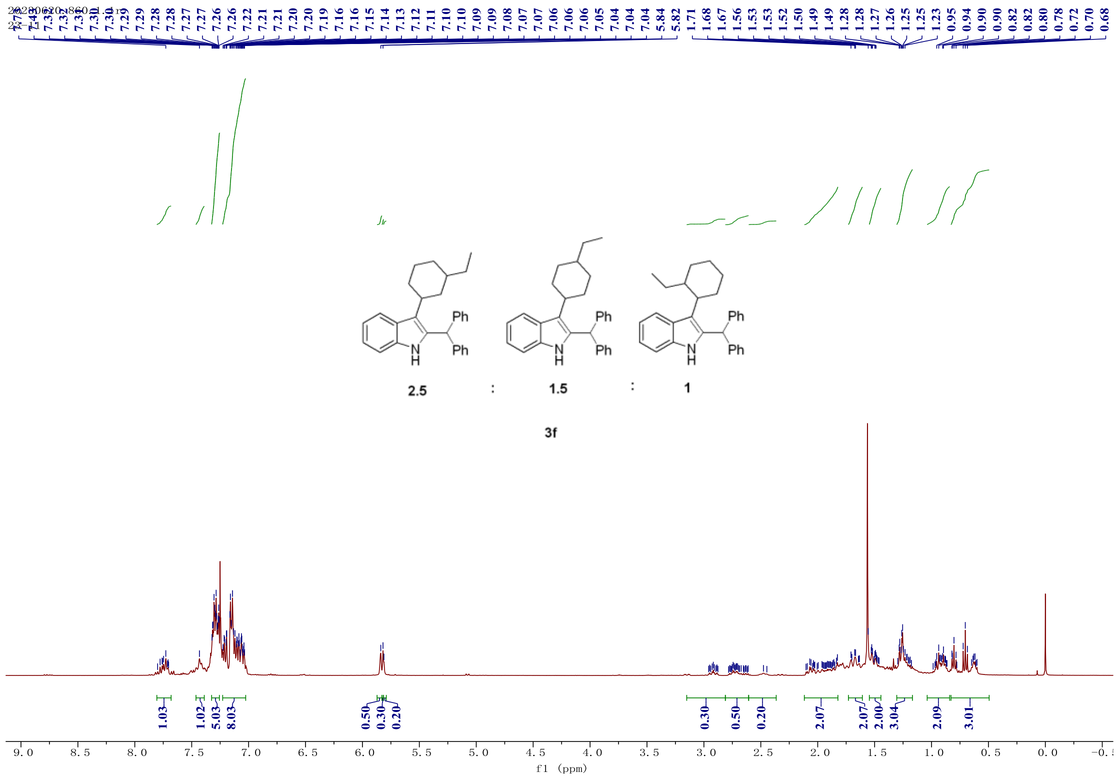
2.5 : 1.5 : 1
3f
f1 (ppm)

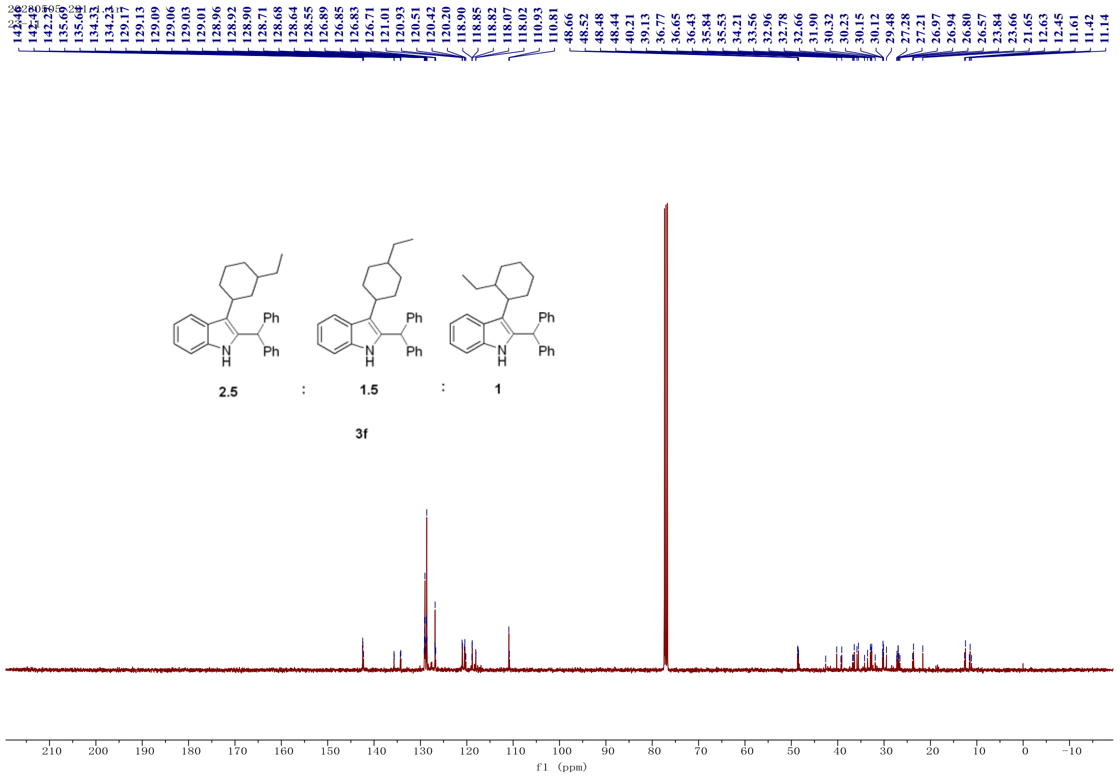
2.5 : 1.5 : 1
3f
f1 (ppm)

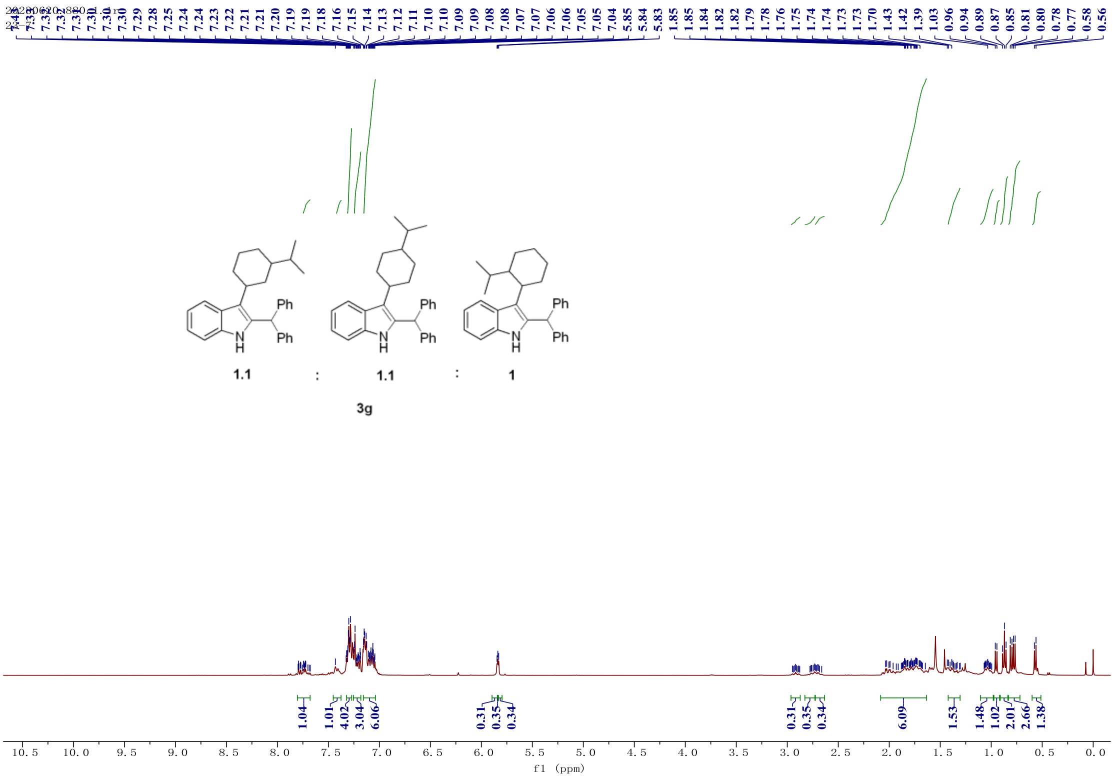
7.33
7.32
7.31
7.30
7.30
7.29
7.28
7.25
7.24
7.24
7.23
7.22
7.21
7.21
7.20
7.19
7.19
7.18
7.16
7.15
7.14
7.13
7.12
7.11
7.10
7.09
7.08
7.08
7.07
7.07
7.06
7.05
7.05
7.04
5.85
5.84
5.83
1.85
1.85
1.84
1.82
1.82
1.79
1.78
1.76
1.75
1.74
1.74
1.73
1.70
1.43
1.42
1.39
1.03
0.96
0.94
0.89
0.87
0.85
0.81
0.80
0.78
0.77
0.58
0.56
Ph
Ph
N
H
1.1
:
1.1
:
1
3g
1.04
1.01
4.02
3.04
6.06
0.31
0.35
0.34
0.31
0.35
0.34
6.09
1.53
1.48
1.02
2.01
2.66
1.38
f1 (ppm)

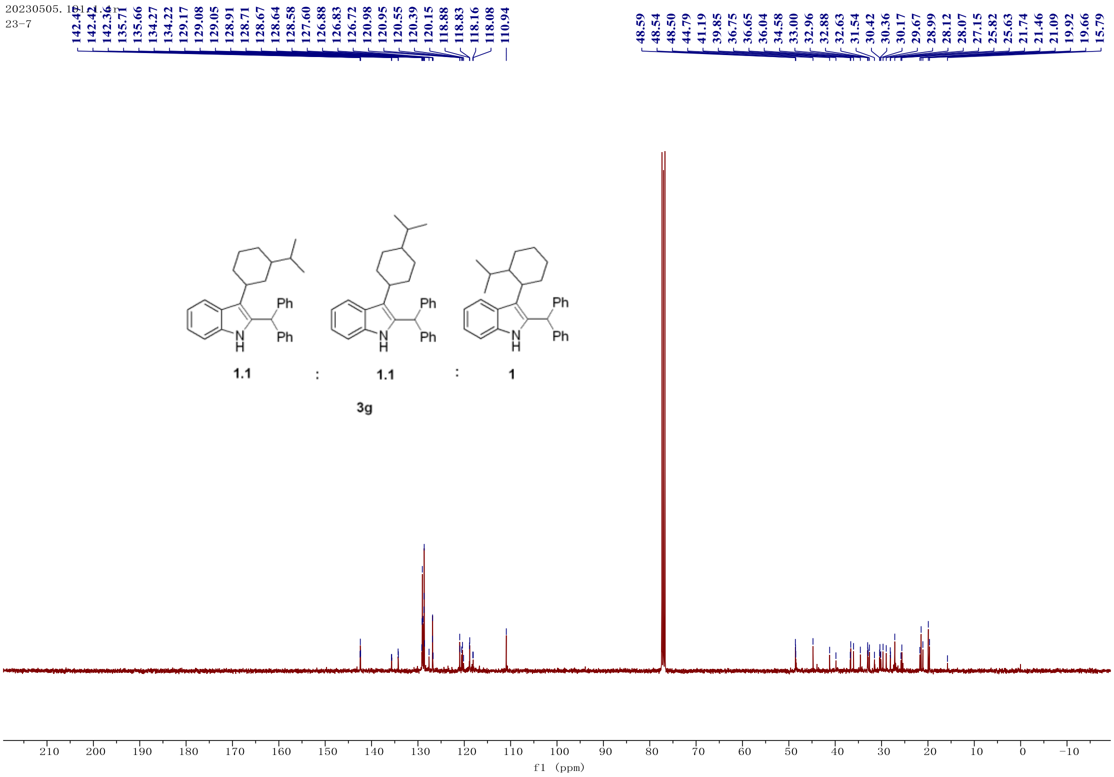
20230505.
23-7
142.42
142.36
135.71
135.66
134.27
134.22
129.17
129.08
129.05
128.91
128.71
128.67
128.64
128.58
127.60
126.88
126.83
126.72
120.98
120.95
120.55
120.39
120.15
118.88
118.83
118.16
118.08
110.94
48.59
48.54
48.50
44.79
41.19
39.85
36.75
36.65
36.04
34.58
33.00
32.96
32.88
32.63
31.54
30.42
30.36
30.17
29.67
28.99
28.12
28.07
27.15
25.82
25.63
21.74
21.46
21.09
19.92
19.66
15.79
Ph
Ph
N
H
1.1
:
1.1
:
1
3g
f1 (ppm)

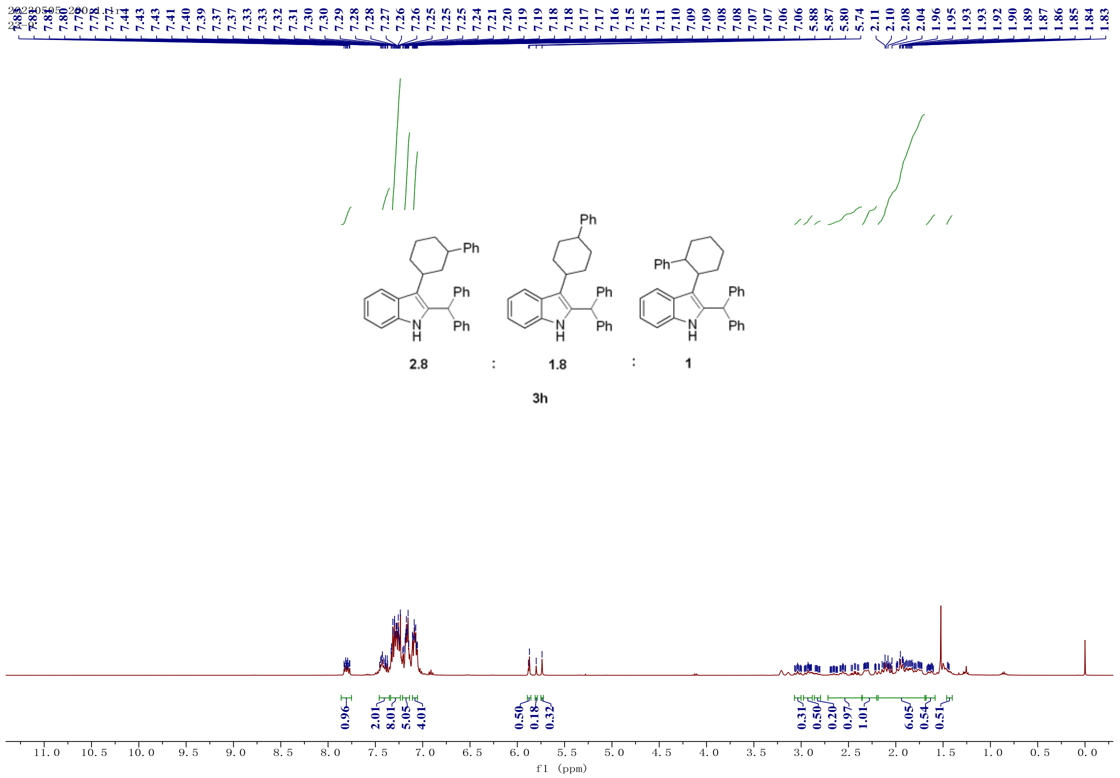

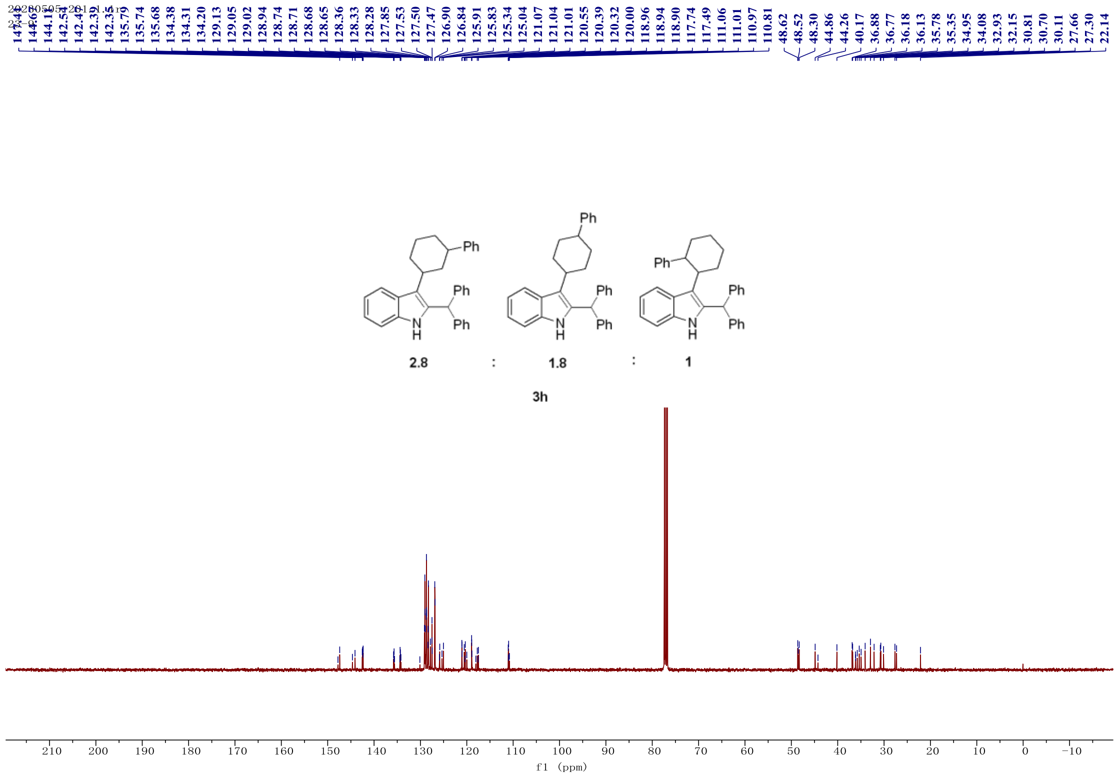

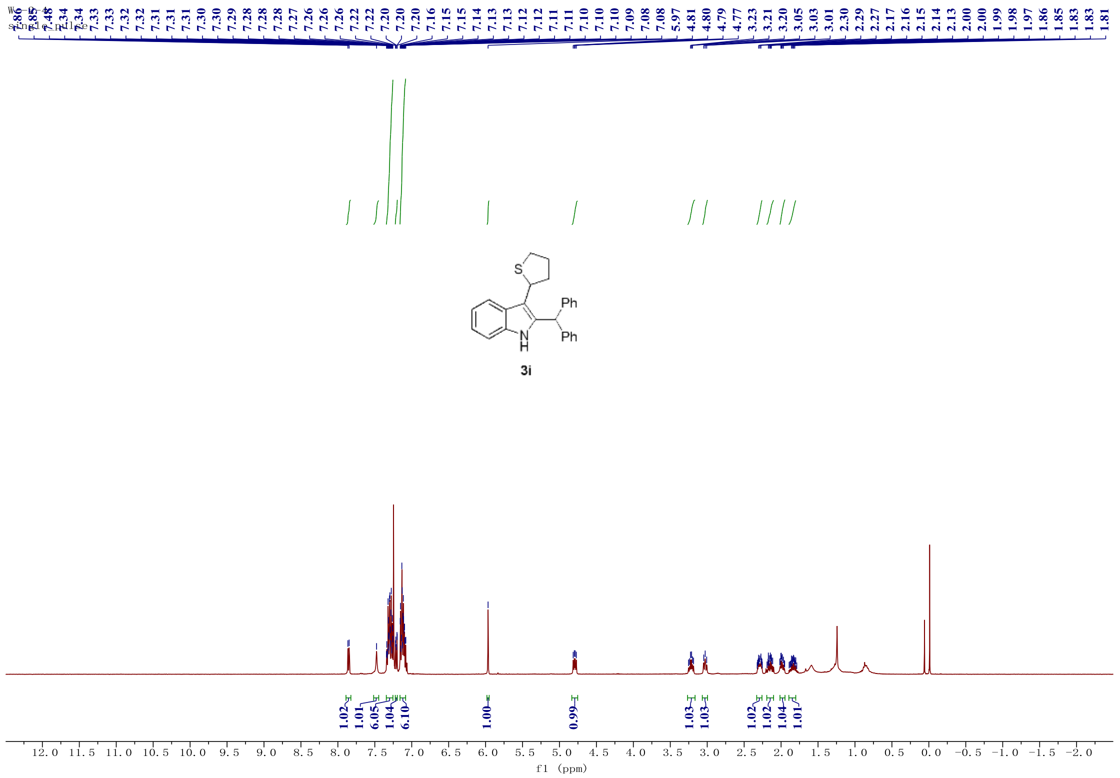

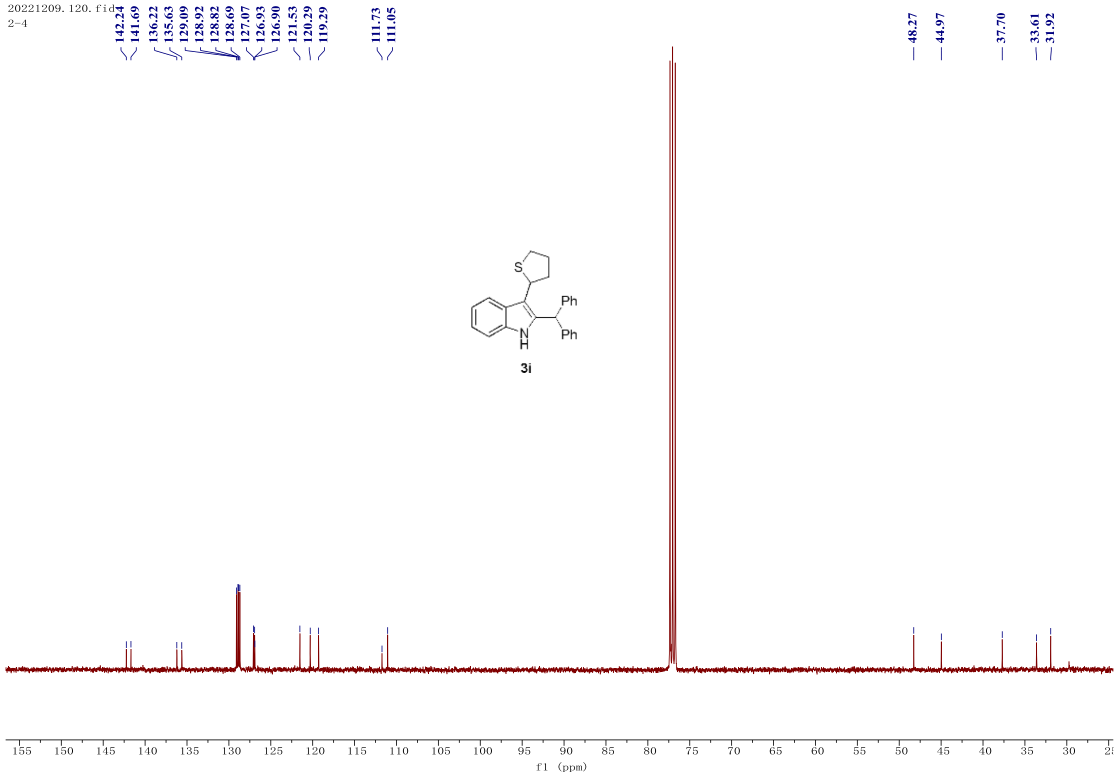

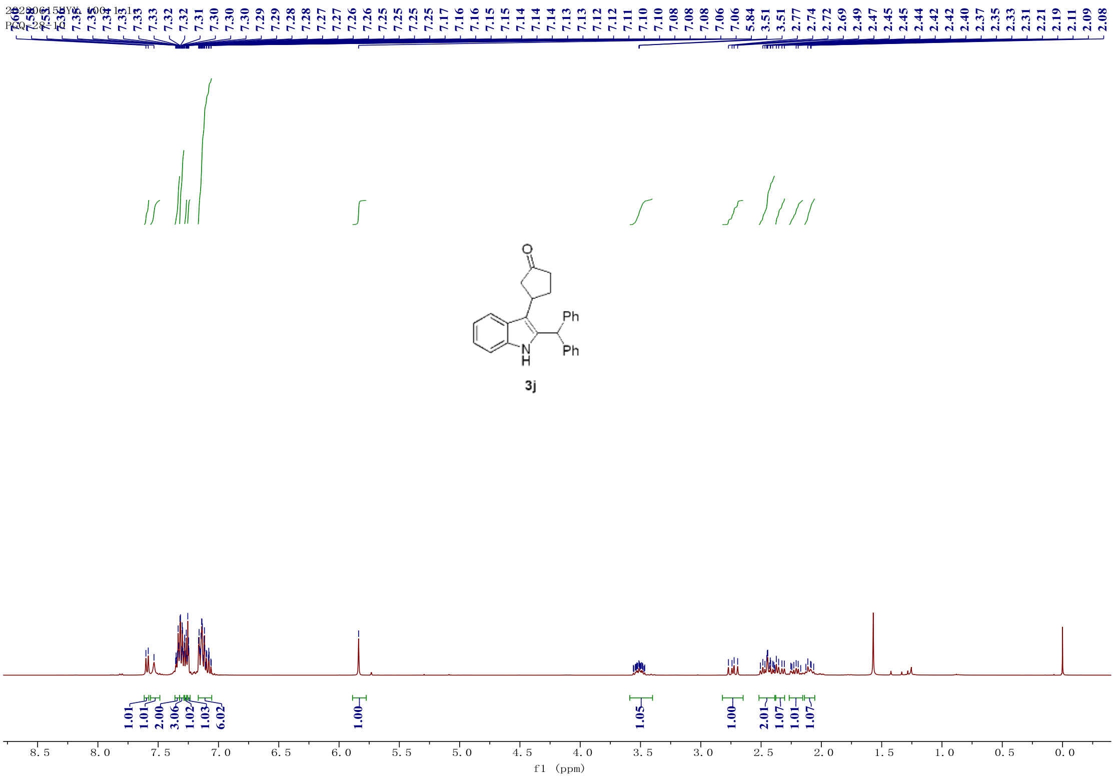
Ph
Ph
N
H
O
3j
1.01
1.01
2.00
3.06
1.02
1.03
6.02
1.00
1.05
1.00
2.01
1.07
1.01
1.07
8.5 8.0 7.5 7.0 6.5 6.0 5.5 5.0 4.5 4.0 3.5 3.0 2.5 2.0 1.5 1.0 0.5 0.0
f1 (ppm)

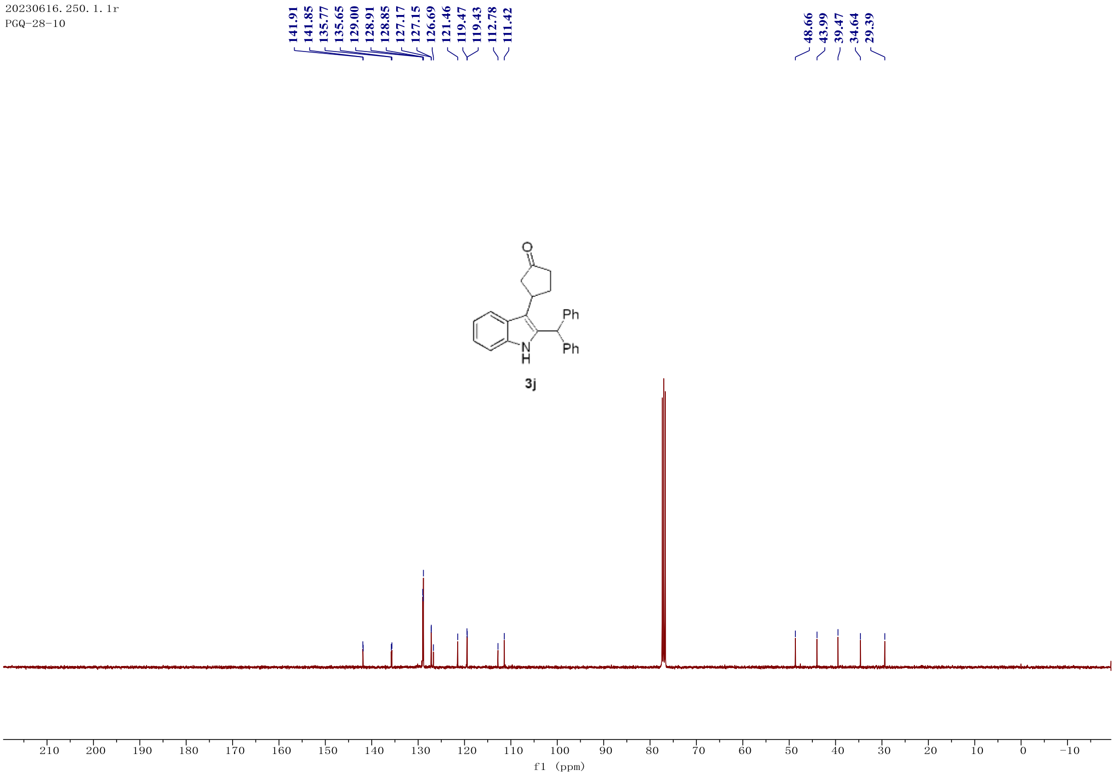
20230616.250.1.1r
PGQ-28-10
141.91
141.85
135.77
135.65
129.00
128.91
128.85
127.17
127.15
126.69
121.46
119.47
119.43
112.78
111.42
48.66
43.99
39.47
34.64
29.39
Ph
Ph
N
H
O
3j
210 200 190 180 170 160 150 140 130 120 110 100 90 80 70 60 50 40 30 20 10 0 -10
f1 (ppm)

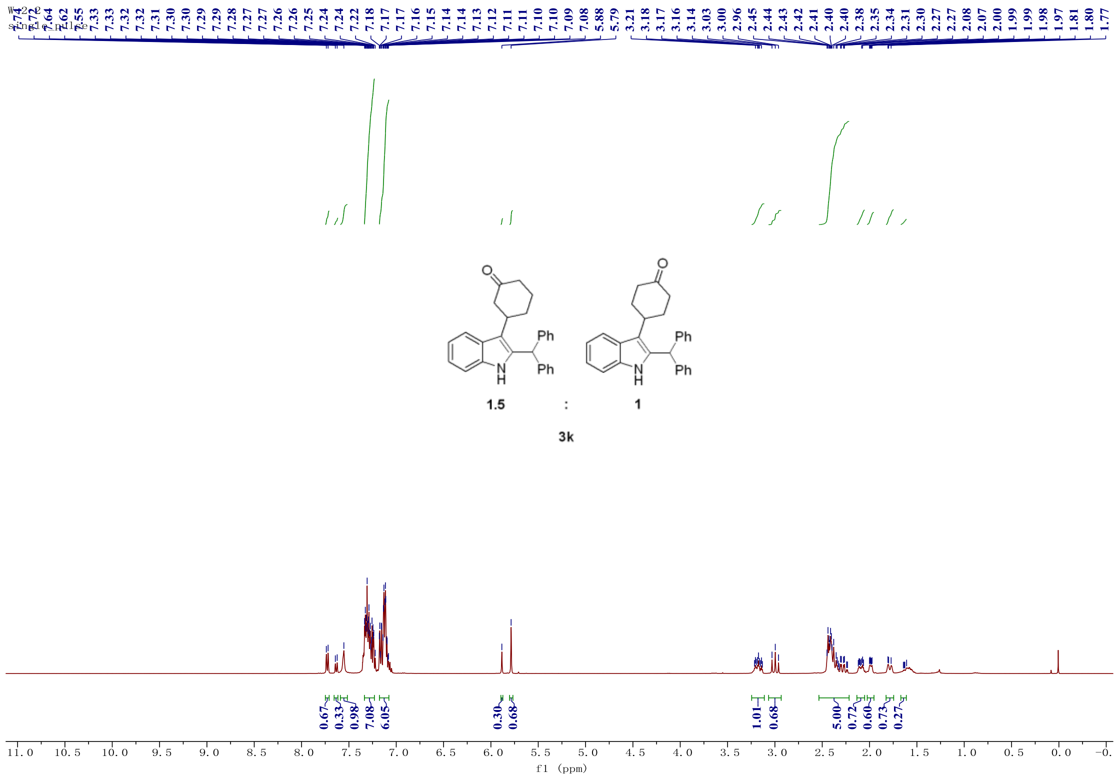
7.74 7.72 7.64 7.62 7.55 7.33 7.33 7.32 7.32 7.31 7.30 7.29 7.29 7.28 7.27 7.27 7.26 7.26 7.25 7.24 7.24 7.22 7.18 7.17 7.17 7.16 7.15 7.14 7.14 7.13 7.12 7.11 7.11 7.10 7.10 7.09 7.08 5.88 5.79 3.21 3.18 3.17 3.16 3.14 3.03 3.00 2.96 2.45 2.44 2.43 2.42 2.41 2.40 2.40 2.38 2.35 2.34 2.31 2.30 2.27 2.27 2.08 2.07 2.00 1.99 1.99 1.98 1.97 1.81 1.80 1.77
Ph
Ph
N
H
Ph
Ph
N
H
1.5
:
1
3k
0.67 0.33 0.98 7.08 6.05 0.30 0.68 1.01 0.68 5.00 0.72 0.60 0.73 0.27
11.0 10.5 10.0 9.5 9.0 8.5 8.0 7.5 7.0 6.5 6.0 5.5 5.0 4.5 4.0 3.5 3.0 2.5 2.0 1.5 1.0 0.5 0.0
f1 (ppm)

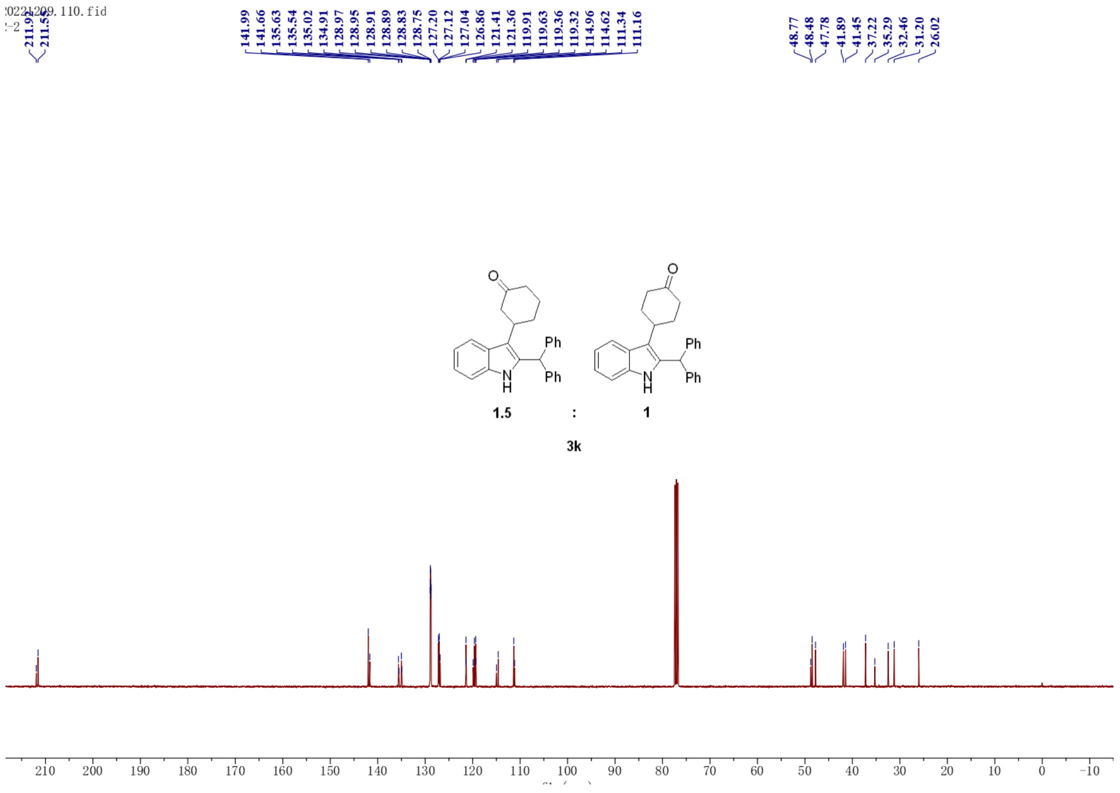
20231209.110.fid
211.91 211.55
141.99 141.66 135.63 135.54 135.02 134.91 128.97 128.95 128.91 128.89 128.83 128.75 127.20 127.12 127.04 126.86 121.41 121.36 119.91 119.63 119.36 119.32 114.96 114.62 111.34 111.16
48.77 48.48 47.78 41.89 41.45 37.22 35.29 32.46 31.20 26.02
Ph
Ph
N
H
Ph
Ph
N
H
1.5
:
1
3k
210 200 190 180 170 160 150 140 130 120 110 100 90 80 70 60 50 40 30 20 10 0 -10

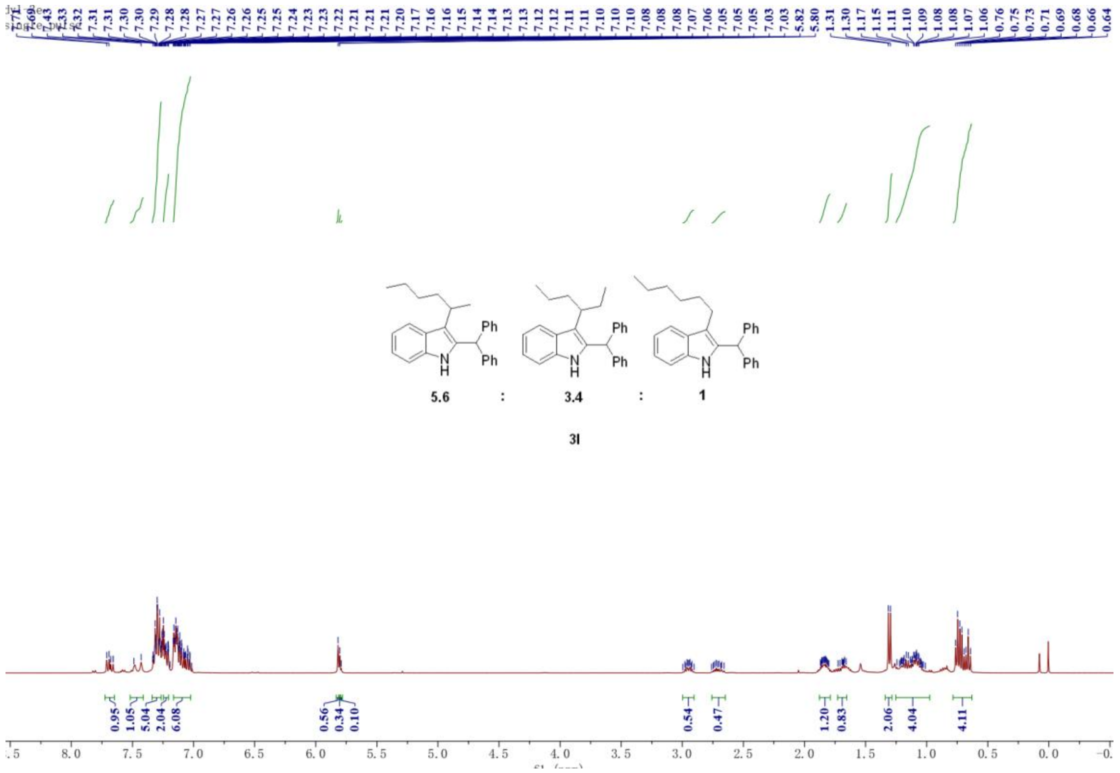
5.6 : 3.4 : 1
3l

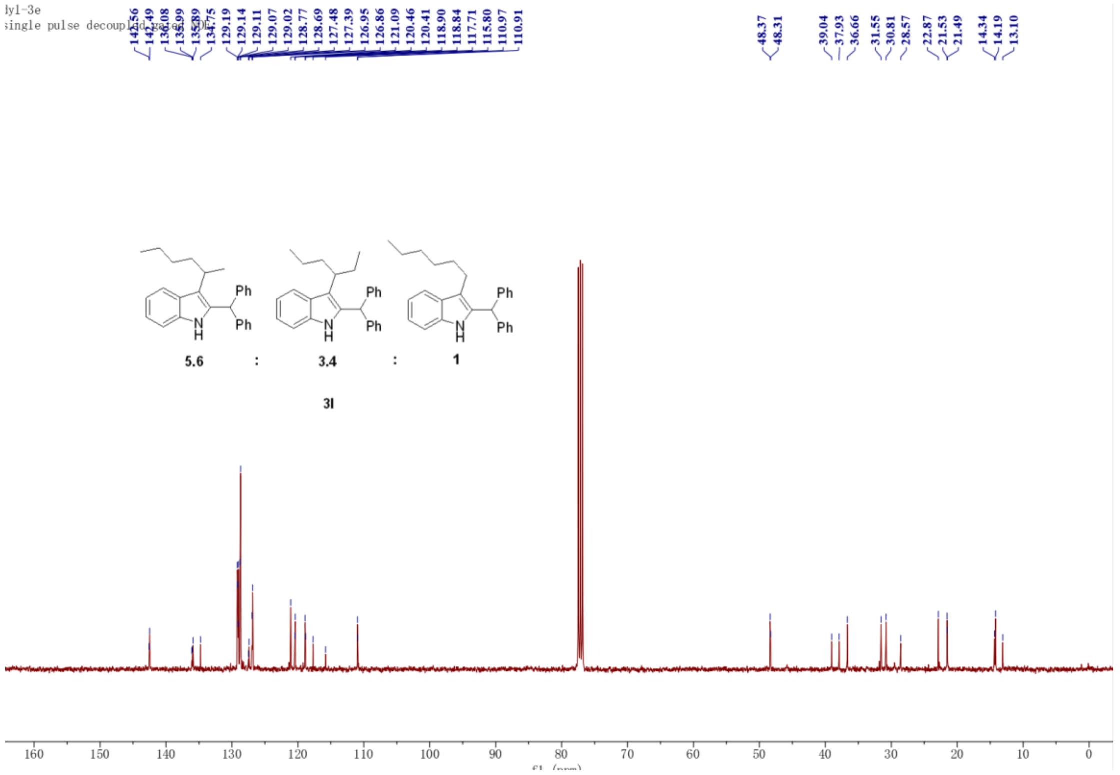
5.6 : 3.4 : 1
3l

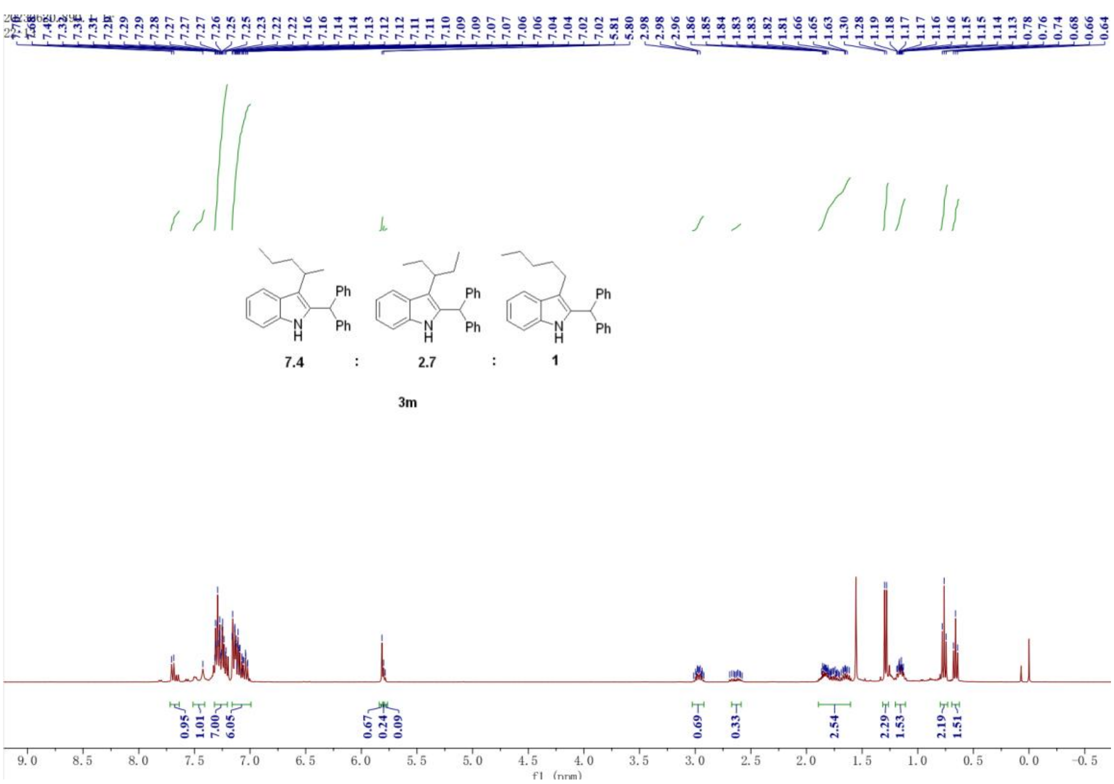
7.70 7.68 7.42 7.32 7.31 7.31 7.29 7.29 7.28 7.27 7.27 7.27 7.26 7.25 7.25 7.23 7.22 7.22 7.16 7.16 7.14 7.14 7.13 7.12 7.12 7.11 7.10 7.09 7.09 7.07 7.07 7.06 7.06 7.04 7.04 7.02 7.02 5.81 5.80 2.98 2.98 2.96 1.86 1.85 1.84 1.83 1.83 1.82 1.81 1.66 1.65 1.63 1.30 1.28 1.19 1.18 1.17 1.17 1.16 1.16 1.15 1.15 1.14 1.13 0.78 0.76 0.74 0.68 0.66 0.64
Ph
Ph
N
H
7.4
:
2.7
:
1
3m
0.95 1.01 7.00 6.05 0.67 0.24 0.09 0.69 0.33 2.54 2.29 1.53 2.19 1.51
9.0 8.5 8.0 7.5 7.0 6.5 6.0 5.5 5.0 4.5 4.0 3.5 3.0 2.5 2.0 1.5 1.0 0.5 0.0 -0.5
f1 (ppm)

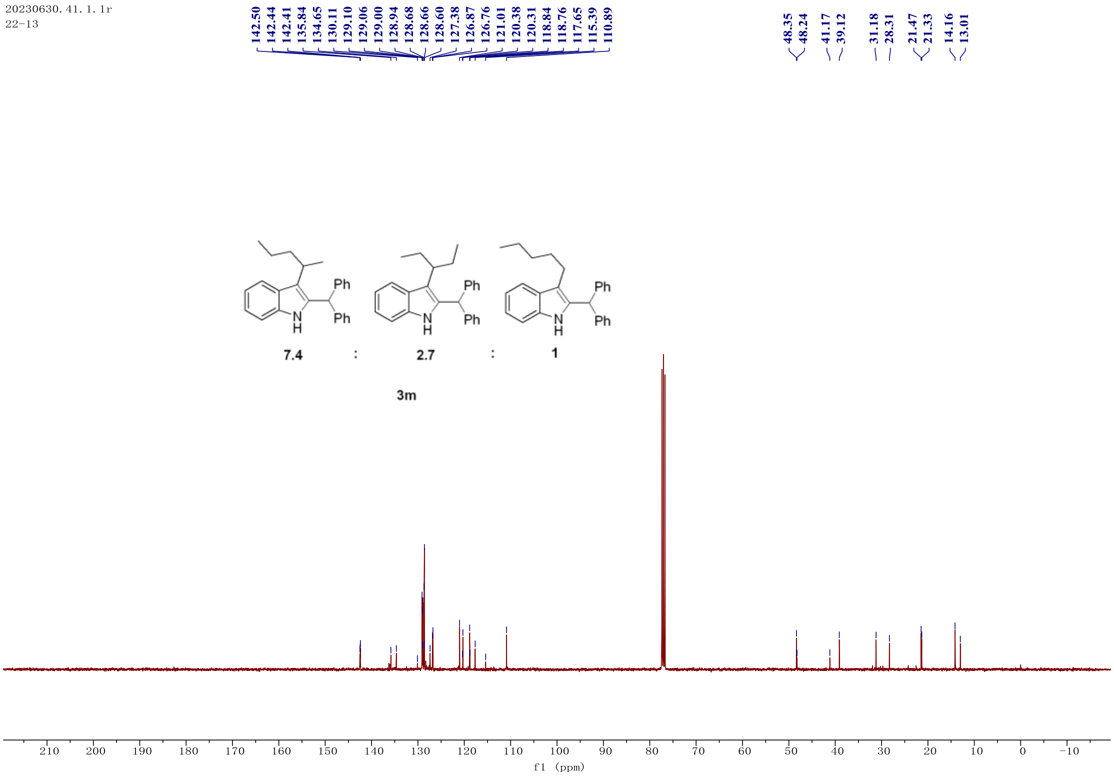
20230630.41.1.1r
22-13
142.50 142.44 142.41 135.84 134.65 130.11 129.10 129.06 129.00 128.94 128.68 128.66 128.60 127.38 126.87 126.76 121.01 120.38 120.31 118.84 118.76 117.65 115.39 110.89
48.35 48.24 41.17 39.12 31.18 28.31 21.47 21.33 14.16 13.01
Ph
Ph
N
H
7.4
:
2.7
:
1
3m
210 200 190 180 170 160 150 140 130 120 110 100 90 80 70 60 50 40 30 20 10 0 -10
f1 (ppm)

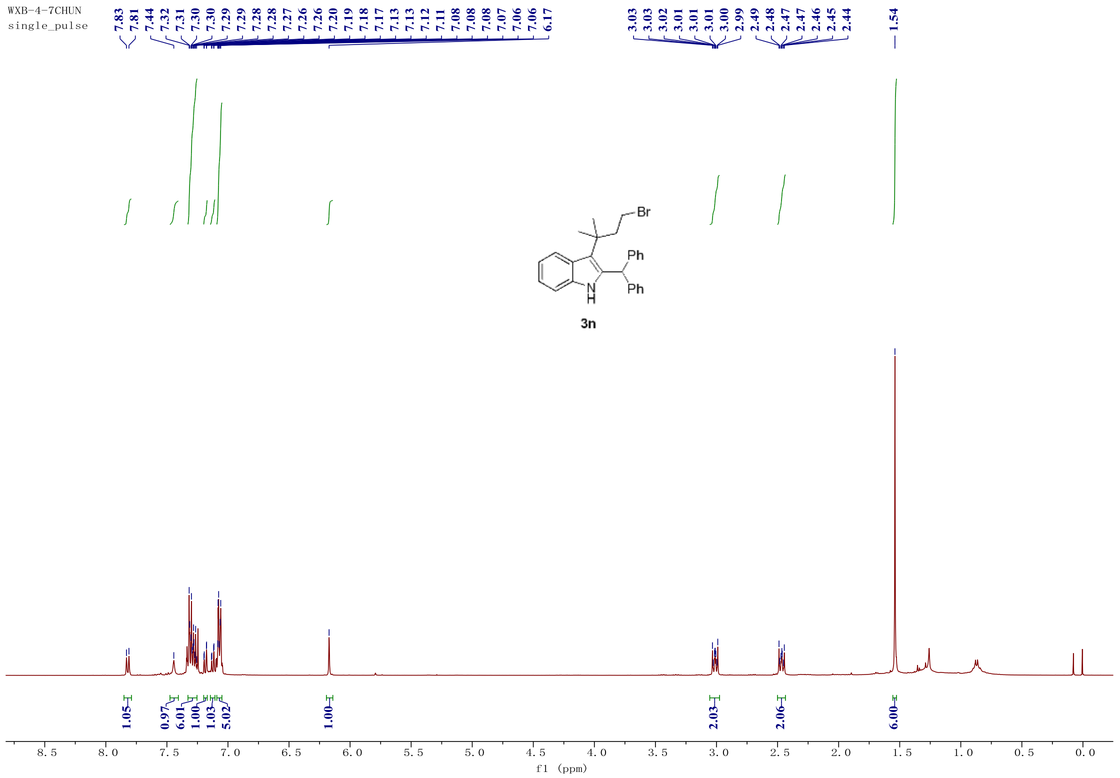
WXB-4-7CHUN
single_pulse
7.83
7.81
7.44
7.32
7.31
7.30
7.30
7.29
7.29
7.28
7.28
7.27
7.26
7.26
7.20
7.19
7.18
7.17
7.13
7.13
7.12
7.11
7.08
7.08
7.07
7.06
7.06
6.17
3.03
3.03
3.02
3.01
3.01
3.00
2.99
2.49
2.48
2.47
2.47
2.46
2.45
2.44
1.54
Br
Ph
Ph
N
H
3n
1.05
0.97
6.01
1.00
1.03
5.02
1.00
2.03
2.06
6.00
f1 (ppm)

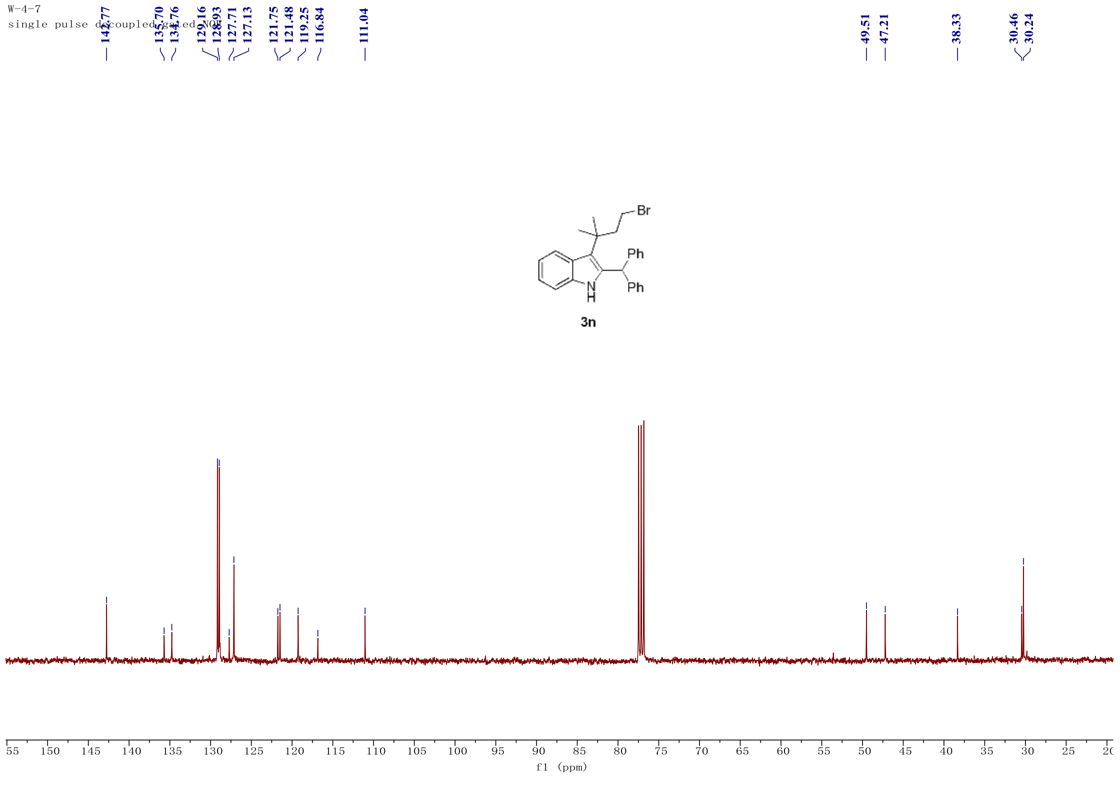
W-4-7
single pulse decoupled gated NOE
142.77
135.70
134.76
129.16
128.93
127.71
127.13
121.75
121.48
119.25
116.84
111.04
49.51
47.21
38.33
30.46
30.24
Br
Ph
Ph
N
H
3n
f1 (ppm)

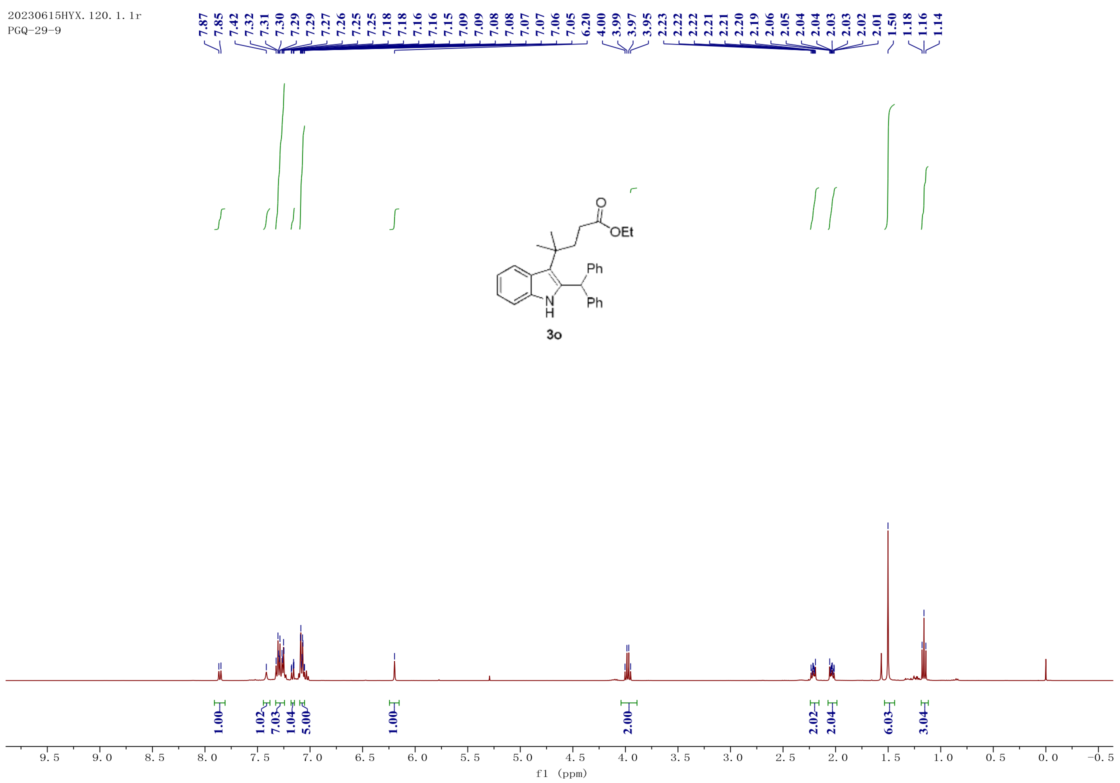
20230615HYX.120.1.1r
PGQ-29-9
7.87 7.85 7.42 7.32 7.31 7.30 7.29 7.29 7.27 7.26 7.25 7.25 7.18 7.18 7.16 7.16 7.15 7.09 7.09 7.08 7.08 7.07 7.07 7.06 7.05 6.20 4.00 3.99 3.97 3.95 2.23 2.22 2.22 2.21 2.21 2.20 2.19 2.06 2.05 2.04 2.04 2.03 2.03 2.02 2.01 1.50 1.18 1.16 1.14
OEt
Ph
Ph
N
H
3o
1.00 1.02 7.03 1.04 5.00 1.00 2.00 2.02 2.04 6.03 3.04
9.5 9.0 8.5 8.0 7.5 7.0 6.5 6.0 5.5 5.0 4.5 4.0 3.5 3.0 2.5 2.0 1.5 1.0 0.5 0.0 -0.5
f1 (ppm)

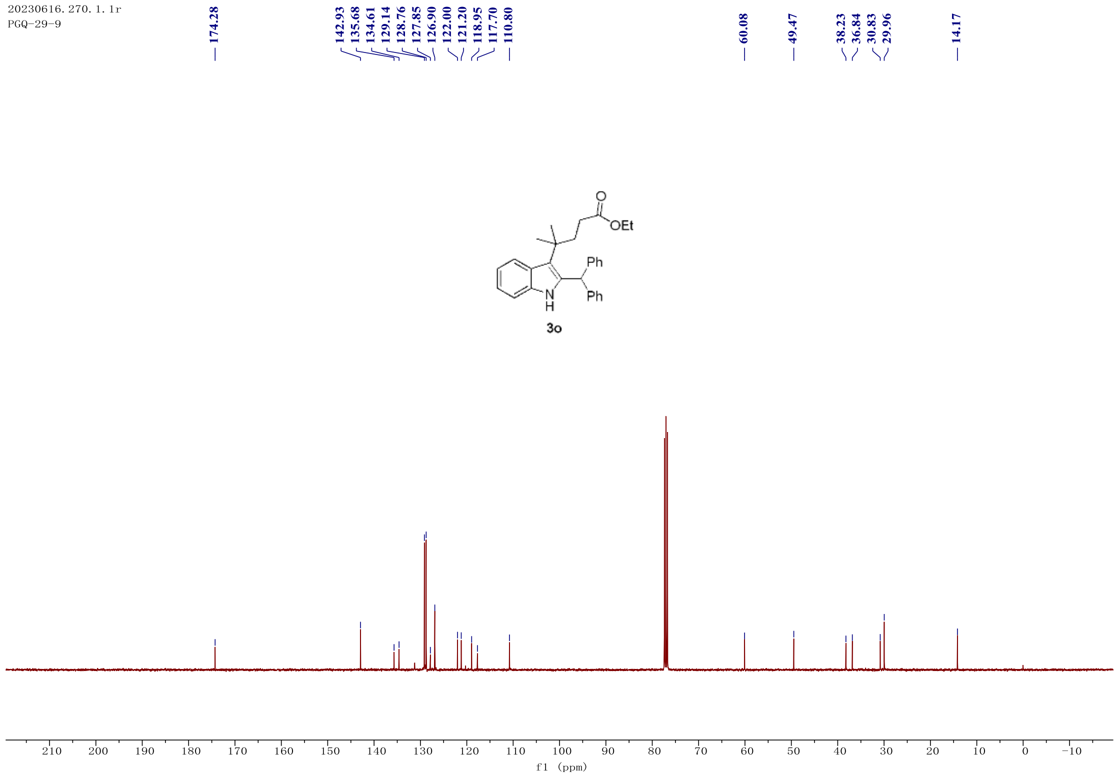
20230616.270.1.1r
PGQ-29-9
174.28
142.93 135.68 134.61 129.14 128.76 127.85 126.90 122.00 121.20 118.95 117.70 110.80
60.08
49.47
38.23 36.84 30.83 29.96
14.17
OEt
Ph
Ph
N
H
3o
210 200 190 180 170 160 150 140 130 120 110 100 90 80 70 60 50 40 30 20 10 0 -10
f1 (ppm)

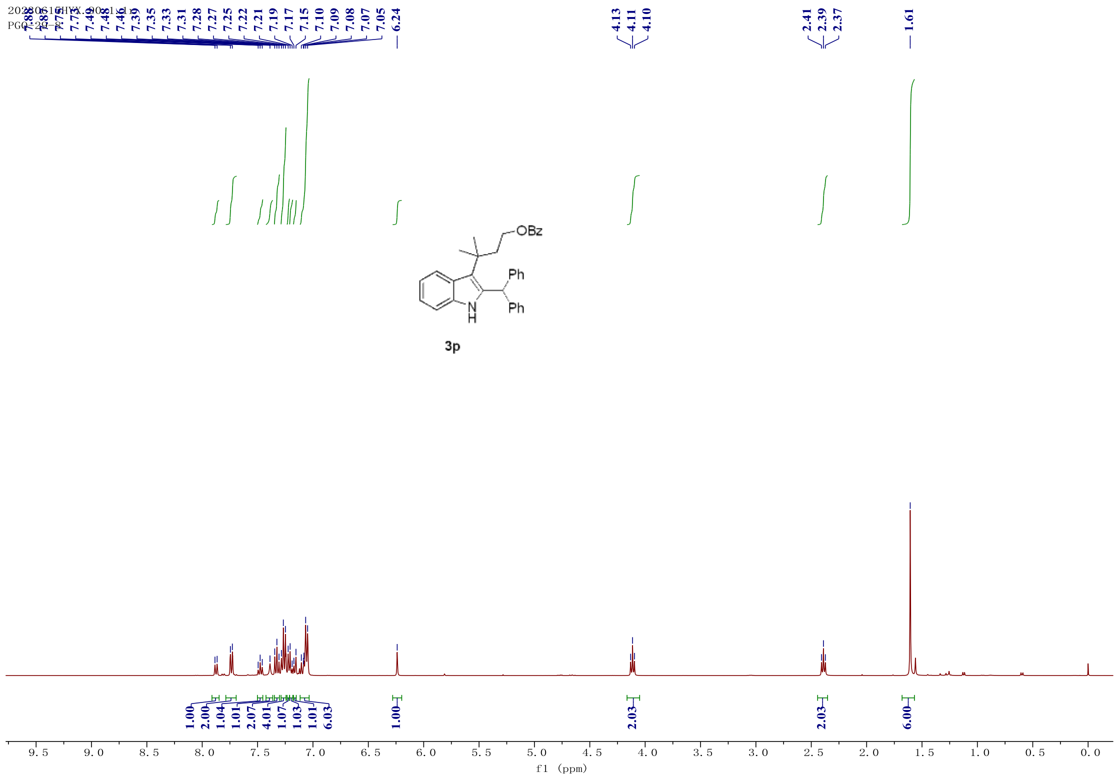
OBz
Ph
Ph
N
H
3p
f1 (ppm)

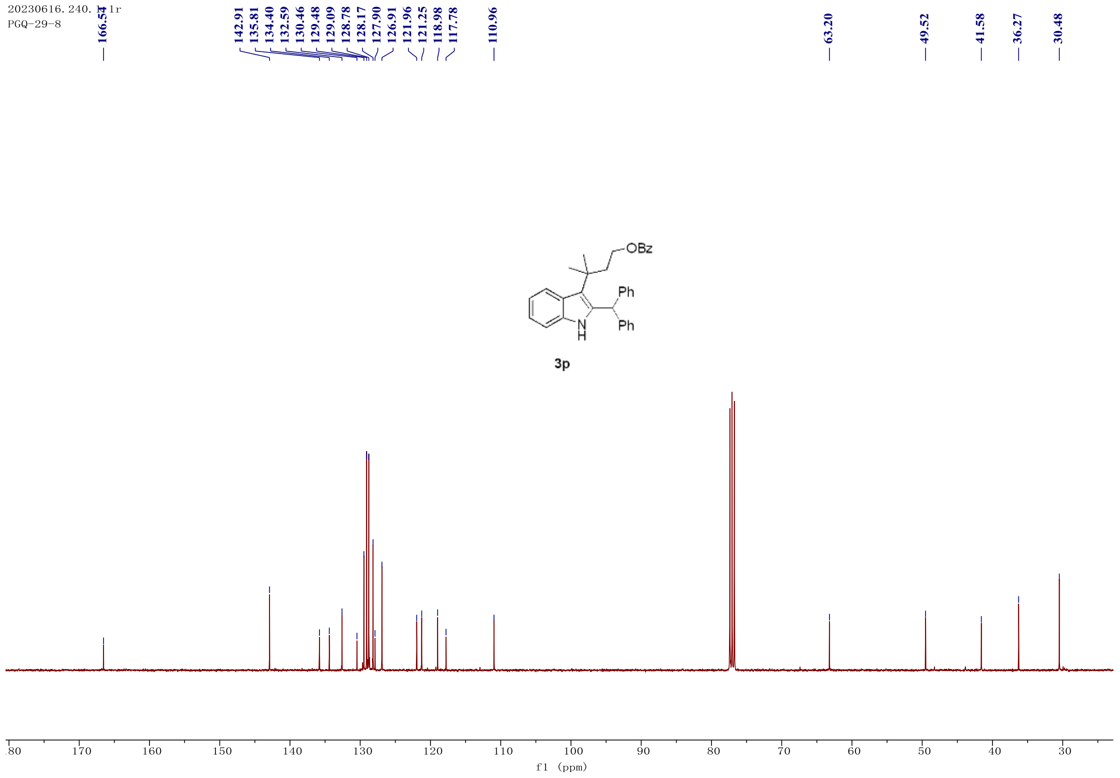
20230616.240.11r
PGQ-29-8
OBz
Ph
Ph
N
H
3p
f1 (ppm)

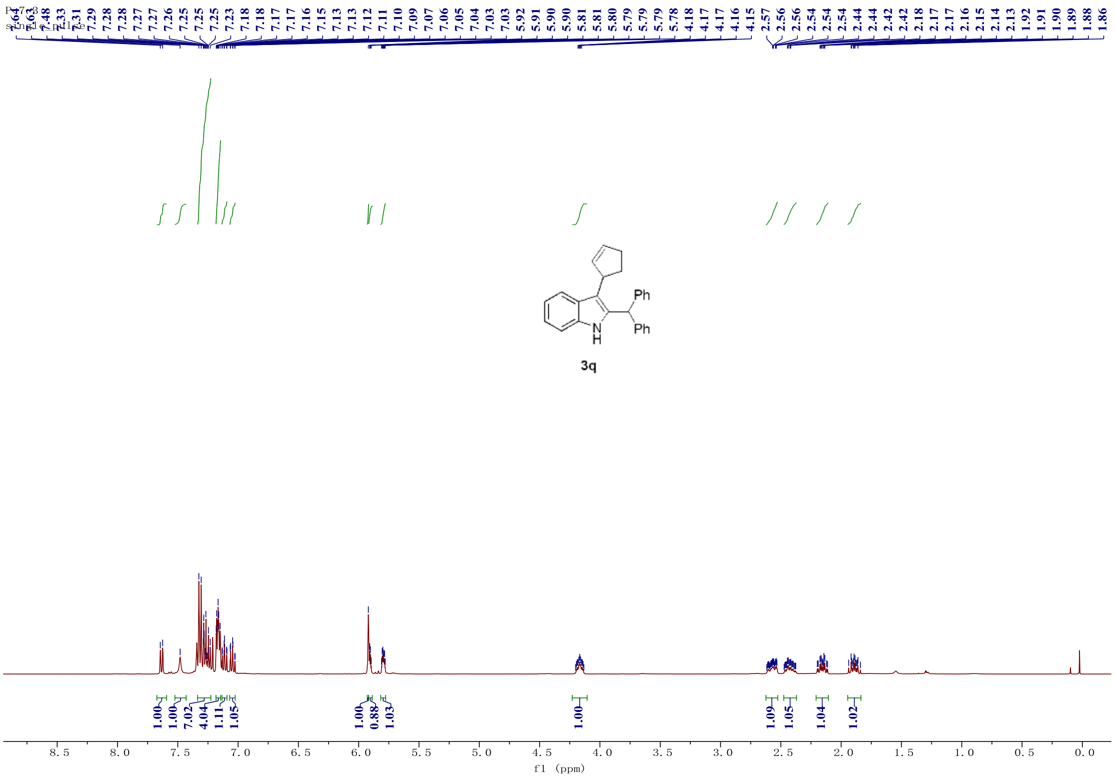
7.63
7.48
7.33
7.31
7.29
7.28
7.27
7.27
7.26
7.25
7.25
7.25
7.23
7.18
7.18
7.17
7.17
7.16
7.15
7.13
7.13
7.12
7.11
7.10
7.09
7.07
7.06
7.05
7.04
7.03
7.03
5.92
5.91
5.90
5.90
5.81
5.81
5.80
5.79
5.79
5.79
5.78
4.18
4.17
4.17
4.16
4.15
2.57
2.56
2.56
2.54
2.54
2.54
2.44
2.44
2.42
2.18
2.17
2.17
2.16
2.15
2.14
2.13
1.92
1.91
1.90
1.89
1.88
1.86
Ph
Ph
N
H
3q
1.00
1.00
7.02
4.04
1.11
1.05
1.00
0.88
1.03
1.00
1.09
1.05
1.04
1.02
f1 (ppm)

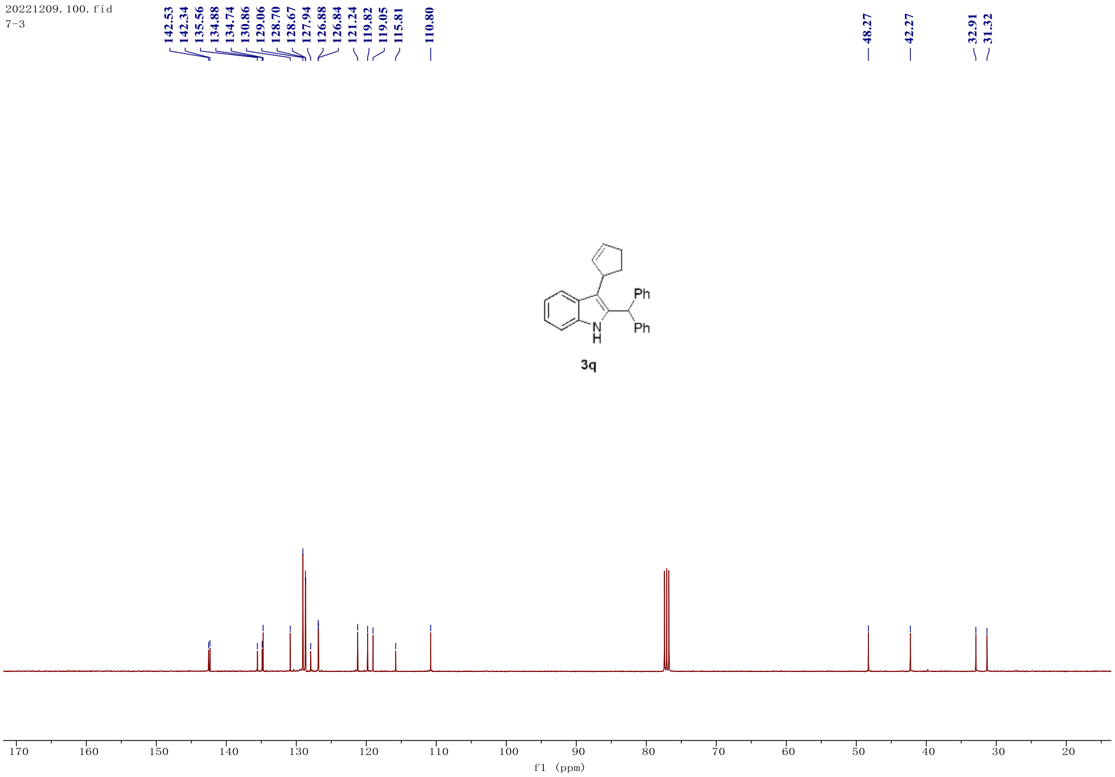
20221209.100.fid
7-3
142.53
142.34
135.56
134.88
134.74
130.86
129.06
128.70
128.67
127.94
126.88
126.84
121.24
119.82
119.05
115.81
110.80
48.27
42.27
32.91
31.32
Ph
Ph
N
H
3q
f1 (ppm)

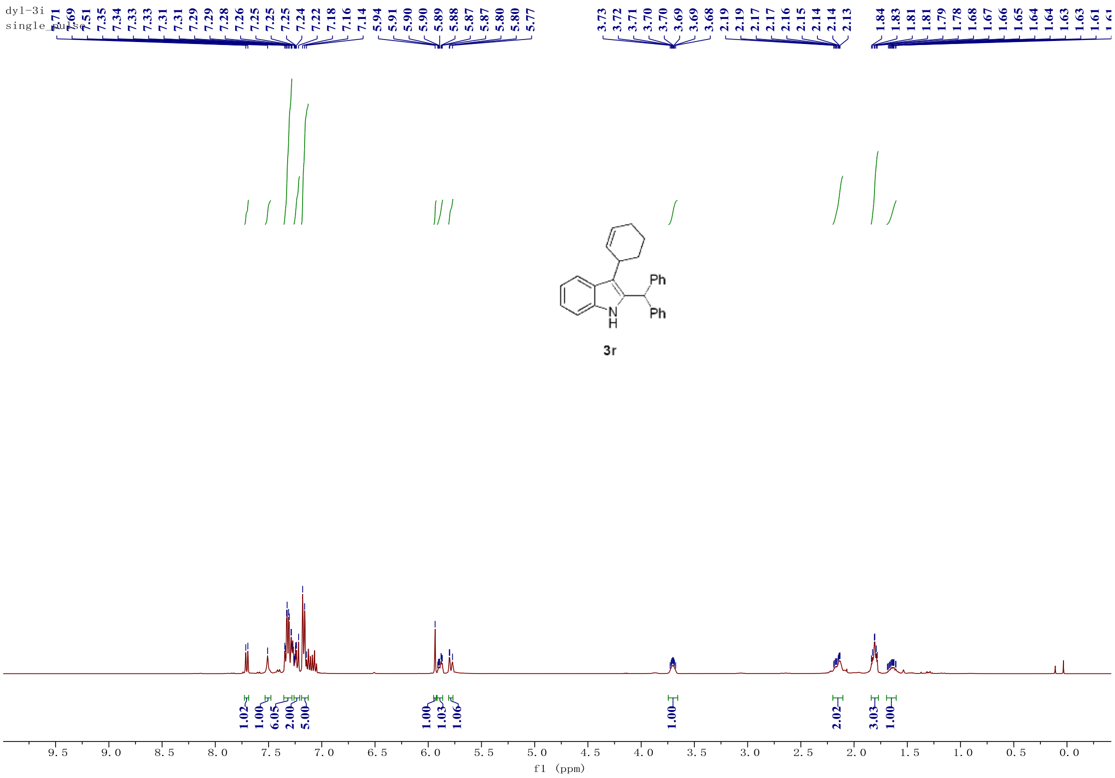
dyl-3i
single_pulse
3r
f1 (ppm)

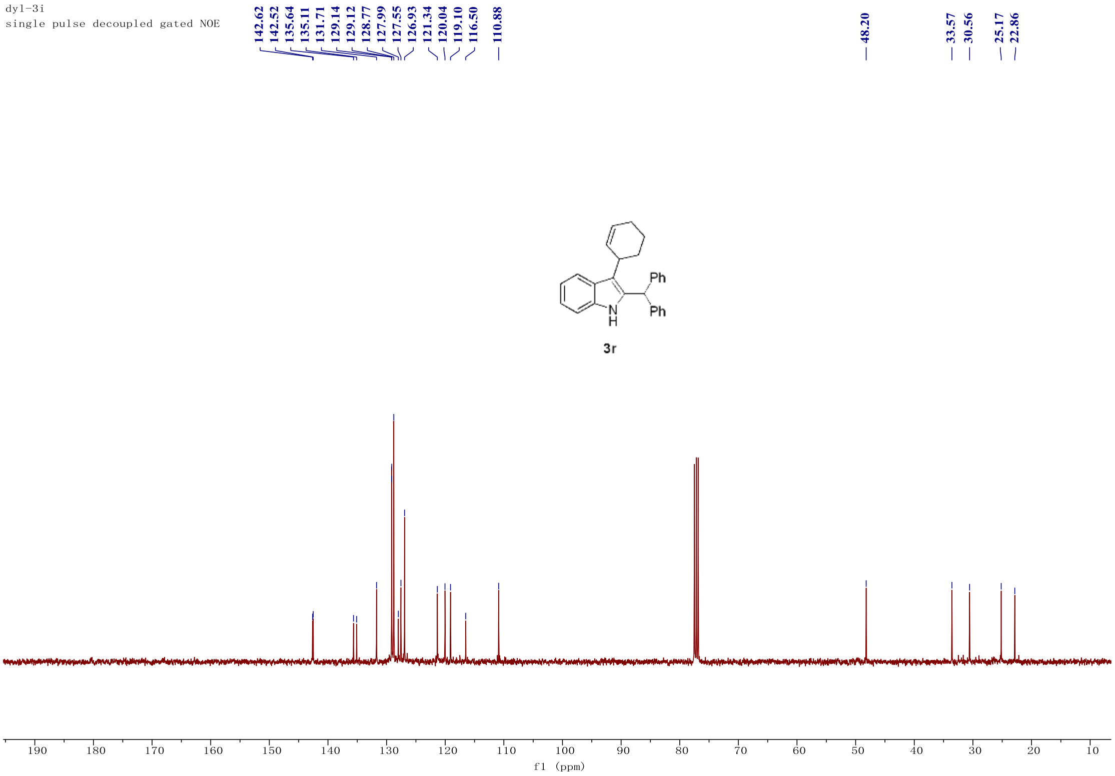
dyl-3i
single pulse decoupled gated NOE
142.62
142.52
135.64
135.11
131.71
129.14
129.12
128.77
127.99
127.55
126.93
121.34
120.04
119.10
116.50
110.88
48.20
33.57
30.56
25.17
22.86
3r
f1 (ppm)

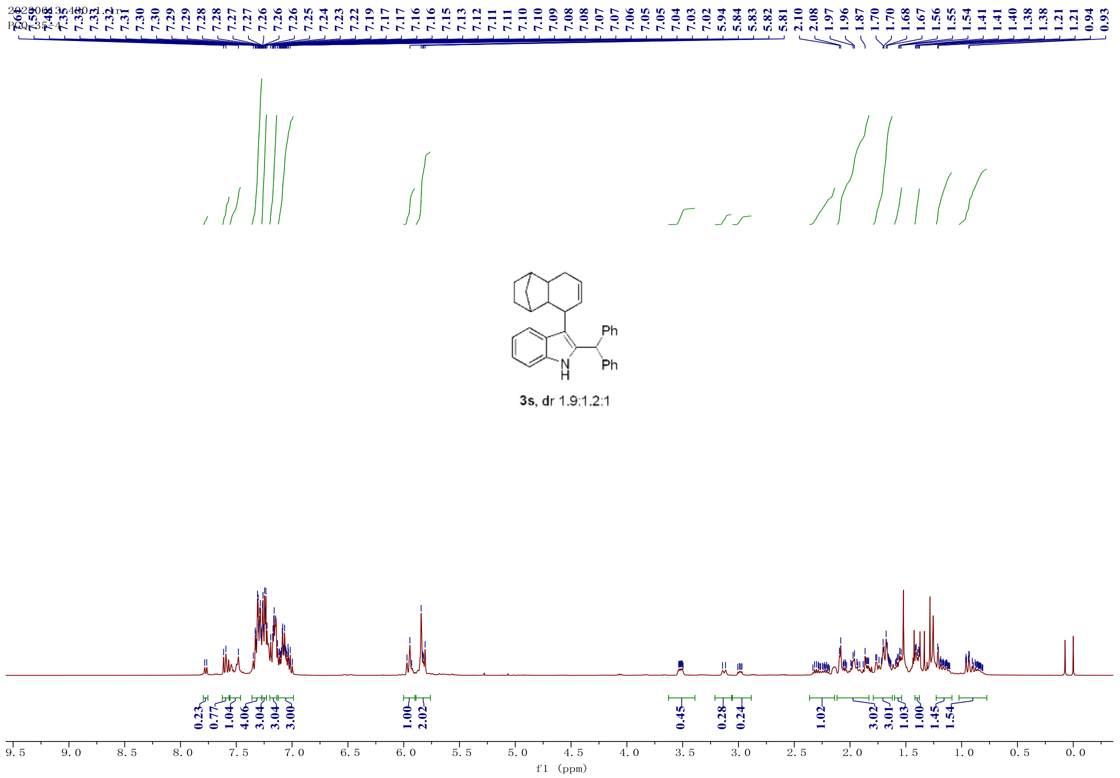
Ph
Ph
N
H
3s, dr 1.9:1.2:1
f1 (ppm)

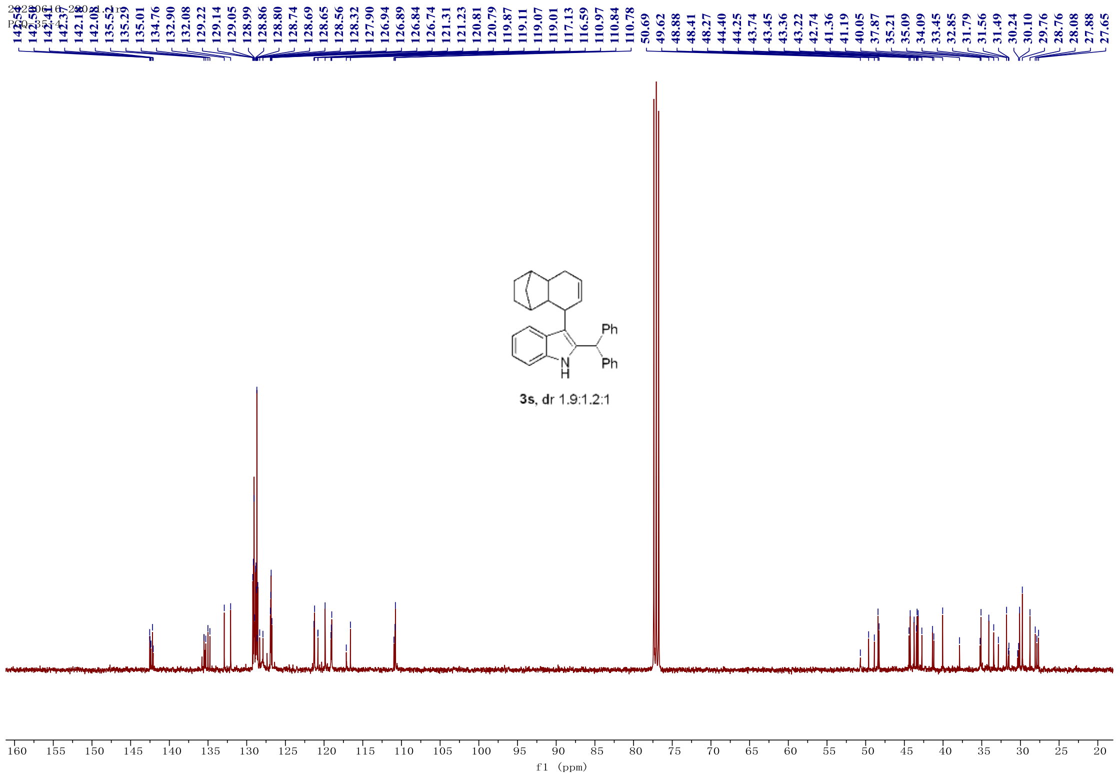
Ph
Ph
N
H
3s, dr 1.9:1.2:1
f1 (ppm)

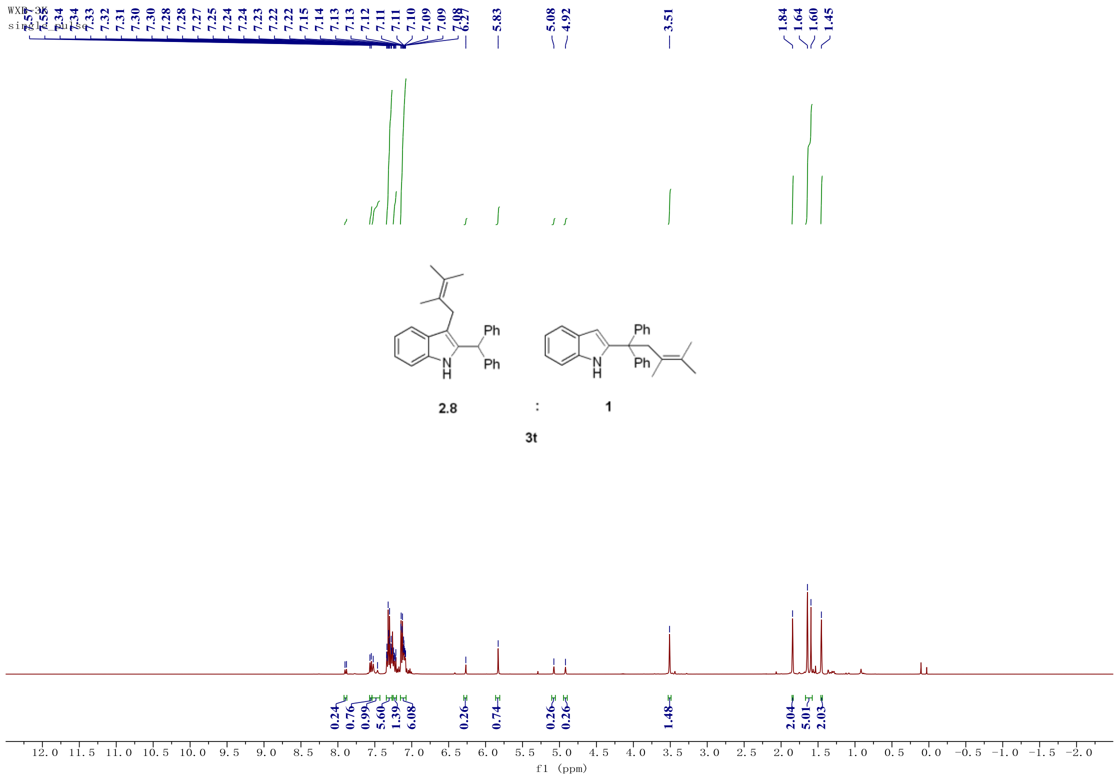

WXB-3K
single pulse
7.34
7.34
7.33
7.32
7.31
7.30
7.30
7.28
7.28
7.27
7.25
7.24
7.24
7.23
7.22
7.22
7.22
7.15
7.14
7.13
7.13
7.12
7.11
7.11
7.10
7.09
7.09
7.08
6.27
5.83
5.08
4.92
3.51
1.84
1.64
1.60
1.45
Ph
Ph
N
H
Ph
Ph
N
H
2.8
:
1
3t
0.24
0.76
0.99
5.60
1.39
6.08
0.26
0.74
0.26
0.26
1.48
2.04
5.01
2.03
12.0 11.5 11.0 10.5 10.0 9.5 9.0 8.5 8.0 7.5 7.0 6.5 6.0 5.5 5.0 4.5 4.0 3.5 3.0 2.5 2.0 1.5 1.0 0.5 0.0 -0.5 -1.0 -1.5 -2.0
f1 (ppm)

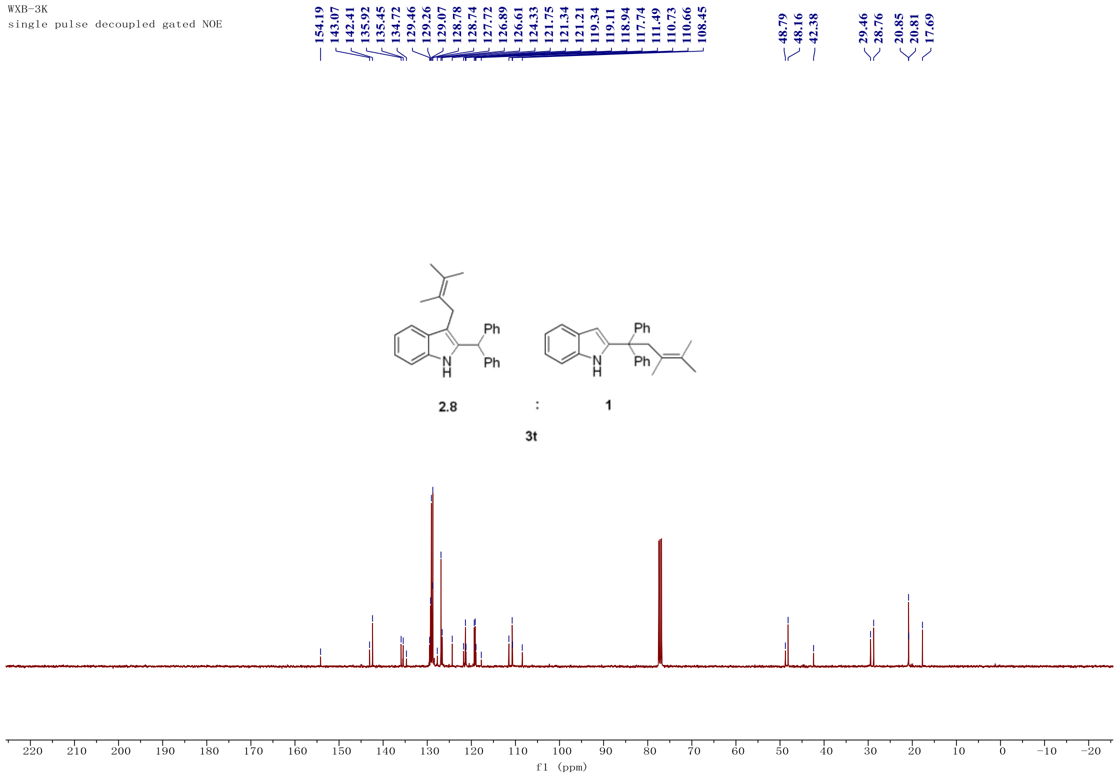

WXB-3K
single pulse decoupled gated NOE
154.19
143.07
142.41
135.92
135.45
134.72
129.46
129.26
129.07
128.78
128.74
127.72
126.89
126.61
124.33
121.75
121.34
121.21
119.34
119.11
118.94
117.74
111.49
110.73
110.66
108.45
48.79
48.16
42.38
29.46
28.76
20.85
20.81
17.69
Ph
Ph
N
H
Ph
Ph
N
H
2.8
:
1
3t
220 210 200 190 180 170 160 150 140 130 120 110 100 90 80 70 60 50 40 30 20 10 0 -10 -20
f1 (ppm)

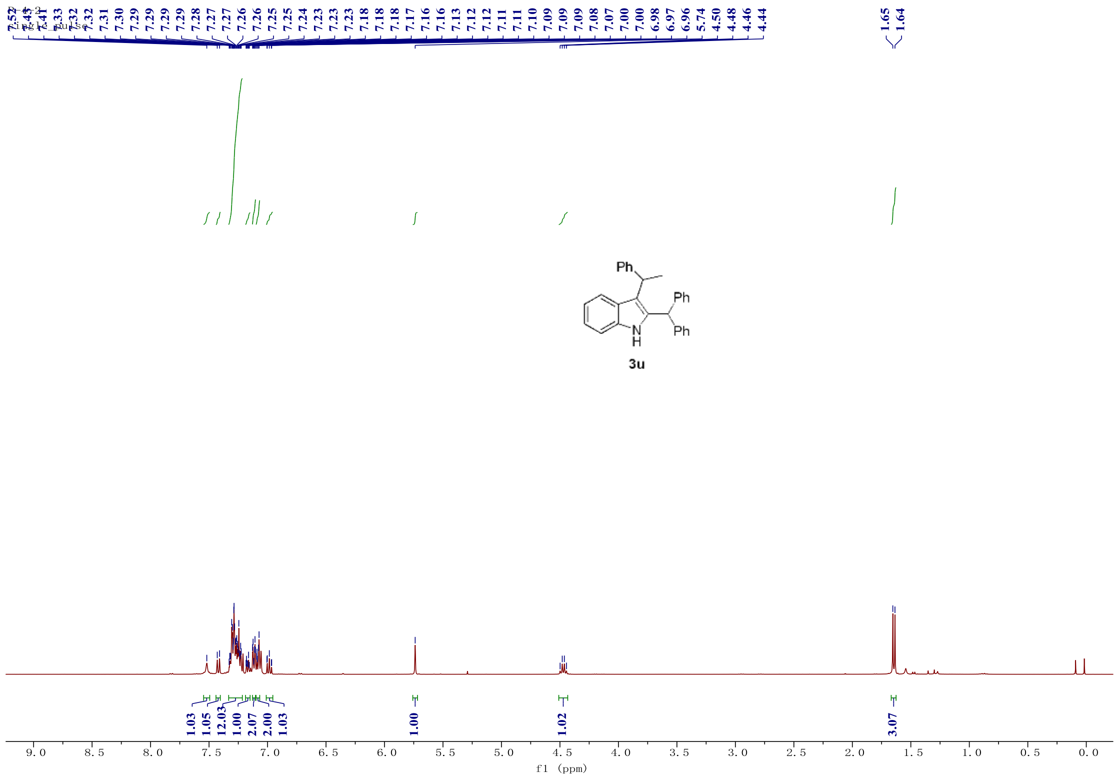
7.33
7.32
7.31
7.30
7.29
7.29
7.29
7.29
7.28
7.27
7.27
7.26
7.26
7.25
7.25
7.24
7.23
7.23
7.23
7.18
7.18
7.17
7.16
7.13
7.12
7.12
7.11
7.11
7.10
7.09
7.09
7.09
7.08
7.07
7.00
6.98
6.97
6.96
5.74
4.50
4.48
4.46
4.44
1.65
1.64
Ph
Ph
Ph
H
3u
1.03
1.05
12.03
1.00
2.07
2.00
1.03
1.00
1.02
3.07
f1 (ppm)

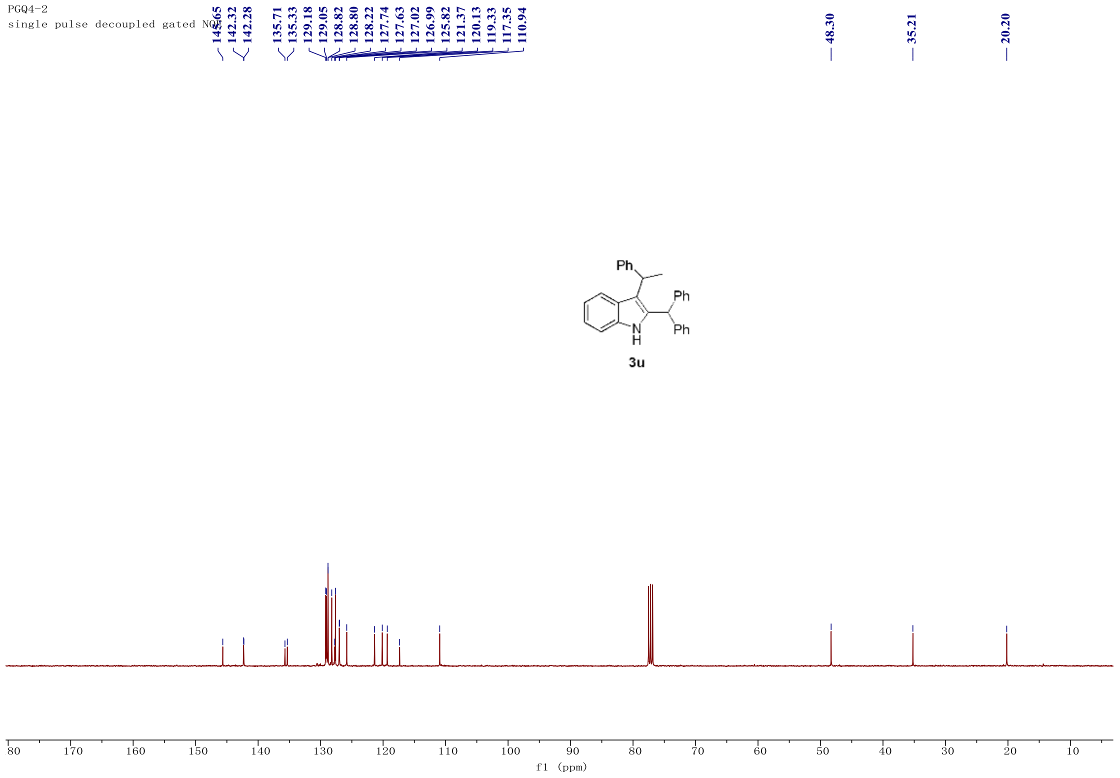
PGQ4-2
single pulse decoupled gated NOE
142.32
142.28
135.71
135.33
129.18
129.05
128.82
128.80
128.22
127.74
127.63
127.02
126.99
125.82
121.37
120.13
119.33
117.35
110.94
48.30
35.21
20.20
Ph
Ph
Ph
H
3u
f1 (ppm)

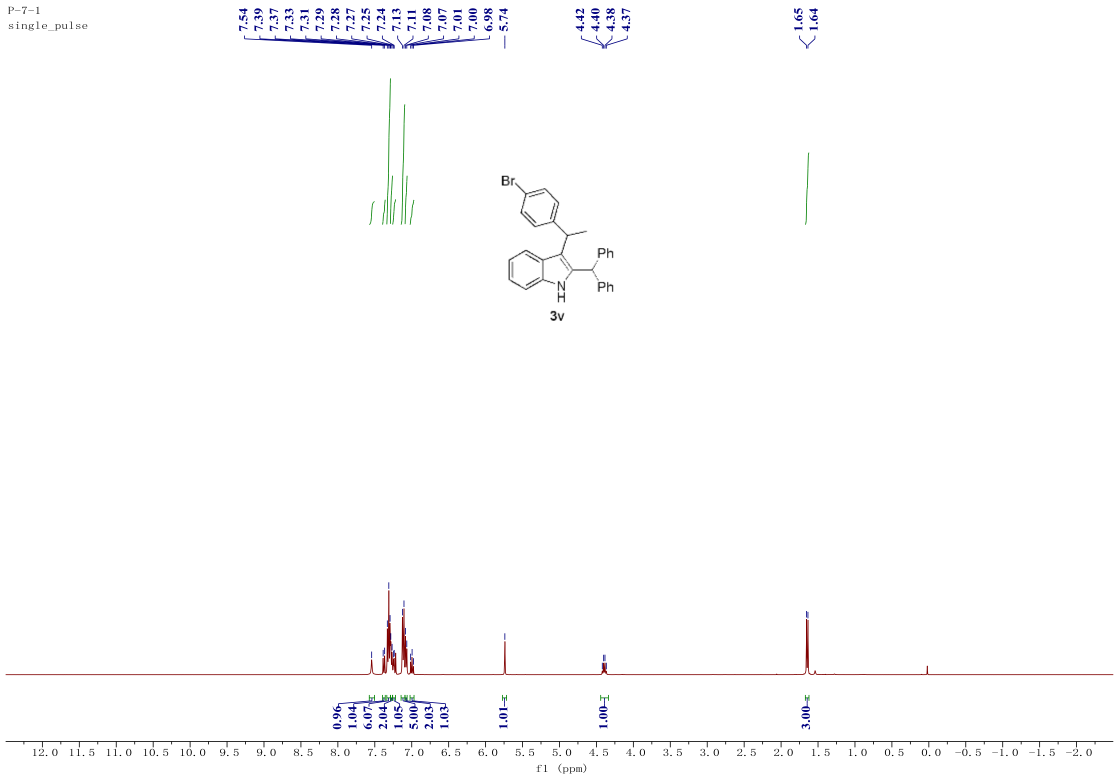
P-7-1
single_pulse
7.54 7.39 7.37 7.33 7.31 7.29 7.28 7.27 7.25 7.24 7.13 7.11 7.08 7.07 7.01 7.00 6.98
5.74
4.42 4.40 4.38 4.37
1.65 1.64
Br
Ph
Ph
N
H
3v
0.96 1.04 6.07 2.04 1.05 5.00 2.03 1.03
1.01
1.00
3.00
12.0 11.5 11.0 10.5 10.0 9.5 9.0 8.5 8.0 7.5 7.0 6.5 6.0 5.5 5.0 4.5 4.0 3.5 3.0 2.5 2.0 1.5 1.0 0.5 0.0 −0.5 −1.0 −1.5 −2.0
f1 (ppm)

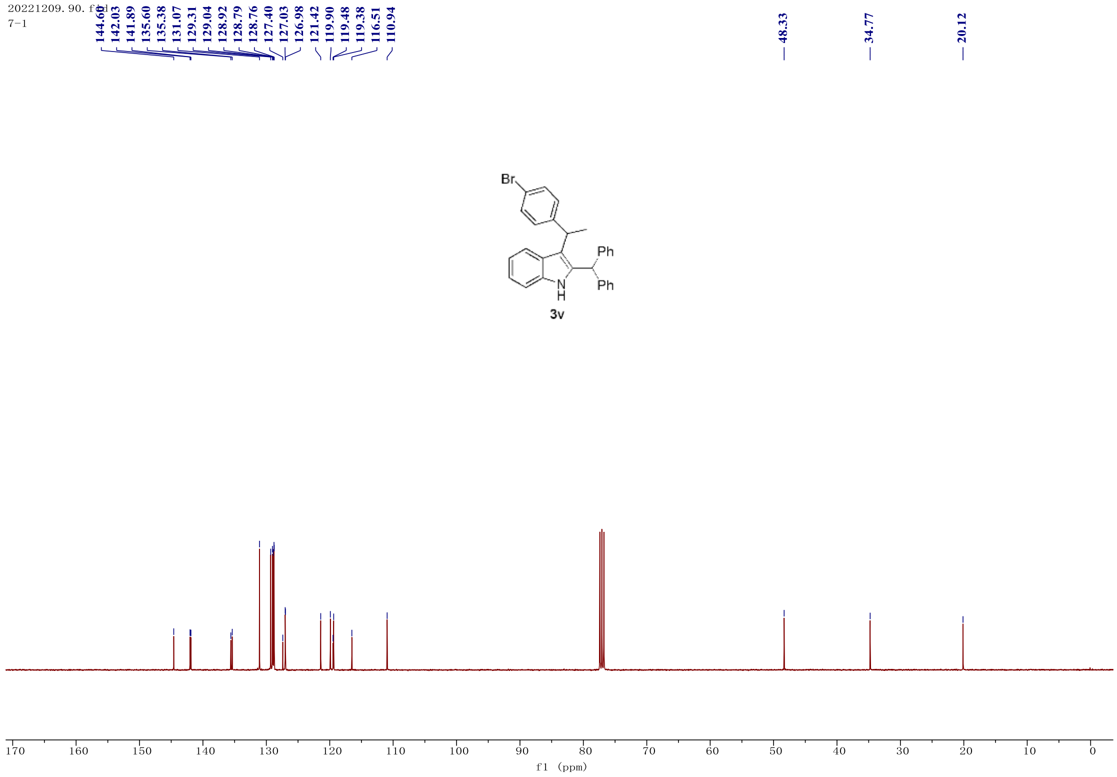
7-1
144.60 142.03 141.89 135.60 135.38 131.07 129.31 129.04 128.92 128.79 128.76 127.40 127.03 126.98 121.42 119.90 119.48 119.38 116.51 110.94
48.33
34.77
20.12
Br
Ph
Ph
N
H
3v
170 160 150 140 130 120 110 100 90 80 70 60 50 40 30 20 10 0
f1 (ppm)

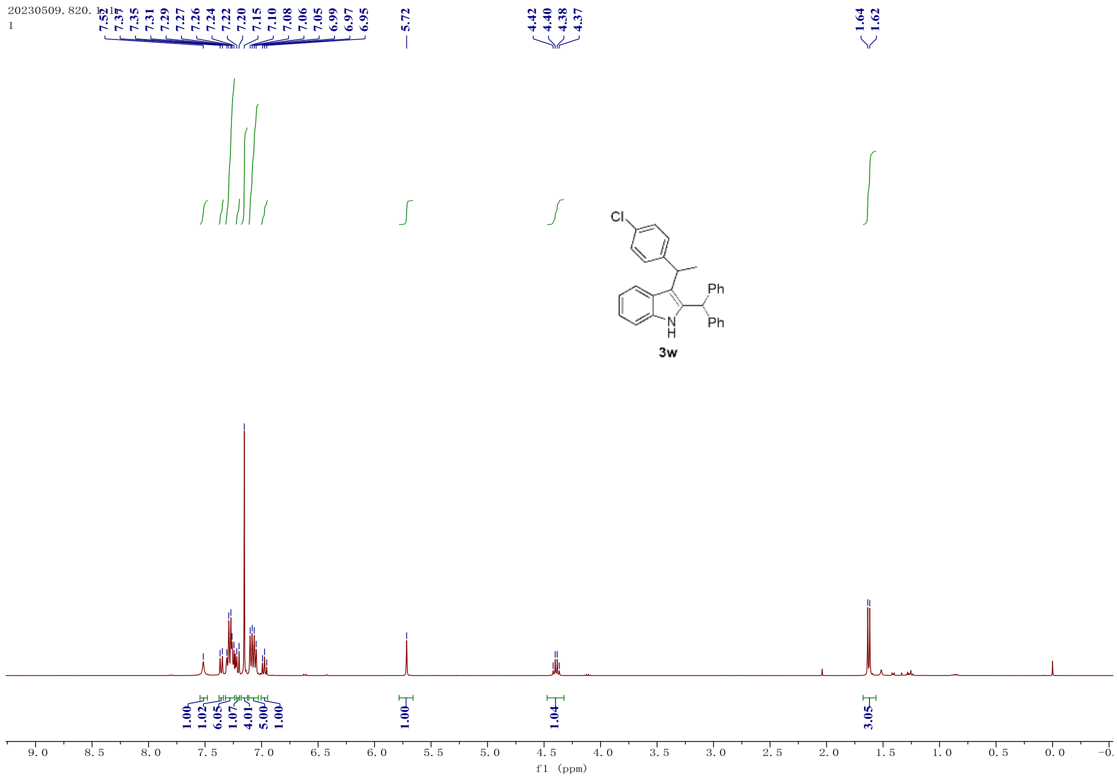
20230509.820.
1
7.52
7.37
7.35
7.31
7.29
7.27
7.26
7.24
7.22
7.20
7.15
7.10
7.08
7.06
7.05
6.99
6.97
6.95
5.72
4.42
4.40
4.38
4.37
1.64
1.62
Cl
Ph
Ph
N
H
3w
1.00
1.02
6.05
1.07
4.01
5.00
1.00
1.00
1.04
3.05
f1 (ppm)

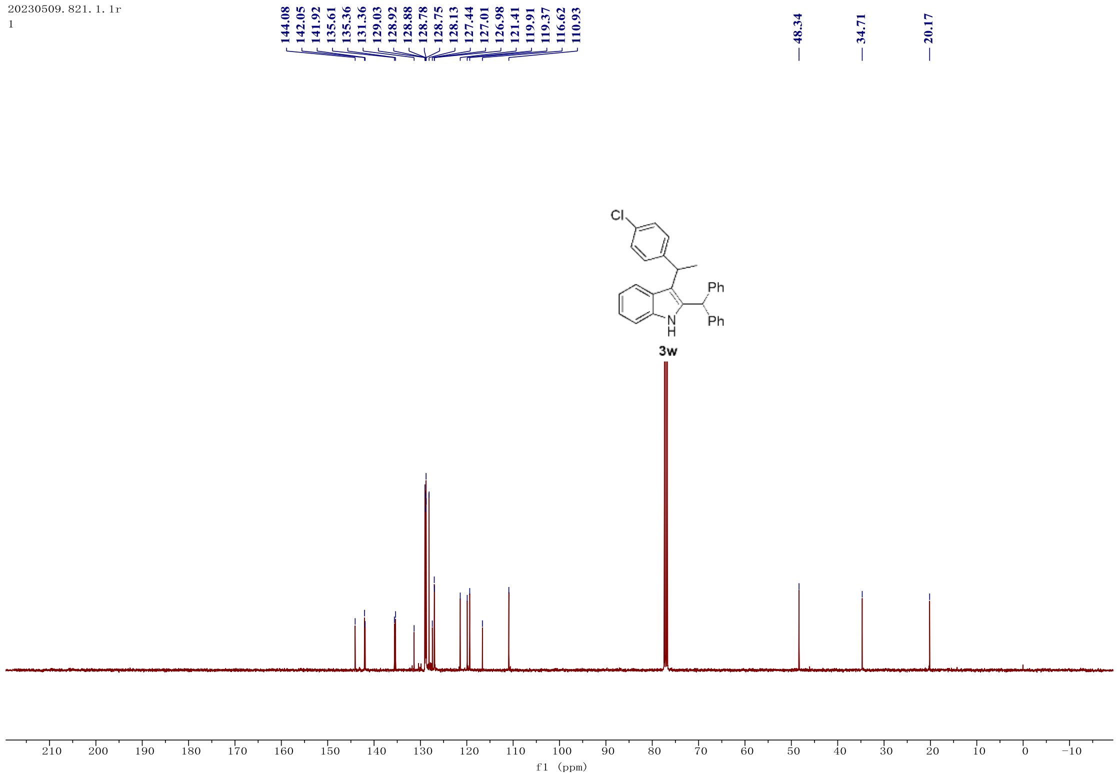
20230509.821.1.1r
1
144.08
142.05
141.92
135.61
135.36
131.36
129.03
128.92
128.88
128.78
128.75
128.13
127.44
127.01
126.98
121.41
119.91
119.37
116.62
110.93
48.34
34.71
20.17
Cl
Ph
Ph
N
H
3w
f1 (ppm)

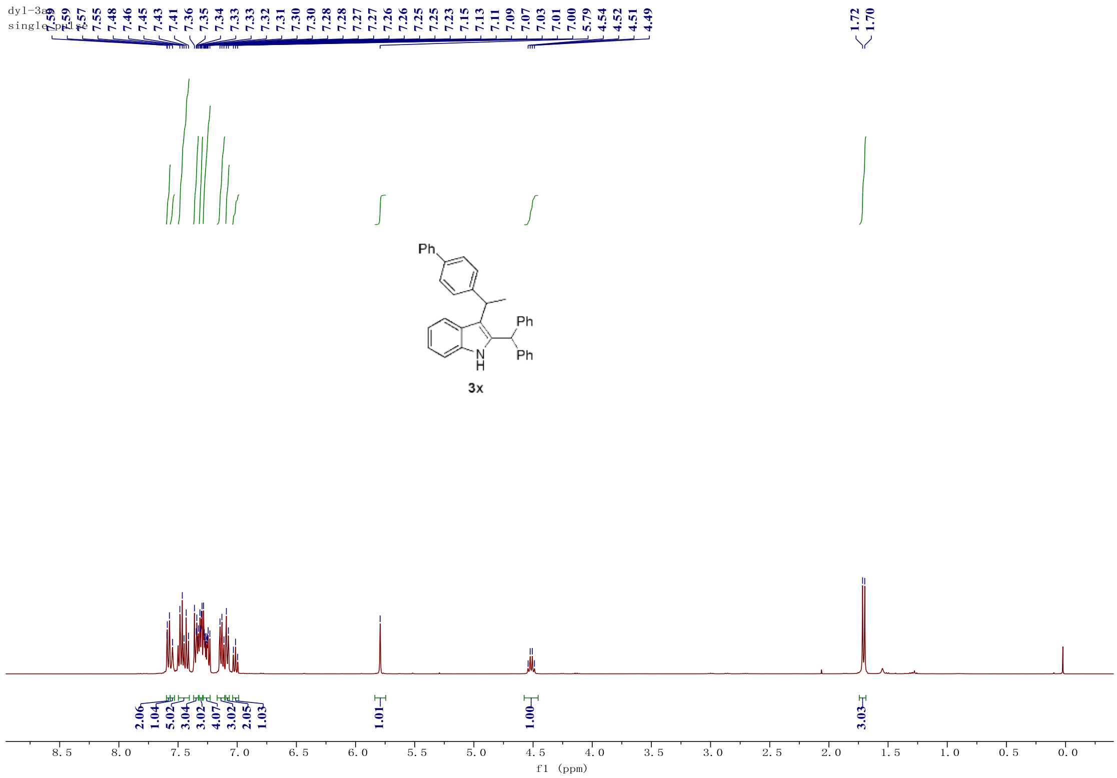
7.59
7.59
7.57
7.55
7.48
7.46
7.45
7.43
7.41
7.36
7.35
7.34
7.33
7.33
7.32
7.31
7.30
7.28
7.28
7.27
7.27
7.26
7.26
7.25
7.25
7.23
7.15
7.13
7.11
7.09
7.07
7.03
7.01
7.00
5.79
4.54
4.52
4.51
4.49
1.72
1.70
Ph
Ph
Ph
N
H
3x
2.06
1.04
5.02
3.04
3.02
4.07
3.02
2.05
1.03
1.01
1.00
3.03
8.5
8.0
7.5
7.0
6.5
6.0
5.5
5.0
4.5
4.0
3.5
3.0
2.5
2.0
1.5
1.0
0.5
0.0
f1 (ppm)

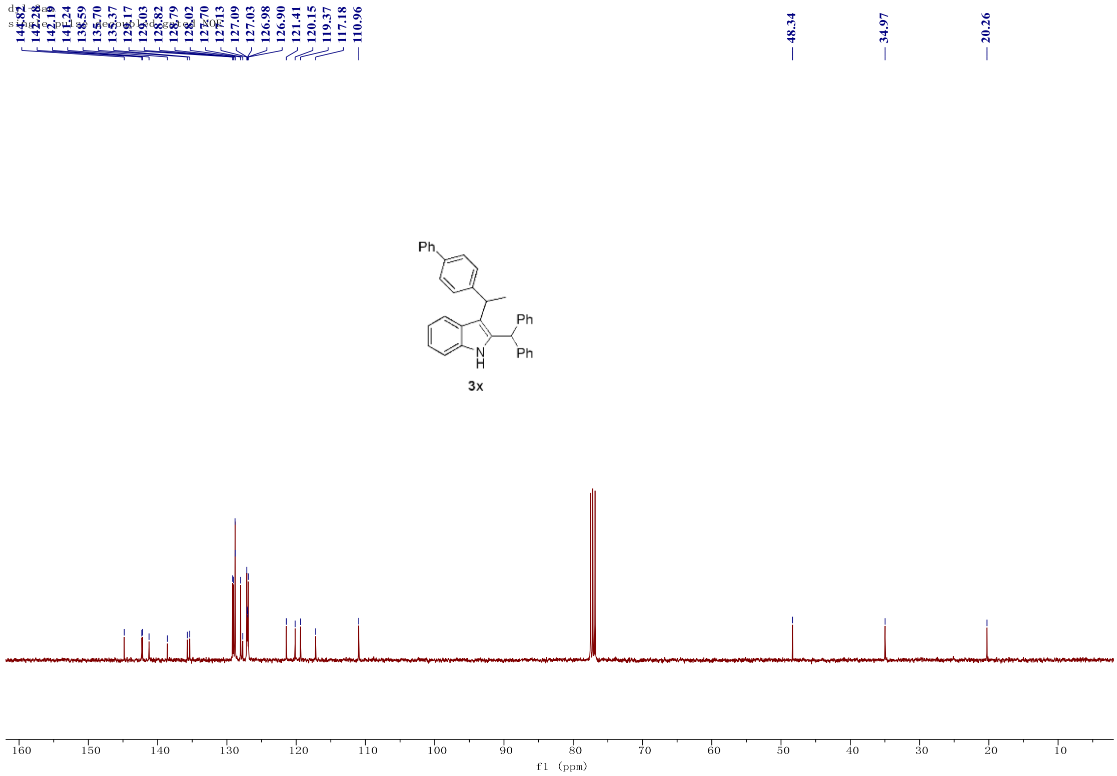
144.82
142.28
142.19
141.24
138.59
135.70
135.37
129.17
129.03
128.82
128.79
128.02
127.70
127.13
127.09
127.03
126.98
126.90
121.41
120.15
119.37
117.18
110.96
48.34
34.97
20.26
Ph
Ph
Ph
N
H
3x
160
150
140
130
120
110
100
90
80
70
60
50
40
30
20
10
f1 (ppm)

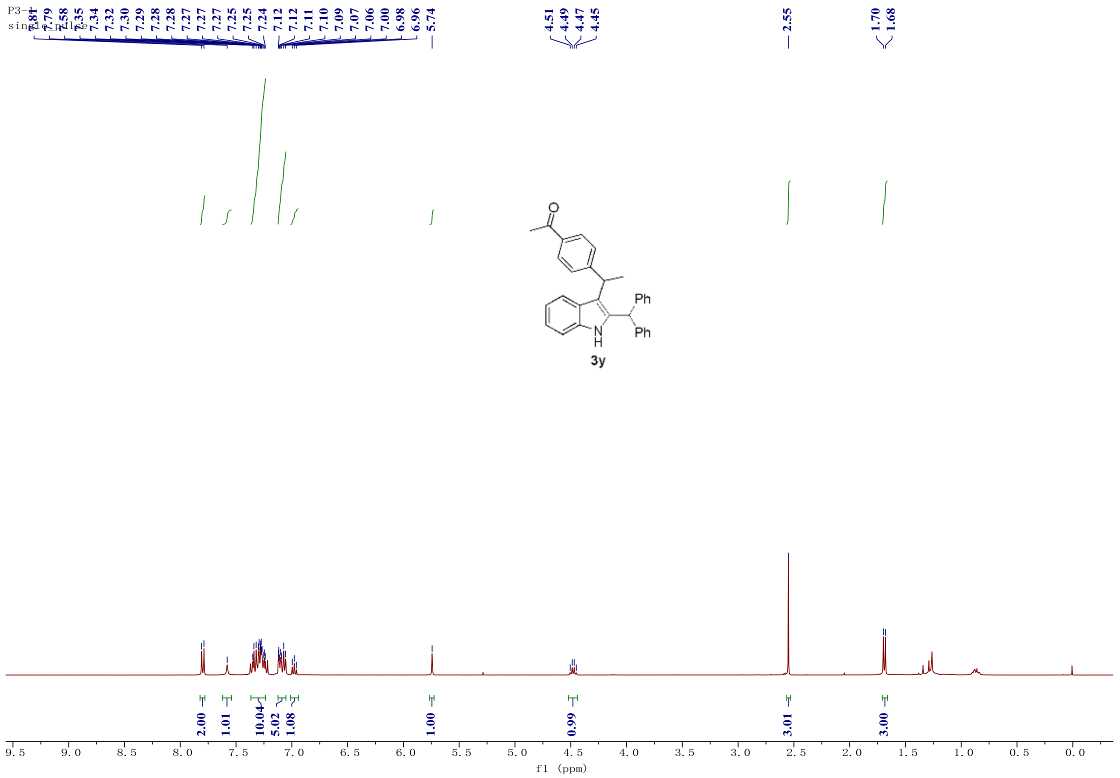
P3-1
single pulse
7.81
7.79
7.58
7.35
7.34
7.32
7.30
7.29
7.28
7.28
7.27
7.27
7.27
7.25
7.25
7.24
7.12
7.12
7.11
7.10
7.09
7.07
7.06
7.00
6.98
6.96
5.74
4.51
4.49
4.47
4.45
2.55
1.70
1.68
Ph
Ph
N
H
3y
2.00
1.01
10.04
5.02
1.08
1.00
0.99
3.01
3.00
9.5 9.0 8.5 8.0 7.5 7.0 6.5 6.0 5.5 5.0 4.5 4.0 3.5 3.0 2.5 2.0 1.5 1.0 0.5 0.0
f1 (ppm)

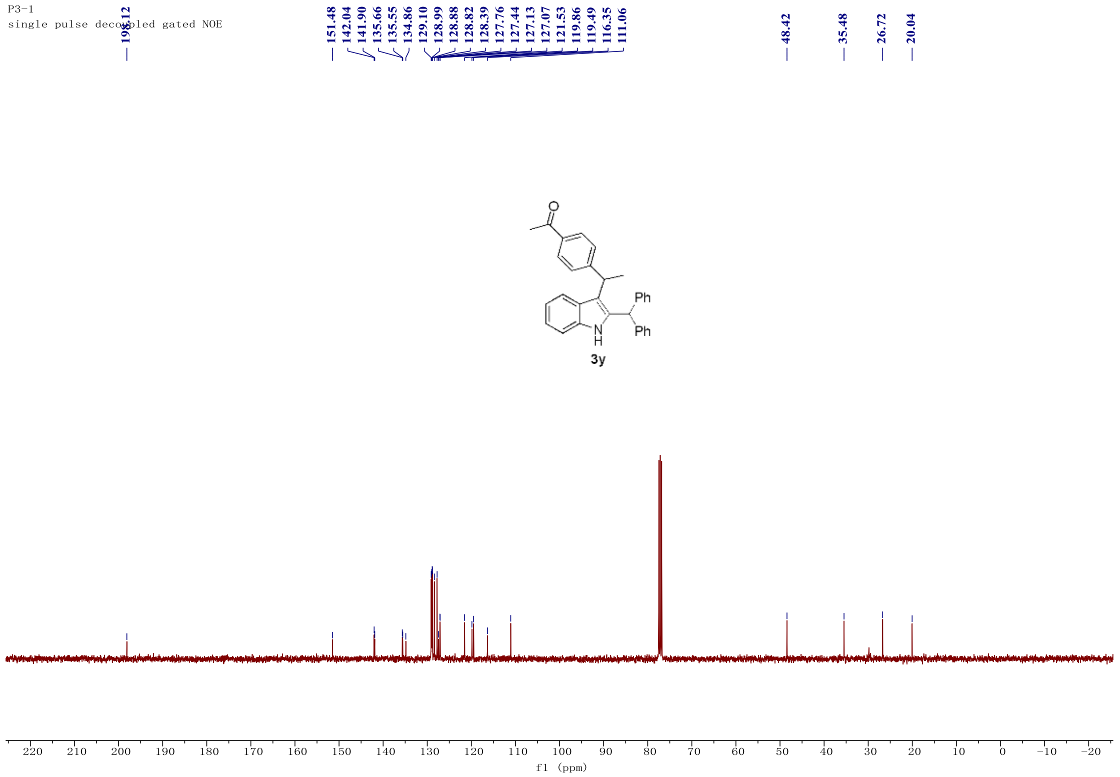
P3-1
single pulse decoupled gated NOE
198.12
151.48
142.04
141.90
135.66
135.55
134.86
129.10
128.99
128.88
128.82
128.39
127.76
127.44
127.13
127.07
121.53
119.86
119.49
116.35
111.06
48.42
35.48
26.72
20.04
Ph
Ph
N
H
3y
220 210 200 190 180 170 160 150 140 130 120 110 100 90 80 70 60 50 40 30 20 10 0 -10 -20
f1 (ppm)

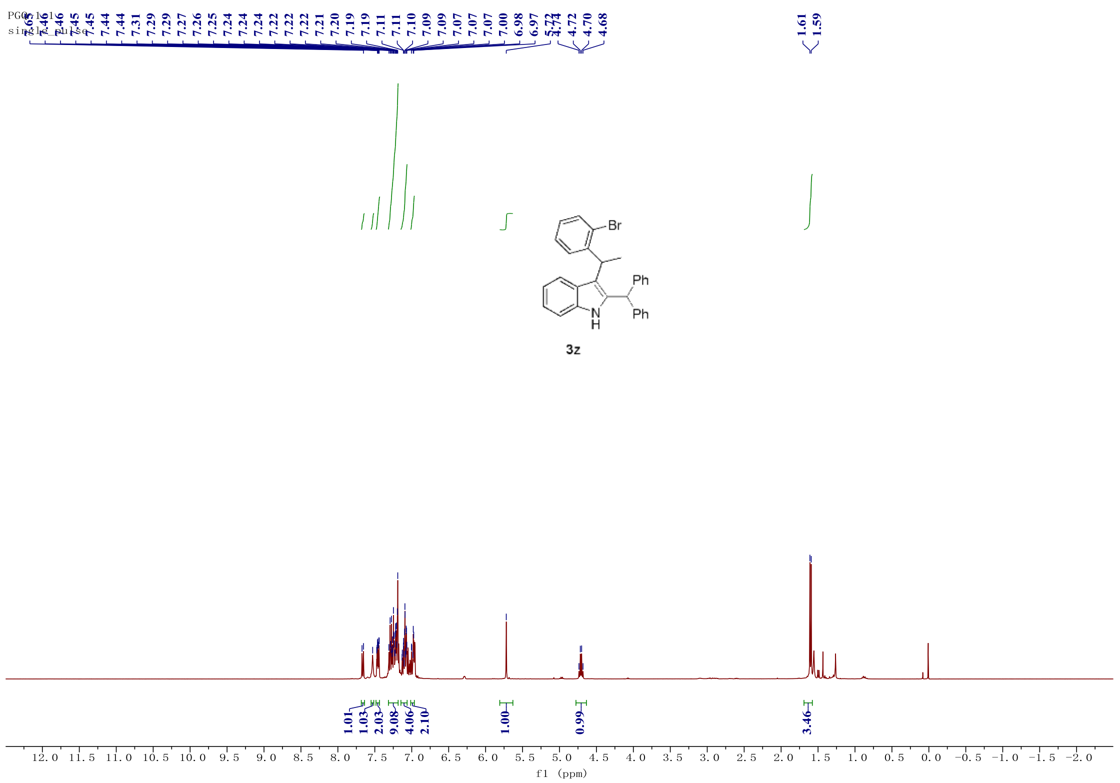

7.46 7.46 7.45 7.45 7.44 7.44 7.31 7.29 7.29 7.27 7.26 7.25 7.24 7.24 7.24 7.22 7.22 7.22 7.21 7.20 7.19 7.19 7.11 7.11 7.10 7.09 7.09 7.07 7.07 7.07 7.00 6.98 6.97
4.72 4.70 4.68
1.61 1.59
Br
Ph
Ph
N
H
3z
1.01 1.03 2.03 9.08 4.06 2.10
1.00
0.99
3.46
12.0 11.5 11.0 10.5 10.0 9.5 9.0 8.5 8.0 7.5 7.0 6.5 6.0 5.5 5.0 4.5 4.0 3.5 3.0 2.5 2.0 1.5 1.0 0.5 0.0 -0.5 -1.0 -1.5 -2.0
f1 (ppm)

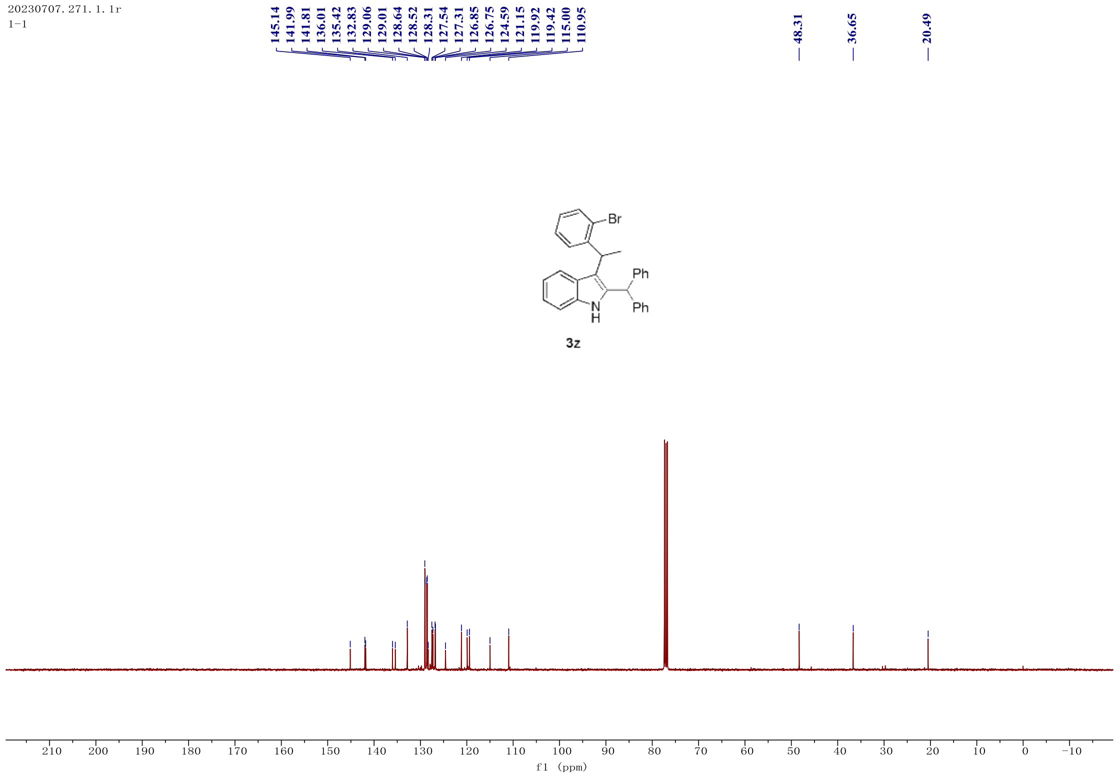

20230707.271.1.1r
1-1
145.14 141.99 141.81 136.01 135.42 132.83 129.06 129.01 128.64 128.52 128.31 127.54 127.31 126.85 126.75 124.59 121.15 119.92 119.42 115.00 110.95
48.31
36.65
20.49
Br
Ph
Ph
N
H
3z
210 200 190 180 170 160 150 140 130 120 110 100 90 80 70 60 50 40 30 20 10 0 -10
f1 (ppm)

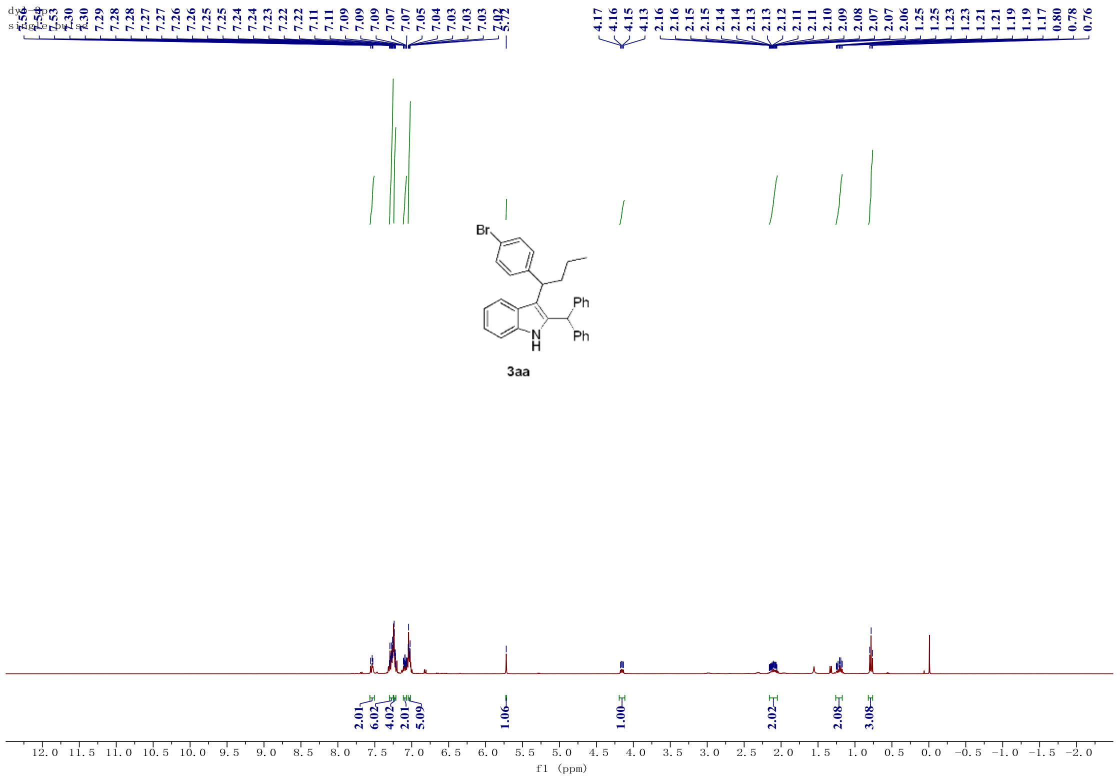
7.56 7.54 7.53 7.30 7.30 7.29 7.28 7.27 7.27 7.26 7.26 7.25 7.24 7.24 7.23 7.22 7.22 7.11 7.11 7.09 7.09 7.07 7.07 7.05 7.04 7.03 7.03 7.03 7.02 5.72
4.17 4.16 4.15 4.13 2.16 2.16 2.15 2.15 2.14 2.14 2.13 2.13 2.12 2.11 2.11 2.10 2.09 2.08 2.07 2.07 2.06 1.25 1.25 1.23 1.23 1.21 1.21 1.19 1.19 1.17 0.80 0.78 0.76
Br
Ph
Ph
N
H
3aa
2.01 6.02 4.02 2.01 5.09 1.06 1.00 2.02 2.08 3.08
12.0 11.5 11.0 10.5 10.0 9.5 9.0 8.5 8.0 7.5 7.0 6.5 6.0 5.5 5.0 4.5 4.0 3.5 3.0 2.5 2.0 1.5 1.0 0.5 0.0 −0.5 −1.0 −1.5 −2.0
f1 (ppm)

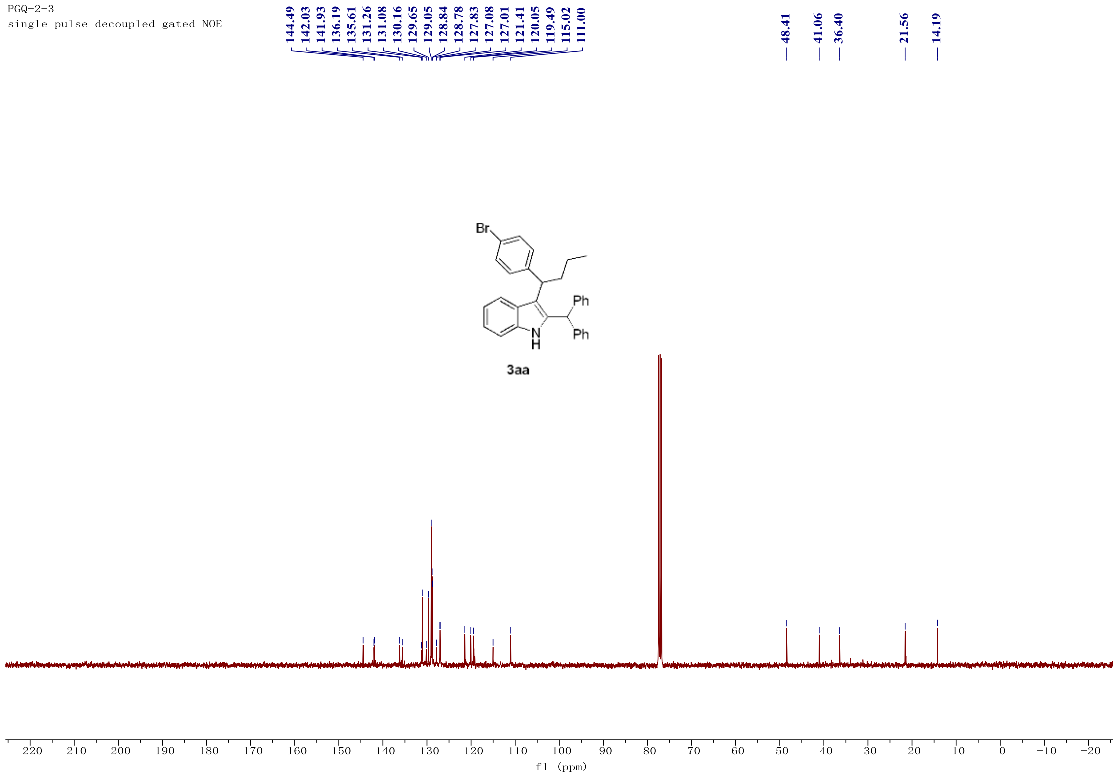
PGQ-2-3
single pulse decoupled gated NOE
144.49 142.03 141.93 136.19 135.61 131.26 131.08 130.16 129.65 129.05 128.84 128.78 127.83 127.08 127.01 121.41 120.05 119.49 115.02 111.00
48.41 41.06 36.40 21.56 14.19
Br
Ph
Ph
N
H
3aa
220 210 200 190 180 170 160 150 140 130 120 110 100 90 80 70 60 50 40 30 20 10 0 −10 −20
f1 (ppm)

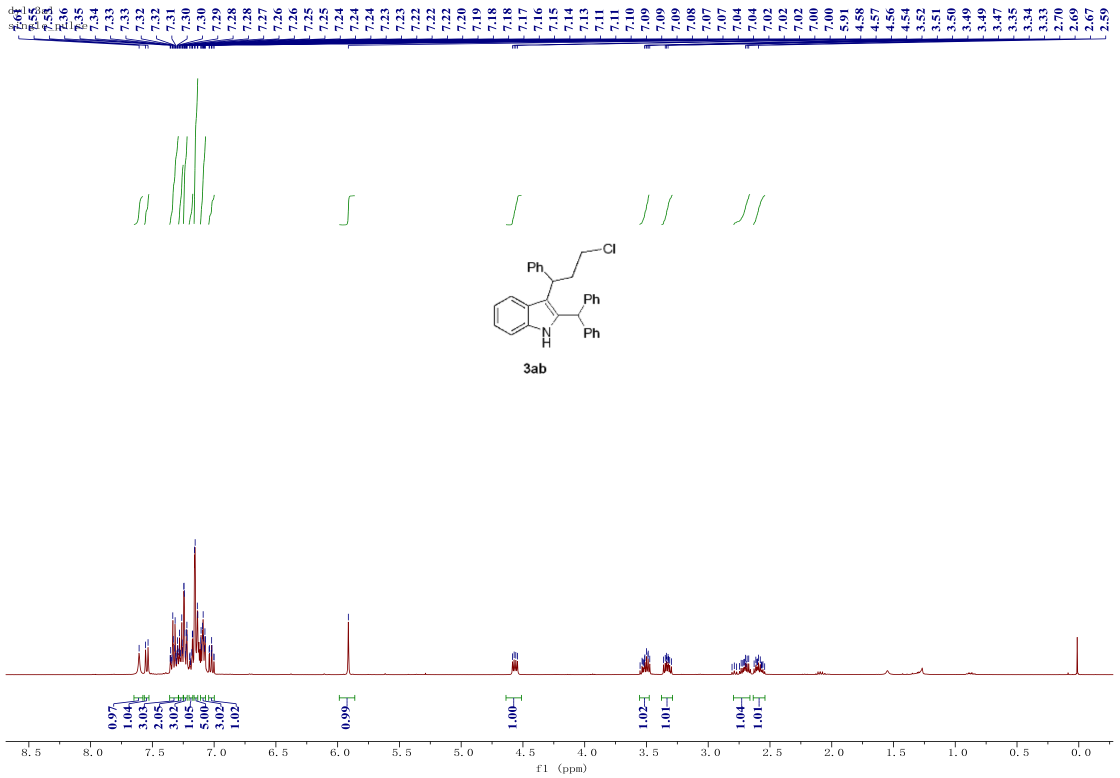
[illegible]
7.61 7.55 7.53 7.36 7.35 7.34 7.33 7.33 7.32 7.32 7.31 7.30 7.29 7.28 7.28 7.27 7.26 7.26 7.25 7.25 7.24 7.24 7.24 7.23 7.23 7.22 7.22 7.20 7.19 7.18 7.18 7.17 7.16 7.15 7.14 7.13 7.11 7.11 7.10 7.09 7.09 7.09 7.08 7.07 7.07 7.04 7.04 7.02 7.02 7.02 7.00 5.91 4.58 4.57 4.56 4.54 3.52 3.51 3.50 3.49 3.49 3.47 3.35 3.34 3.33 2.70 2.69 2.67 2.59
Ph
Cl
Ph
Ph
N
H
3ab
0.97 1.04 3.03 2.05 3.02 1.05 5.00 3.02 1.02 0.99 1.00 1.02 1.01 1.04 1.01
8.5 8.0 7.5 7.0 6.5 6.0 5.5 5.0 4.5 4.0 3.5 3.0 2.5 2.0 1.5 1.0 0.5 0.0
f1 (ppm)

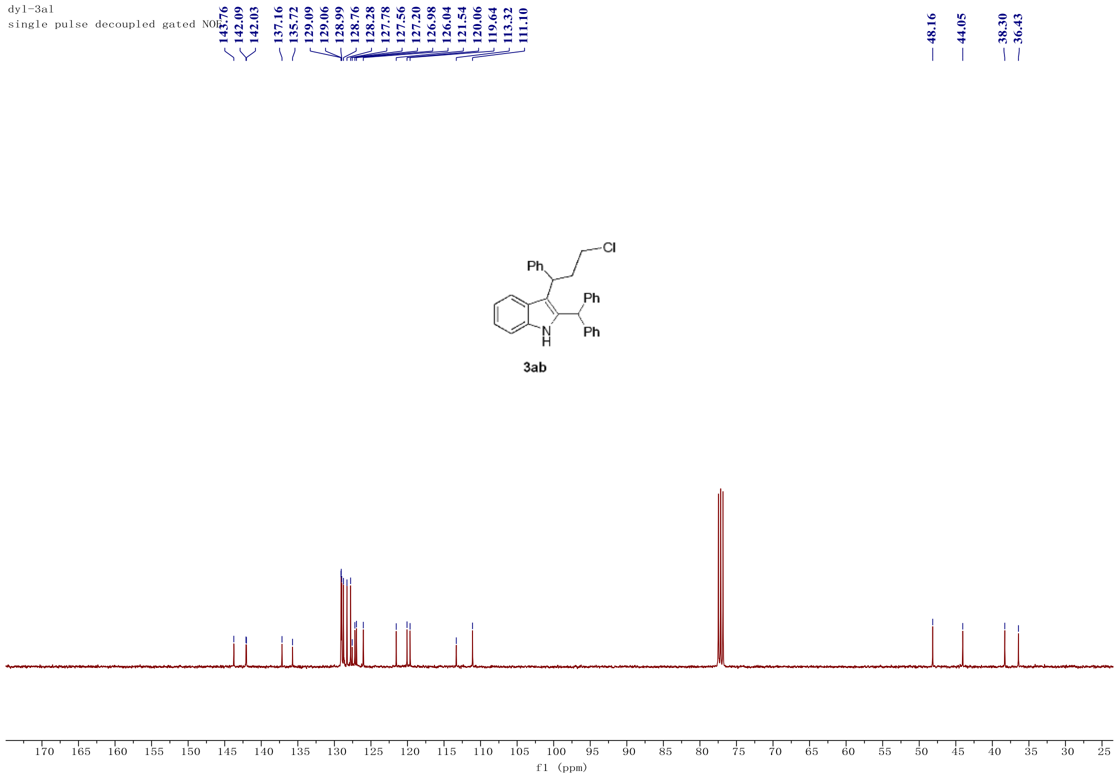
dyl-3al
single pulse decoupled gated NOE
143.76 142.09 142.03 137.16 135.72 129.09 129.06 128.99 128.76 128.28 127.78 127.56 127.20 126.98 126.04 121.54 120.06 119.64 113.32 111.10 48.16 44.05 38.30 36.43
Ph
Cl
Ph
Ph
N
H
3ab
170 165 160 155 150 145 140 135 130 125 120 115 110 105 100 95 90 85 80 75 70 65 60 55 50 45 40 35 30 25
f1 (ppm)

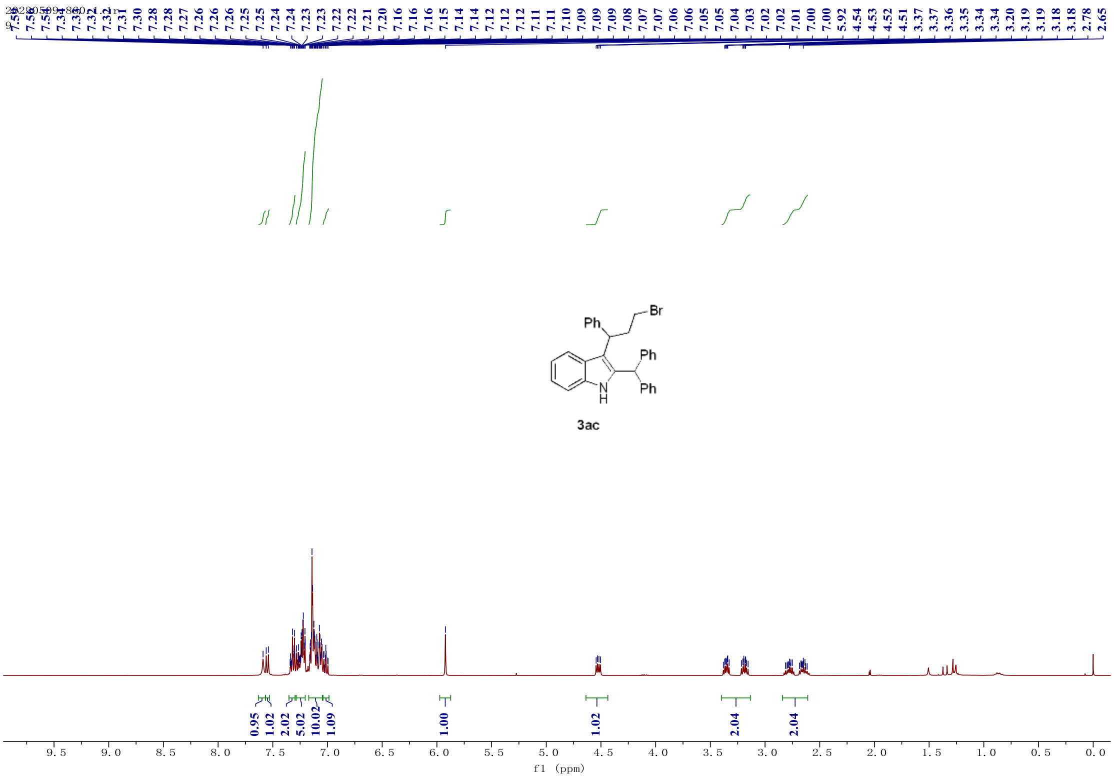
7.56 7.54 7.34 7.33 7.32 7.32 7.31 7.30 7.28 7.28 7.27 7.26 7.26 7.25 7.25 7.24 7.24 7.23 7.23 7.22 7.22 7.21 7.20 7.16 7.16 7.15 7.14 7.14 7.12 7.12 7.12 7.11 7.11 7.10 7.09 7.09 7.09 7.08 7.07 7.07 7.06 7.06 7.05 7.05 7.04 7.04 7.03 7.02 7.02 7.01 7.00 5.92 4.54 4.53 4.52 4.51 3.37 3.37 3.36 3.35 3.34 3.34 3.20 3.19 3.19 3.18 2.78 2.65
Ph
Br
Ph
Ph
N
H
3ac
0.95 1.02 2.02 5.02 10.02 1.09 1.00 1.02 2.04 2.04
9.5 9.0 8.5 8.0 7.5 7.0 6.5 6.0 5.5 5.0 4.5 4.0 3.5 3.0 2.5 2.0 1.5 1.0 0.5 0.0
f1 (ppm)

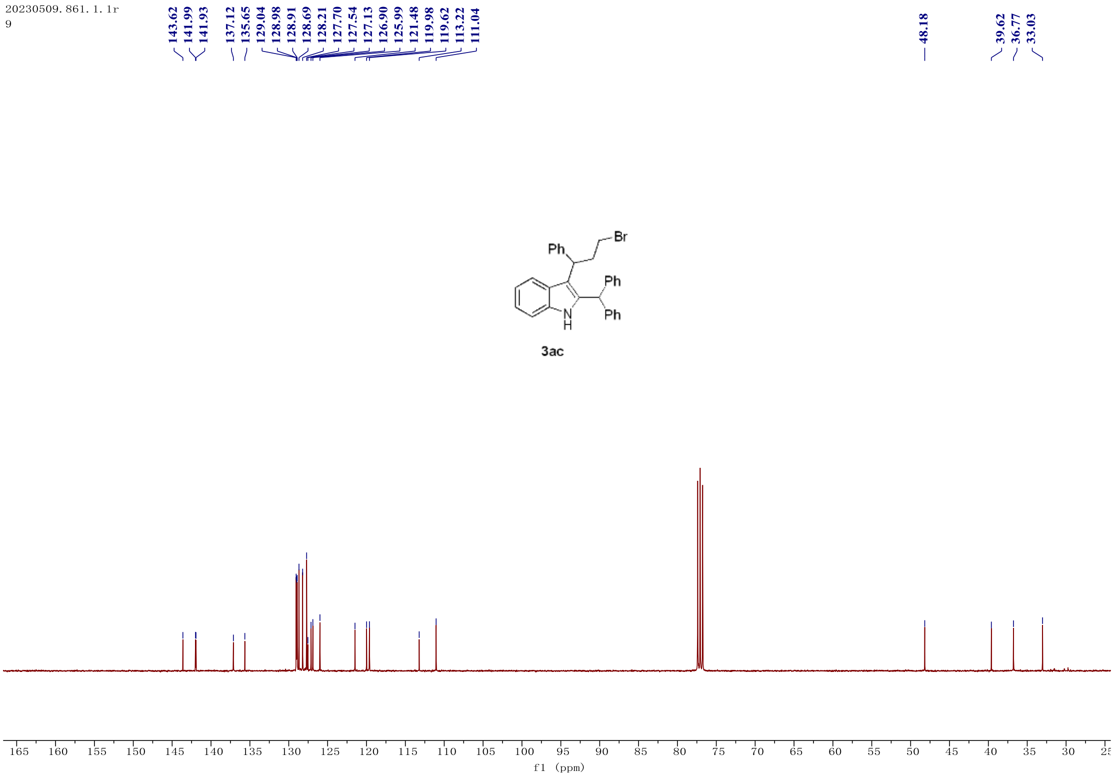
20230509.861.1.1r
9
143.62 141.99 141.93 137.12 135.65 129.04 128.98 128.91 128.69 128.21 127.70 127.54 127.13 126.90 125.99 121.48 119.98 119.62 113.22 111.04
48.18
39.62 36.77 33.03
Ph
Br
Ph
Ph
N
H
3ac
165 160 155 150 145 140 135 130 125 120 115 110 105 100 95 90 85 80 75 70 65 60 55 50 45 40 35 30
f1 (ppm)

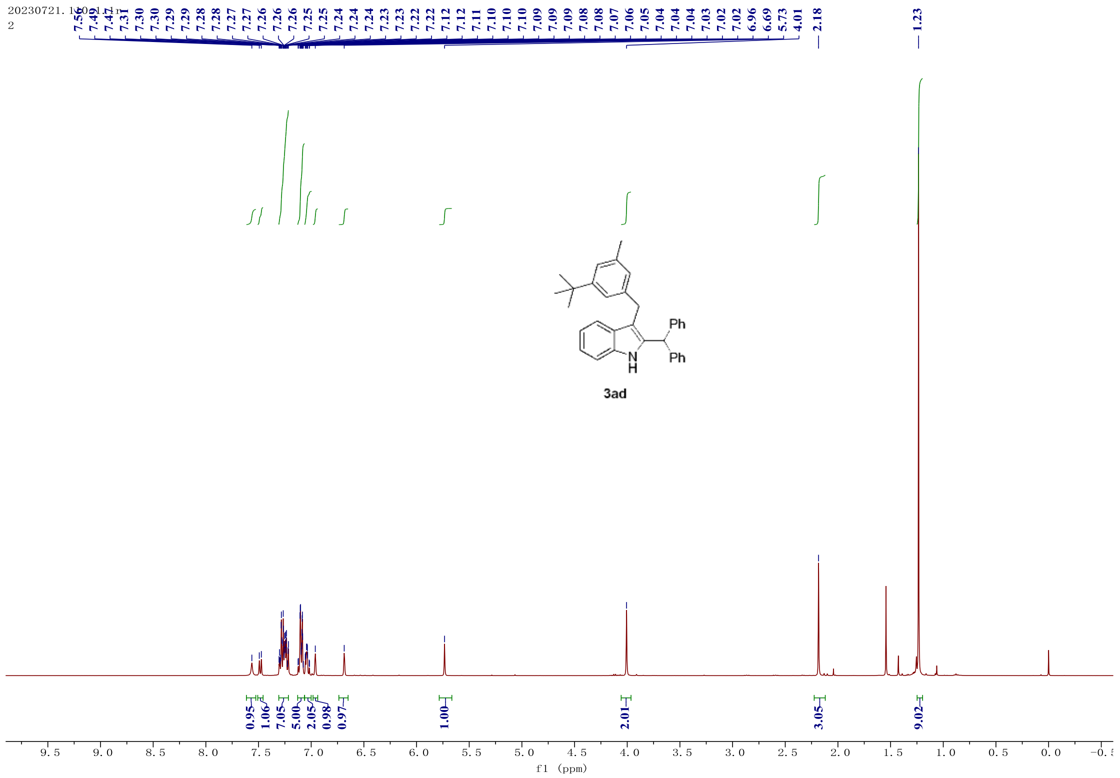
20230721.110.1.1r
2
7.56
7.49
7.47
7.31
7.30
7.30
7.29
7.29
7.28
7.28
7.27
7.27
7.26
7.26
7.26
7.25
7.25
7.24
7.24
7.24
7.23
7.23
7.22
7.22
7.12
7.12
7.11
7.10
7.10
7.10
7.09
7.09
7.09
7.08
7.08
7.07
7.06
7.05
7.04
7.04
7.04
7.03
7.02
7.02
6.96
6.69
5.73
4.01
2.18
1.23
Ph
Ph
N
H
3ad
0.95
1.06
7.05
5.00
2.05
0.98
0.97
1.00
2.01
3.05
9.02
f1 (ppm)

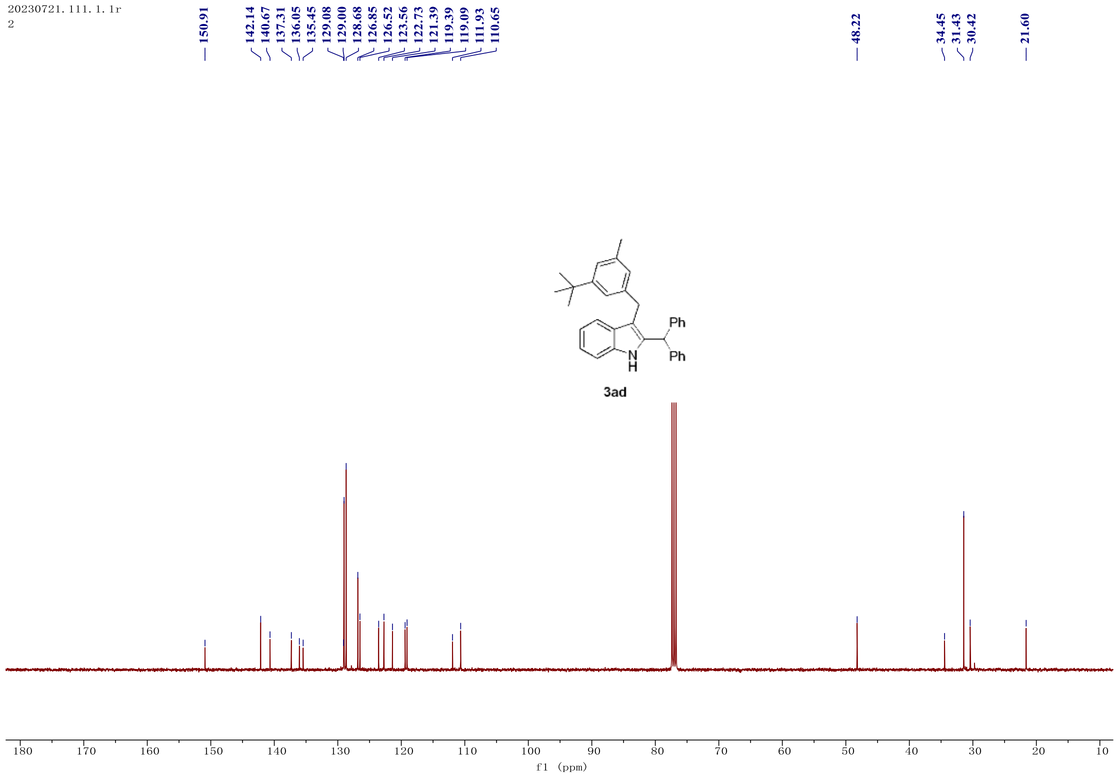
20230721.111.1.1r
2
150.91
142.14
140.67
137.31
136.05
135.45
129.08
129.00
128.68
126.85
126.52
123.56
122.73
121.39
119.39
119.09
111.93
110.65
48.22
34.45
31.43
30.42
21.60
Ph
Ph
N
H
3ad
f1 (ppm)

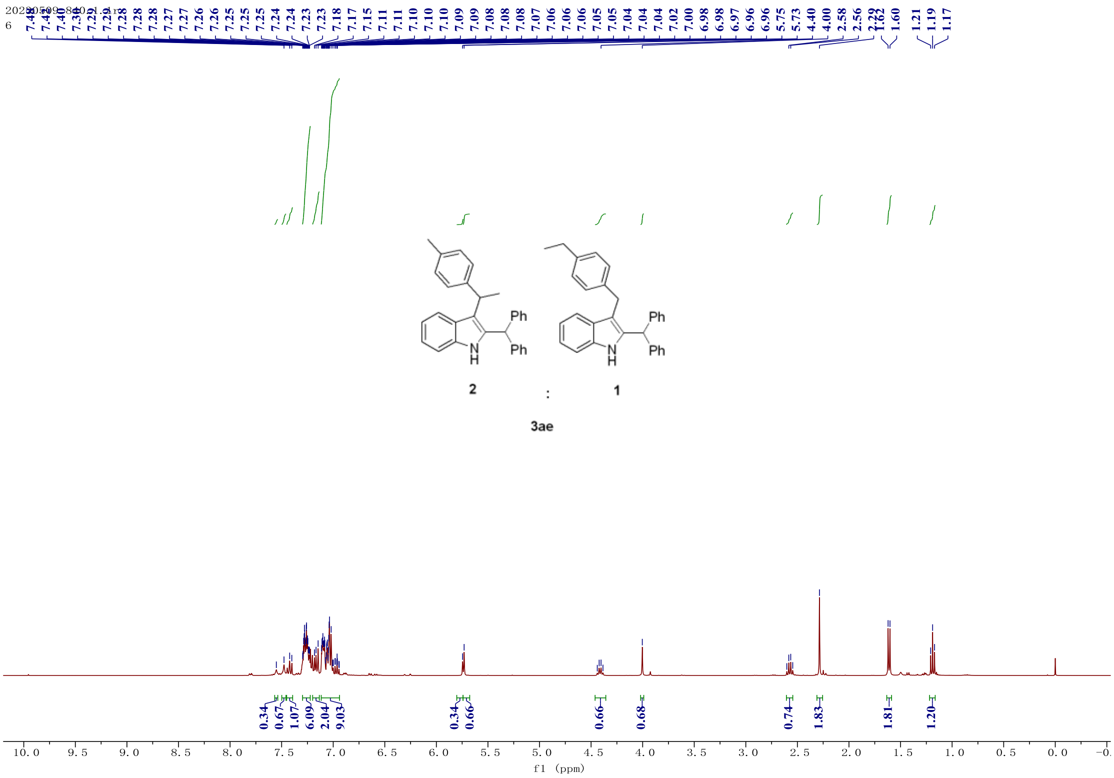
2
:
1
3ae
Ph
Ph
H
f1 (ppm)

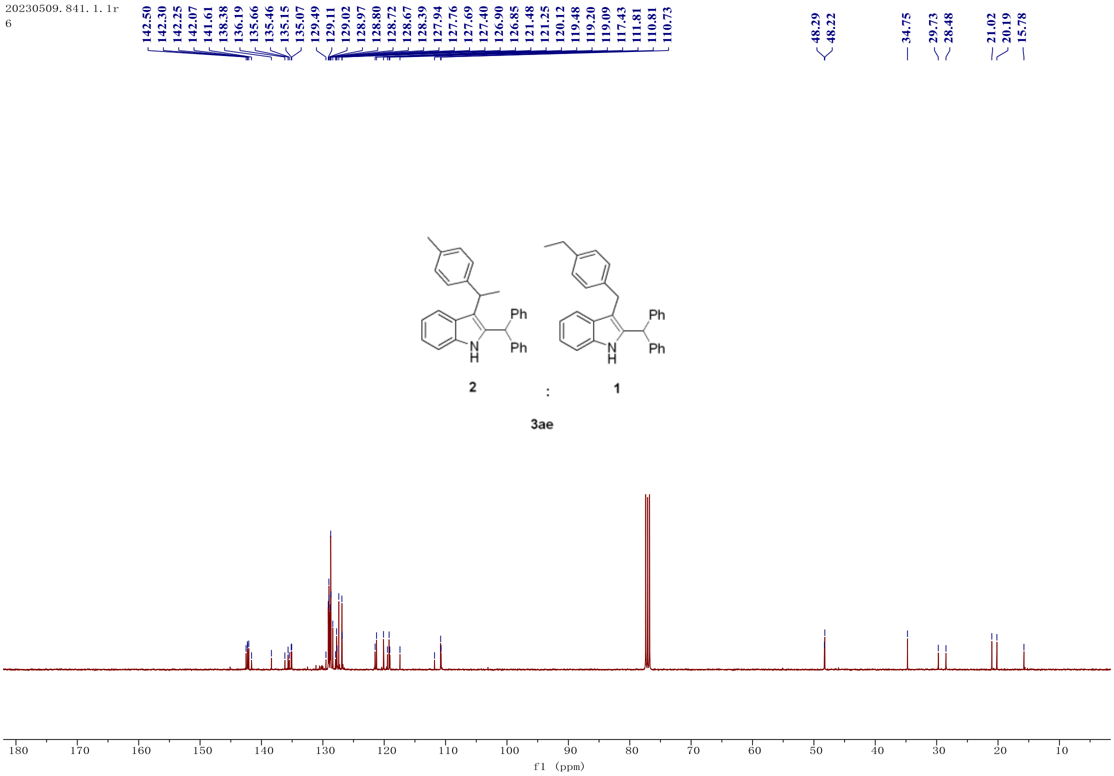
20230509.841.1.1r
142.50
142.30
142.25
142.07
141.61
138.38
136.19
135.66
135.46
135.15
135.07
129.49
129.11
129.02
128.97
128.80
128.72
128.67
128.39
127.94
127.76
127.69
127.40
126.90
126.85
121.48
121.25
120.12
119.48
119.20
119.09
117.43
111.81
110.81
110.73
48.29
48.22
34.75
29.73
28.48
21.02
20.19
15.78
2
:
1
3ae
Ph
Ph
H
f1 (ppm)

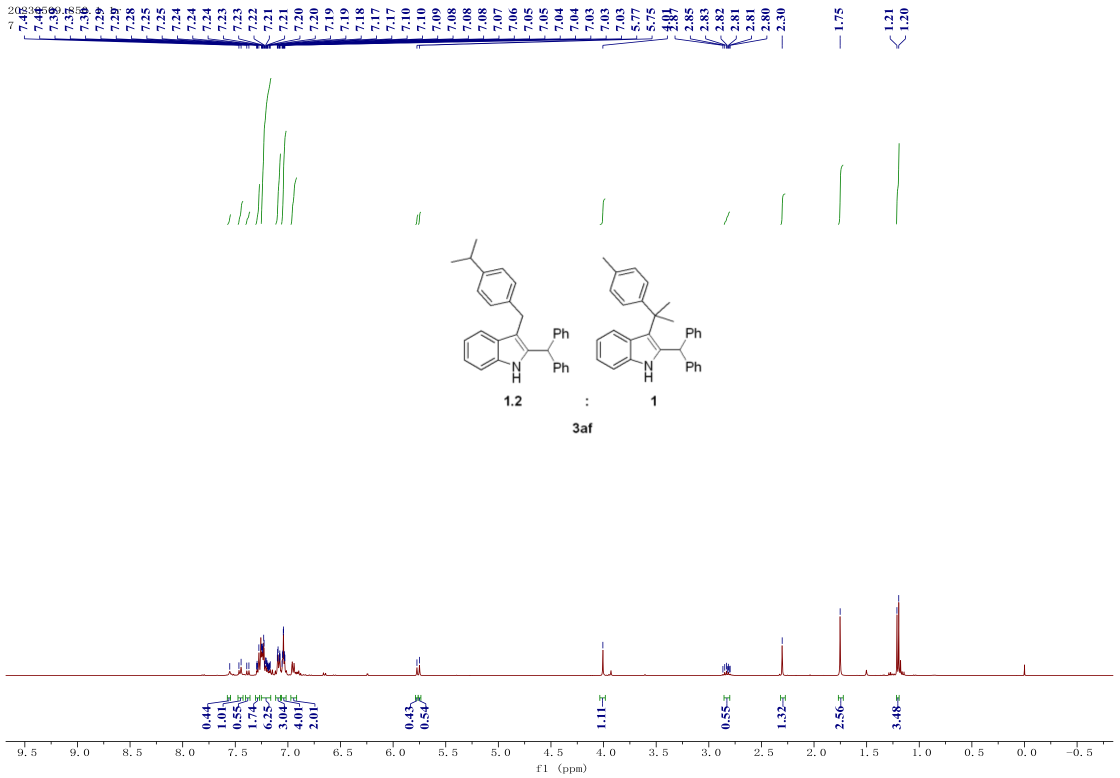
Ph
Ph
1.2
:
1
3af
f1 (ppm)

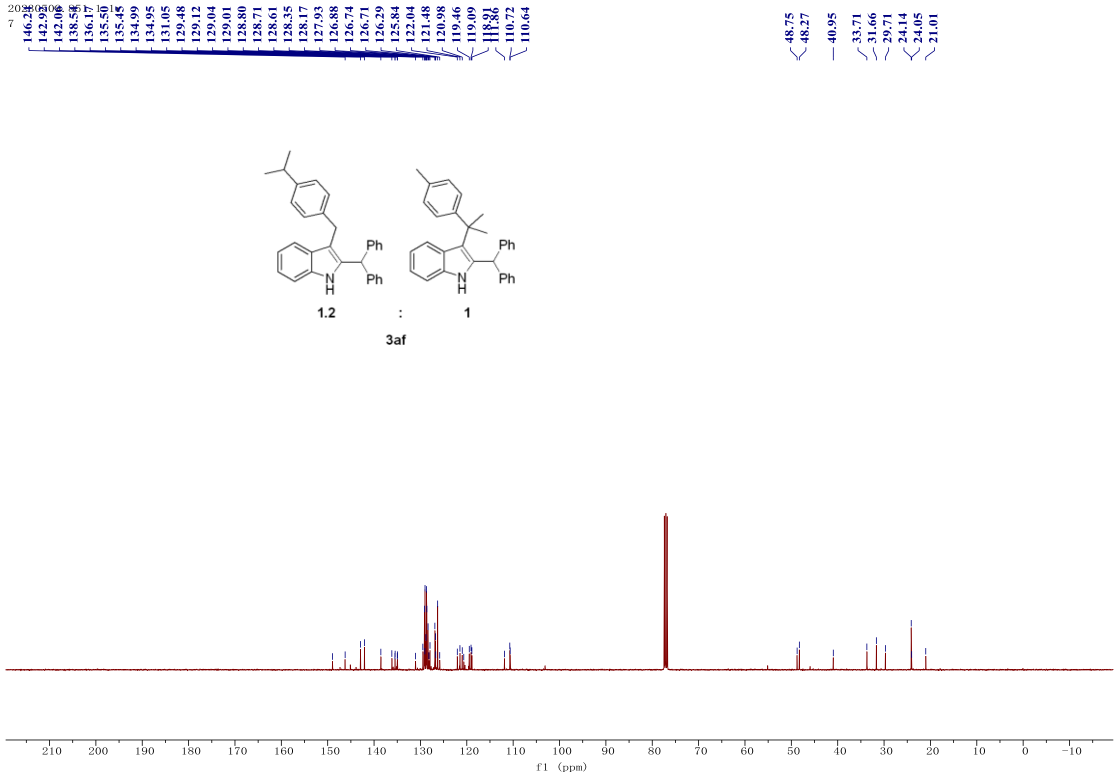
Ph
Ph
1.2
:
1
3af
f1 (ppm)

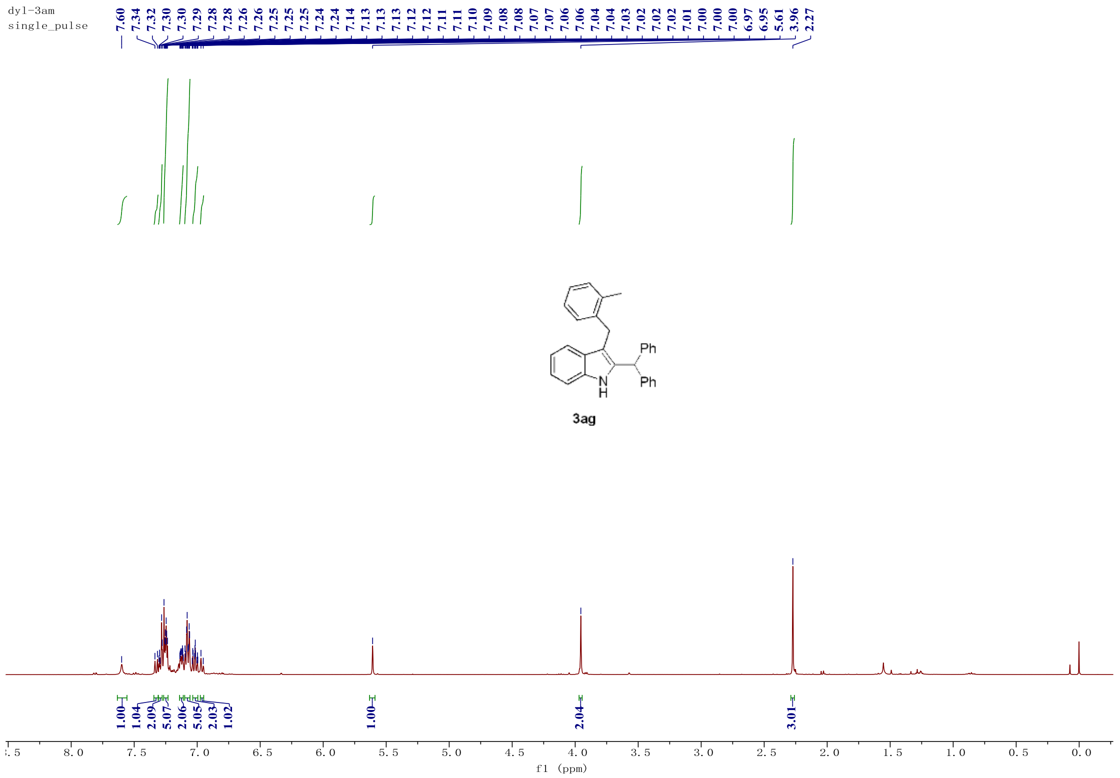
dyl-3am
single_pulse
7.60 7.34 7.32 7.30 7.30 7.29 7.28 7.26 7.26 7.25 7.25 7.24 7.24 7.14 7.13 7.13 7.12 7.12 7.11 7.11 7.10 7.09 7.08 7.08 7.07 7.07 7.06 7.06 7.04 7.04 7.03 7.02 7.02 7.02 7.01 7.00 7.00 6.97 6.95 5.61 3.96 2.27
Ph
Ph
N
H
3ag
1.00 1.04 2.09 5.07 2.06 5.05 2.03 1.02 1.00 2.04 3.01
8.0 7.5 7.0 6.5 6.0 5.5 5.0 4.5 4.0 3.5 3.0 2.5 2.0 1.5 1.0 0.5 0.0
f1 (ppm)

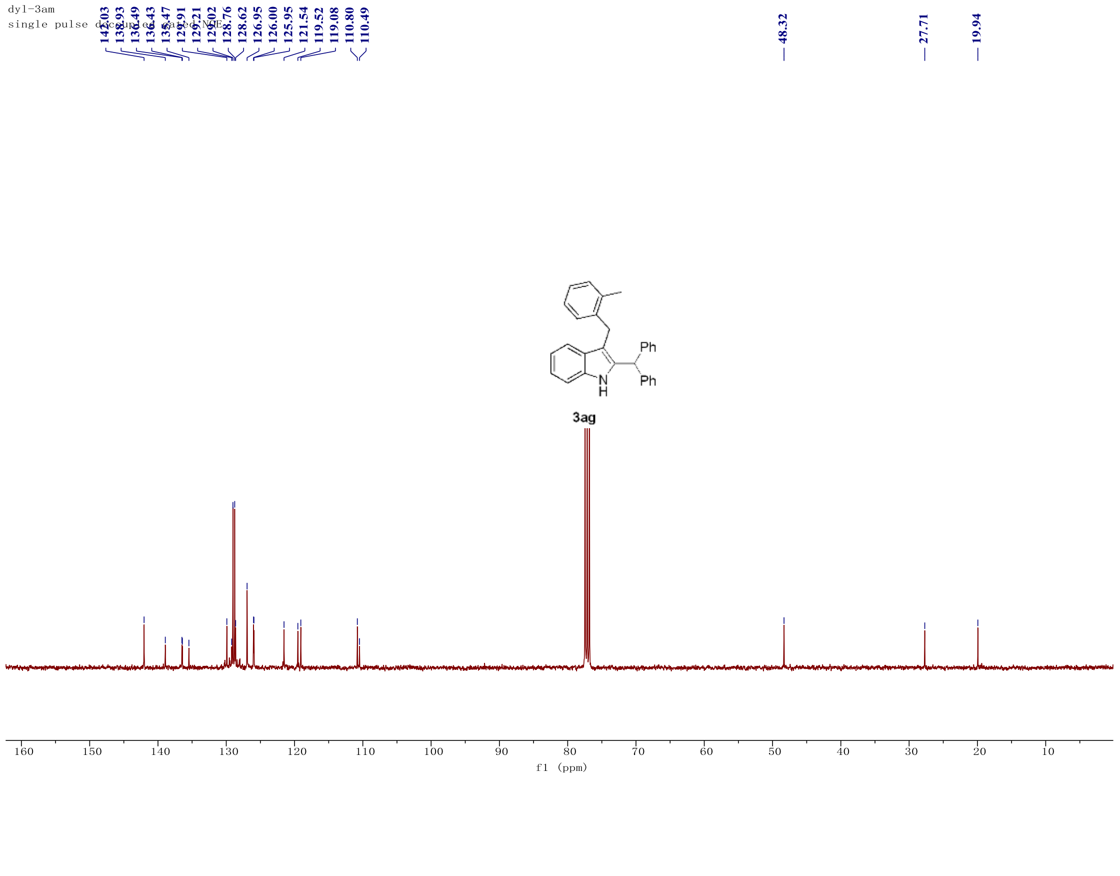
dyl-3am
single pulse
142.03 138.93 136.49 136.43 135.47 129.91 129.21 129.02 128.76 128.62 126.95 126.00 125.95 121.54 119.52 119.08 110.80 110.49
48.32
27.71
19.94
Ph
Ph
N
H
3ag
160 150 140 130 120 110 100 90 80 70 60 50 40 30 20 10
f1 (ppm)

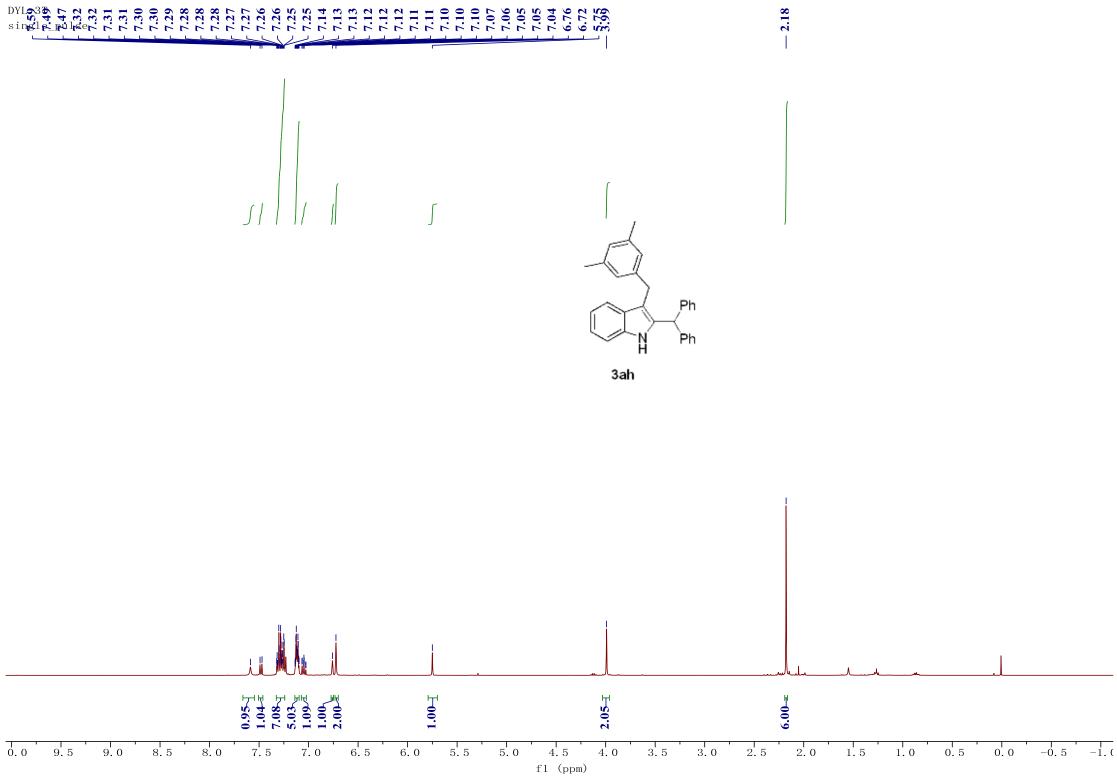
7.49
7.47
7.32
7.31
7.31
7.30
7.29
7.28
7.28
7.27
7.27
7.26
7.26
7.25
7.25
7.14
7.13
7.13
7.12
7.12
7.12
7.11
7.11
7.10
7.10
7.10
7.07
7.06
7.05
7.05
7.04
6.76
6.72
2.18
Ph
Ph
N
H
3ah
0.95
1.04
7.08
5.03
1.09
1.00
2.00
1.00
2.05
6.00
f1 (ppm)

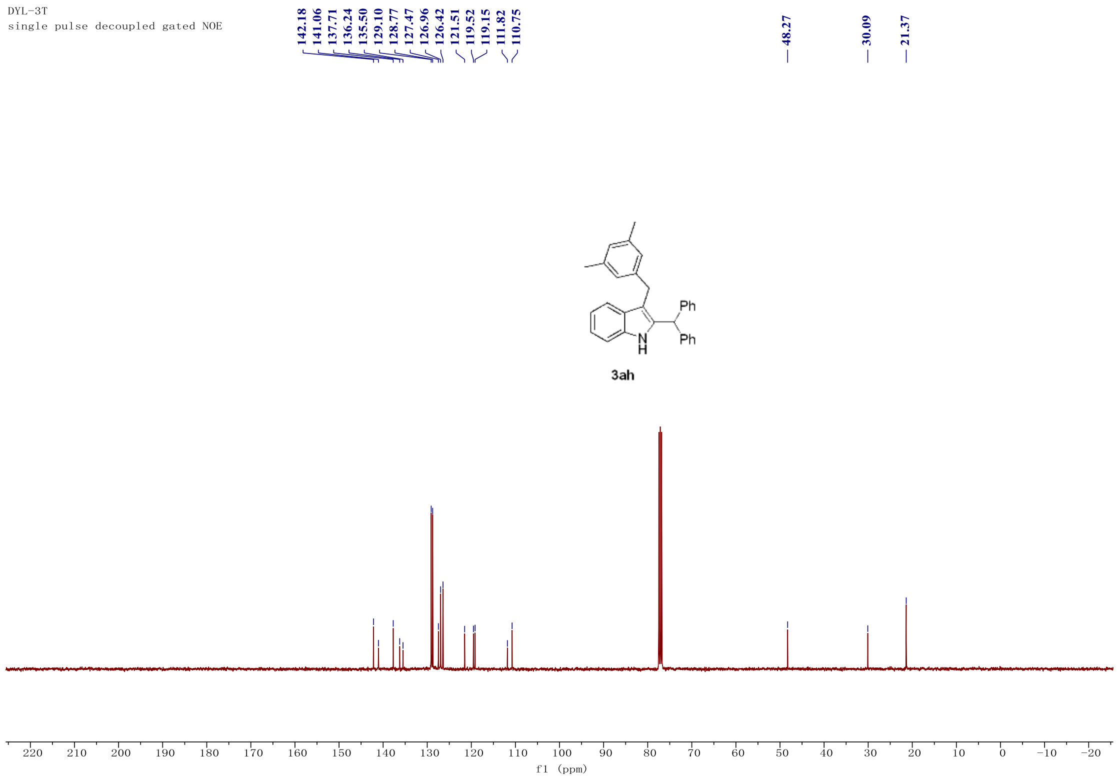
DYL-3T
single pulse decoupled gated NOE
142.18
141.06
137.71
136.24
135.50
129.10
128.77
127.47
126.96
126.42
121.51
119.52
119.15
111.82
110.75
48.27
30.09
21.37
Ph
Ph
N
H
3ah
f1 (ppm)

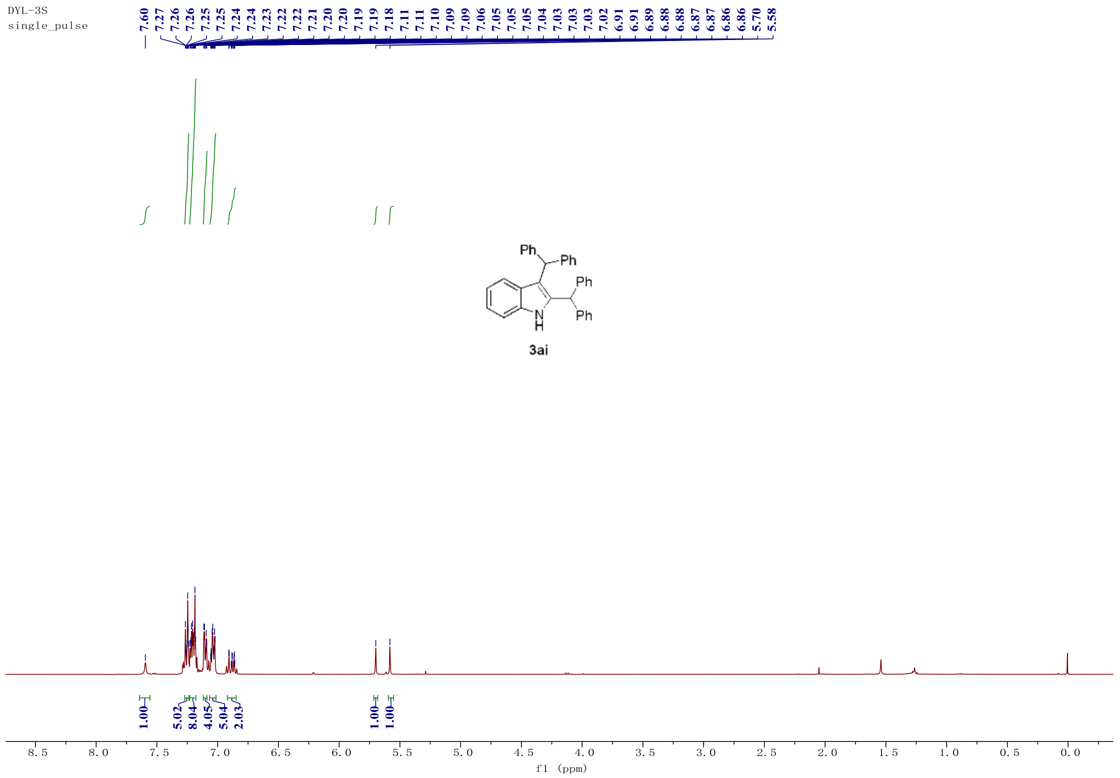
DYL-3S
single_pulse
7.60 7.27 7.26 7.26 7.25 7.25 7.24 7.24 7.23 7.22 7.22 7.21 7.20 7.20 7.19 7.19 7.18 7.11 7.11 7.10 7.09 7.09 7.06 7.05 7.05 7.05 7.04 7.03 7.03 7.03 7.02 6.91 6.89 6.88 6.88 6.87 6.87 6.86 6.86 5.70 5.58
Ph
Ph
Ph
Ph
N
H
3ai
1.00 5.02 8.04 4.05 5.04 2.03 1.00 1.00
8.5 8.0 7.5 7.0 6.5 6.0 5.5 5.0 4.5 4.0 3.5 3.0 2.5 2.0 1.5 1.0 0.5 0.0
f1 (ppm)

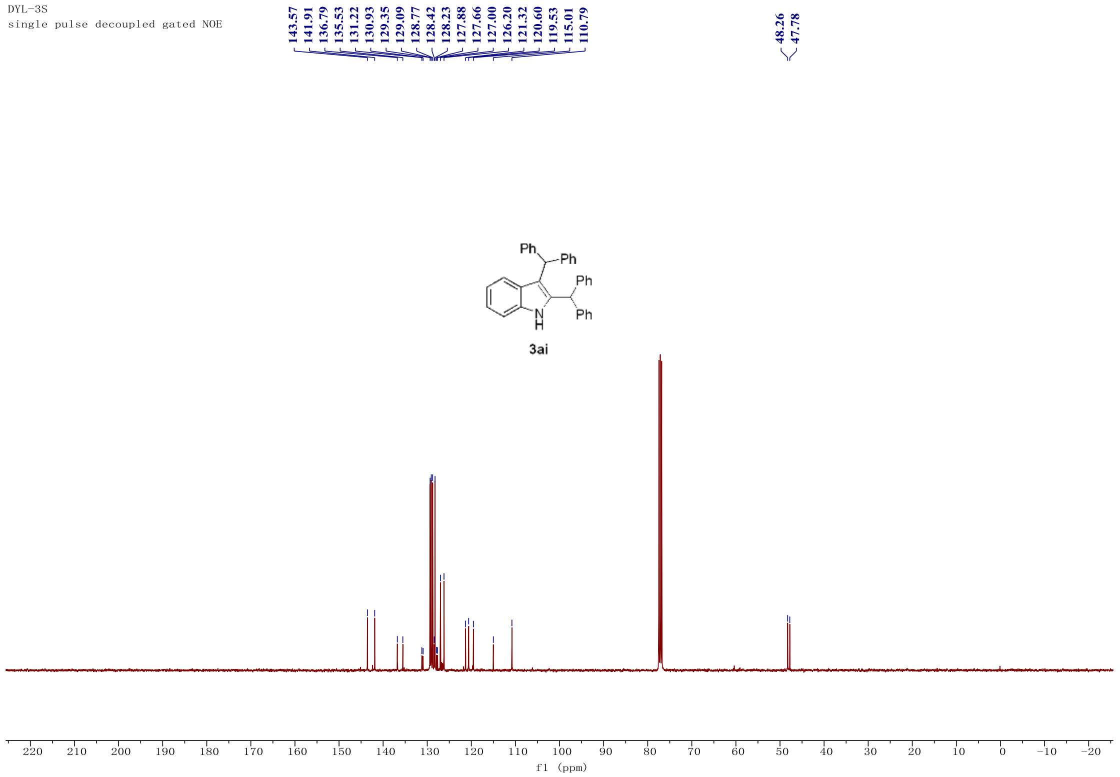
DYL-3S
single pulse decoupled gated NOE
143.57 141.91 136.79 135.53 131.22 130.93 129.35 129.09 128.77 128.42 128.23 127.88 127.66 127.00 126.20 121.32 120.60 119.53 115.01 110.79
48.26 47.78
Ph
Ph
Ph
Ph
N
H
3ai
220 210 200 190 180 170 160 150 140 130 120 110 100 90 80 70 60 50 40 30 20 10 0 -10 -20
f1 (ppm)

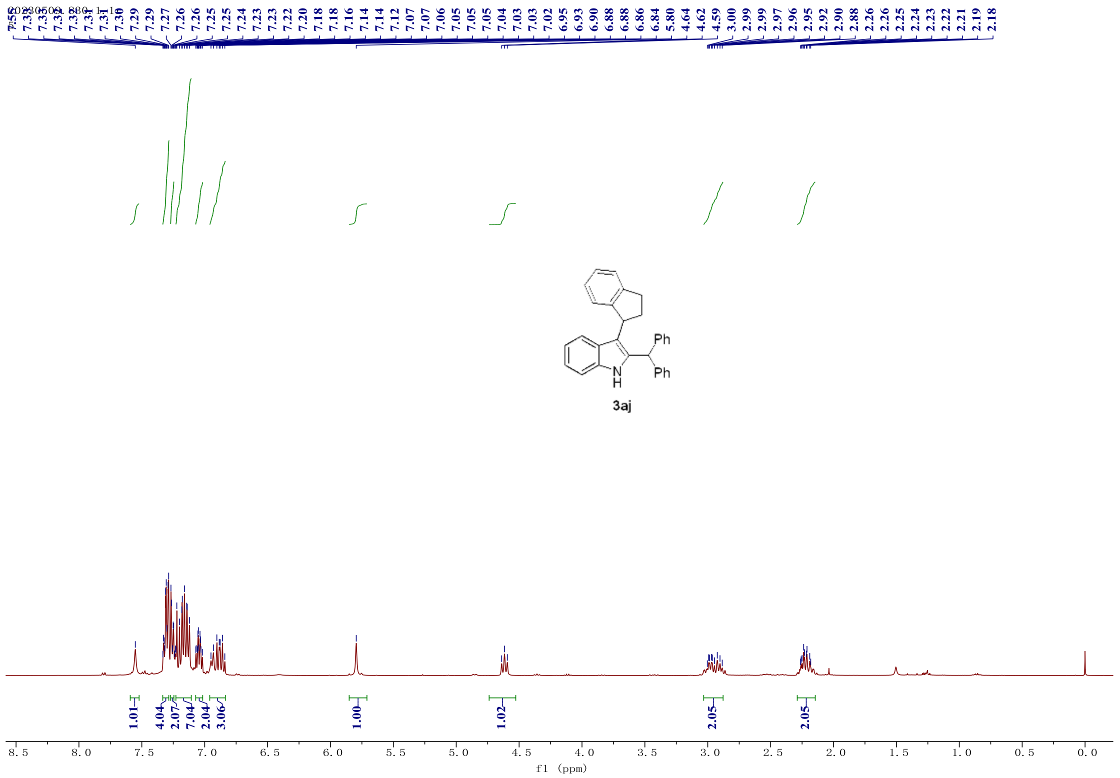

20230509.830.1.1r
7.59 7.33 7.32 7.32 7.31 7.31 7.30 7.29 7.29 7.27 7.26 7.26 7.25 7.25 7.24 7.23 7.23 7.22 7.20 7.18 7.16 7.14 7.14 7.12 7.07 7.06 7.05 7.05 7.05 7.04 7.03 7.03 7.02 6.95 6.93 6.90 6.88 6.86 6.84 5.80 4.64 4.62 4.59 3.00 2.99 2.99 2.97 2.96 2.95 2.92 2.90 2.88 2.26 2.26 2.25 2.24 2.23 2.22 2.21 2.19 2.18
Ph
Ph
N
H
3aj
1.01 4.04 2.07 7.04 2.04 3.06 1.00 1.02 2.05 2.05
8.5 8.0 7.5 7.0 6.5 6.0 5.5 5.0 4.5 4.0 3.5 3.0 2.5 2.0 1.5 1.0 0.5 0.0
f1 (ppm)

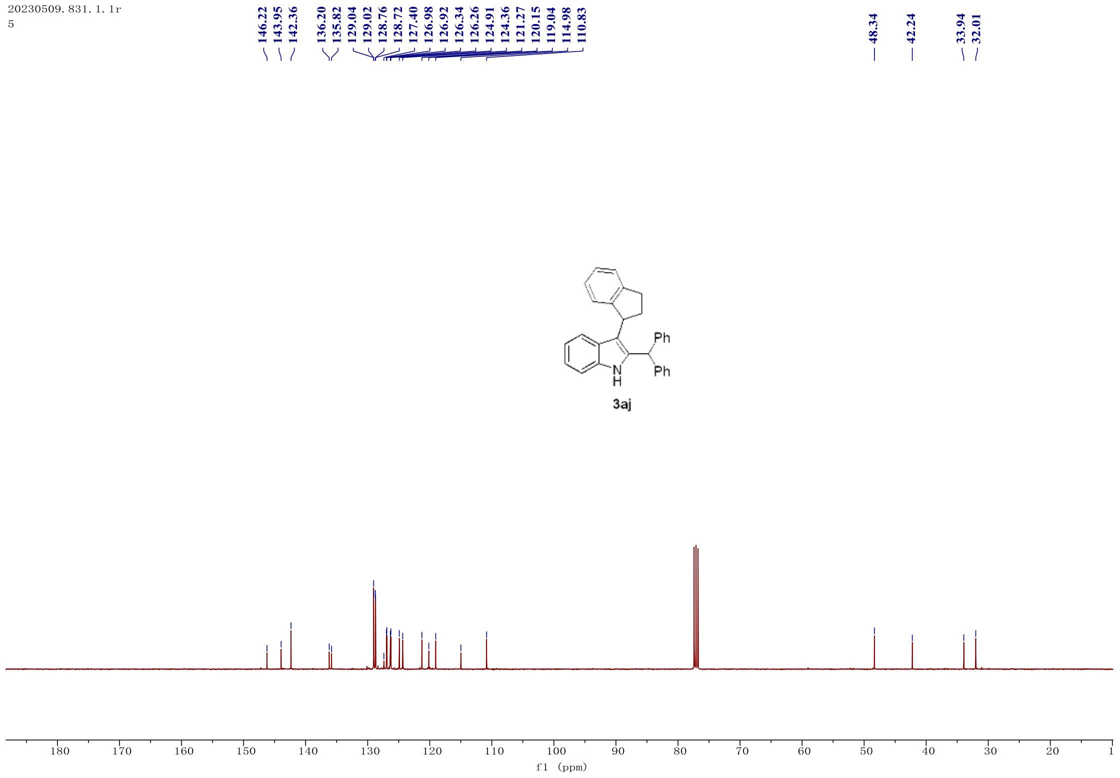

20230509.831.1.1r
5
146.22 143.95 142.36 136.20 135.82 129.04 129.02 128.76 128.72 127.40 126.98 126.92 126.34 126.26 124.91 124.36 121.27 120.15 119.04 114.98 110.83
48.34 42.24 33.94 32.01
Ph
Ph
N
H
3aj
180 170 160 150 140 130 120 110 100 90 80 70 60 50 40 30 20 1
f1 (ppm)

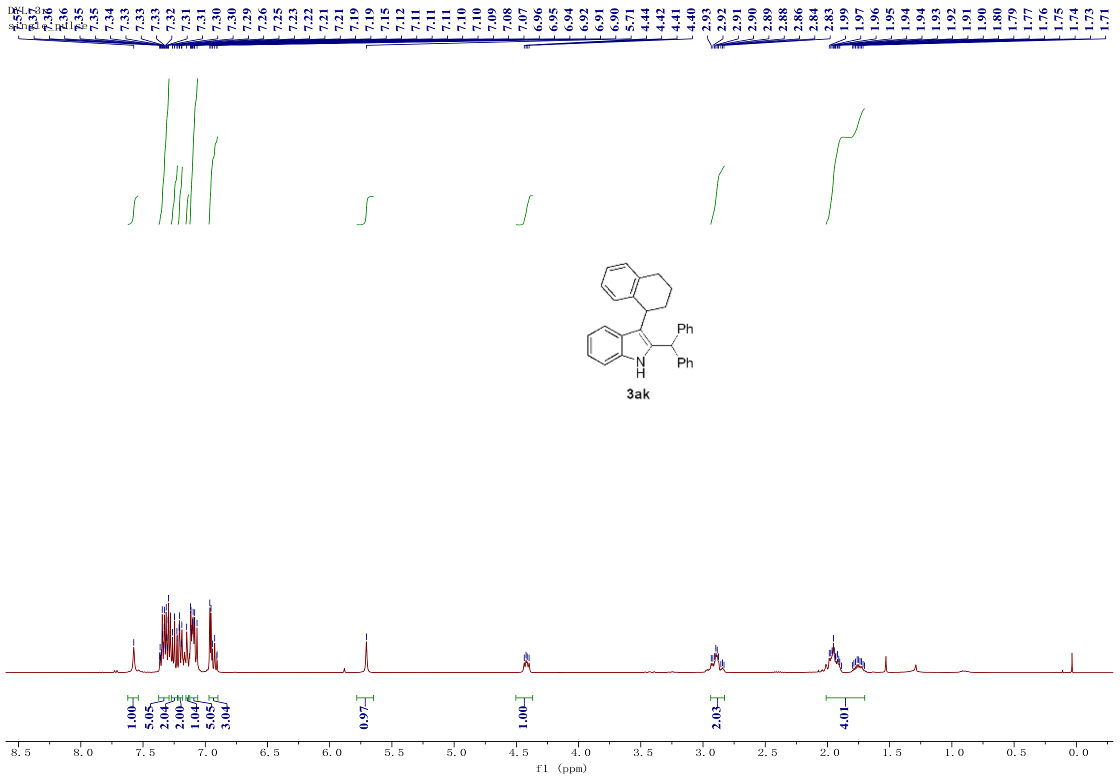
7.37 7.36 7.35 7.35 7.34 7.33 7.33 7.32 7.31 7.30 7.30 7.29 7.26 7.25 7.23 7.22 7.21 7.21 7.19 7.19 7.15 7.12 7.11 7.11 7.10 7.10 7.09 7.08 7.07 6.96 6.95 6.94 6.92 6.91 6.90 5.71 4.44 4.42 4.41 4.40 2.93 2.92 2.91 2.90 2.89 2.88 2.86 2.84 2.83 1.99 1.97 1.96 1.95 1.94 1.94 1.93 1.92 1.91 1.90 1.80 1.79 1.77 1.76 1.75 1.74 1.73 1.71
Ph
Ph
N
H
3ak
1.00 5.05 2.04 2.00 1.04 5.05 3.04 0.97 1.00 2.03 4.01
8.5 8.0 7.5 7.0 6.5 6.0 5.5 5.0 4.5 4.0 3.5 3.0 2.5 2.0 1.5 1.0 0.5 0.0
f1 (ppm)

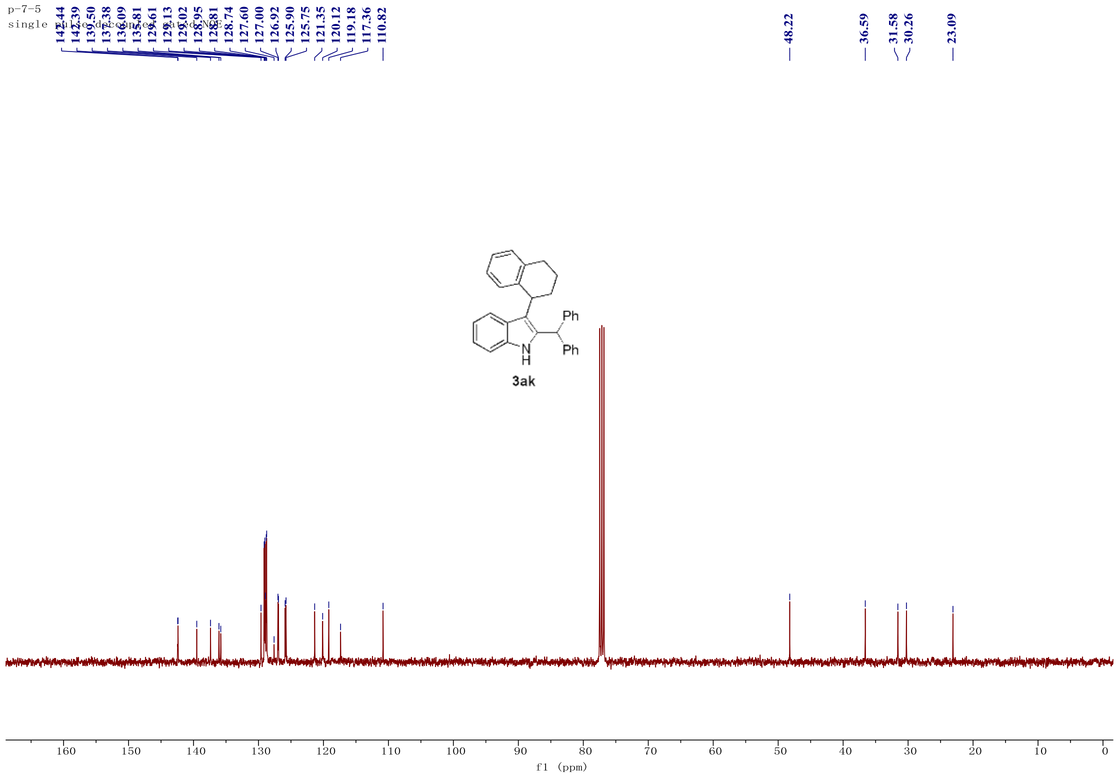
p-7-5
142.44 142.39 139.50 137.38 136.09 135.81 129.61 129.13 129.02 128.95 128.81 128.74 127.60 127.00 126.92 125.90 125.75 121.35 120.12 119.18 117.36 110.82 48.22 36.59 31.58 30.26 23.09
Ph
Ph
N
H
3ak
160 150 140 130 120 110 100 90 80 70 60 50 40 30 20 10 0
f1 (ppm)

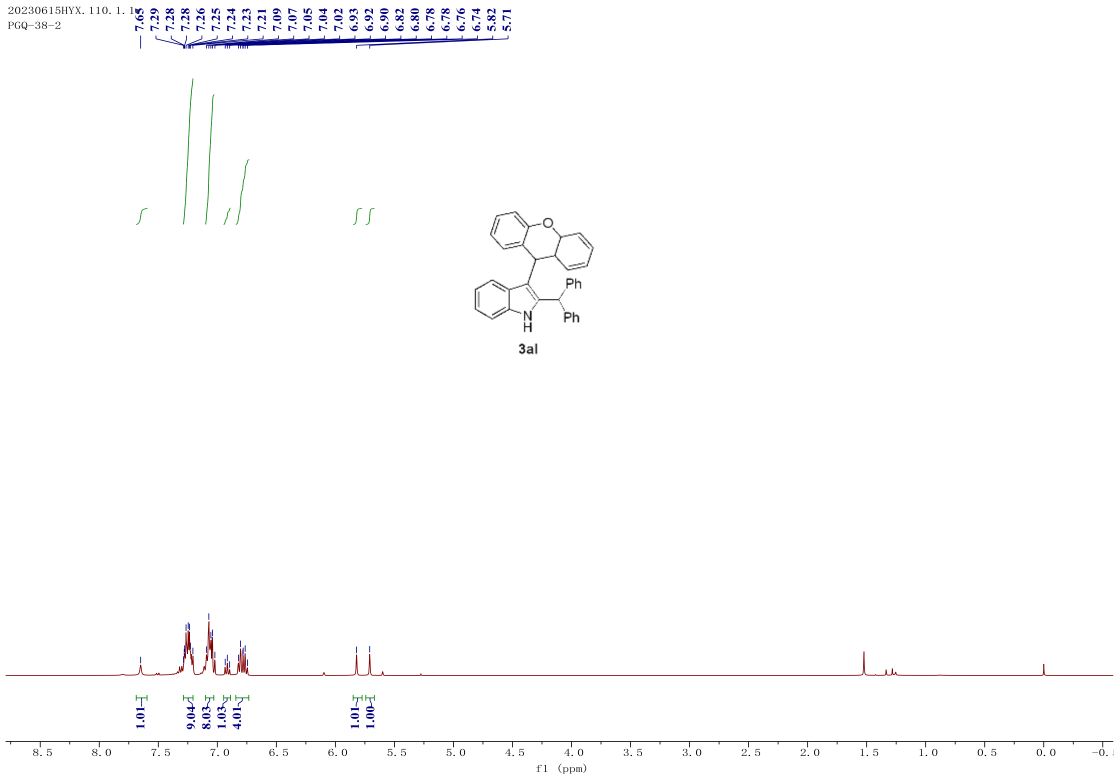
20230615HYX.110.1.1r
PGQ-38-2
7.65 7.29 7.28 7.26 7.25 7.24 7.23 7.21 7.09 7.07 7.05 7.04 7.02 6.93 6.92 6.90 6.82 6.80 6.78 6.78 6.76 6.74 5.82 5.71
Ph
Ph
N
H
O
3al
1.01 9.04 8.03 1.03 4.01 1.01 1.00
f1 (ppm)

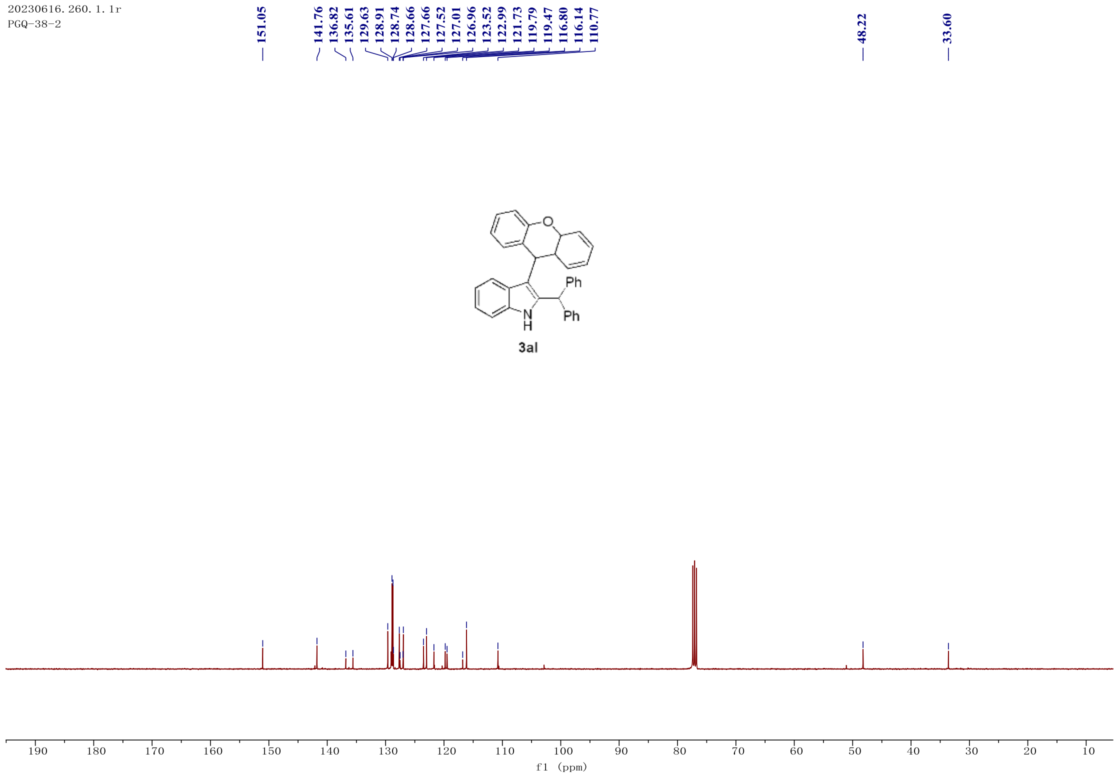
20230616.260.1.1r
PGQ-38-2
151.05 141.76 136.82 135.61 129.63 128.91 128.74 128.66 127.66 127.52 127.01 126.96 123.52 122.99 121.73 119.79 119.47 116.80 116.14 110.77 48.22 33.60
Ph
Ph
N
H
O
3al
f1 (ppm)

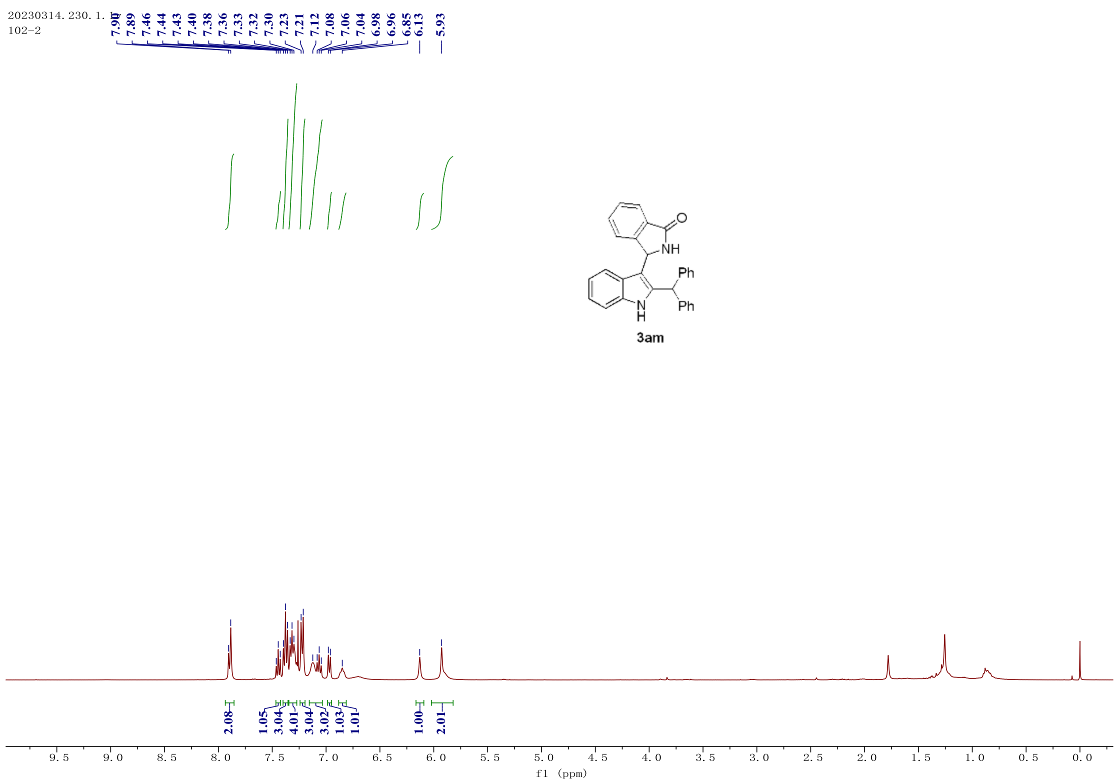

20230314.230.1.1r
102-2
7.90
7.89
7.46
7.44
7.43
7.40
7.38
7.36
7.33
7.32
7.30
7.23
7.21
7.12
7.08
7.06
7.04
6.98
6.96
6.85
6.13
5.93
O
NH
Ph
Ph
N
H
3am
2.08
1.05
3.04
4.01
3.04
3.02
1.03
1.01
1.00
2.01
9.5
9.0
8.5
8.0
7.5
7.0
6.5
6.0
5.5
5.0
4.5
4.0
3.5
3.0
2.5
2.0
1.5
1.0
0.5
0.0
f1 (ppm)

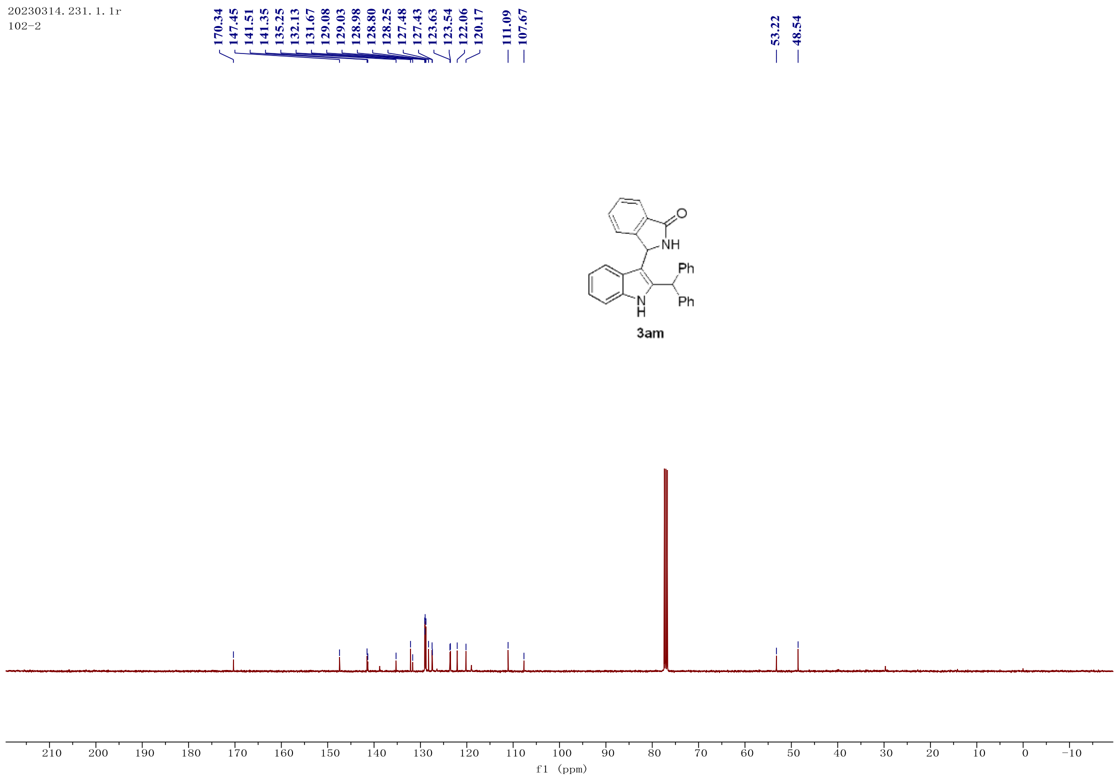

20230314.231.1.1r
102-2
170.34
147.45
141.51
141.35
135.25
132.13
131.67
129.08
129.03
128.98
128.80
128.25
127.48
127.43
123.63
123.54
122.06
120.17
111.09
107.67
53.22
48.54
O
NH
Ph
Ph
N
H
3am
210
200
190
180
170
160
150
140
130
120
110
100
90
80
70
60
50
40
30
20
10
0
-10
f1 (ppm)

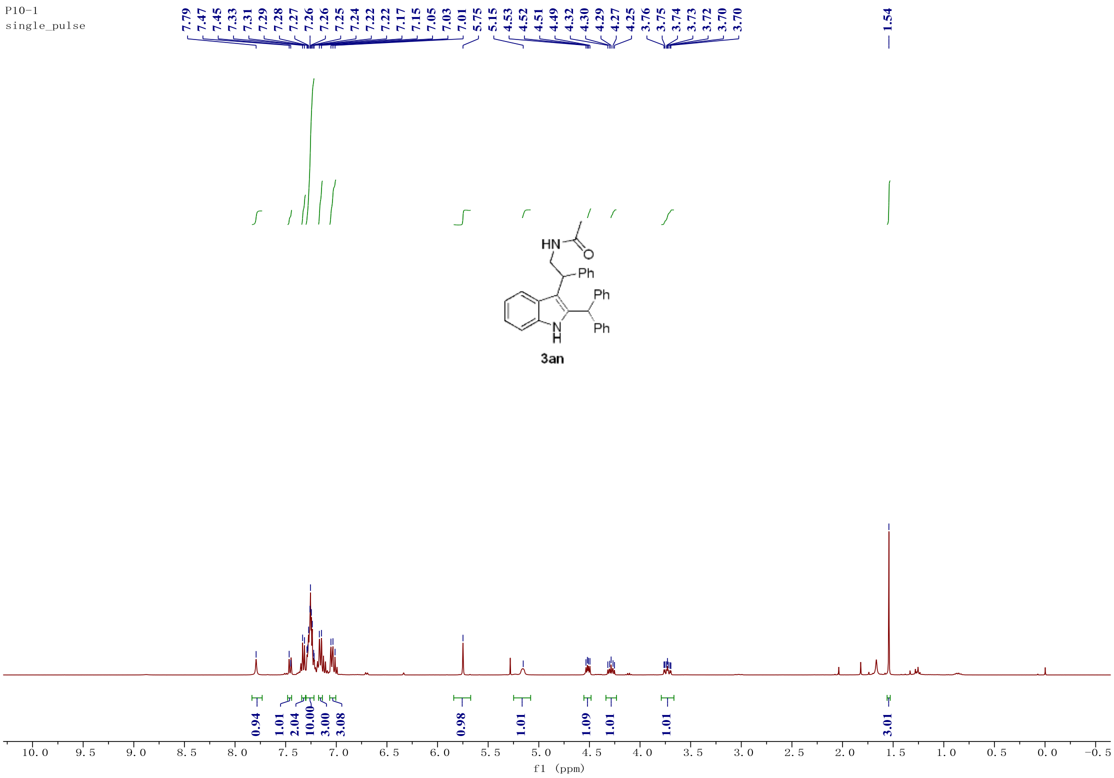
P10-1
single_pulse
7.79 7.47 7.45 7.33 7.31 7.29 7.28 7.27 7.26 7.26 7.25 7.24 7.22 7.22 7.17 7.15 7.05 7.03 7.01 5.75 5.15 4.53 4.52 4.51 4.49 4.32 4.30 4.29 4.27 4.25 3.76 3.75 3.74 3.73 3.72 3.70 3.70
1.54
HN
O
Ph
Ph
Ph
N
H
3an
0.94 1.01 2.04 10.00 3.00 3.08 0.98 1.01 1.09 1.01 1.01 3.01
f1 (ppm)

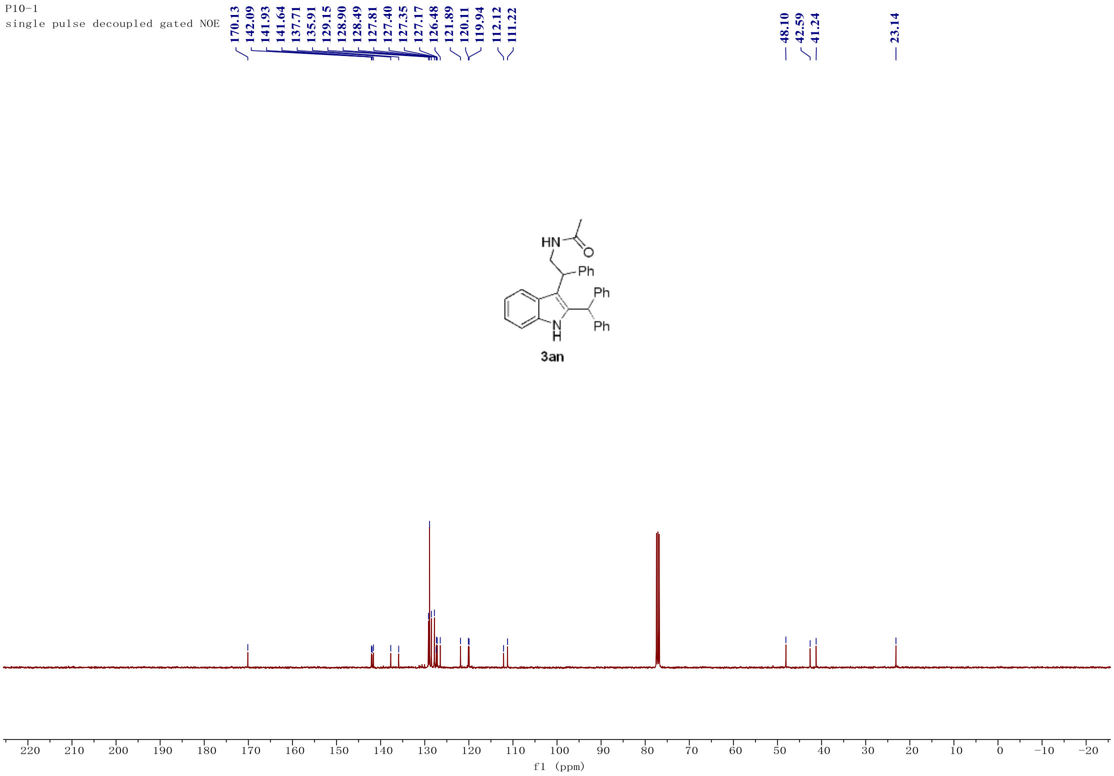
P10-1
single pulse decoupled gated NOE
170.13 142.09 141.93 141.64 137.71 135.91 129.15 128.90 128.49 127.81 127.40 127.35 127.17 126.48 121.89 120.11 119.94 112.12 111.22
48.10 42.59 41.24
23.14
HN
O
Ph
Ph
Ph
N
H
3an
f1 (ppm)

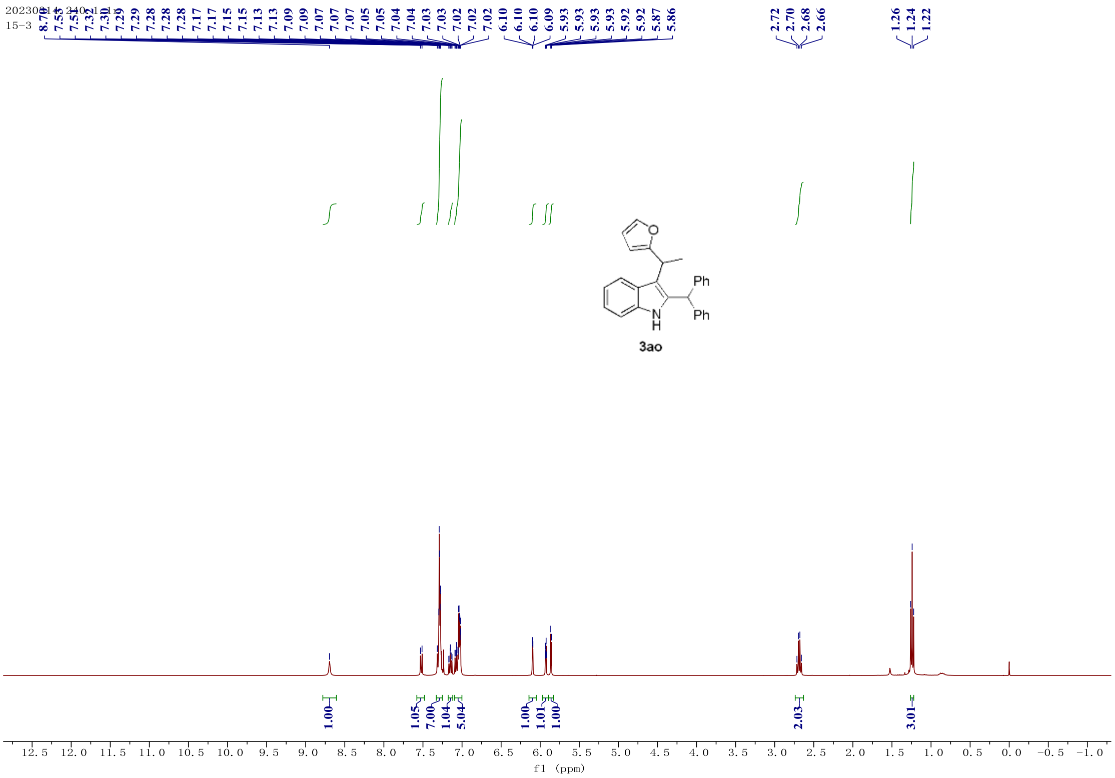
15-3
8.70
7.53
7.51
7.32
7.30
7.29
7.29
7.28
7.28
7.28
7.17
7.15
7.15
7.13
7.13
7.09
7.09
7.07
7.07
7.07
7.05
7.05
7.04
7.04
7.03
7.03
7.02
7.02
7.02
6.10
6.10
6.10
6.09
5.93
5.93
5.93
5.93
5.92
5.92
5.87
5.86
2.72
2.70
2.68
2.66
1.26
1.24
1.22
Ph
Ph
N
H
3ao
1.00
1.05
7.00
1.04
5.04
1.00
1.01
1.00
2.03
3.01
f1 (ppm)

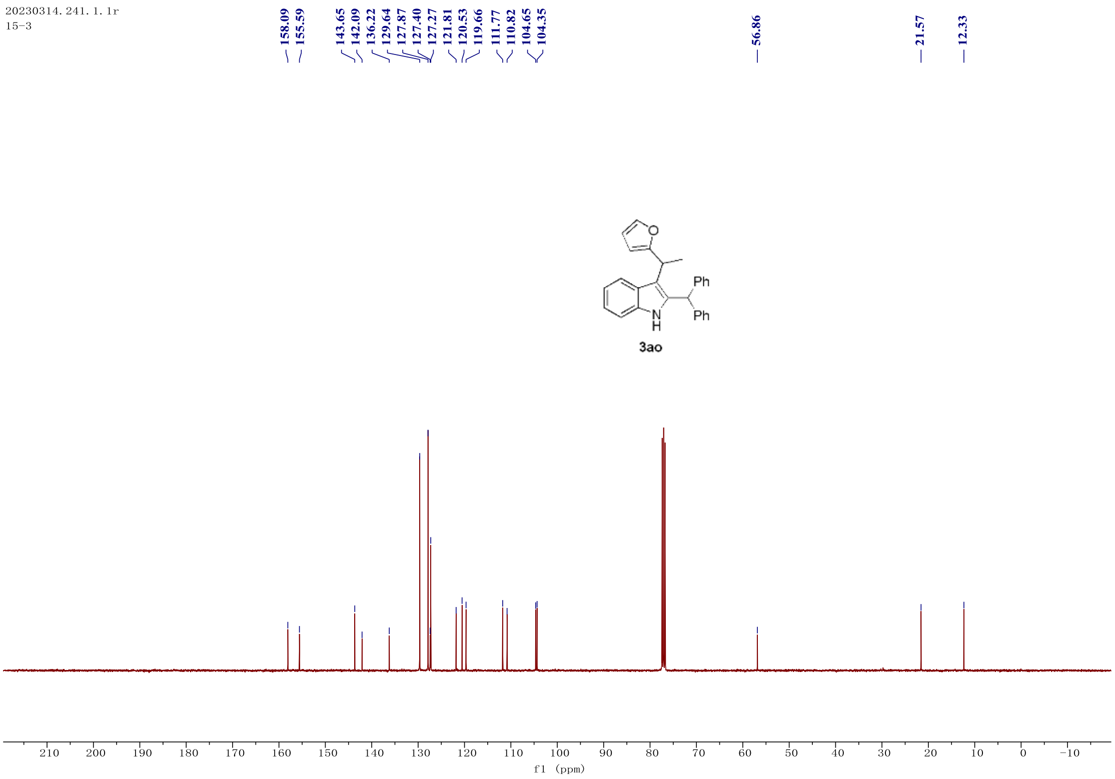
20230314.241.1.1r
15-3
158.09
155.59
143.65
142.09
136.22
129.64
127.87
127.40
127.27
121.81
120.53
119.66
111.77
110.82
104.65
104.35
56.86
21.57
12.33
Ph
Ph
N
H
3ao
f1 (ppm)

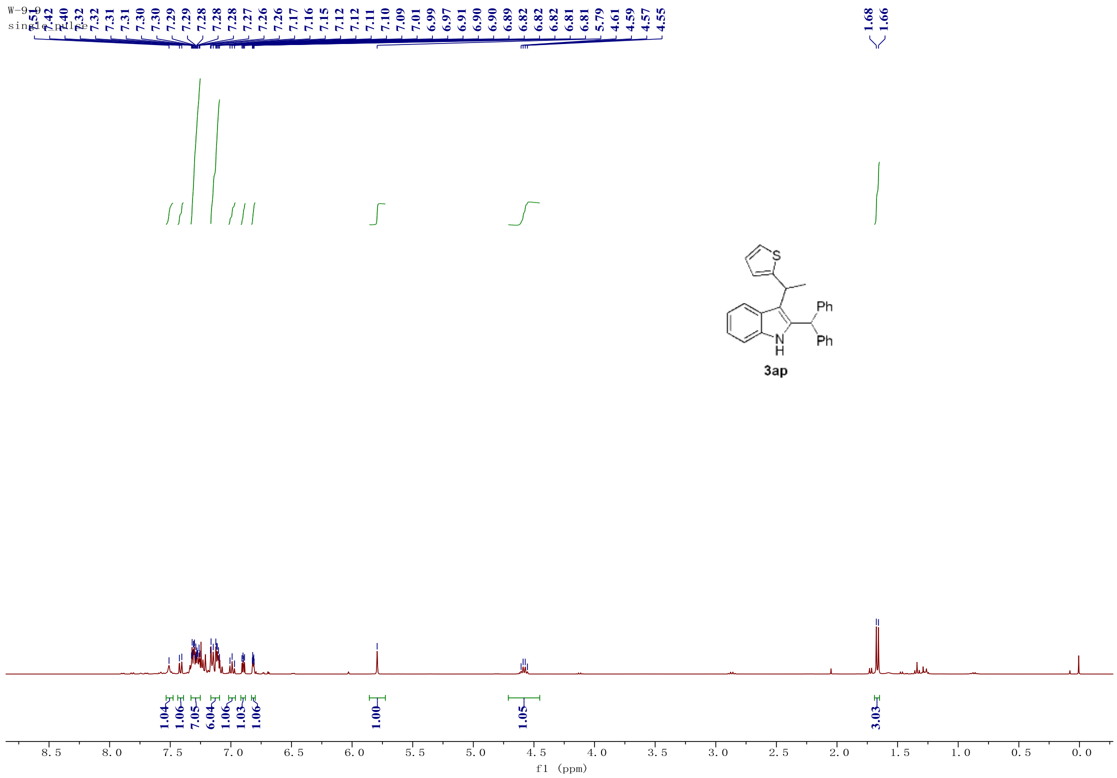
W-9-9
single pulse
7.51 7.42 7.40 7.32 7.32 7.31 7.30 7.29 7.29 7.28 7.28 7.28 7.27 7.26 7.26 7.17 7.16 7.15 7.12 7.12 7.11 7.10 7.09 7.01 6.99 6.97 6.91 6.90 6.90 6.89 6.82 6.82 6.82 6.81 6.81 5.79 4.61 4.59 4.57 4.55
1.68 1.66
S
Ph
Ph
N
H
3ap
1.04 1.06 7.05 6.04 1.06 1.03 1.06 1.00 1.05 3.03
8.5 8.0 7.5 7.0 6.5 6.0 5.5 5.0 4.5 4.0 3.5 3.0 2.5 2.0 1.5 1.0 0.5 0.0
f1 (ppm)

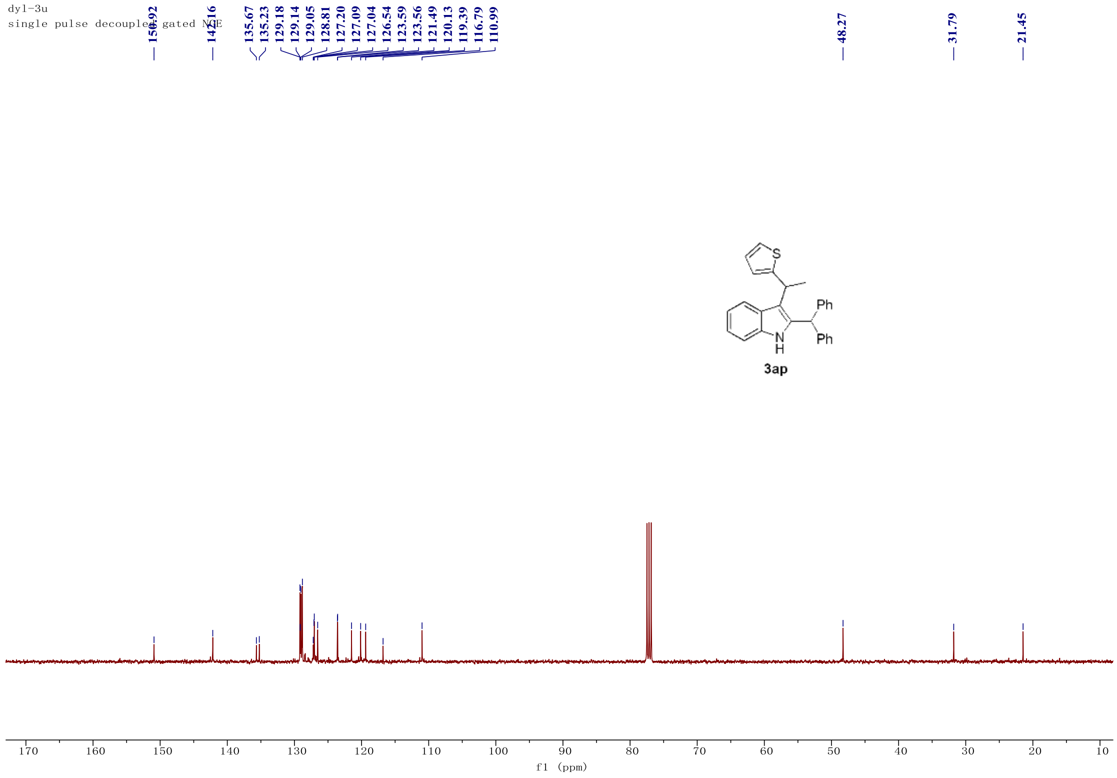
dyl-3u
single pulse decoupled gated NOE
150.92 142.16 135.67 135.23 129.18 129.14 129.05 128.81 127.20 127.09 127.04 126.54 123.59 123.56 121.49 120.13 119.39 116.79 110.99
48.27 31.79 21.45
S
Ph
Ph
N
H
3ap
170 160 150 140 130 120 110 100 90 80 70 60 50 40 30 20 10
f1 (ppm)

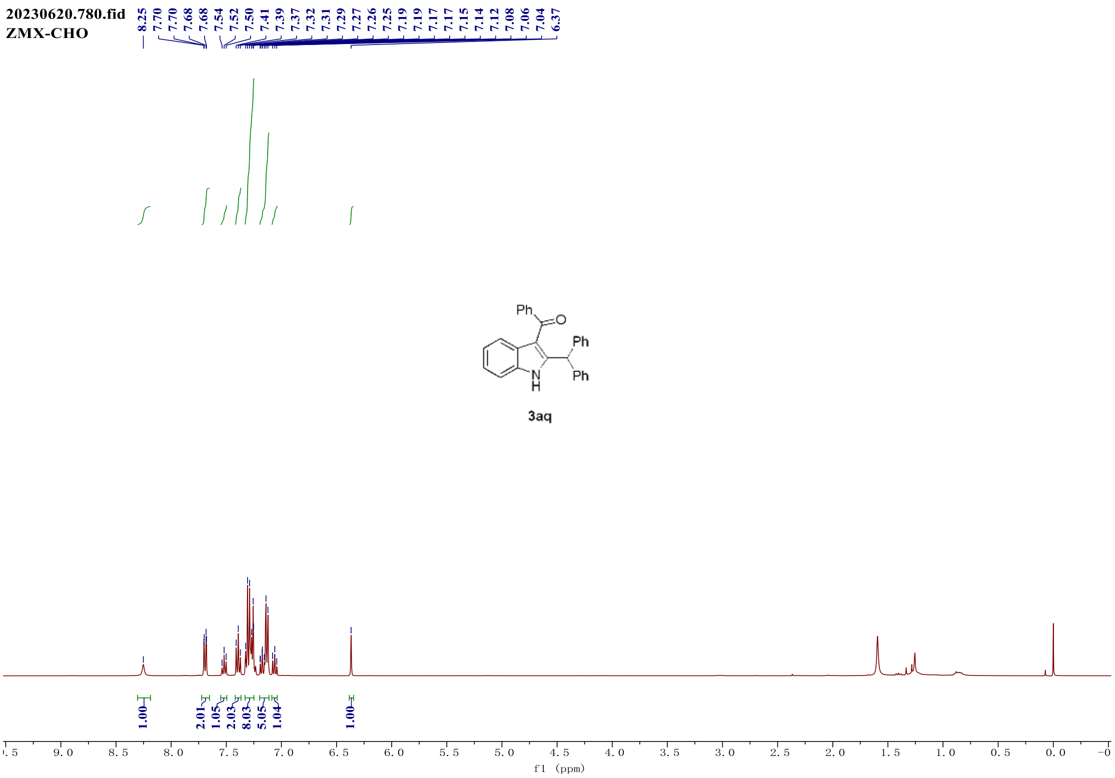
20230620.780.fid
ZMX-CHO
8.25
7.70
7.68
7.68
7.54
7.52
7.50
7.41
7.39
7.37
7.32
7.31
7.29
7.27
7.26
7.25
7.19
7.19
7.17
7.17
7.15
7.14
7.12
7.08
7.06
7.04
6.37
Ph
O
Ph
Ph
N
H
3aq
1.00
2.01
1.05
2.03
8.03
5.05
1.04
1.00
f1 (ppm)

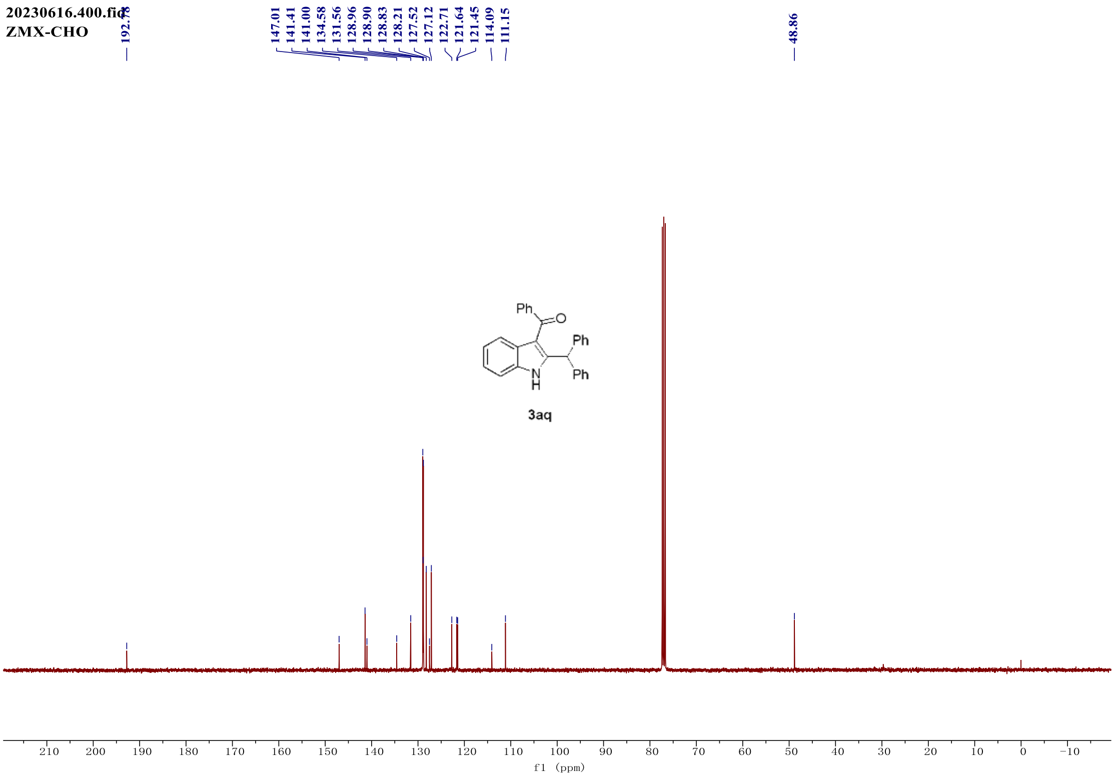
20230616.400.fid
ZMX-CHO
192.78
147.01
141.41
141.00
134.58
131.56
128.96
128.90
128.83
128.21
127.52
127.12
122.71
121.64
121.45
114.09
111.15
48.86
Ph
O
Ph
Ph
N
H
3aq
f1 (ppm)

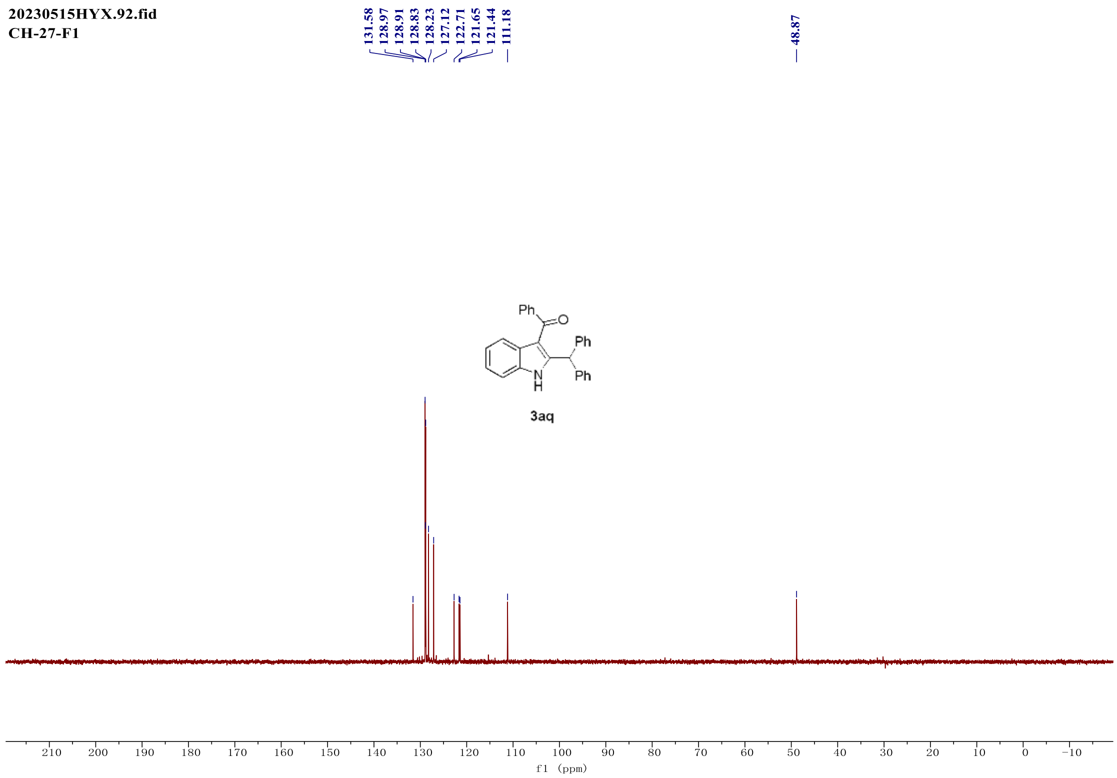
20230515HYX.92.fid
CH-27-F1
131.58
128.97
128.91
128.83
128.23
127.12
122.71
121.65
121.44
111.18
48.87
Ph
O
Ph
Ph
N
H
3aq
f1 (ppm)

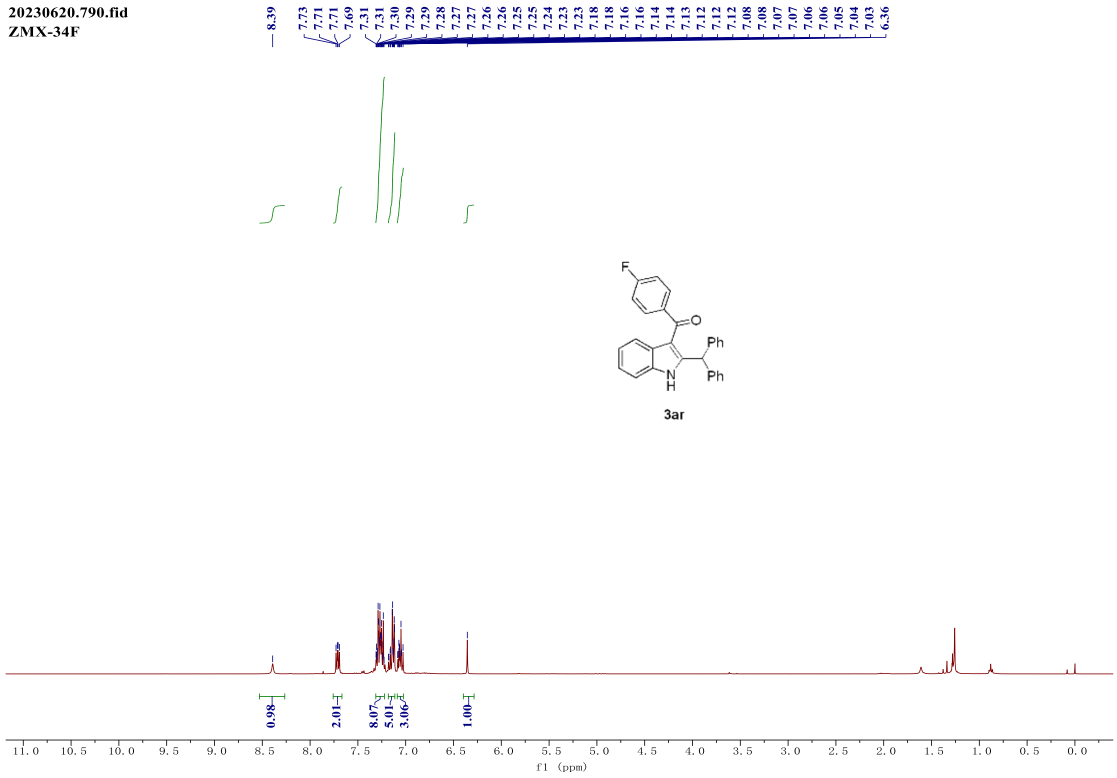
20230620.790.fid
ZMX-34F
8.39
7.73
7.71
7.71
7.69
7.31
7.31
7.30
7.29
7.29
7.28
7.27
7.27
7.26
7.25
7.25
7.24
7.23
7.23
7.18
7.16
7.16
7.14
7.14
7.13
7.12
7.12
7.12
7.08
7.08
7.07
7.07
7.06
7.06
7.05
7.04
7.03
6.36
F
O
Ph
Ph
N
H
3ar
0.98
2.01
8.07
5.01
3.06
1.00
f1 (ppm)

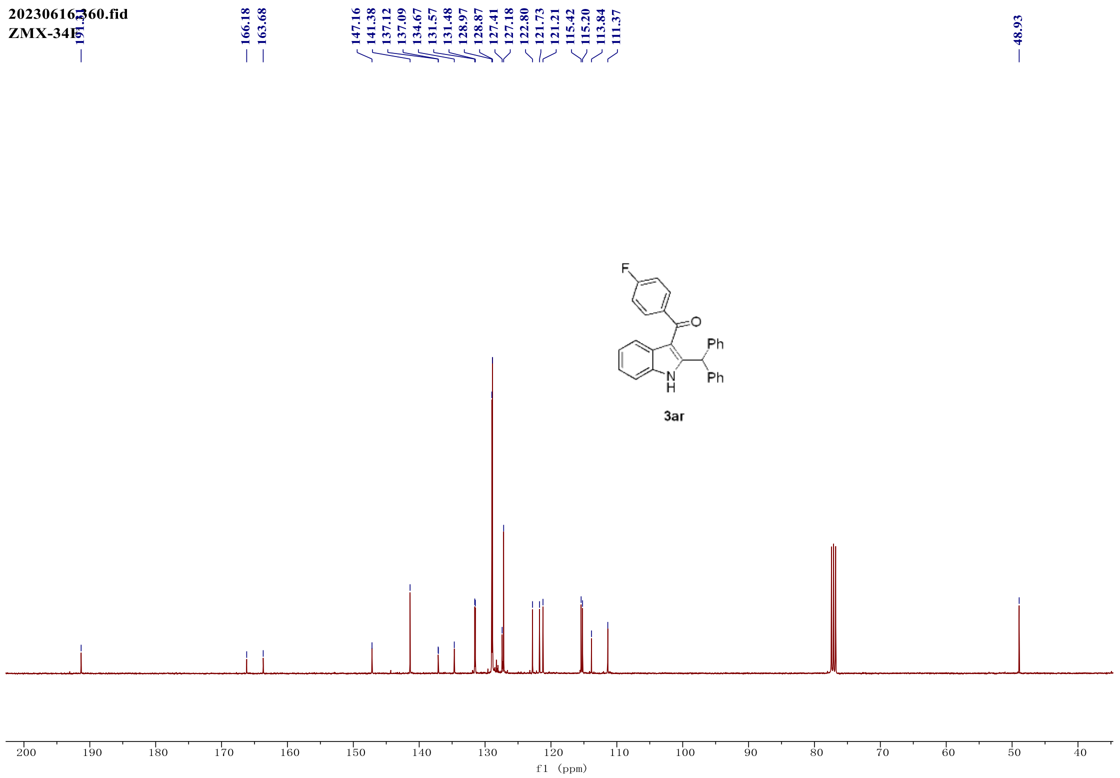
20230616.360.fid
ZMX-34F
166.18
163.68
147.16
141.38
137.12
137.09
134.67
131.57
131.48
128.97
128.87
127.41
127.18
122.80
121.73
121.21
115.42
115.20
113.84
111.37
48.93
F
O
Ph
Ph
N
H
3ar
f1 (ppm)

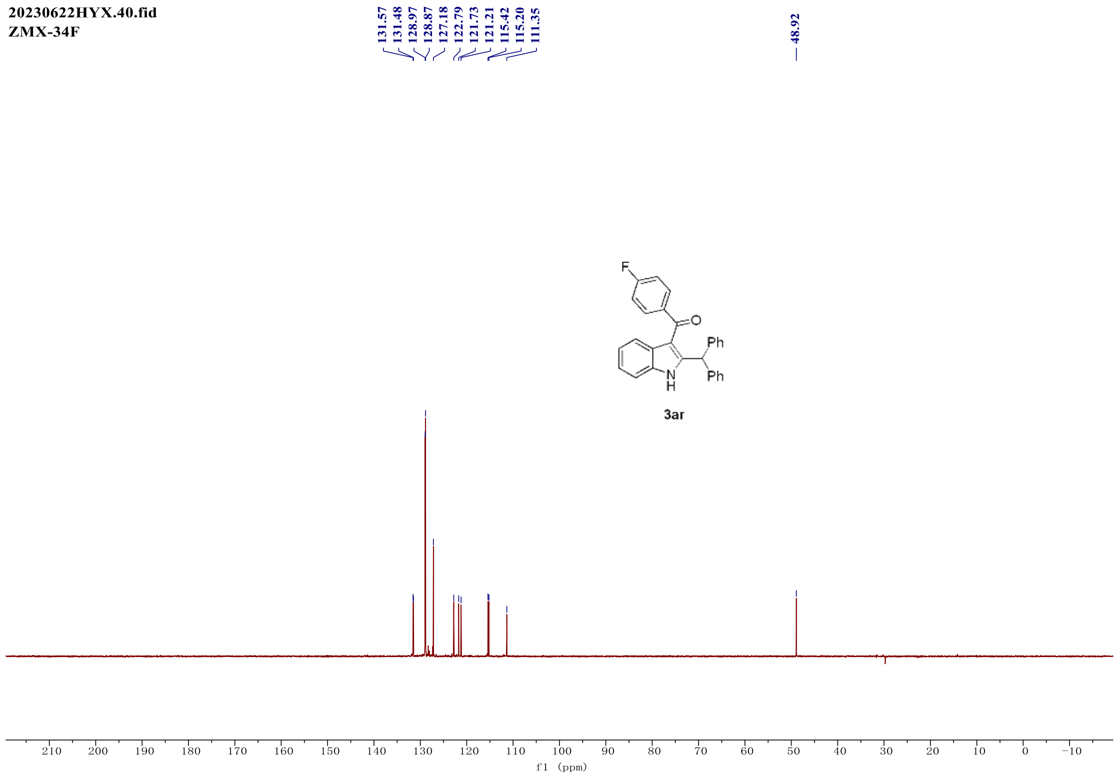
20230622HYX.40.fid
ZMX-34F
131.57
131.48
128.97
128.87
127.18
122.79
121.73
121.21
115.42
115.20
111.35
48.92
F
O
Ph
Ph
N
H
3ar
f1 (ppm)

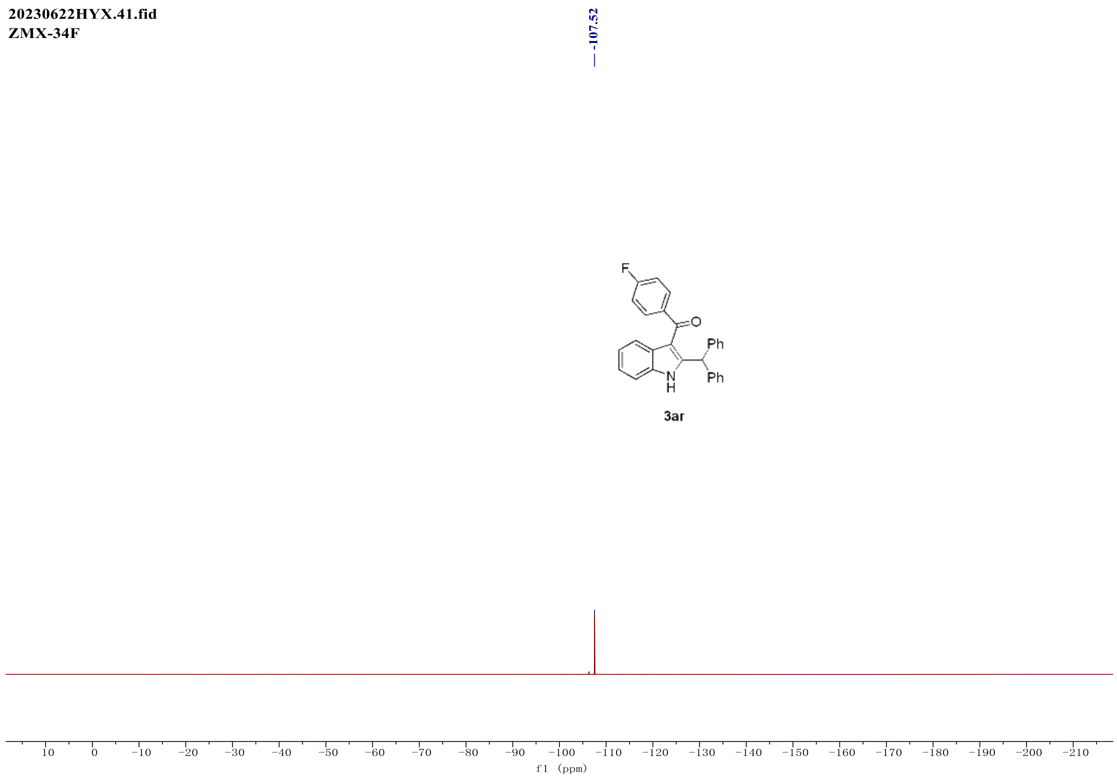
20230622HYX.41.fid
ZMX-34F
-107.52
F
O
Ph
Ph
N
H
3ar
10 0 −10 −20 −30 −40 −50 −60 −70 −80 −90 −100 −110 −120 −130 −140 −150 −160 −170 −180 −190 −200 −210
f1 (ppm)

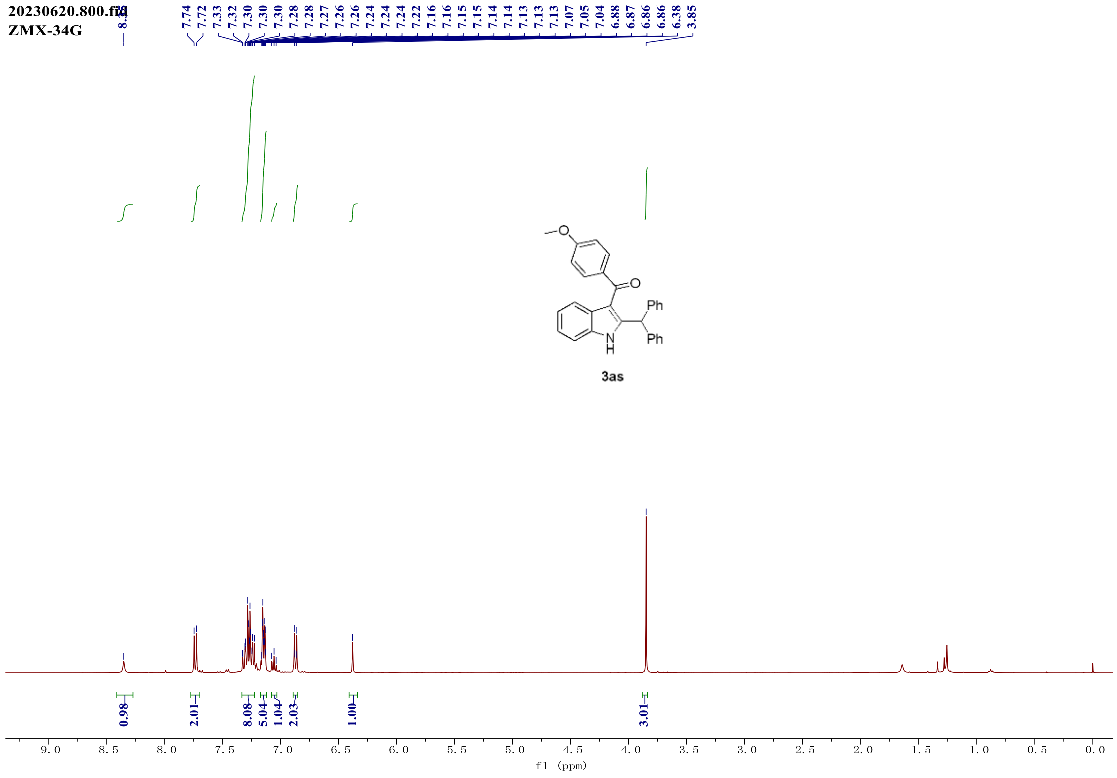
ZMX-34G
7.74 7.72 7.33 7.32 7.30 7.30 7.30 7.28 7.28 7.27 7.26 7.26 7.24 7.24 7.24 7.22 7.16 7.16 7.15 7.15 7.14 7.14 7.13 7.13 7.13 7.07 7.05 7.04 6.88 6.87 6.86 6.86 6.38 3.85
O
O
Ph
Ph
N
H
3as
0.98 2.01 8.08 5.04 1.04 2.03 1.00 3.01
9.0 8.5 8.0 7.5 7.0 6.5 6.0 5.5 5.0 4.5 4.0 3.5 3.0 2.5 2.0 1.5 1.0 0.5 0.0
f1 (ppm)

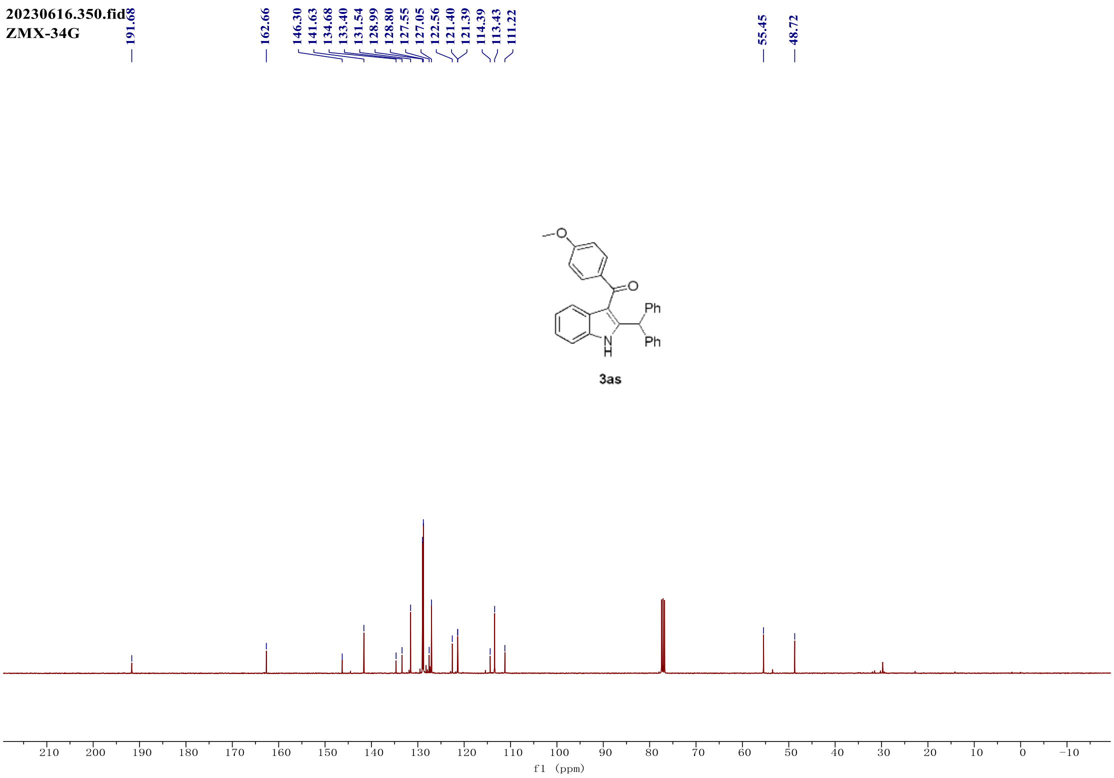
20230616.350.fid
ZMX-34G
191.68
162.66
146.30
141.63
134.68
133.40
131.54
128.99
128.80
127.55
127.05
122.56
121.40
121.39
114.39
113.43
111.22
55.45
48.72
Ph
Ph
O
N
H
3as
210 200 190 180 170 160 150 140 130 120 110 100 90 80 70 60 50 40 30 20 10 0 -10
f1 (ppm)

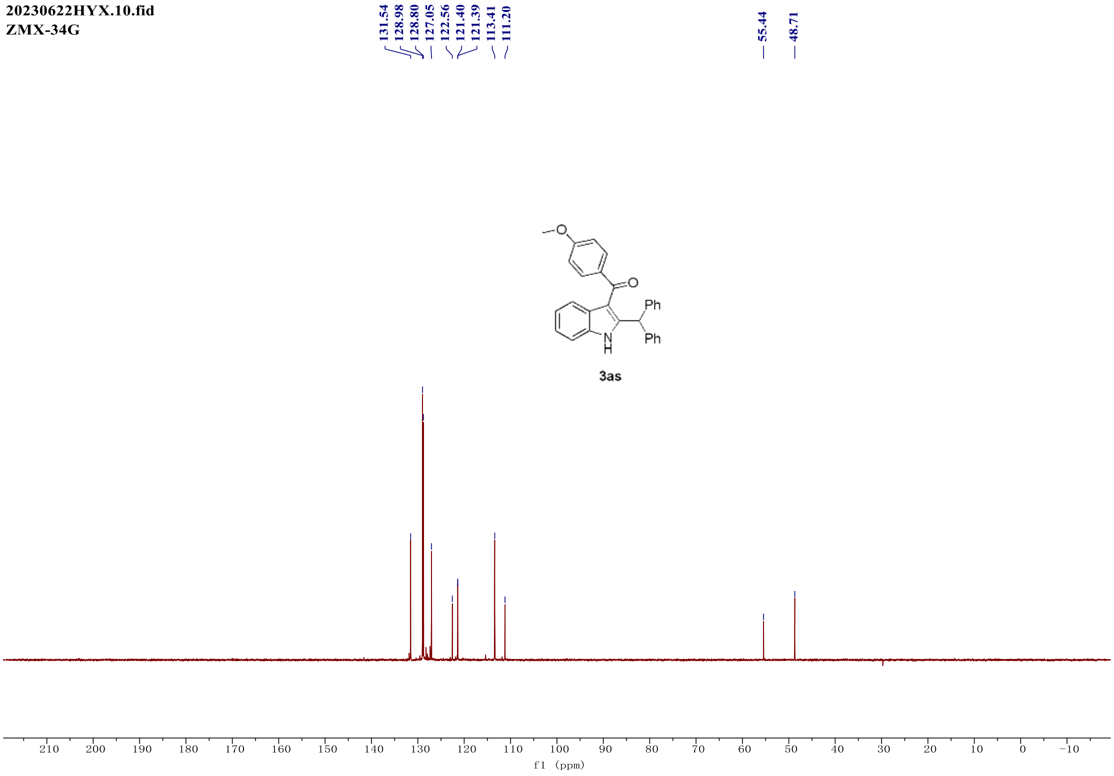
20230622HYX.10.fid
ZMX-34G
131.54
128.98
128.80
127.05
122.56
121.40
121.39
113.41
111.20
55.44
48.71
Ph
Ph
O
N
H
3as
210 200 190 180 170 160 150 140 130 120 110 100 90 80 70 60 50 40 30 20 10 0 -10
f1 (ppm)

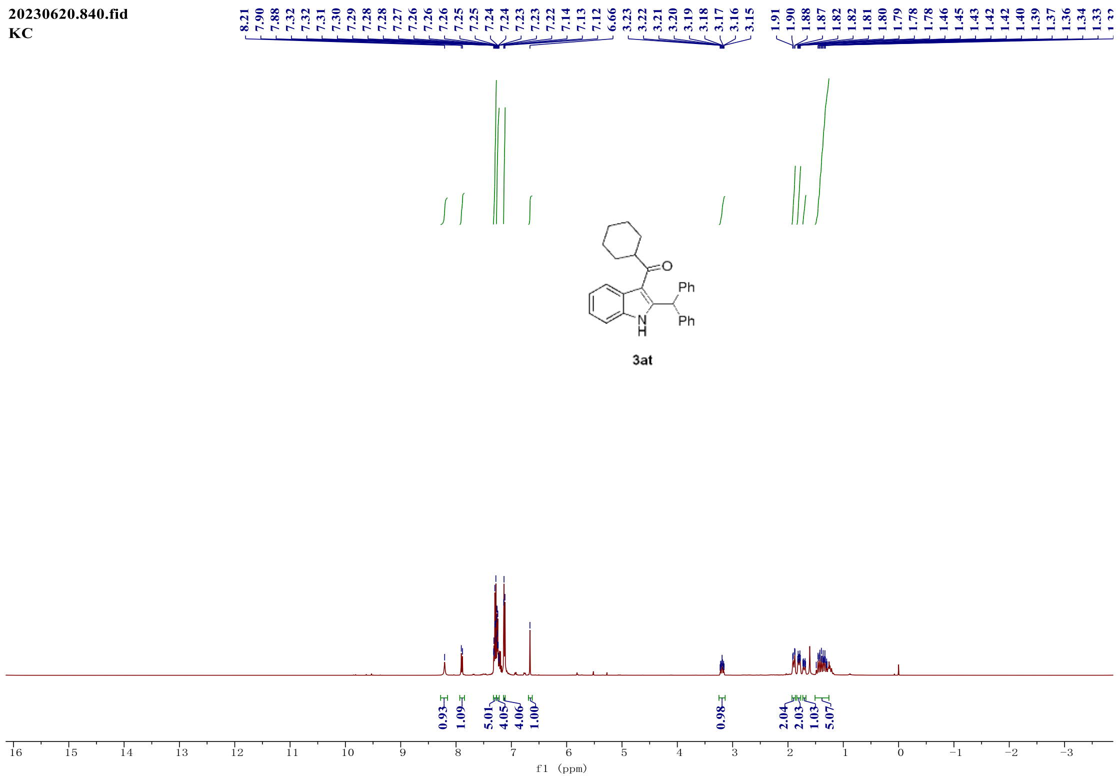
20230620.840.fid
KC
8.21 7.90 7.88 7.32 7.32 7.31 7.30 7.29 7.28 7.28 7.27 7.26 7.26 7.26 7.25 7.25 7.24 7.24 7.24 7.23 7.23 7.22 7.14 7.13 7.12 6.66 3.23 3.22 3.21 3.20 3.19 3.18 3.17 3.16 3.15 1.91 1.90 1.88 1.87 1.82 1.82 1.81 1.80 1.79 1.78 1.78 1.46 1.45 1.43 1.42 1.42 1.40 1.39 1.37 1.36 1.34 1.33
Ph
Ph
O
N
H
3at
0.93 1.09 5.01 4.05 4.06 1.00 0.98 2.04 2.03 1.03 5.07
16 15 14 13 12 11 10 9 8 7 6 5 4 3 2 1 0 −1 −2 −3
f1 (ppm)

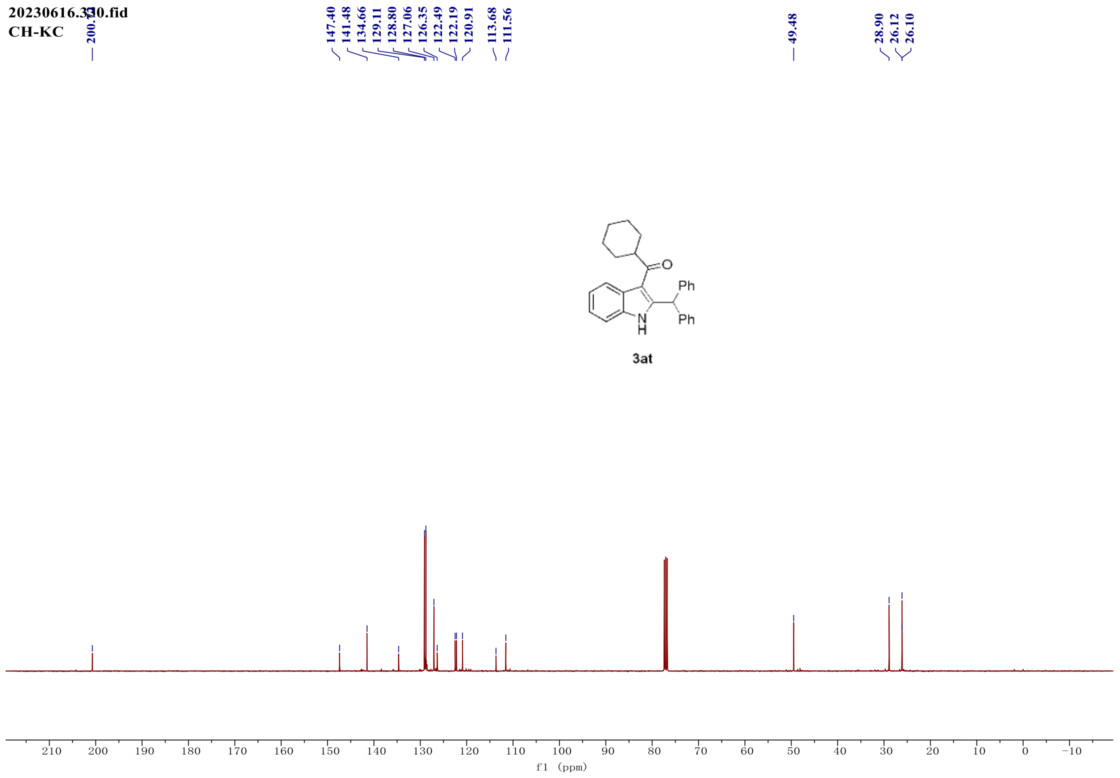
CH-KC
147.40 141.48 134.66 129.11 128.80 127.06 126.35 122.49 122.19 120.91 113.68 111.56 49.48 28.90 26.12 26.10
Ph
Ph
O
N
H
3at
210 200 190 180 170 160 150 140 130 120 110 100 90 80 70 60 50 40 30 20 10 0 −10
f1 (ppm)

20230622HYX.20.fid
CH-KC

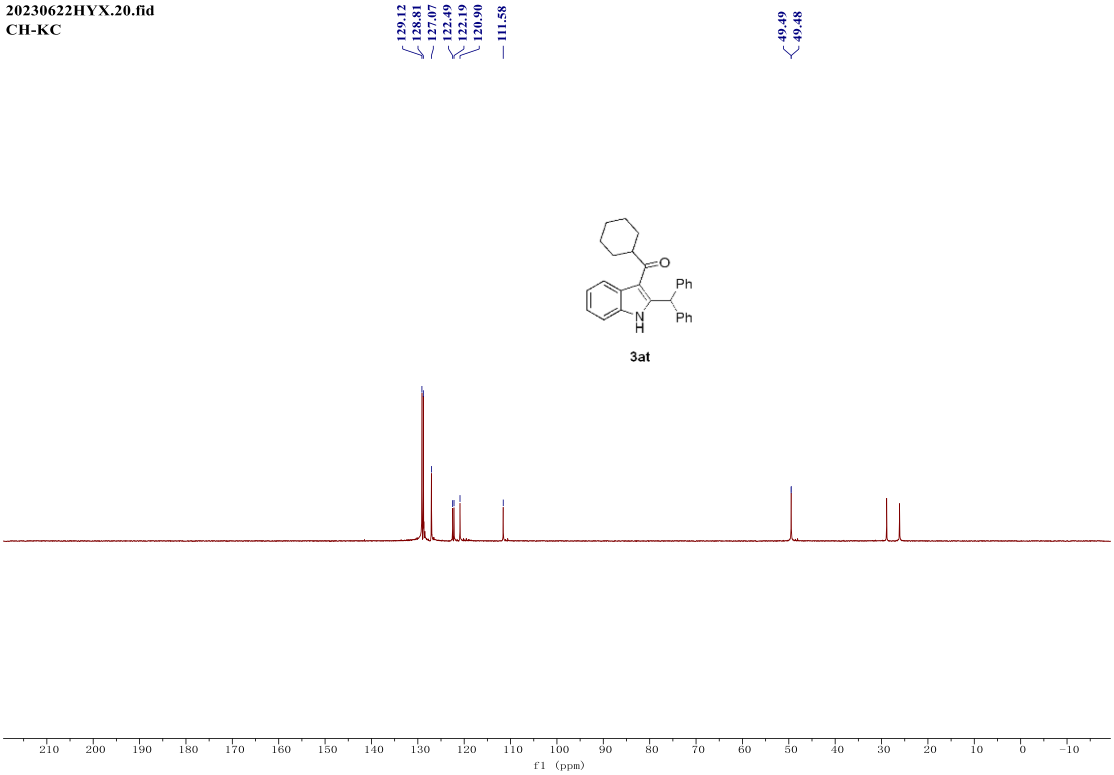

3at

20230620.820.fid
CH-KD

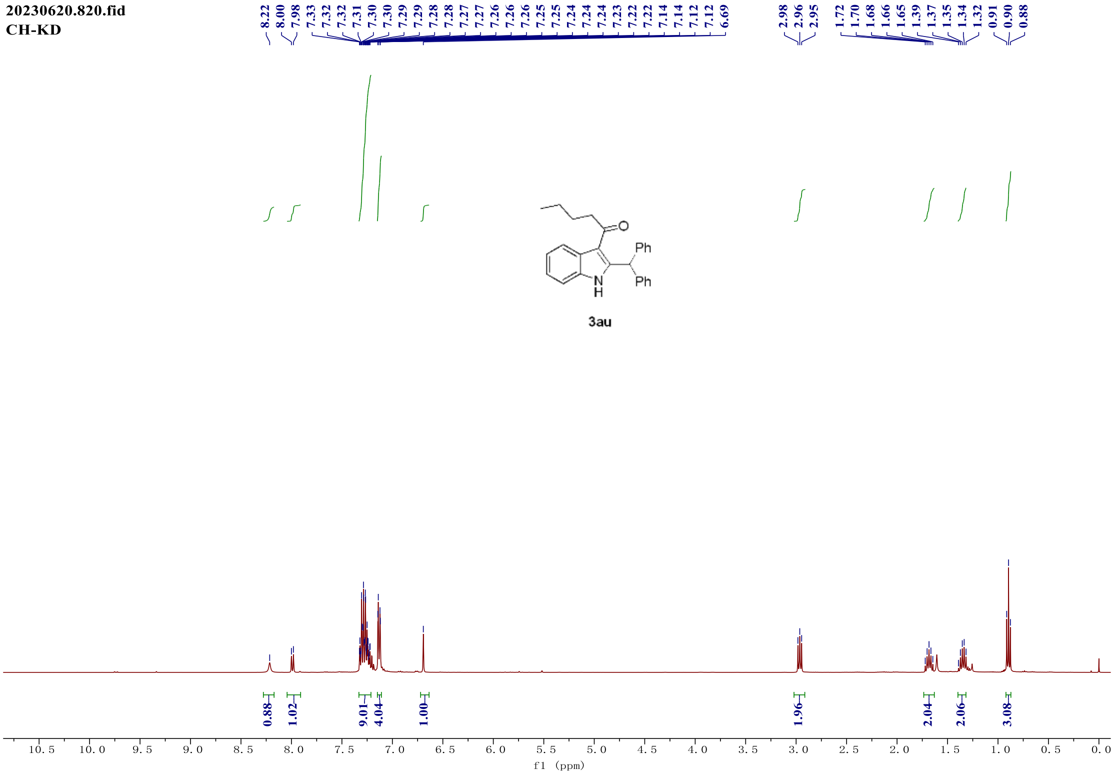

3au

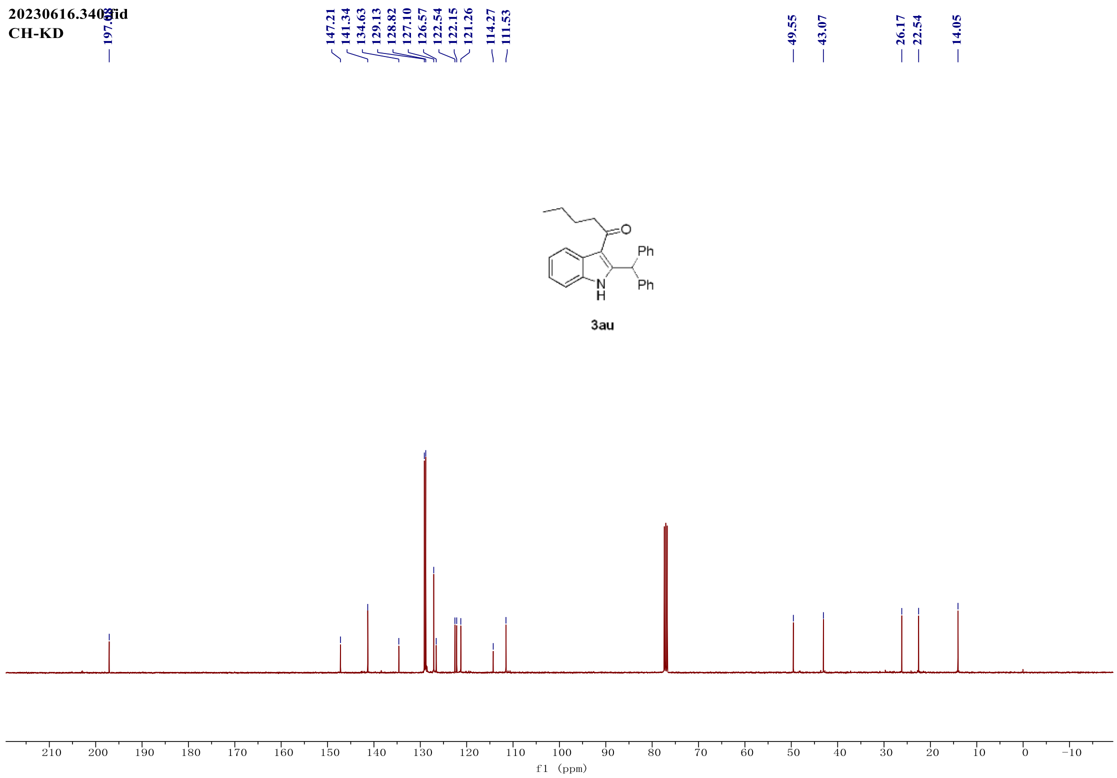
20230616.340[illegible]fid
CH-KD
197.[illegible]
147.21
141.34
134.63
129.13
128.82
127.10
126.57
122.54
122.15
121.26
114.27
111.53
49.55
43.07
26.17
22.54
14.05
Ph
Ph
N
H
3au
210 200 190 180 170 160 150 140 130 120 110 100 90 80 70 60 50 40 30 20 10 0 -10
f1 (ppm)

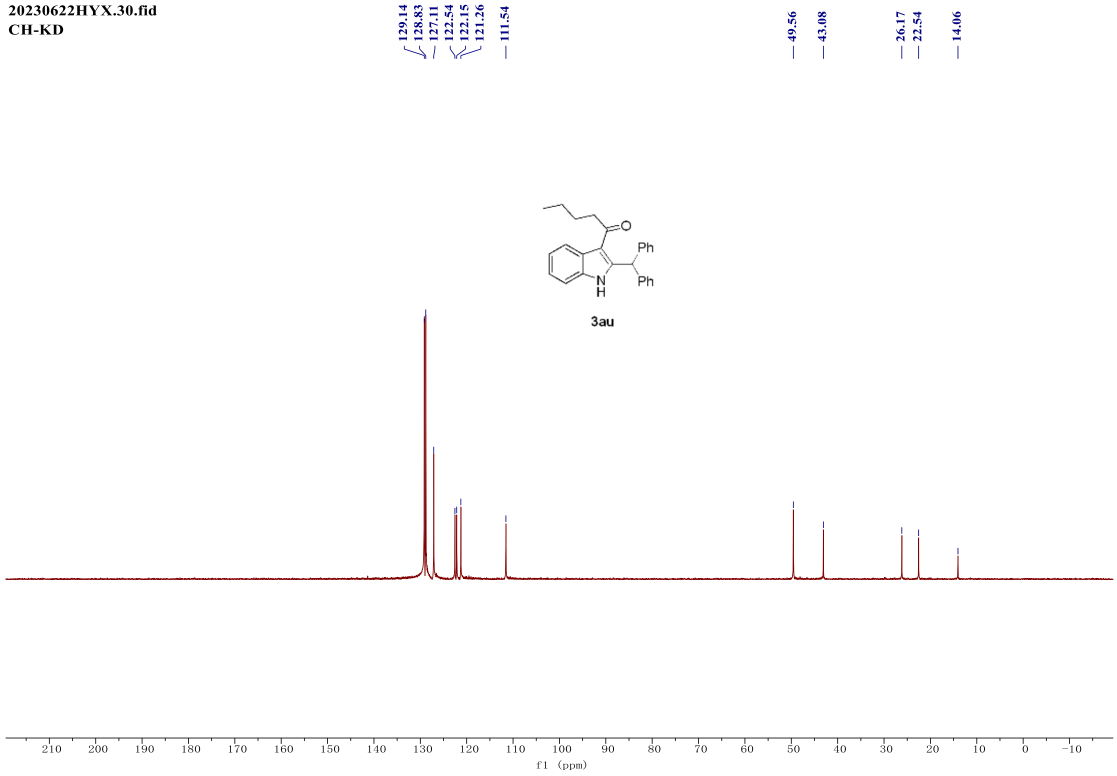
20230622HYX.30.fid
CH-KD
129.14
128.83
127.11
122.54
122.15
121.26
111.54
49.56
43.08
26.17
22.54
14.06
Ph
Ph
N
H
3au
210 200 190 180 170 160 150 140 130 120 110 100 90 80 70 60 50 40 30 20 10 0 -10
f1 (ppm)

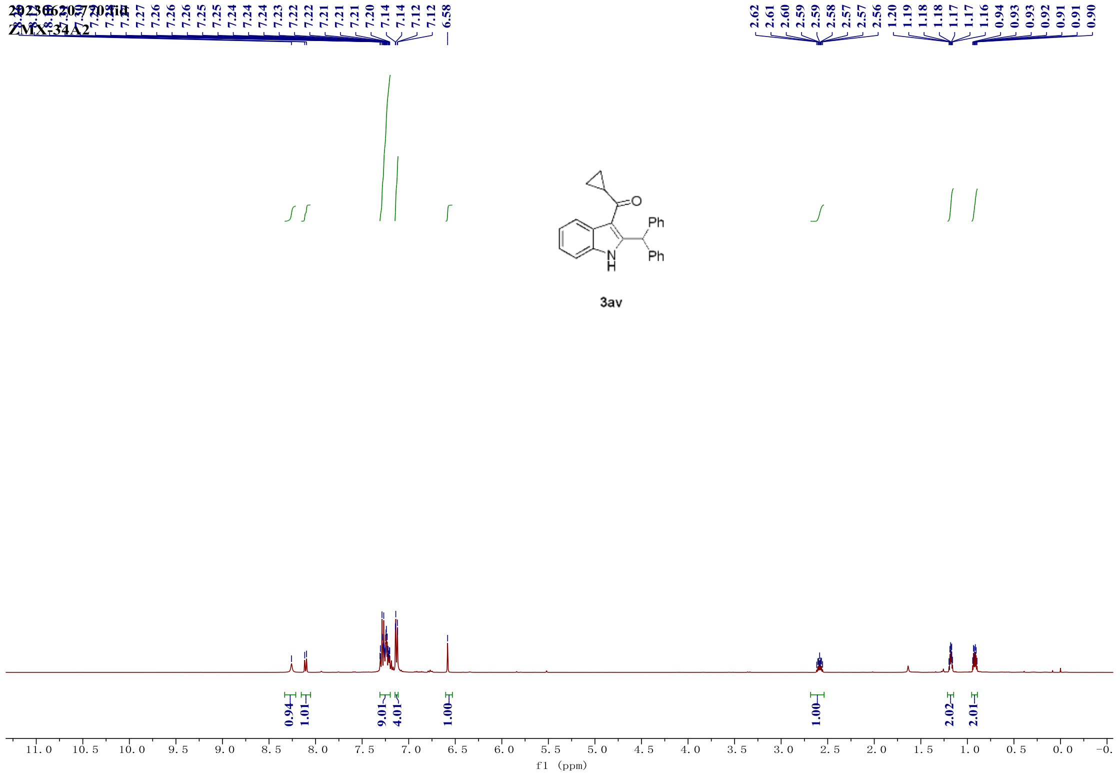
ZMX-34A2
7.27 7.26 7.26 7.25 7.25 7.24 7.24 7.24 7.23 7.22 7.22 7.21 7.21 7.21 7.20 7.14 7.14 7.12 7.12 6.58
2.62 2.61 2.60 2.59 2.59 2.58 2.57 2.57 2.56 1.20 1.19 1.18 1.17 1.17 1.16 0.94 0.93 0.93 0.92 0.91 0.90
Ph
Ph
N
H
3av
0.94 1.01 9.01 4.01 1.00 1.00 2.02 2.01
f1 (ppm)

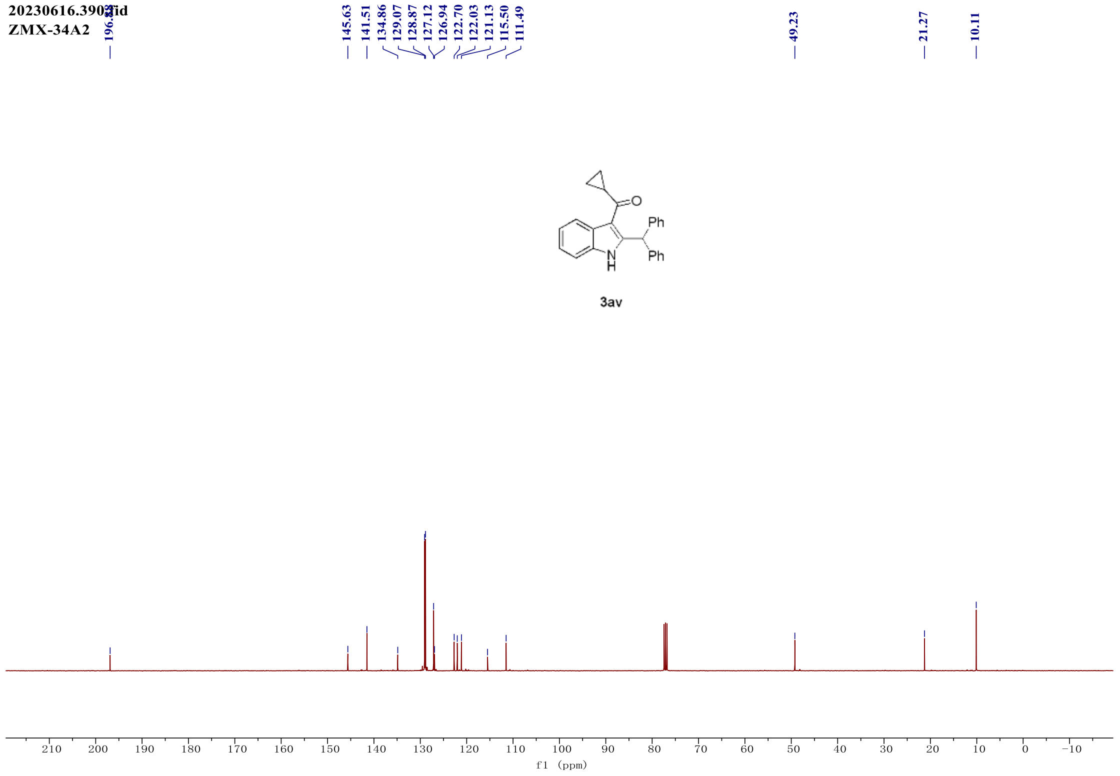
20230616.390.fid
ZMX-34A2
196.8
145.63 141.51 134.86 129.07 128.87 127.12 126.94 122.70 122.03 121.13 115.50 111.49
49.23
21.27
10.11
Ph
Ph
N
H
3av
f1 (ppm)

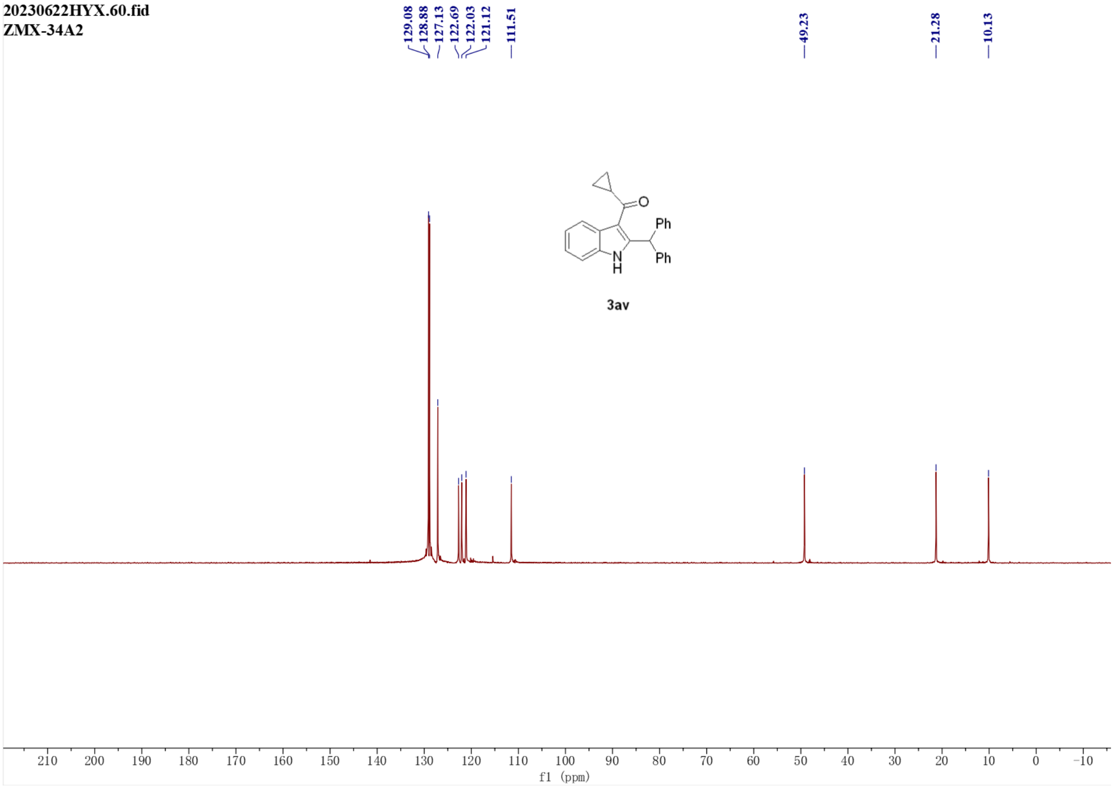
20230622HYX.60.fid
ZMX-34A2
129.08
128.88
127.13
122.69
122.03
121.12
111.51
49.23
21.28
10.13
Ph
Ph
O
N
H
3av
f1 (ppm)

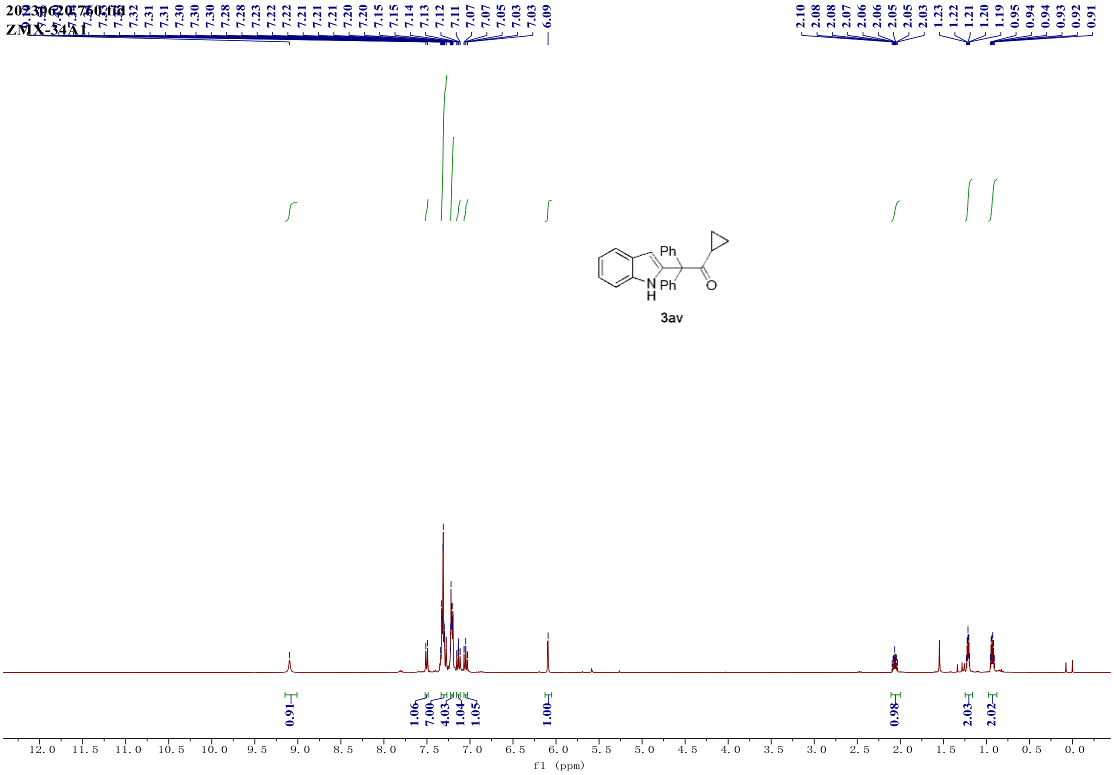
6.09
2.10
2.08
2.08
2.07
2.06
2.06
2.05
2.05
2.03
1.23
1.22
1.21
1.20
1.19
0.95
0.94
0.94
0.93
0.92
0.91
Ph
Ph
O
N
H
3av
0.91
1.06
7.00
4.03
1.04
1.05
1.00
0.98
2.03
2.02
f1 (ppm)

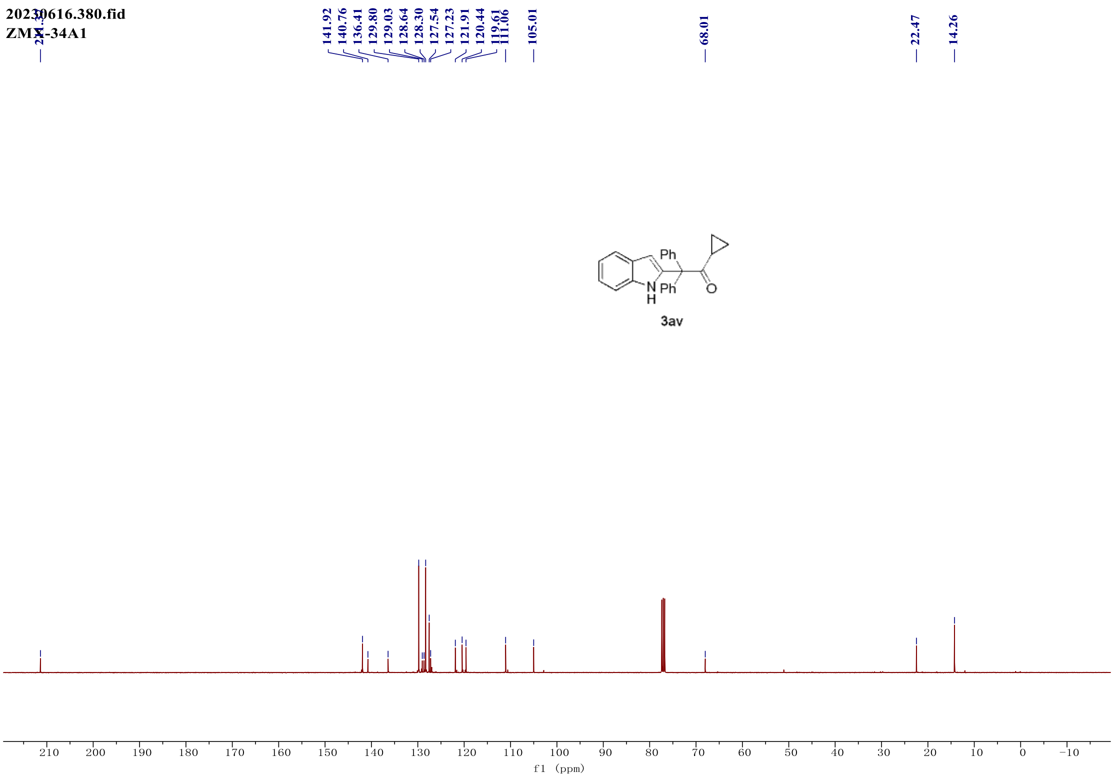
20230616.380.fid
ZMX-34A1
141.92
140.76
136.41
129.80
129.03
128.64
128.30
127.54
127.23
121.91
120.44
119.61
111.06
105.01
68.01
22.47
14.26
Ph
N
H
Ph
O
3av
f1 (ppm)

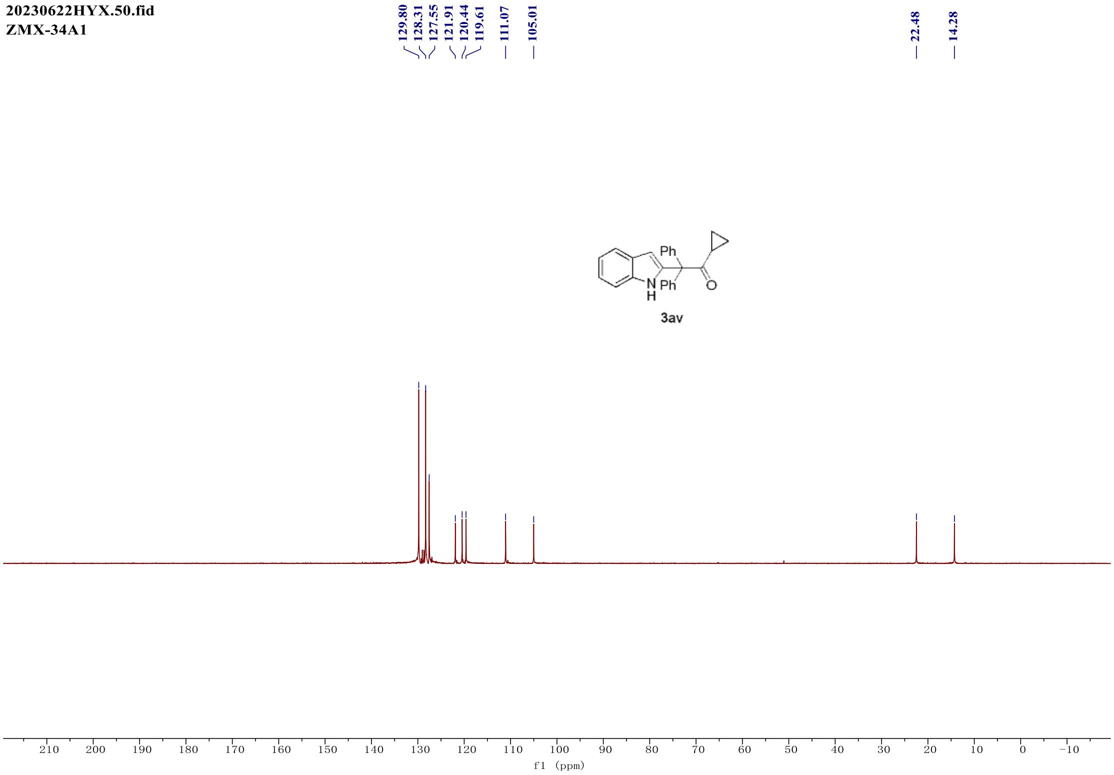
20230622HYX.50.fid
ZMX-34A1
129.80
128.31
127.55
121.91
120.44
119.61
111.07
105.01
22.48
14.28
Ph
N
H
Ph
O
3av
f1 (ppm)

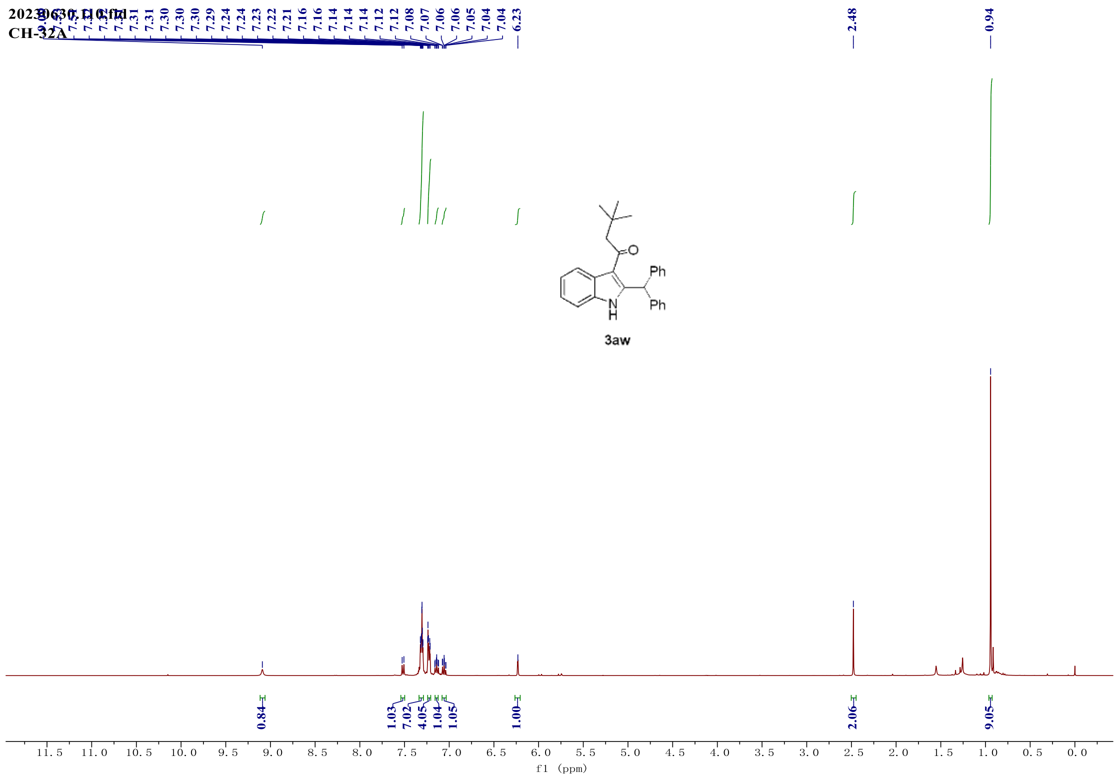
CH-
7.31
7.31
7.30
7.30
7.29
7.24
7.24
7.23
7.22
7.21
7.16
7.16
7.14
7.14
7.12
7.12
7.08
7.07
7.06
7.06
7.05
7.04
7.04
6.23
2.48
0.94
Ph
O
N
H
Ph
3aw
0.84
1.03
7.02
4.05
1.04
1.05
1.00
2.06
9.05
11.5 11.0 10.5 10.0 9.5 9.0 8.5 8.0 7.5 7.0 6.5 6.0 5.5 5.0 4.5 4.0 3.5 3.0 2.5 2.0 1.5 1.0 0.5 0.0
f1 (ppm)

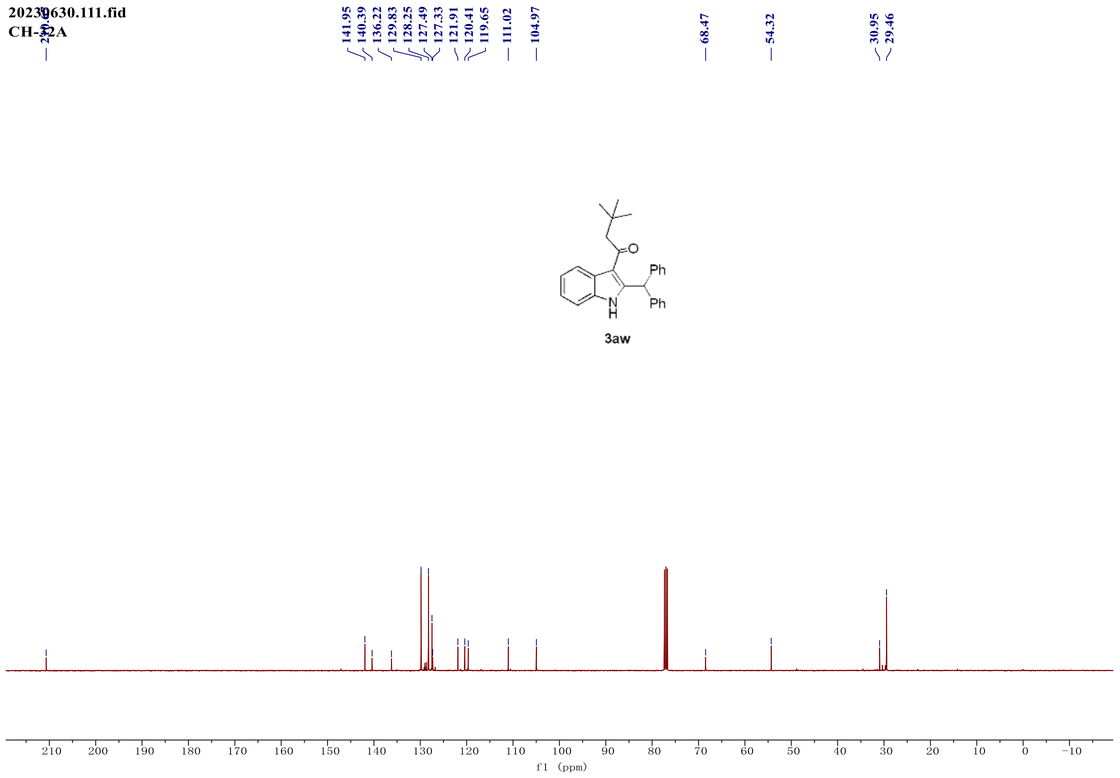
20230630.111.fid
CH-
141.95
140.39
136.22
129.83
128.25
127.49
127.33
121.91
120.41
119.65
111.02
104.97
68.47
54.32
30.95
29.46
Ph
O
N
H
Ph
3aw
210 200 190 180 170 160 150 140 130 120 110 100 90 80 70 60 50 40 30 20 10 0 -10
f1 (ppm)

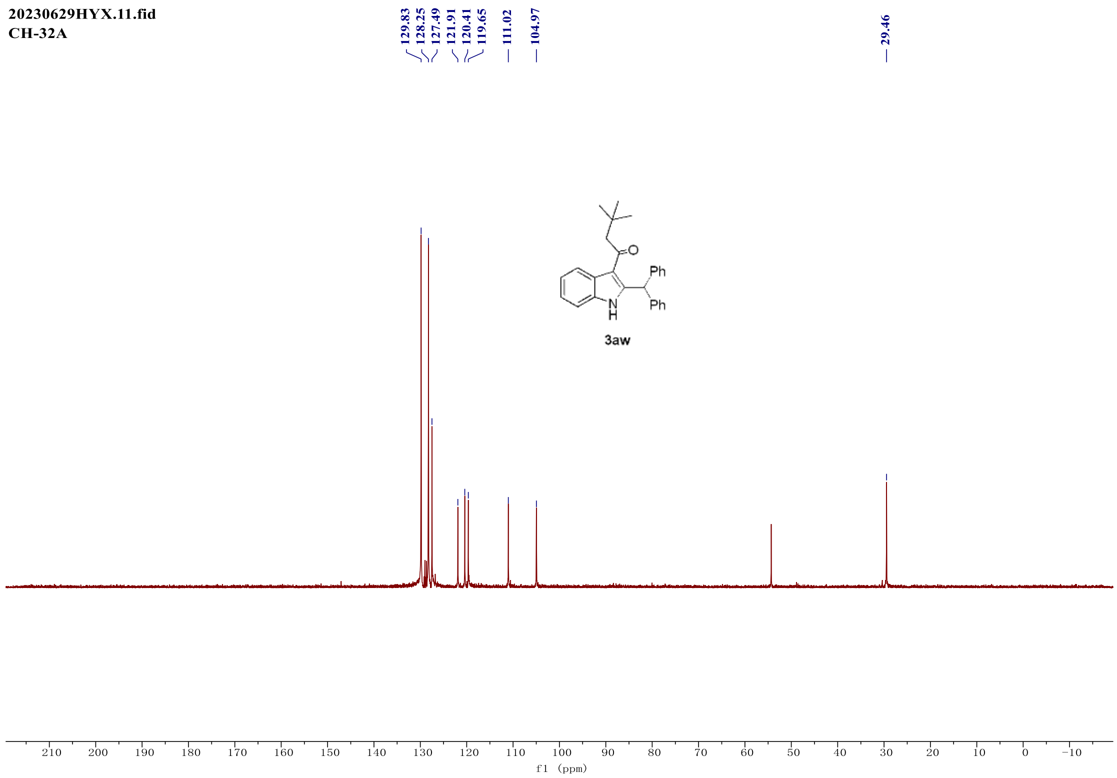
20230629HYX.11.fid
CH-32A
129.83
128.25
127.49
121.91
120.41
119.65
111.02
104.97
29.46
Ph
O
Ph
N
H
3aw
210 200 190 180 170 160 150 140 130 120 110 100 90 80 70 60 50 40 30 20 10 0 -10
f1 (ppm)

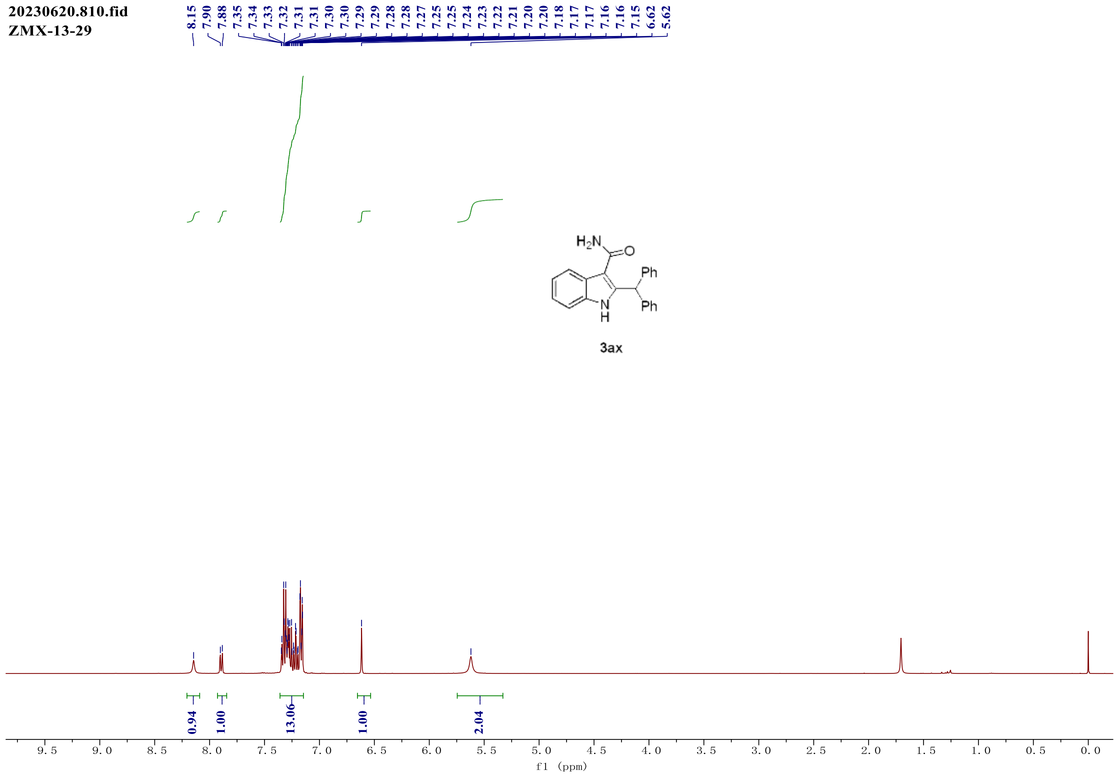
20230620.810.fid
ZMX-13-29
8.15
7.90
7.88
7.35
7.34
7.33
7.32
7.31
7.31
7.30
7.30
7.29
7.29
7.28
7.27
7.25
7.25
7.24
7.23
7.22
7.21
7.20
7.20
7.18
7.17
7.17
7.16
7.16
7.15
6.62
5.62
H2N
O
Ph
Ph
N
H
3ax
0.94
1.00
13.06
1.00
2.04
9.5 9.0 8.5 8.0 7.5 7.0 6.5 6.0 5.5 5.0 4.5 4.0 3.5 3.0 2.5 2.0 1.5 1.0 0.5 0.0
f1 (ppm)

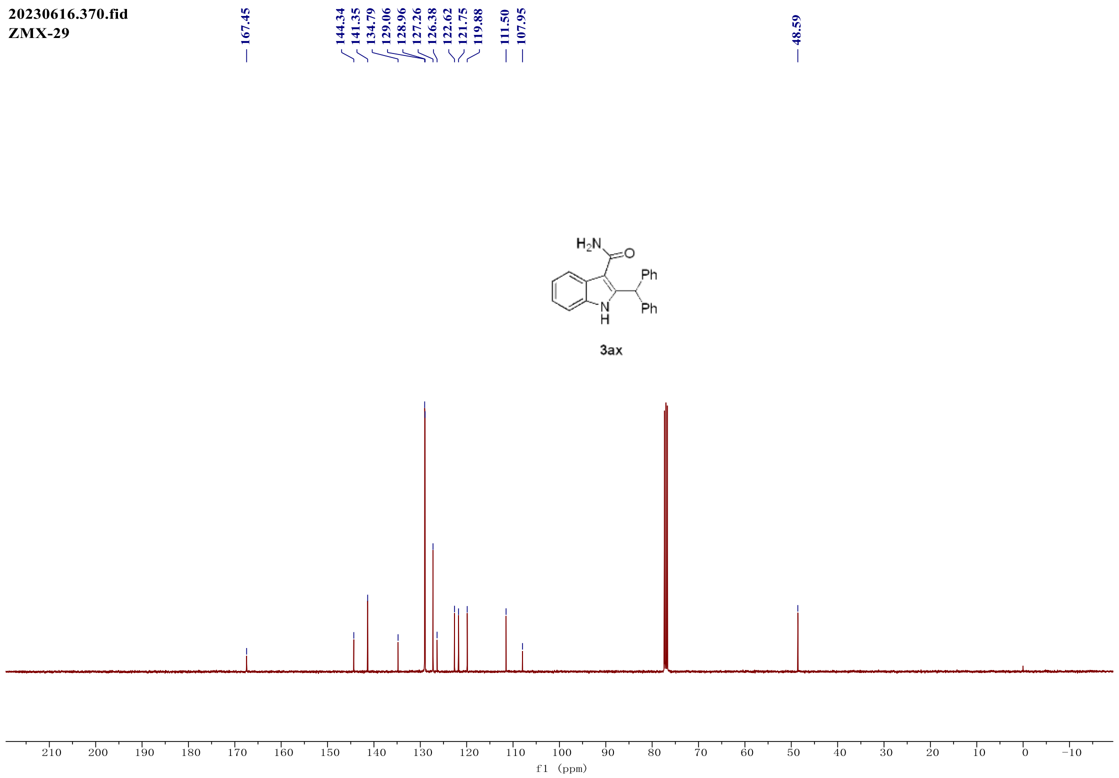
20230616.370.fid
ZMX-29
167.45
144.34
141.35
134.79
129.06
128.96
127.26
126.38
122.62
121.75
119.88
111.50
107.95
48.59
3ax
f1 (ppm)

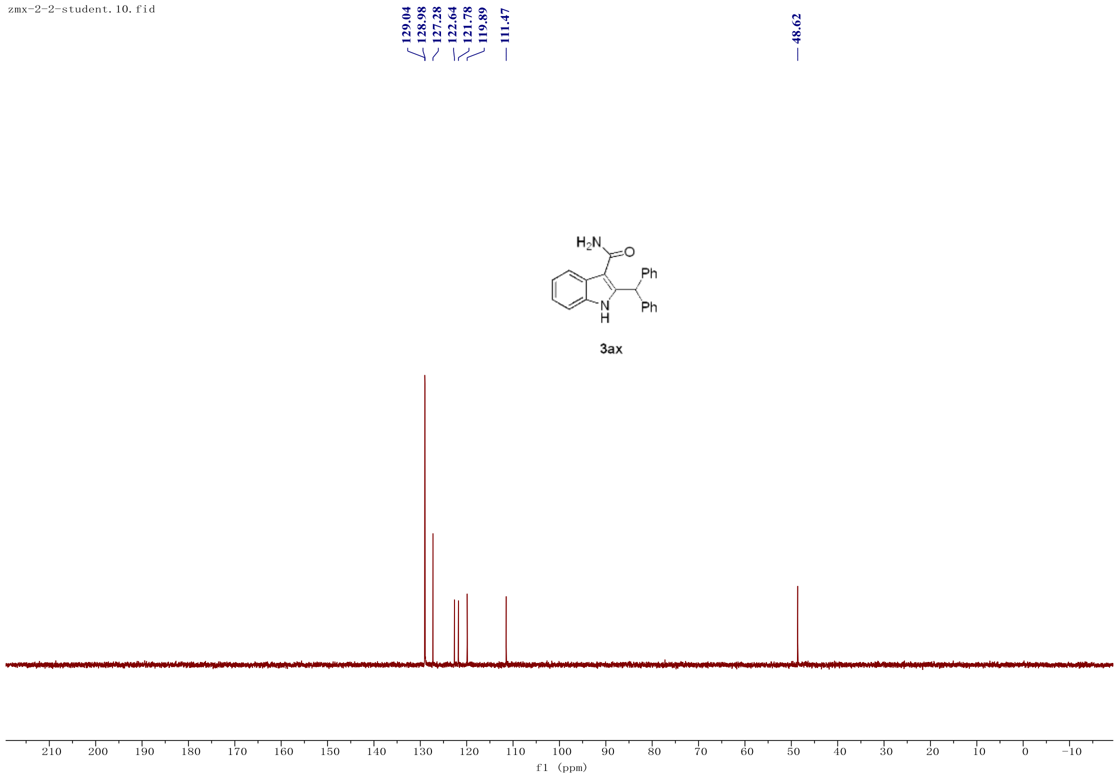
zmx-2-2-student.10.fid
129.04
128.98
127.28
122.64
121.78
119.89
111.47
48.62
3ax
f1 (ppm)

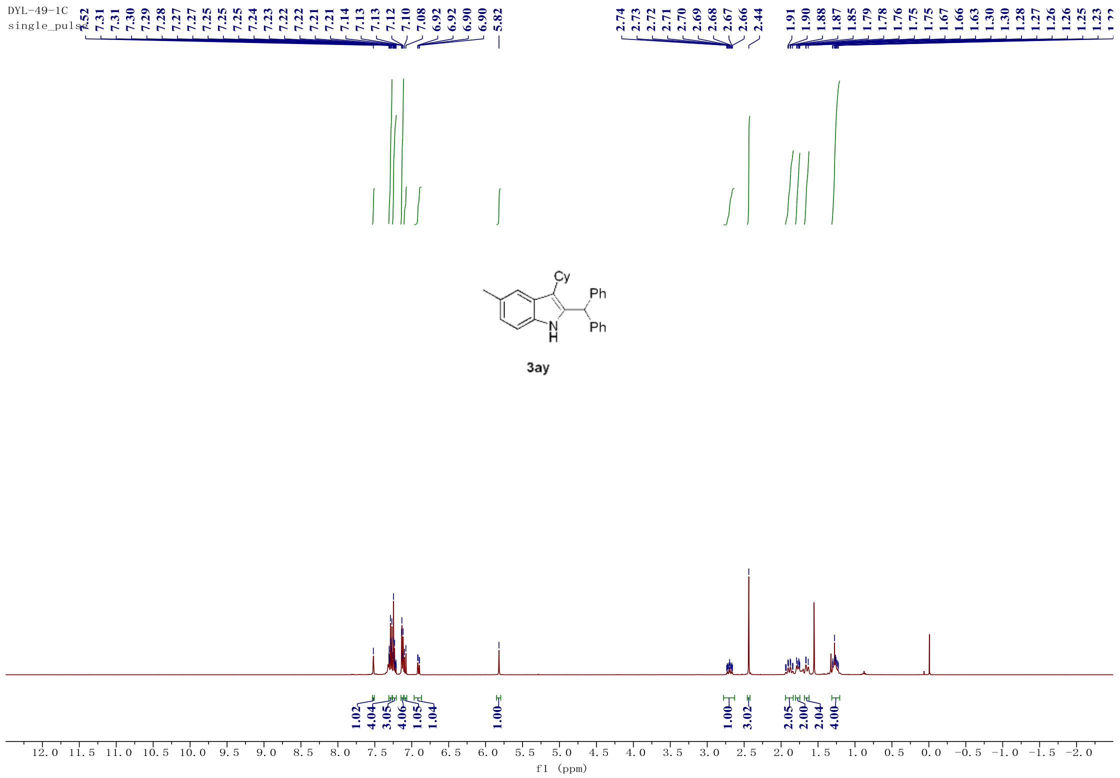
DYL-49-1C
single_pulse
7.52
7.31
7.30
7.29
7.28
7.27
7.27
7.25
7.25
7.24
7.23
7.22
7.22
7.21
7.21
7.14
7.13
7.12
7.10
7.08
6.92
6.92
6.90
6.90
5.82
2.74
2.73
2.72
2.71
2.70
2.69
2.68
2.67
2.66
2.44
1.91
1.90
1.88
1.87
1.85
1.79
1.78
1.76
1.75
1.75
1.67
1.66
1.63
1.30
1.28
1.27
1.26
1.26
1.25
1.23
Cy
Ph
N
H
Ph
3ay
1.02
4.04
3.05
4.06
1.05
1.04
1.00
1.00
3.02
2.05
2.00
2.04
4.00
12.0 11.5 11.0 10.5 10.0 9.5 9.0 8.5 8.0 7.5 7.0 6.5 6.0 5.5 5.0 4.5 4.0 3.5 3.0 2.5 2.0 1.5 1.0 0.5 0.0 -0.5 -1.0 -1.5 -2.0
f1 (ppm)

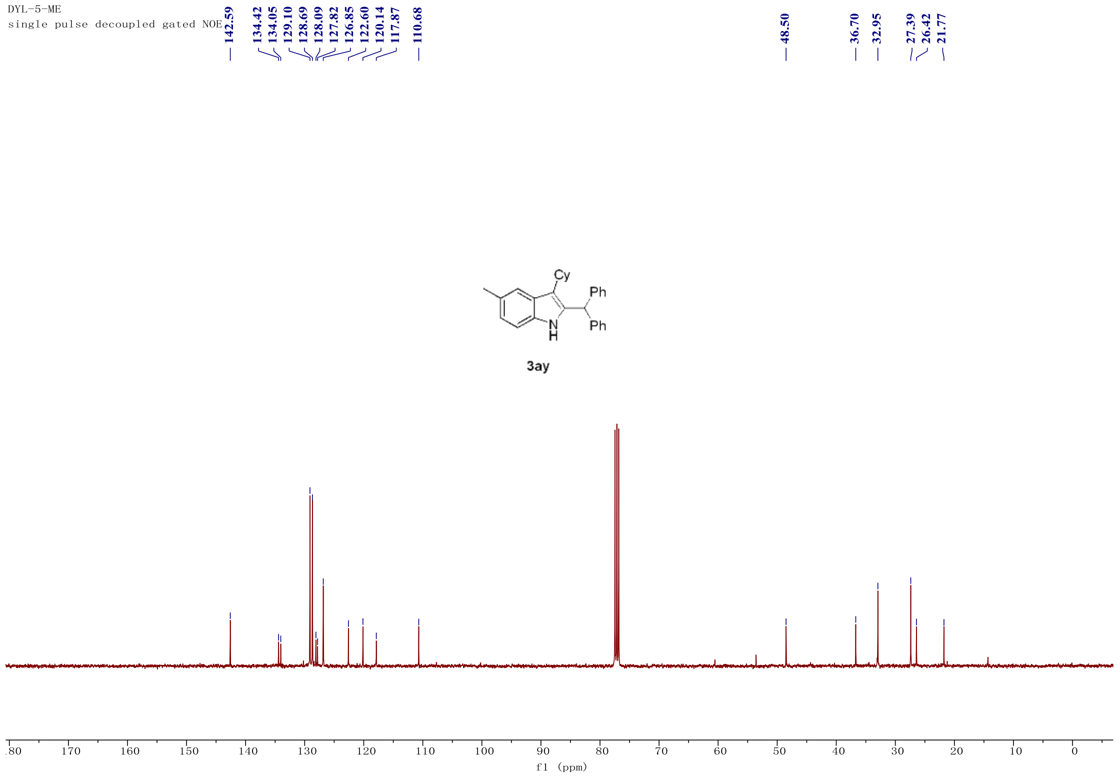
DYL-5-ME
single pulse decoupled gated NOE
142.59
134.42
134.05
129.10
128.69
128.09
127.82
126.85
122.60
120.14
117.87
110.68
48.50
36.70
32.95
27.39
26.42
21.77
Cy
Ph
N
H
Ph
3ay
180 170 160 150 140 130 120 110 100 90 80 70 60 50 40 30 20 10 0
f1 (ppm)

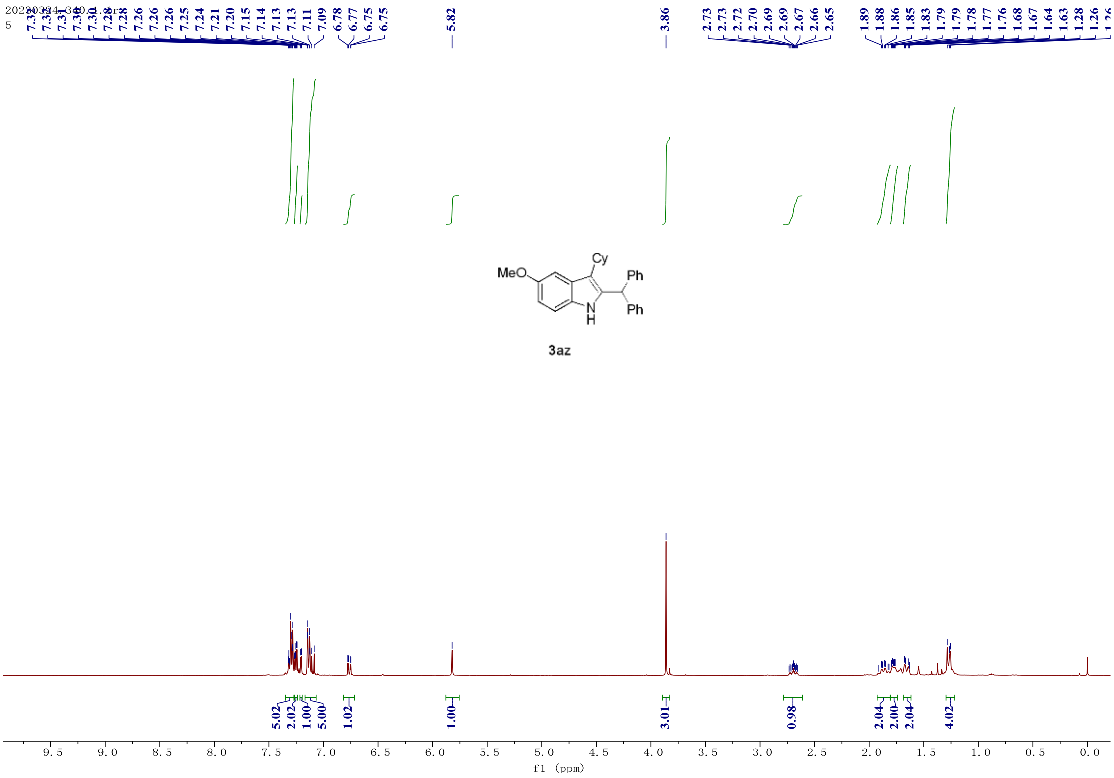
MeO
Cy
Ph
Ph
N
H
3az
5.02
2.02
1.00
5.00
1.02
1.00
3.01
0.98
2.04
2.00
2.04
4.02
f1 (ppm)

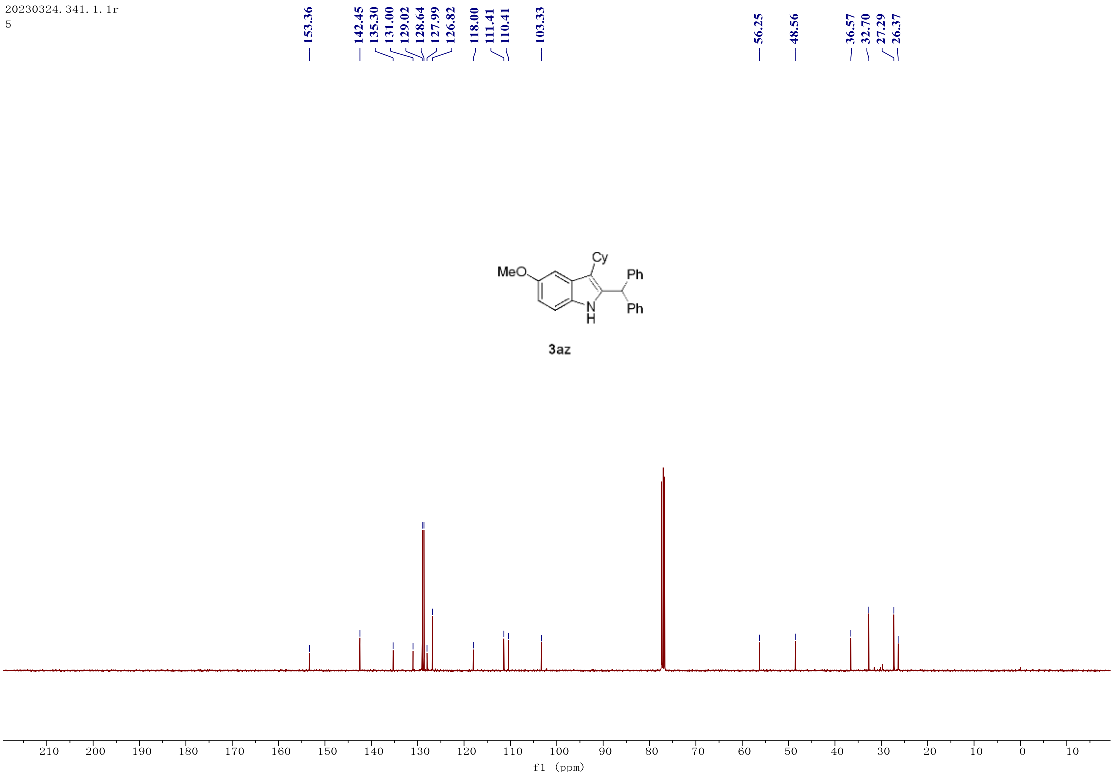
20230324.341.1.1r
5
153.36
142.45
135.30
131.00
129.02
128.64
127.99
126.82
118.00
111.41
110.41
103.33
56.25
48.56
36.57
32.70
27.29
26.37
MeO
Cy
Ph
Ph
N
H
3az
f1 (ppm)

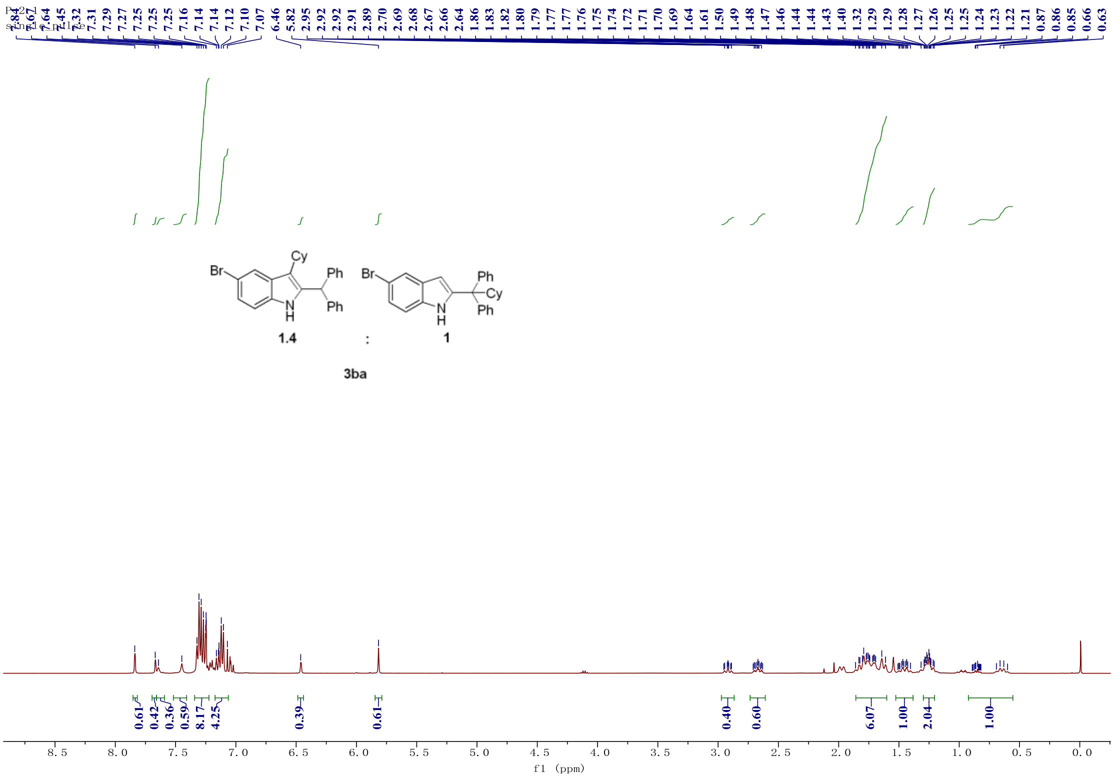
7.84
7.67
7.64
7.45
7.32
7.31
7.29
7.27
7.25
7.25
7.25
7.16
7.14
7.14
7.12
7.10
7.07
6.46
5.82
2.95
2.92
2.92
2.91
2.89
2.70
2.69
2.68
2.67
2.66
2.64
1.86
1.83
1.82
1.80
1.79
1.77
1.77
1.76
1.75
1.74
1.72
1.71
1.70
1.69
1.64
1.61
1.50
1.49
1.48
1.47
1.46
1.44
1.44
1.43
1.40
1.32
1.29
1.29
1.28
1.27
1.26
1.25
1.24
1.23
1.22
1.21
0.87
0.86
0.85
0.66
0.63
Br
Cy
Ph
Ph
N
H
Br
Ph
Cy
Ph
N
H
1.4
:
1
3ba
0.61
0.42
0.36
0.59
8.17
4.25
0.39
0.61
0.40
0.60
6.07
1.00
2.04
1.00
8.5
8.0
7.5
7.0
6.5
6.0
5.5
5.0
4.5
4.0
3.5
3.0
2.5
2.0
1.5
1.0
0.5
0.0
f1 (ppm)

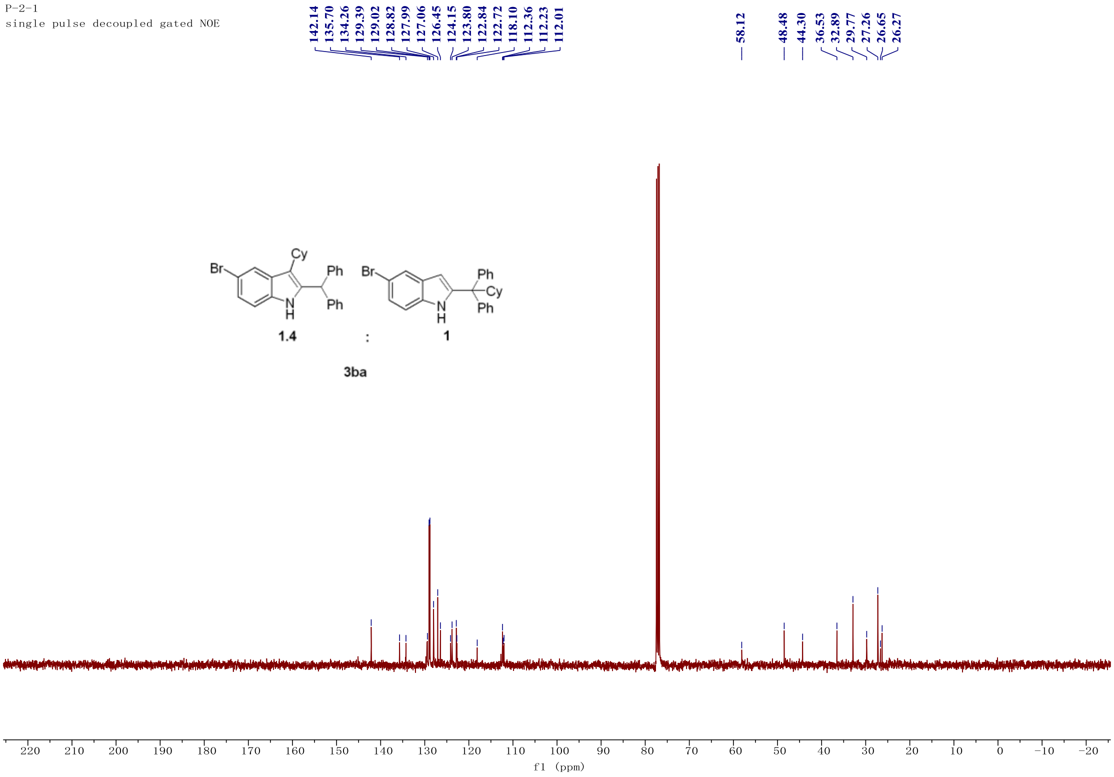
P-2-1
single pulse decoupled gated NOE
142.14
135.70
134.26
129.39
129.02
128.82
127.99
127.06
126.45
124.15
123.80
122.84
122.72
118.10
112.36
112.23
112.01
58.12
48.48
44.30
36.53
32.89
29.77
27.26
26.65
26.27
Br
Cy
Ph
Ph
N
H
Br
Ph
Cy
Ph
N
H
1.4
:
1
3ba
220
210
200
190
180
170
160
150
140
130
120
110
100
90
80
70
60
50
40
30
20
10
0
-10
-20
f1 (ppm)

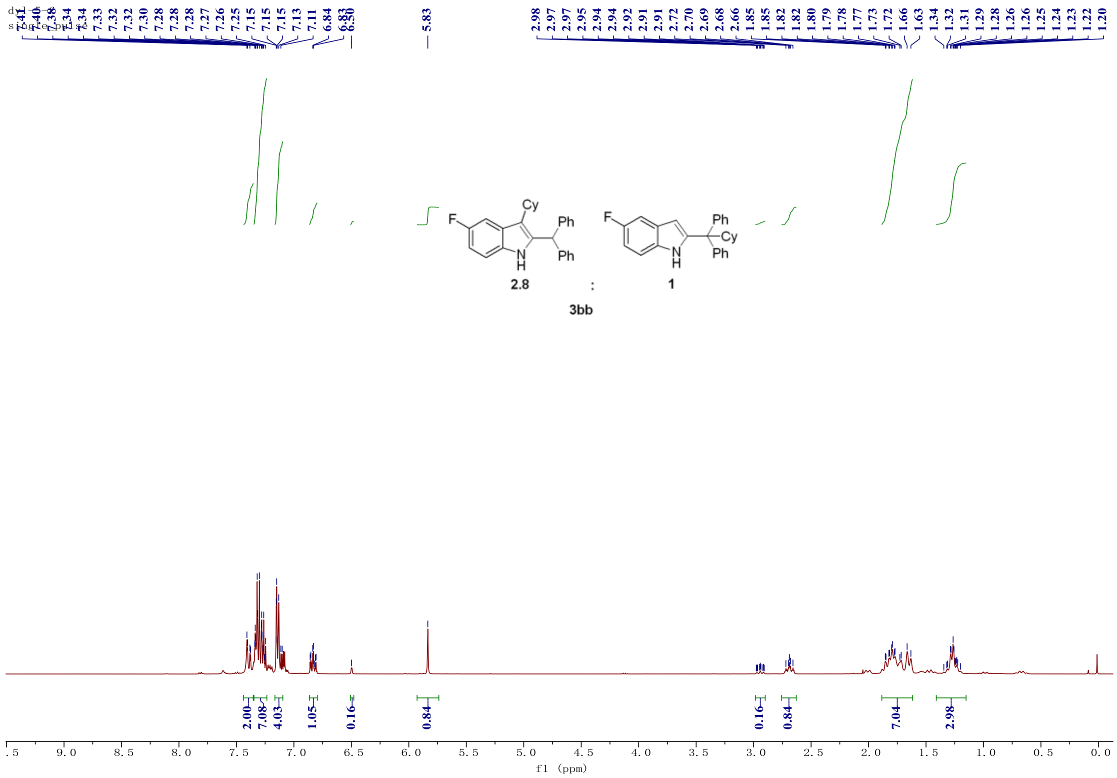
dyl-5-f
single pulse
7.41 7.40 7.38 7.34 7.34 7.33 7.32 7.32 7.30 7.28 7.28 7.28 7.27 7.26 7.25 7.15 7.15 7.15 7.13 7.11 6.84 6.83 6.50
5.83
2.98 2.97 2.97 2.95 2.94 2.94 2.92 2.91 2.91 2.72 2.70 2.69 2.68 2.66 1.85 1.85 1.82 1.82 1.80 1.79 1.78 1.77 1.73 1.72 1.66 1.63 1.34 1.32 1.31 1.29 1.28 1.26 1.26 1.25 1.24 1.23 1.22 1.20
F
Cy
Ph
Ph
N
H
2.8
:
F
Ph
Cy
Ph
N
H
1
3bb
2.00 7.08 4.03 1.05 0.16 0.84 0.16 0.84 7.04 2.98
f1 (ppm)

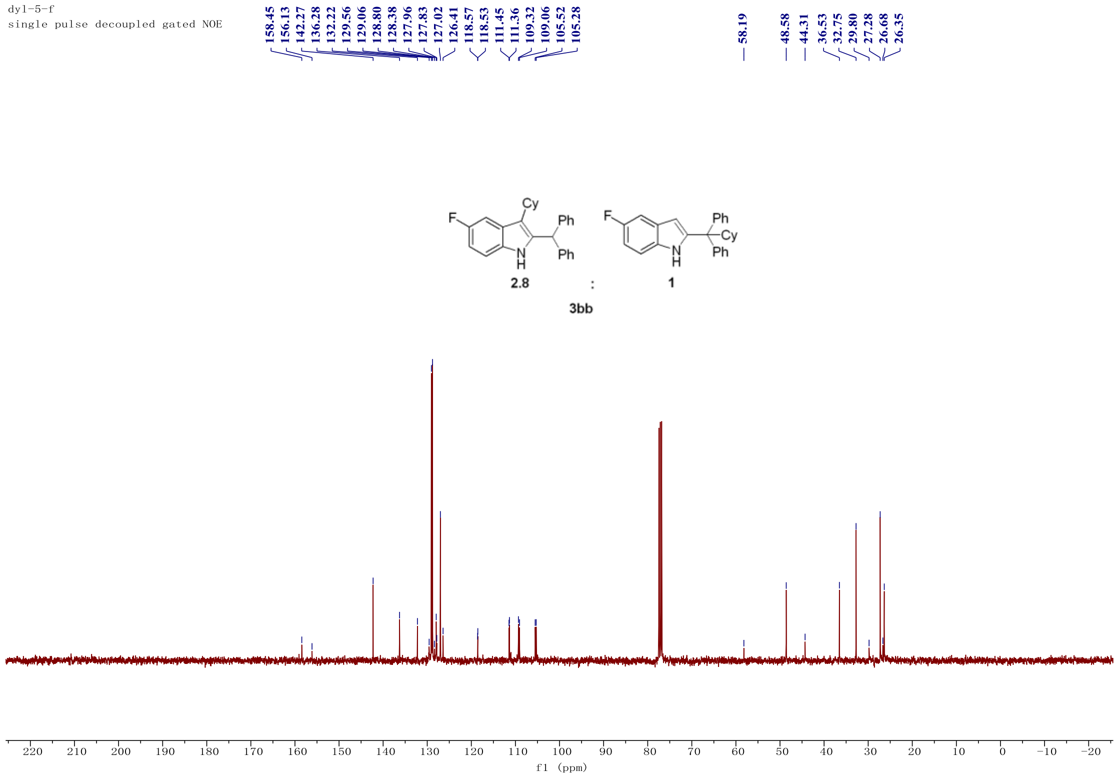
dyl-5-f
single pulse decoupled gated NOE
158.45 156.13 142.27 136.28 132.22 129.56 129.06 128.80 128.38 127.96 127.83 127.02 126.41 118.57 118.53 111.45 111.36 109.32 109.06 105.52 105.28
58.19
48.58 44.31 36.53 32.75 29.80 27.28 26.68 26.35
F
Cy
Ph
Ph
N
H
2.8
:
F
Ph
Cy
Ph
N
H
1
3bb
f1 (ppm)

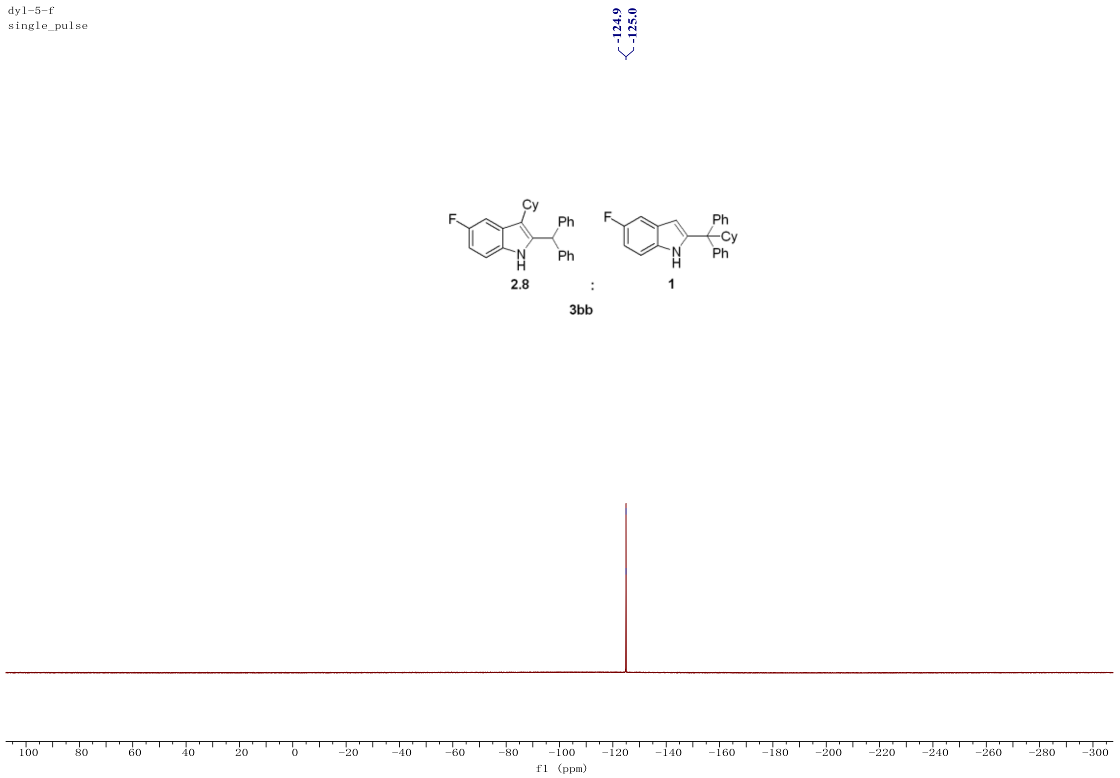
dyl-5-f
single_pulse
-124.9
-125.0
Cy
F
Ph
Ph
N
H
2.8
:
F
Ph
Cy
Ph
N
H
1
3bb
100 80 60 40 20 0 −20 −40 −60 −80 −100 −120 −140 −160 −180 −200 −220 −240 −260 −280 −300
f1 (ppm)

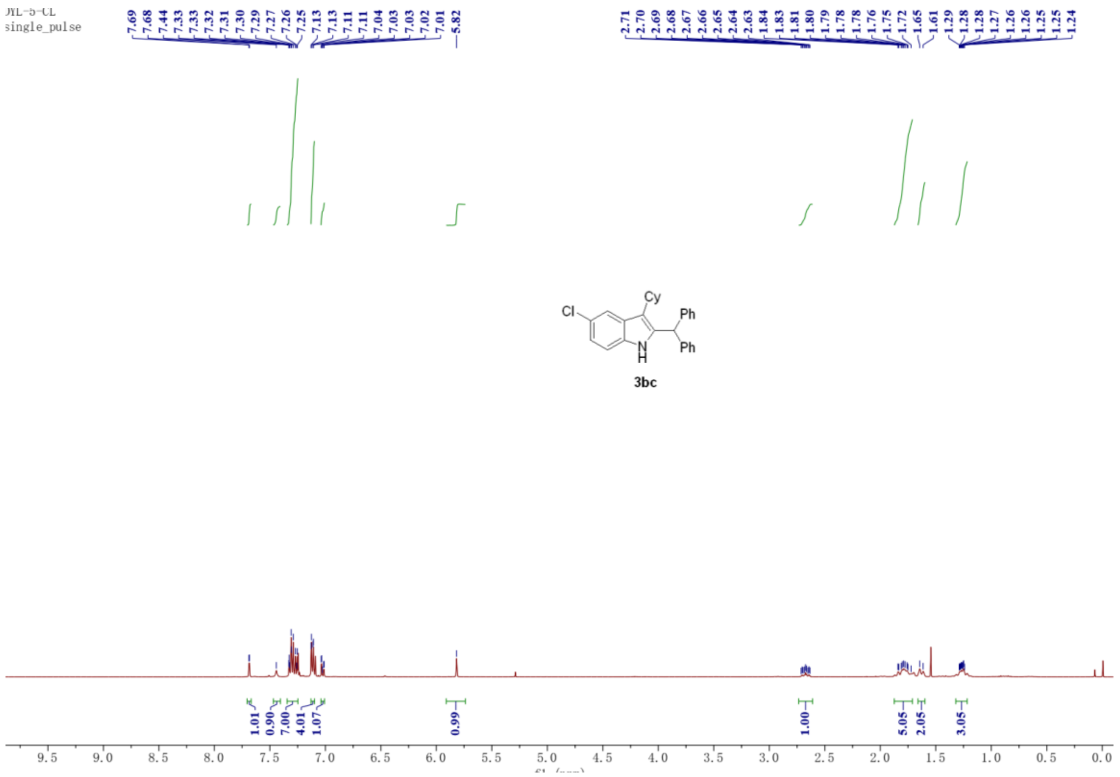
single_pulse
7.69
7.68
7.44
7.33
7.32
7.31
7.30
7.29
7.27
7.26
7.25
7.13
7.13
7.11
7.11
7.04
7.03
7.03
7.02
7.01
5.82
2.71
2.70
2.69
2.68
2.67
2.66
2.65
2.64
2.63
1.84
1.83
1.81
1.80
1.79
1.78
1.78
1.76
1.75
1.72
1.65
1.61
1.29
1.28
1.27
1.26
1.26
1.25
1.25
1.24
Cl
Cy
Ph
Ph
N
H
3bc
1.01
0.90
7.00
4.01
1.07
0.99
1.00
5.05
2.05
3.05
9.5 9.0 8.5 8.0 7.5 7.0 6.5 6.0 5.5 5.0 4.5 4.0 3.5 3.0 2.5 2.0 1.5 1.0 0.5 0.0

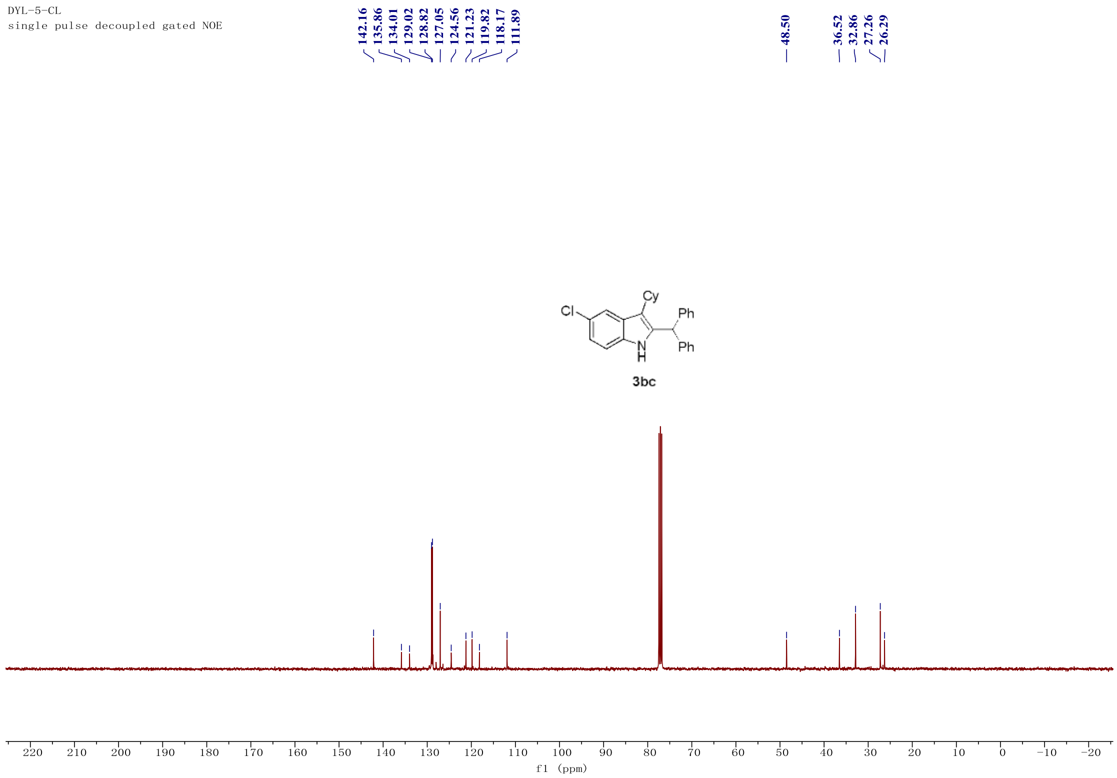
DYL-5-CL
single pulse decoupled gated NOE
142.16
135.86
134.01
129.02
128.82
127.05
124.56
121.23
119.82
118.17
111.89
48.50
36.52
32.86
27.26
26.29
Cl
Cy
Ph
Ph
N
H
3bc
220 210 200 190 180 170 160 150 140 130 120 110 100 90 80 70 60 50 40 30 20 10 0 -10 -20
f1 (ppm)

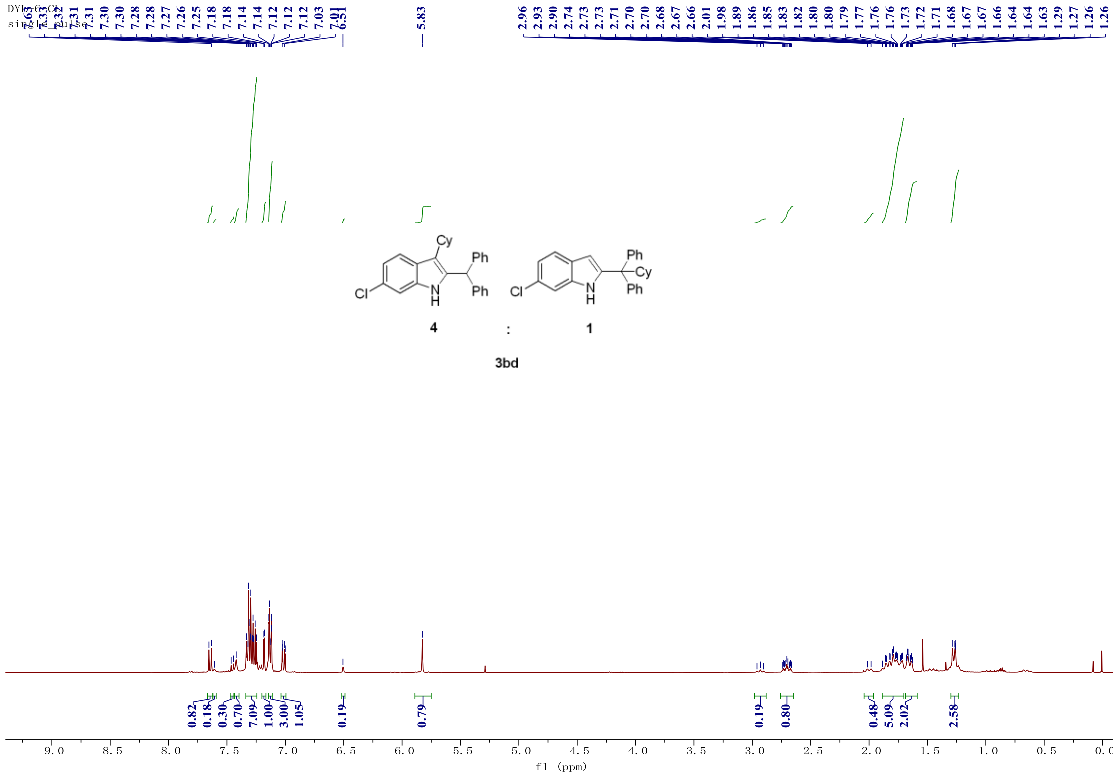
5.83
2.96
2.93
2.90
2.74
2.73
2.73
2.71
2.70
2.70
2.68
2.67
2.66
2.01
1.98
1.89
1.86
1.85
1.83
1.82
1.80
1.79
1.77
1.76
1.76
1.73
1.72
1.71
1.68
1.67
1.66
1.64
1.64
1.63
1.29
1.27
1.26
1.26
Cy
Ph
Ph
Cl
N
H
4
:
1
3bd
0.82
0.18
0.30
0.70
7.09
1.00
3.00
1.05
0.19
0.79
0.19
0.80
0.48
5.09
2.02
2.58
9.0 8.5 8.0 7.5 7.0 6.5 6.0 5.5 5.0 4.5 4.0 3.5 3.0 2.5 2.0 1.5 1.0 0.5 0.0
f1 (ppm)

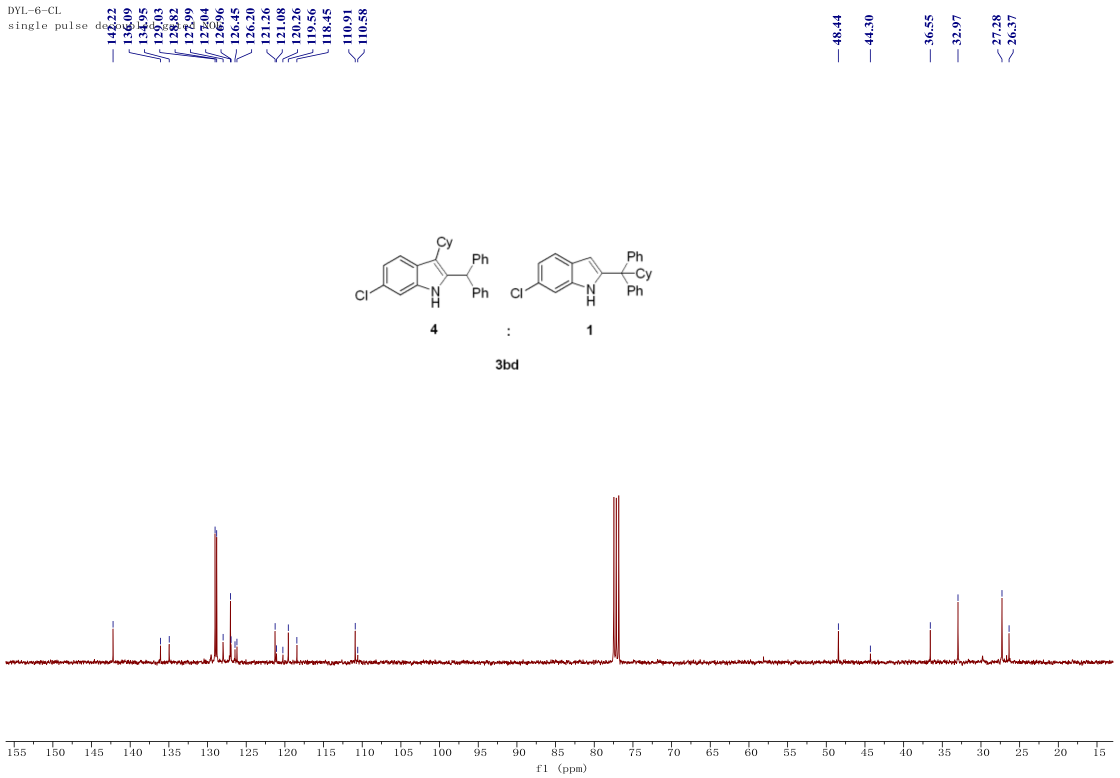
DYL-6-CL
single pulse decoupled gated NOE
142.22
136.09
134.95
129.03
128.82
127.99
127.04
126.96
126.45
126.20
121.26
121.08
120.26
119.56
118.45
110.91
110.58
48.44
44.30
36.55
32.97
27.28
26.37
Cy
Ph
Ph
Cl
N
H
Ph
Cy
Ph
Cl
N
H
4
:
1
3bd
155 150 145 140 135 130 125 120 115 110 105 100 95 90 85 80 75 70 65 60 55 50 45 40 35 30 25 20 15
f1 (ppm)

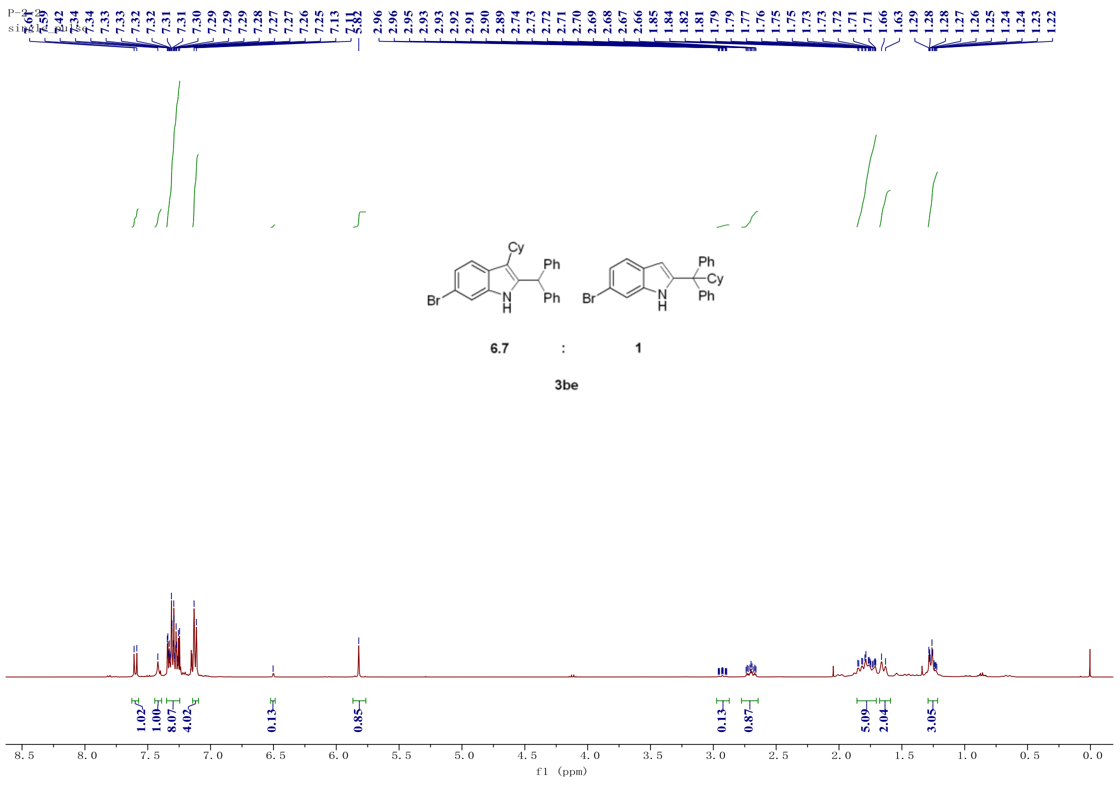
7.61
7.59
7.42
7.34
7.34
7.33
7.33
7.32
7.32
7.31
7.31
7.30
7.29
7.29
7.29
7.28
7.27
7.27
7.26
7.25
7.13
7.11
5.82
2.96
2.96
2.95
2.93
2.93
2.92
2.91
2.90
2.89
2.74
2.73
2.72
2.71
2.70
2.69
2.68
2.67
2.66
1.85
1.84
1.82
1.81
1.79
1.79
1.77
1.76
1.75
1.75
1.73
1.73
1.72
1.71
1.71
1.66
1.63
1.29
1.28
1.28
1.27
1.26
1.25
1.24
1.24
1.23
1.22
Cy
Ph
Ph
Br
N
H
Ph
Cy
Ph
Br
N
H
6.7
:
1
3be
1.02
1.00
8.07
4.02
0.13
0.85
0.13
0.87
5.09
2.04
3.05
8.5 8.0 7.5 7.0 6.5 6.0 5.5 5.0 4.5 4.0 3.5 3.0 2.5 2.0 1.5 1.0 0.5 0.0
f1 (ppm)

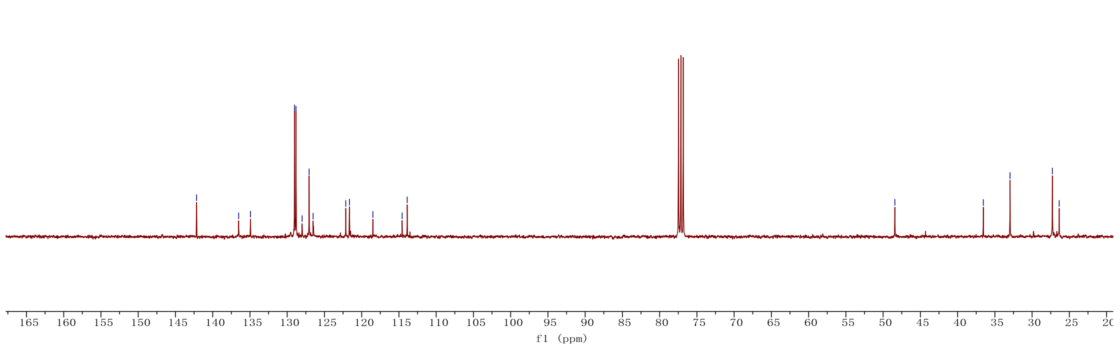

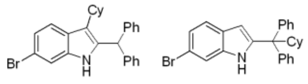

6.7 : 1

3be

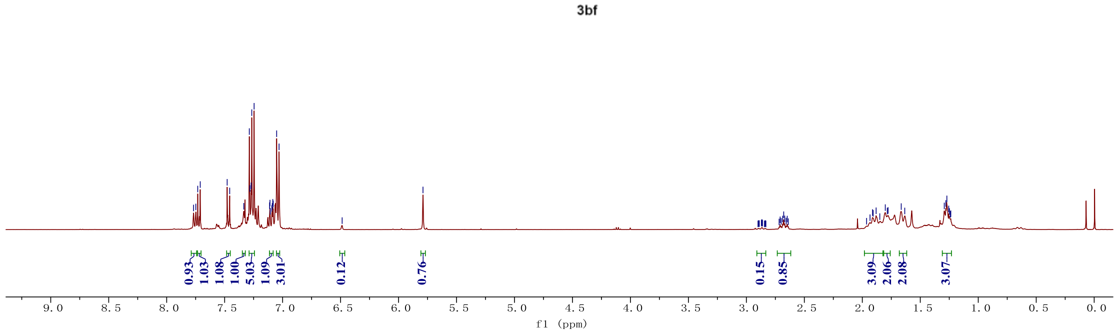

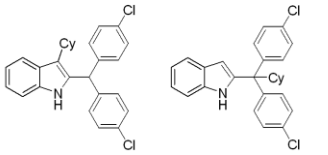

5.6 : 1

3bf

DYL-P-CL
single pulse decoupled gated NOE

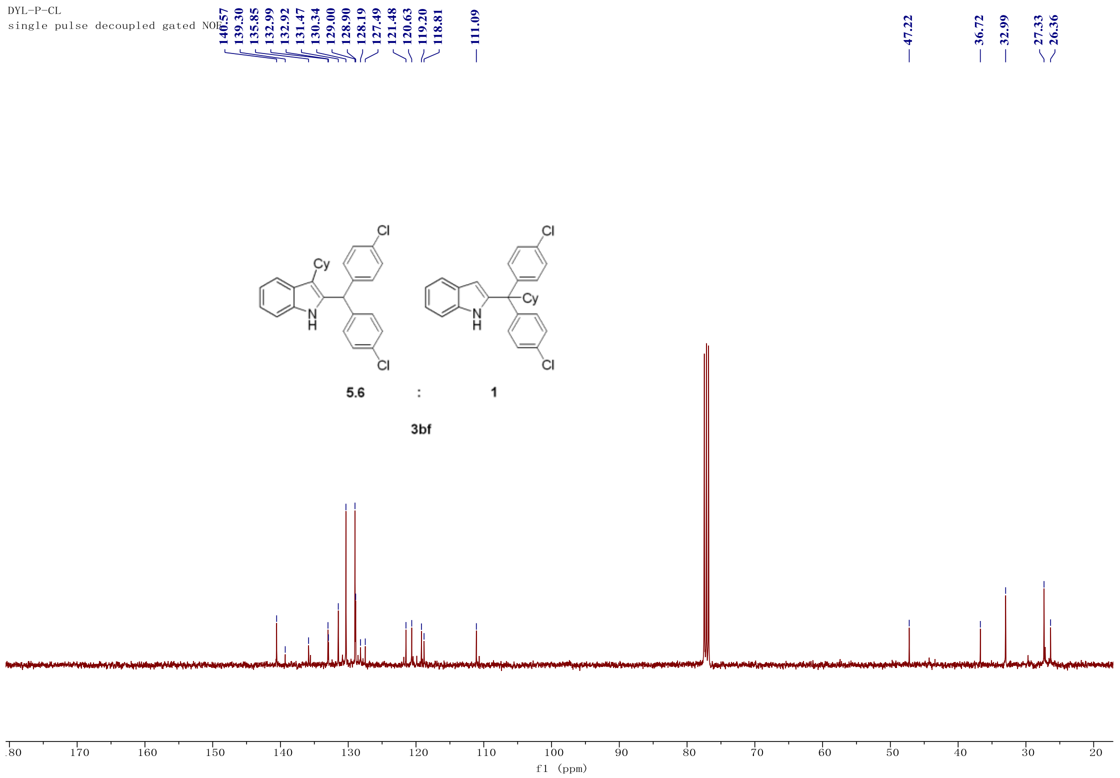

DYL-P-F
single_pulse

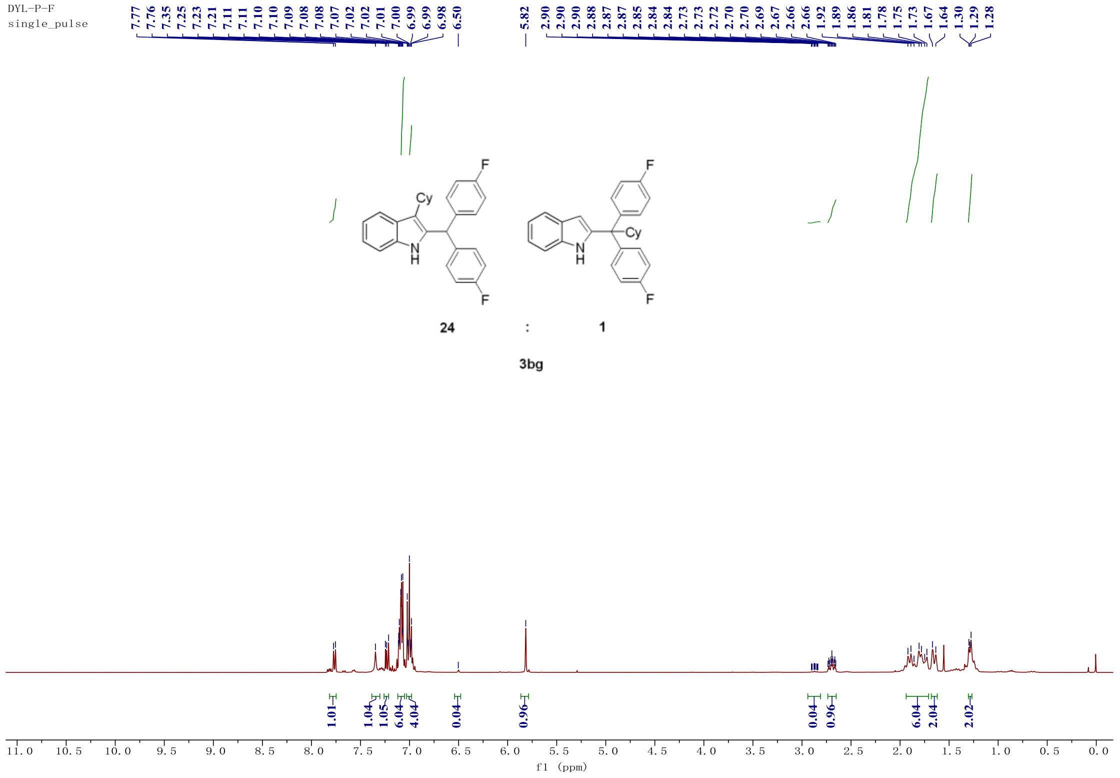

DYL-P-F
single pulse decoupled gated NOE

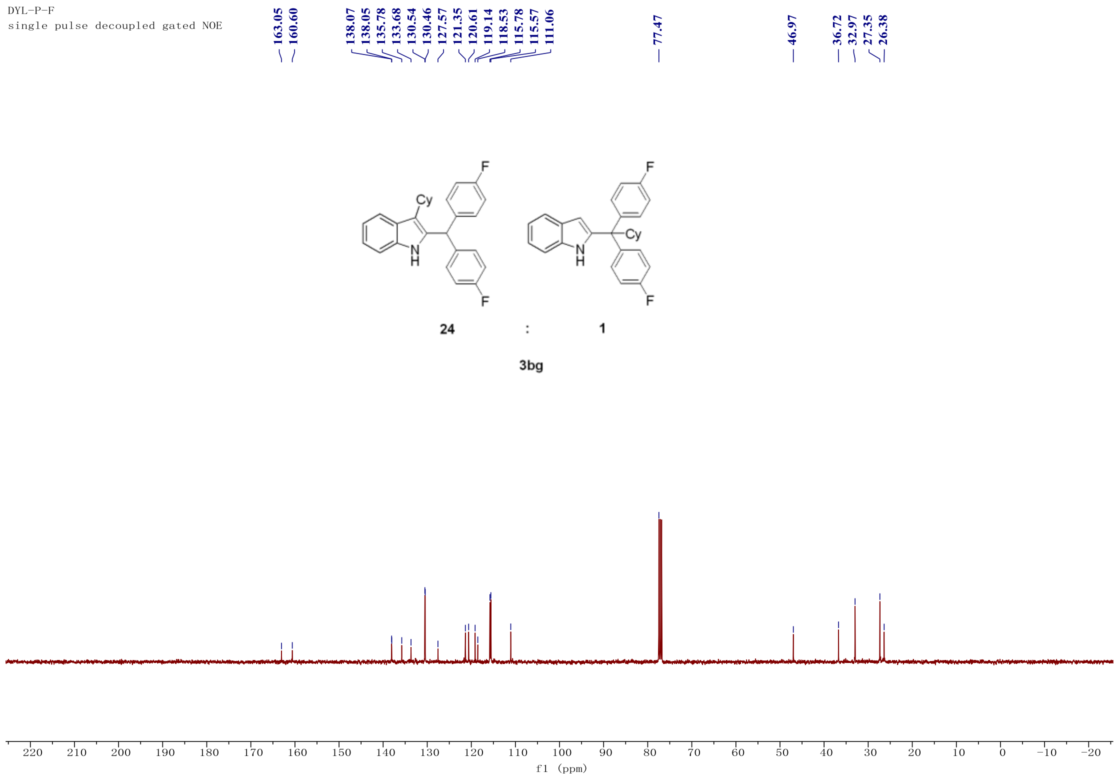

DYL-P-F
single_pulse

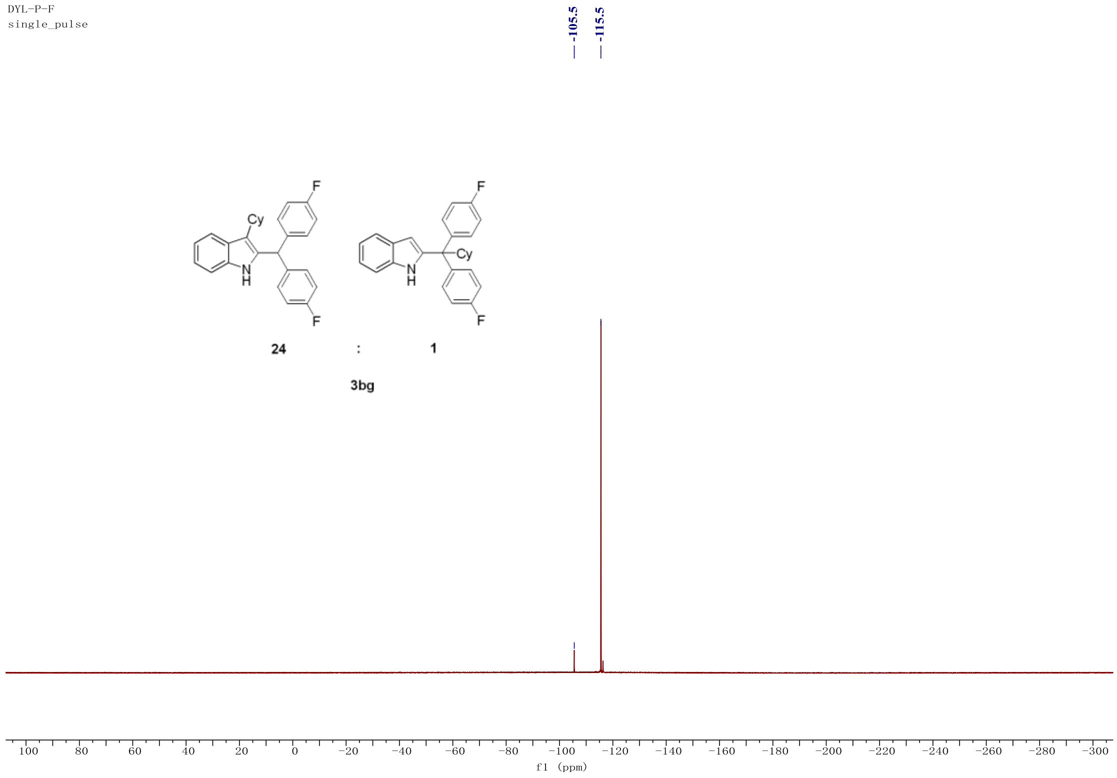

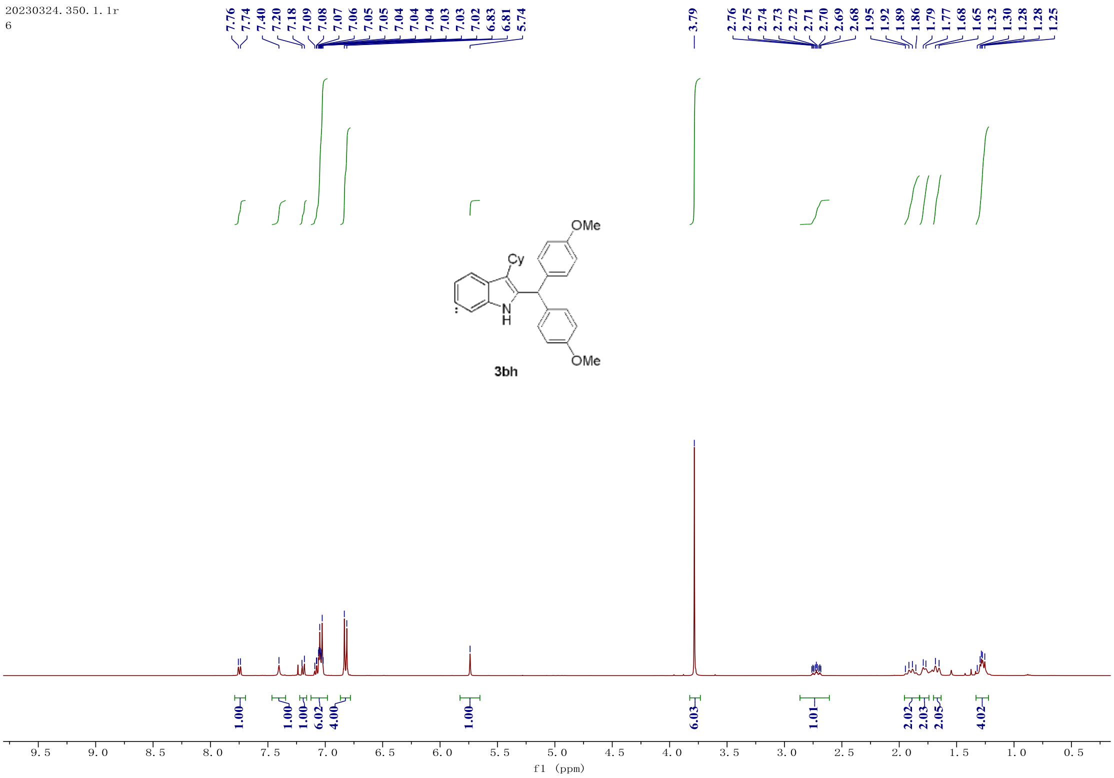
20230324.350.1.1r
6
7.76
7.74
7.40
7.20
7.18
7.09
7.08
7.07
7.06
7.05
7.05
7.04
7.04
7.04
7.03
7.03
7.02
6.83
6.81
5.74
3.79
2.76
2.75
2.74
2.73
2.72
2.71
2.70
2.69
2.68
1.95
1.92
1.89
1.86
1.79
1.77
1.68
1.65
1.32
1.30
1.28
1.28
1.25
OMe
Cy
N
H
OMe
3bh
1.00
1.00
1.00
6.02
4.00
1.00
6.03
1.01
2.02
2.03
2.05
4.02
f1 (ppm)

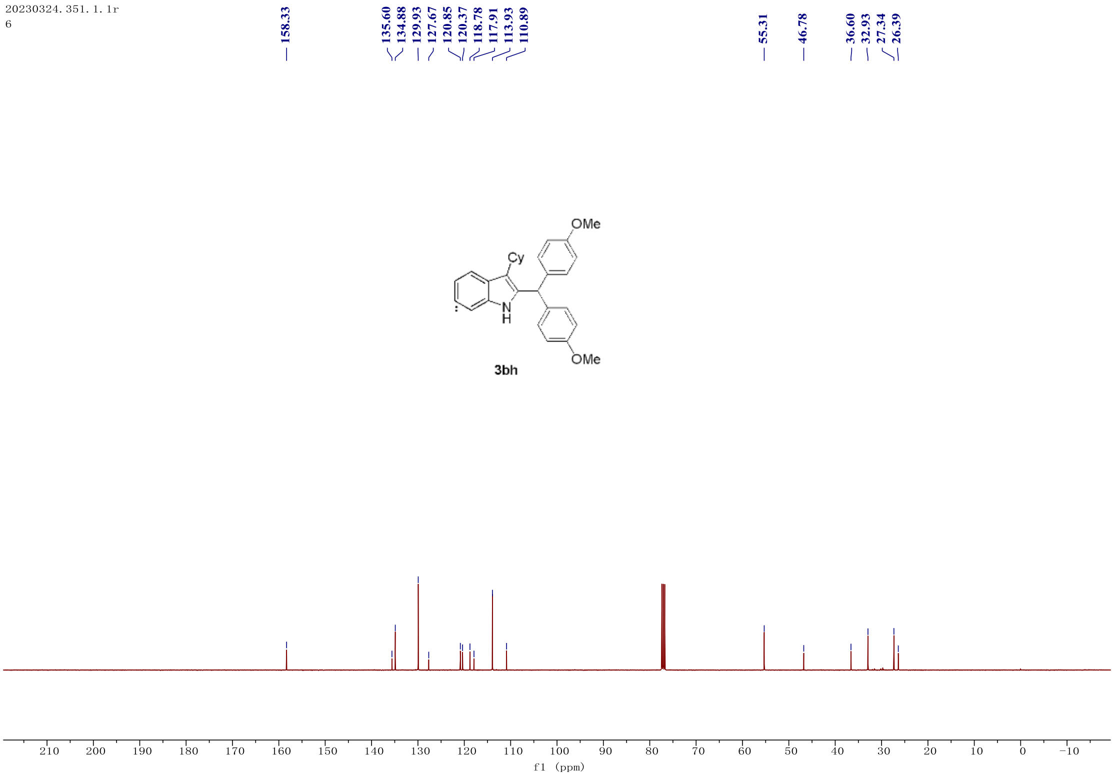
20230324.351.1.1r
6
158.33
135.60
134.88
129.93
127.67
120.85
120.37
118.78
117.91
113.93
110.89
55.31
46.78
36.60
32.93
27.34
26.39
OMe
Cy
N
H
OMe
3bh
f1 (ppm)

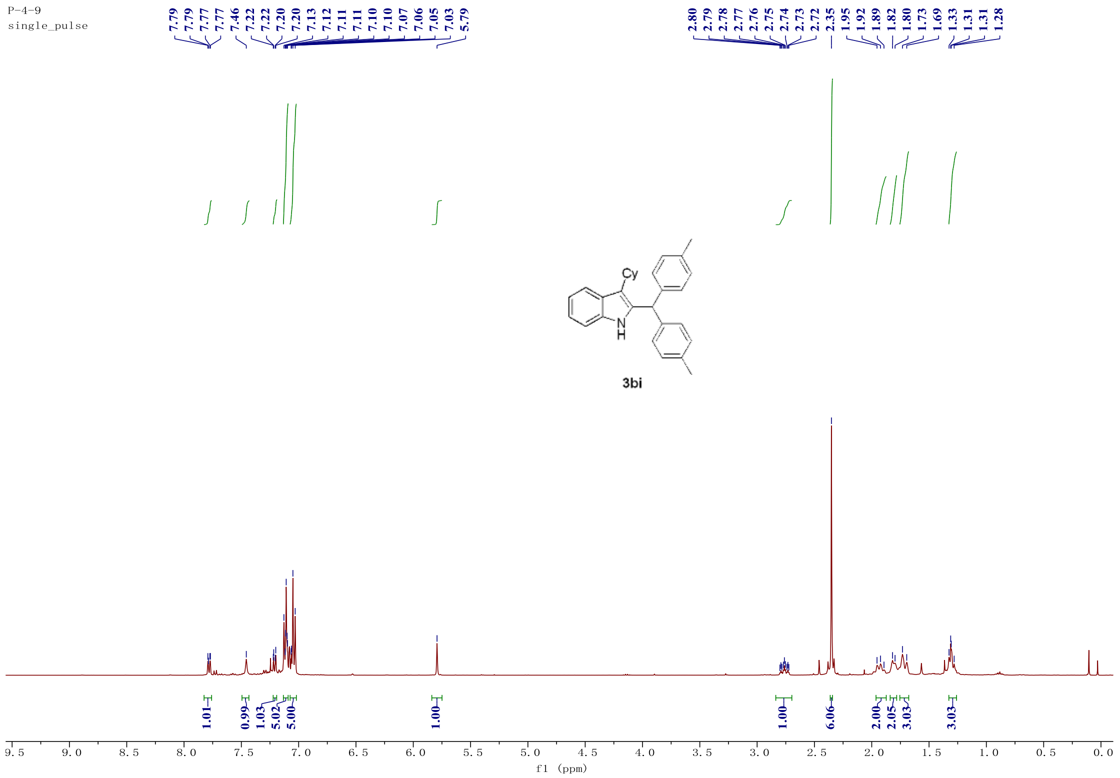
P-4-9
single_pulse
7.79 7.79 7.77 7.77 7.46 7.22 7.22 7.20 7.20 7.13 7.12 7.11 7.11 7.10 7.10 7.07 7.06 7.05 7.03 5.79
2.80 2.79 2.78 2.77 2.76 2.75 2.74 2.73 2.72 2.35 1.95 1.92 1.89 1.82 1.80 1.73 1.69 1.33 1.31 1.31 1.28
Cy
H
N
3bi
1.01 0.99 1.03 5.02 5.00 1.00 1.00 6.06 2.00 2.05 3.03 3.03
f1 (ppm)

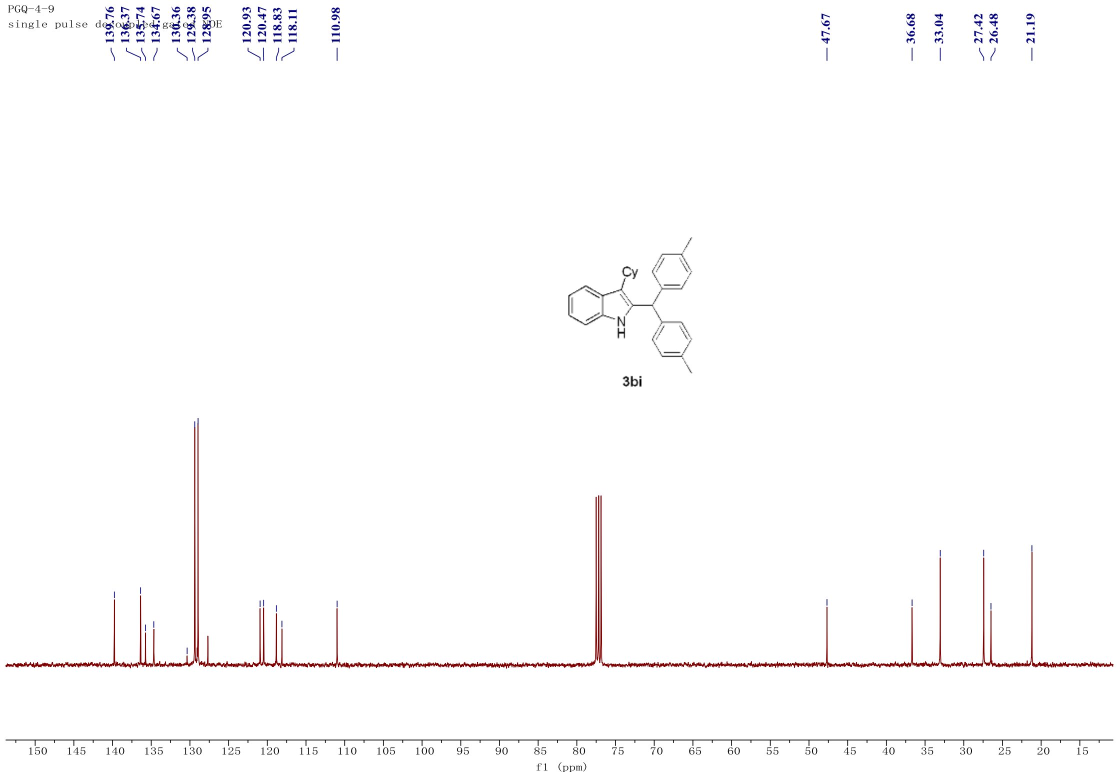
PGQ-4-9
single pulse decoupled gated NOE
139.76 136.37 135.74 134.67 130.36 129.38 128.95 120.93 120.47 118.83 118.11 110.98
47.67 36.68 33.04 27.42 26.48 21.19
Cy
H
N
3bi
f1 (ppm)

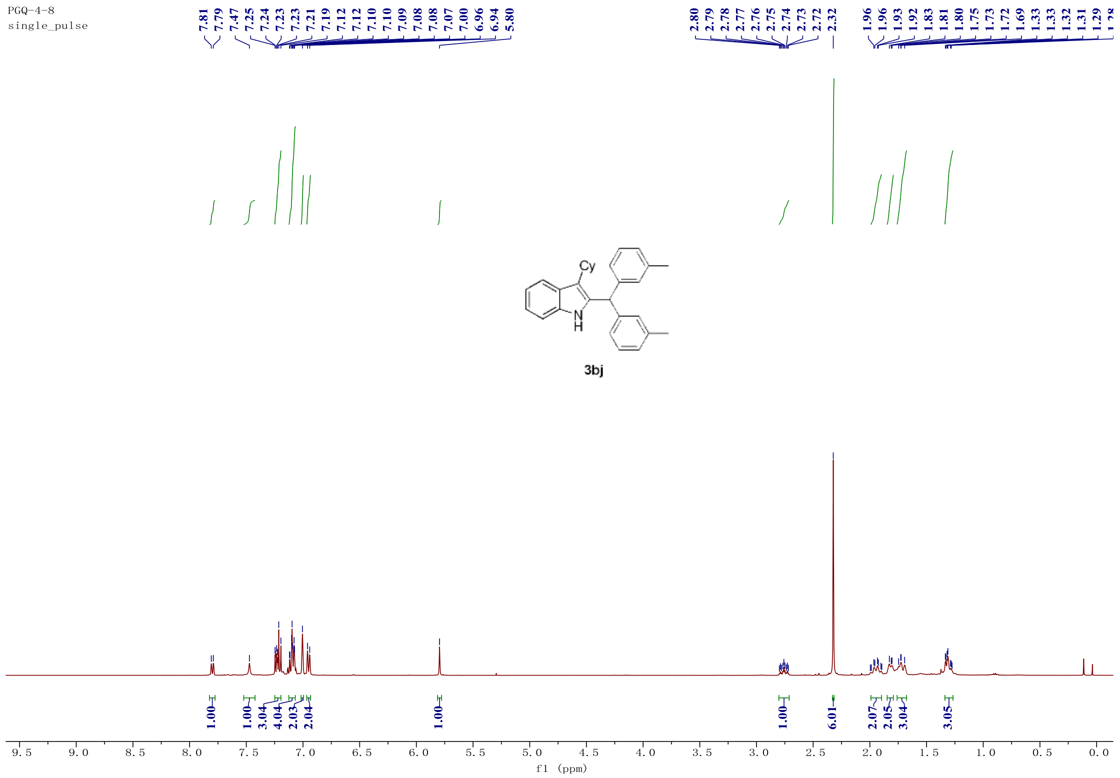
PGQ-4-8
single_pulse
7.81 7.79 7.47 7.25 7.24 7.23 7.23 7.21 7.19 7.12 7.10 7.09 7.08 7.08 7.07 7.00 6.96 6.94 5.80
2.80 2.79 2.78 2.77 2.76 2.75 2.74 2.73 2.72 2.32
1.96 1.96 1.93 1.92 1.83 1.81 1.80 1.75 1.73 1.72 1.69 1.33 1.33 1.32 1.31 1.29
Cy
N
H
3bj
1.00 1.00 3.04 4.04 2.03 2.04 1.00 1.00 6.01 2.07 2.05 3.04 3.05
f1 (ppm)

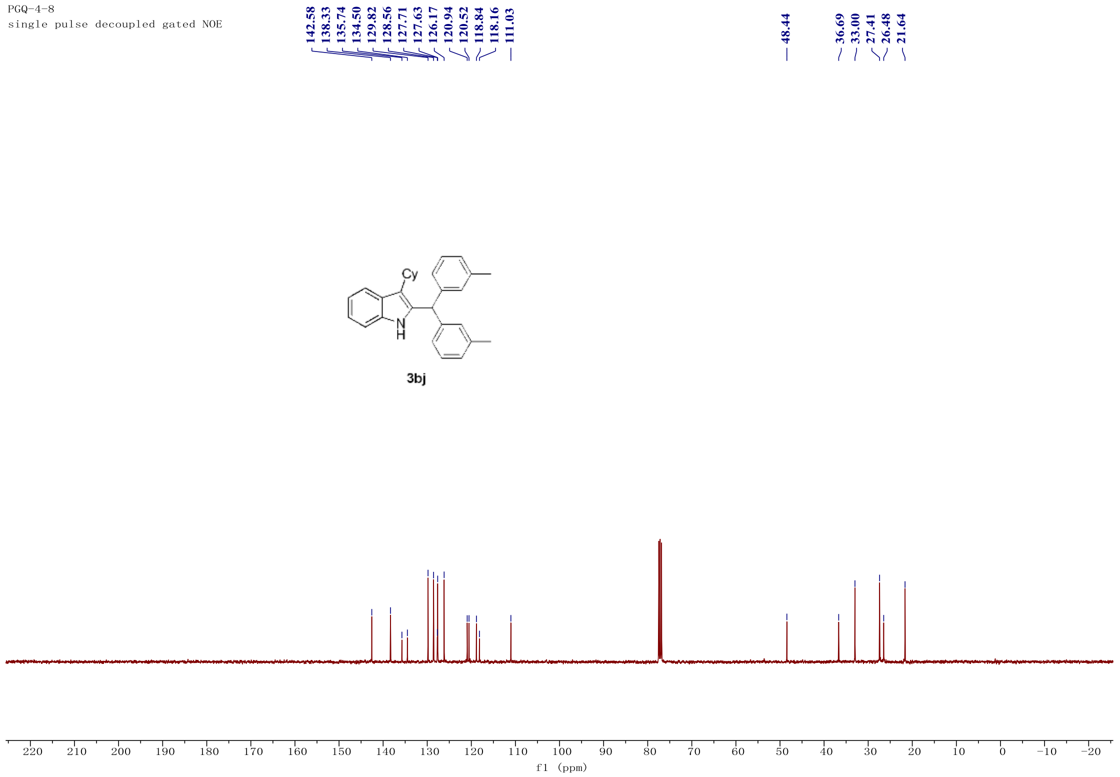
PGQ-4-8
single pulse decoupled gated NOE
142.58 138.33 135.74 134.50 129.82 128.56 127.71 127.63 126.17 120.94 120.52 118.84 118.16 111.03
48.44
36.69 33.00 27.41 26.48 21.64
Cy
N
H
3bj
f1 (ppm)

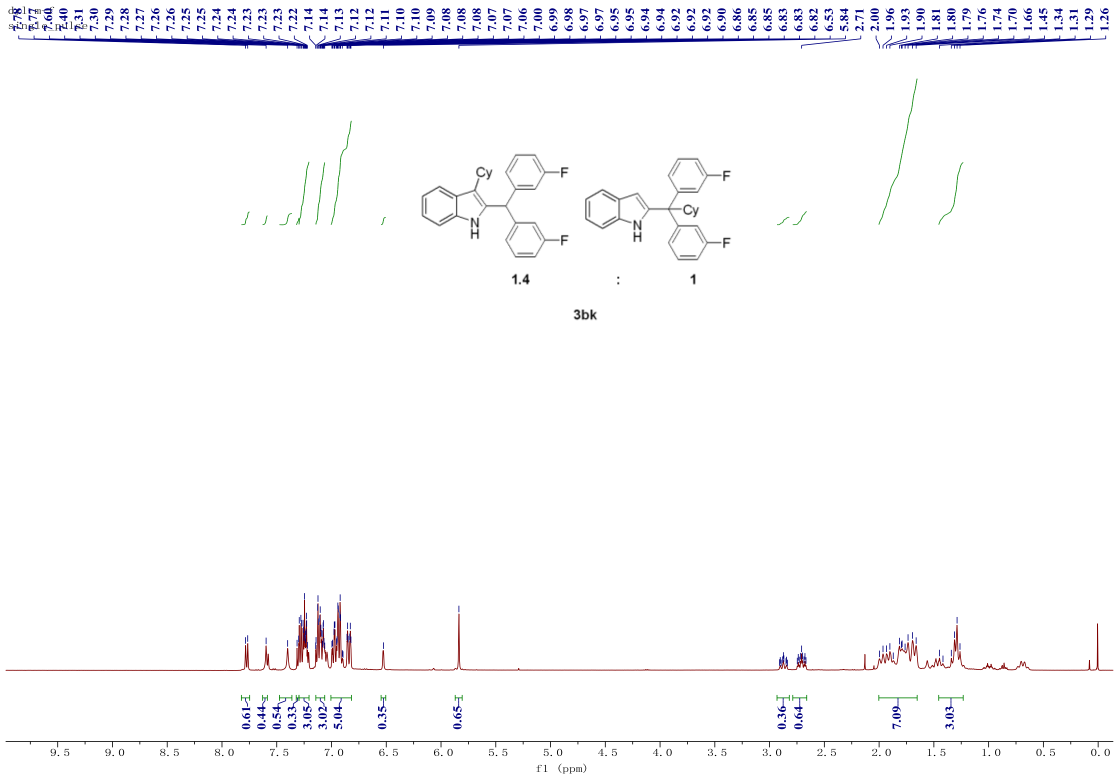
Cy
F
N
H
Cy
1.4
:
1
3bk
f1 (ppm)

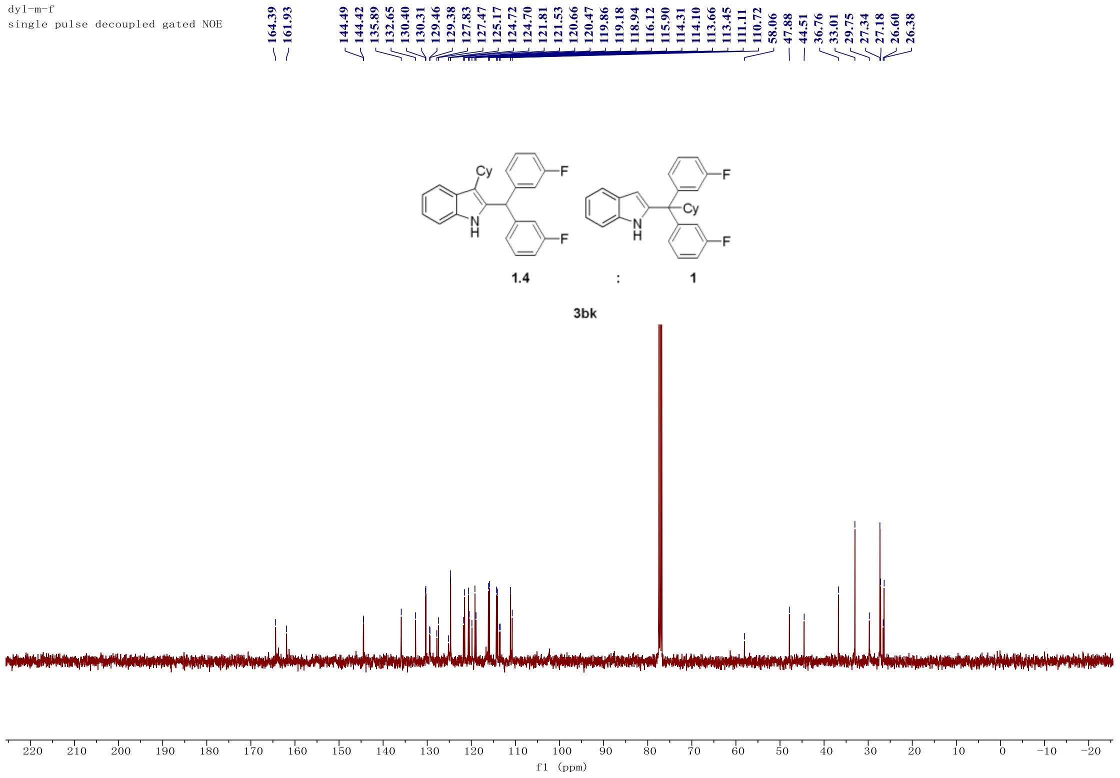
dyl-m-f
single pulse decoupled gated NOE
1.4
:
1
3bk
f1 (ppm)

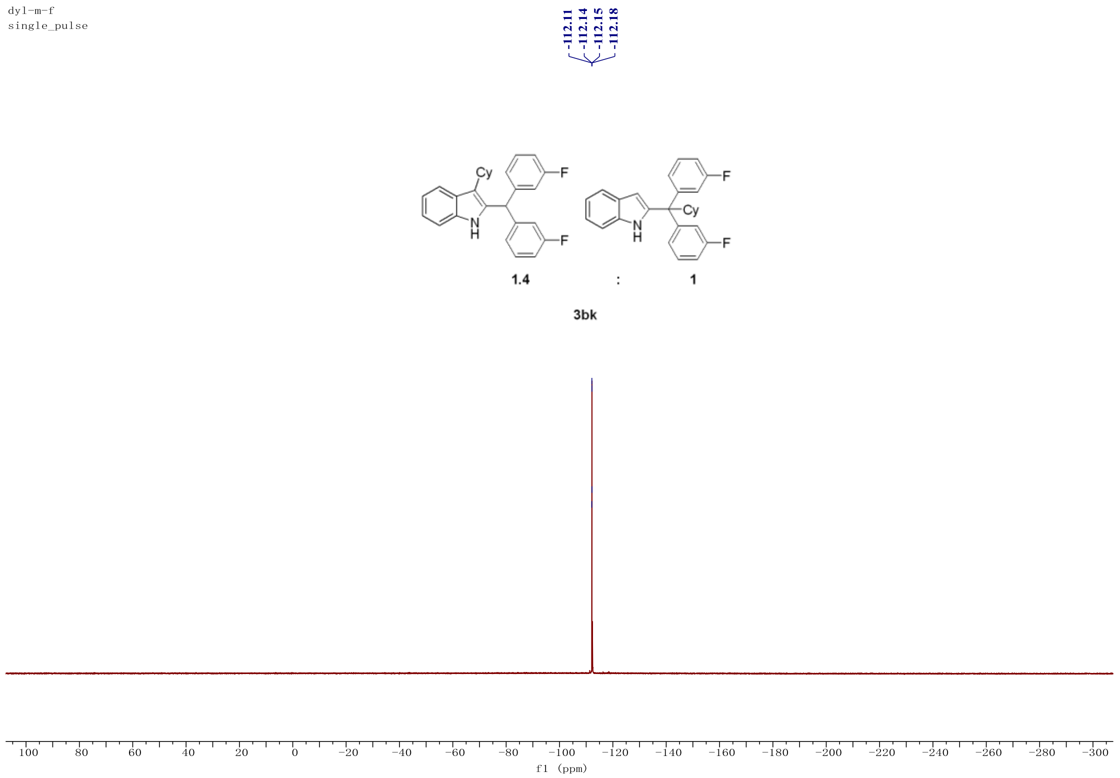
dyl-m-f
single_pulse
-112.11
-112.14
-112.15
-112.18
Cy
F
N
H
1.4
:
1
3bk
f1 (ppm)

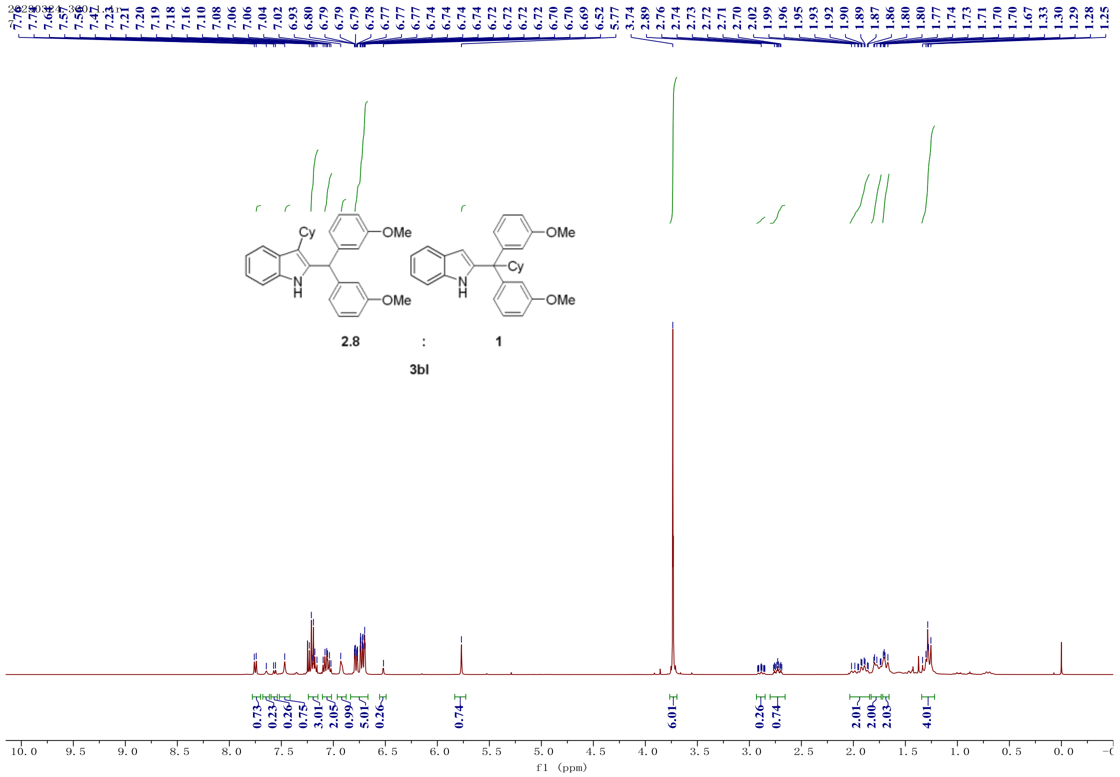
7.68
7.57
7.56
7.41
7.23
7.21
7.20
7.19
7.18
7.16
7.10
7.08
7.06
7.06
7.04
7.02
6.93
6.80
6.79
6.79
6.78
6.77
6.77
6.74
6.74
6.74
6.72
6.72
6.72
6.70
6.70
6.69
6.52
5.77
3.74
2.89
2.76
2.74
2.72
2.71
2.70
2.02
1.99
1.96
1.95
1.93
1.92
1.90
1.89
1.87
1.86
1.80
1.77
1.74
1.73
1.71
1.70
1.67
1.33
1.30
1.29
1.28
1.25
Cy
OMe
N
H
2.8
:
1
3bl
0.73
0.23
0.26
0.75
3.01
2.05
0.99
5.01
0.26
0.74
6.01
0.26
0.74
2.01
2.00
2.03
4.01
f1 (ppm)

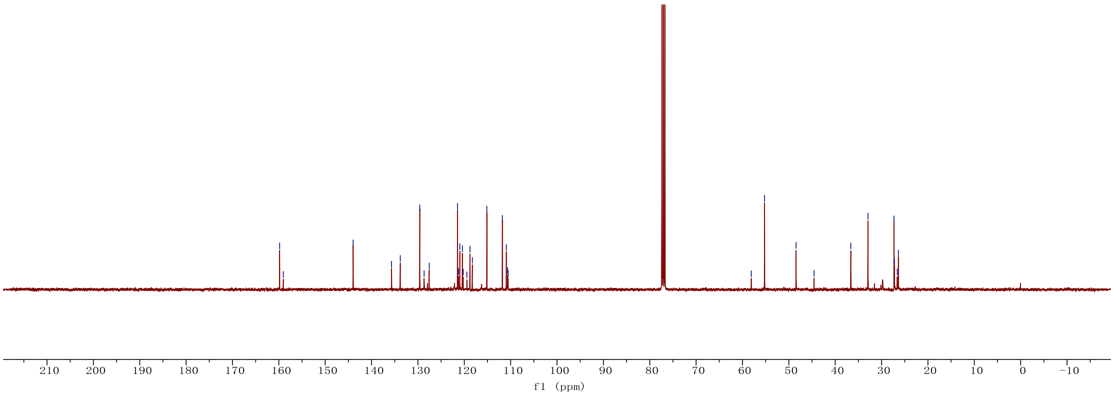
20230324.361.1.1r
7
159.83
159.03
143.97
135.69
133.80
129.60
128.66
127.56
121.46
121.26
120.96
120.42
120.23
119.45
118.77
118.26
115.14
111.79
110.94
110.67
110.54
58.10
55.23
48.44
44.56
36.63
32.93
27.32
27.24
26.62
26.38
210 200 190 180 170 160 150 140 130 120 110 100 90 80 70 60 50 40 30 20 10 0 -10
f1 (ppm)

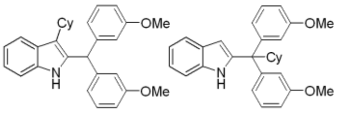
Cy
OMe
OMe
N
H
Cy
OMe
OMe
N
H

2.8 : **1**

3bl

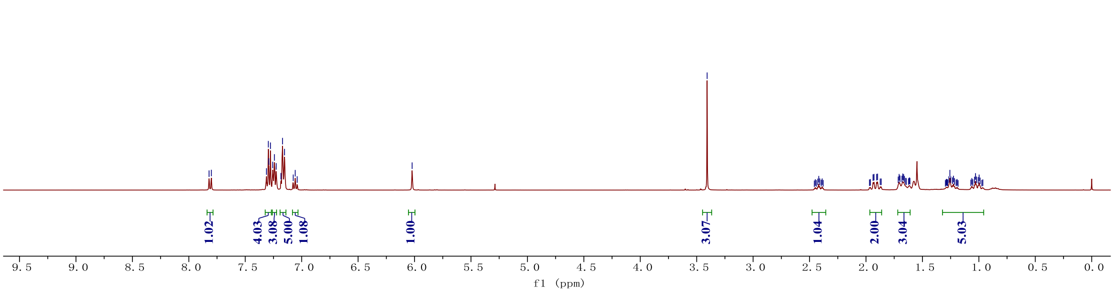
7.80
7.31
7.30
7.29
7.28
7.26
7.24
7.23
7.18
7.17
7.15
7.07
7.06
7.04
6.02
3.41
2.46
2.45
2.44
2.43
2.42
2.41
2.40
2.39
2.38
1.97
1.96
1.94
1.93
1.91
1.90
1.87
1.71
1.71
1.70
1.68
1.67
1.67
1.66
1.65
1.64
1.62
1.61
1.30
1.29
1.28
1.28
1.28
1.27
1.26
1.25
1.23
1.22
1.22
1.20
1.19
1.18
1.07
1.06
1.05
1.04
1.03
1.02
1.01
1.00
0.99
0.97
0.96
1.02
4.03
3.08
5.00
1.08
1.00
3.07
1.04
2.00
3.04
5.03
9.5 9.0 8.5 8.0 7.5 7.0 6.5 6.0 5.5 5.0 4.5 4.0 3.5 3.0 2.5 2.0 1.5 1.0 0.5 0.0
f1 (ppm)

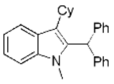
Cy
Ph
Ph
N

3bm

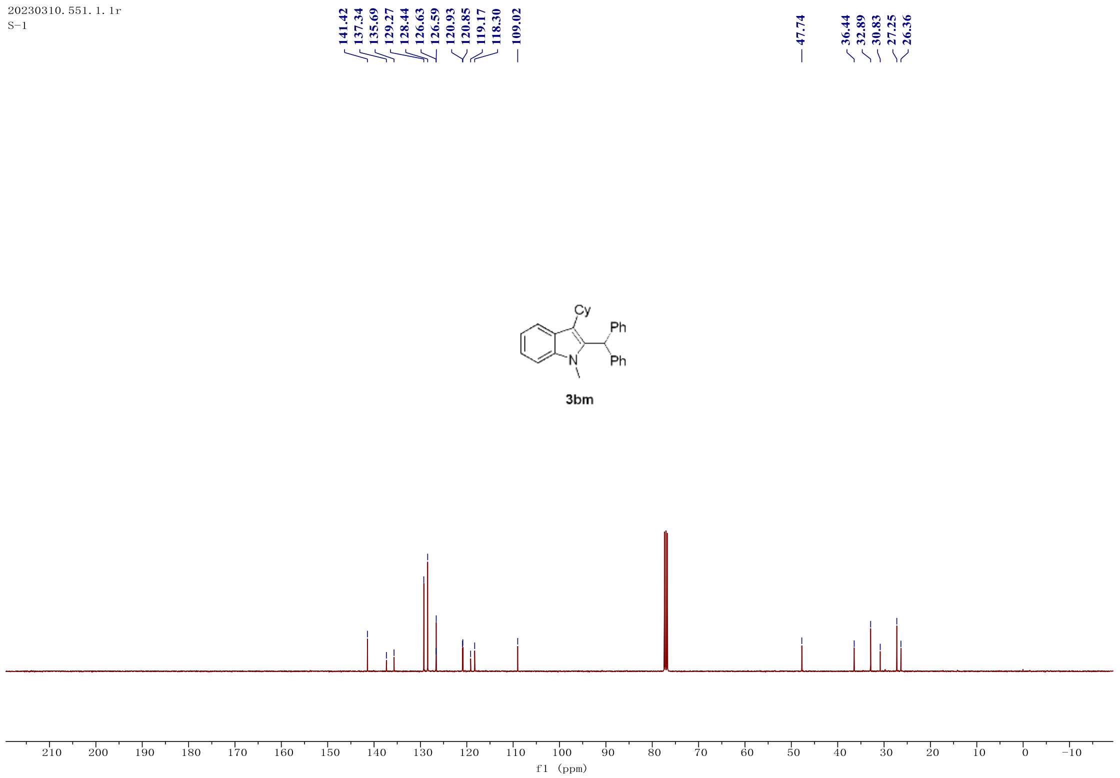
20230310.551.1.1r
S-1
141.42
137.34
135.69
129.27
128.44
126.63
126.59
120.93
120.85
119.17
118.30
109.02
47.74
36.44
32.89
30.83
27.25
26.36
Cy
Ph
Ph
N
f1 (ppm)

3bm

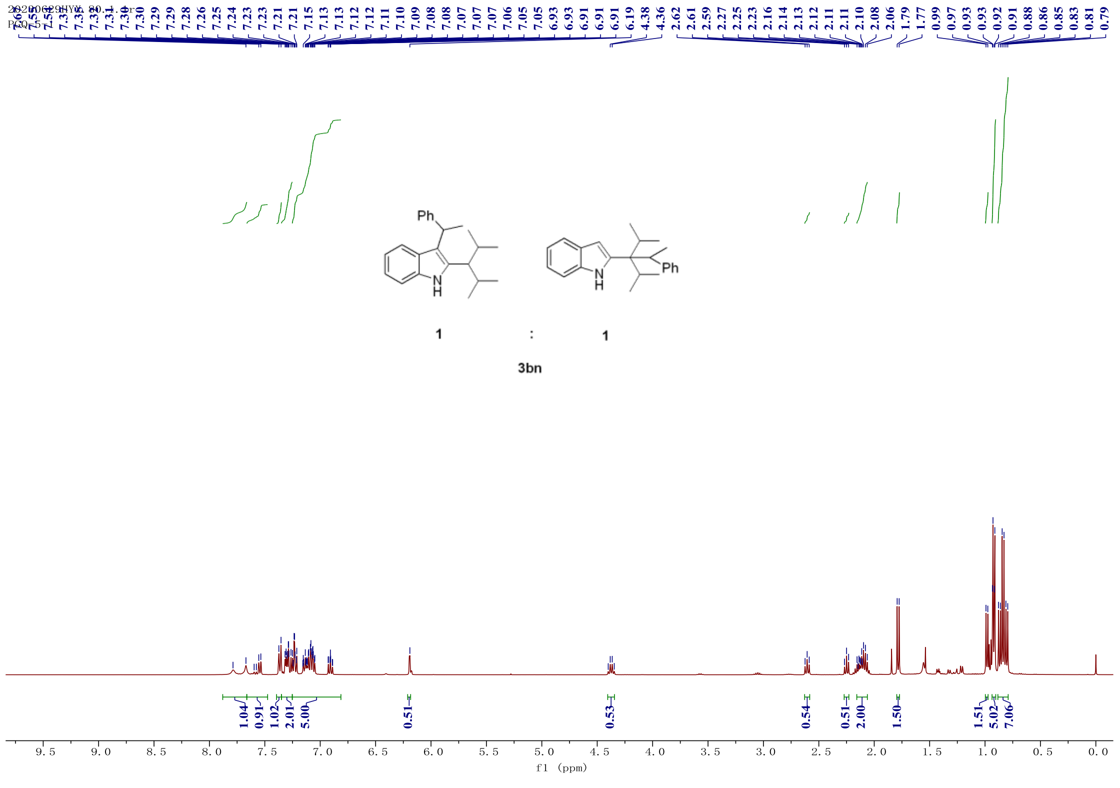
Ph
Ph
H
N
H
f1 (ppm)

1 : 1

3bn

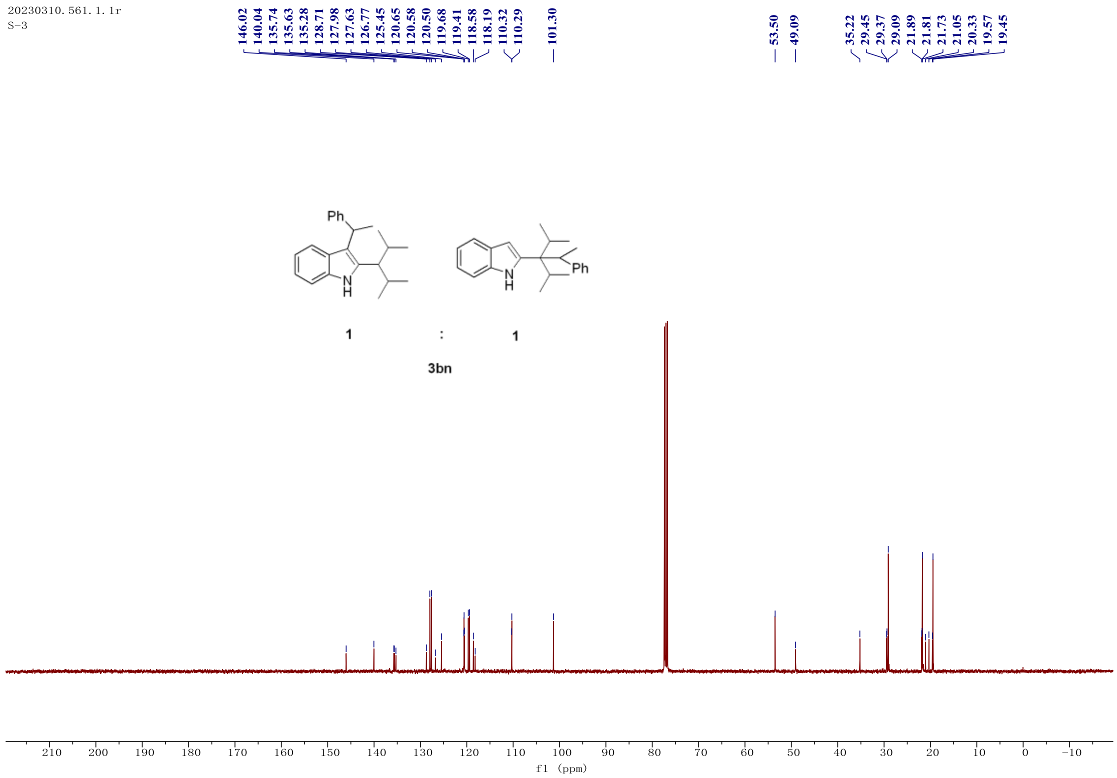
20230310.561.1.1r
S-3
146.02 140.04 135.74 135.63 135.28 128.71 127.98 127.63 126.77 125.45 120.65 120.58 120.50 119.68 119.41 118.58 118.19 110.32 110.29 101.30
53.50 49.09
35.22 29.45 29.37 29.09 21.89 21.81 21.73 21.05 20.33 19.57 19.45
Ph
H
N
Ph
N
H
1 : 1
3bn
f1 (ppm)

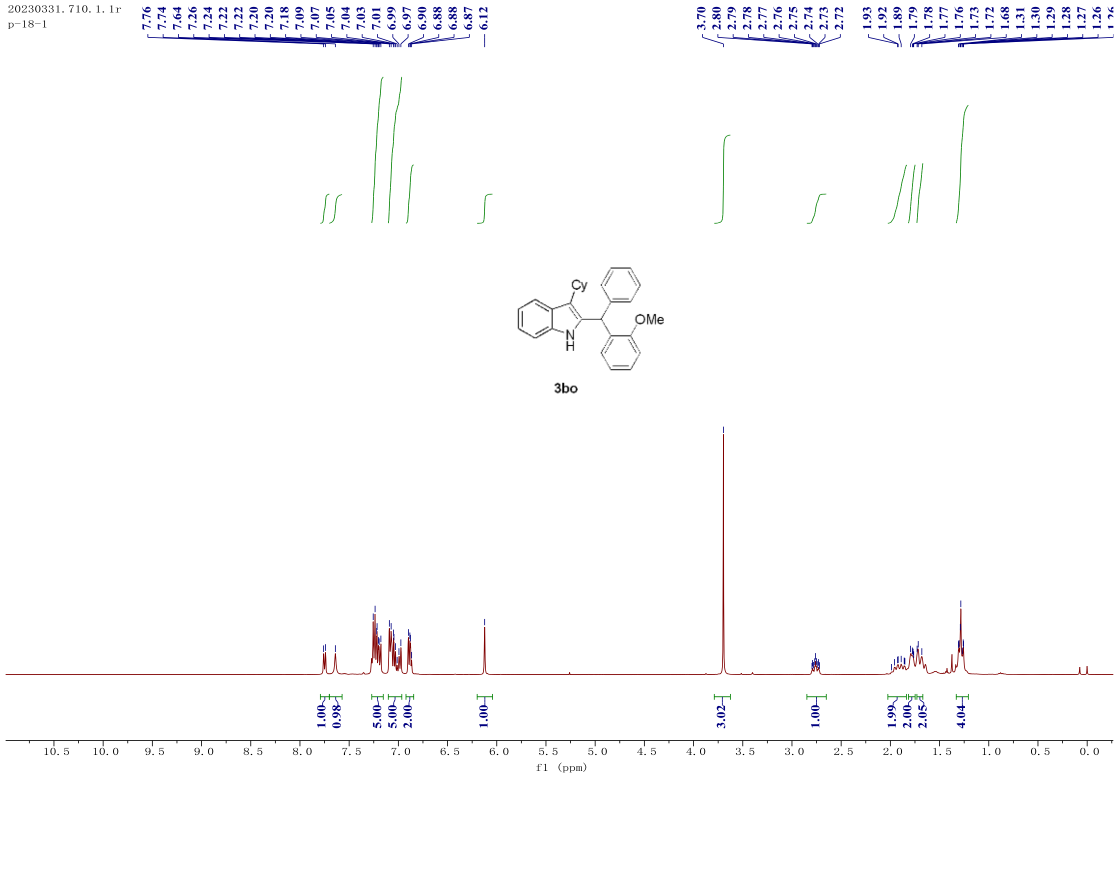
20230331.710.1.1r
p-18-1
7.76 7.74 7.64 7.26 7.24 7.22 7.20 7.18 7.09 7.07 7.05 7.04 7.03 7.01 6.99 6.97 6.90 6.88 6.87 6.12
3.70 2.80 2.79 2.78 2.77 2.76 2.75 2.74 2.73 2.72
1.93 1.92 1.89 1.79 1.78 1.77 1.76 1.73 1.72 1.68 1.31 1.30 1.29 1.28 1.27 1.26
Cy
OMe
N
H
3bo
1.00 0.98 5.00 5.00 2.00 1.00 3.02 1.00 1.99 2.00 2.05 4.04
f1 (ppm)

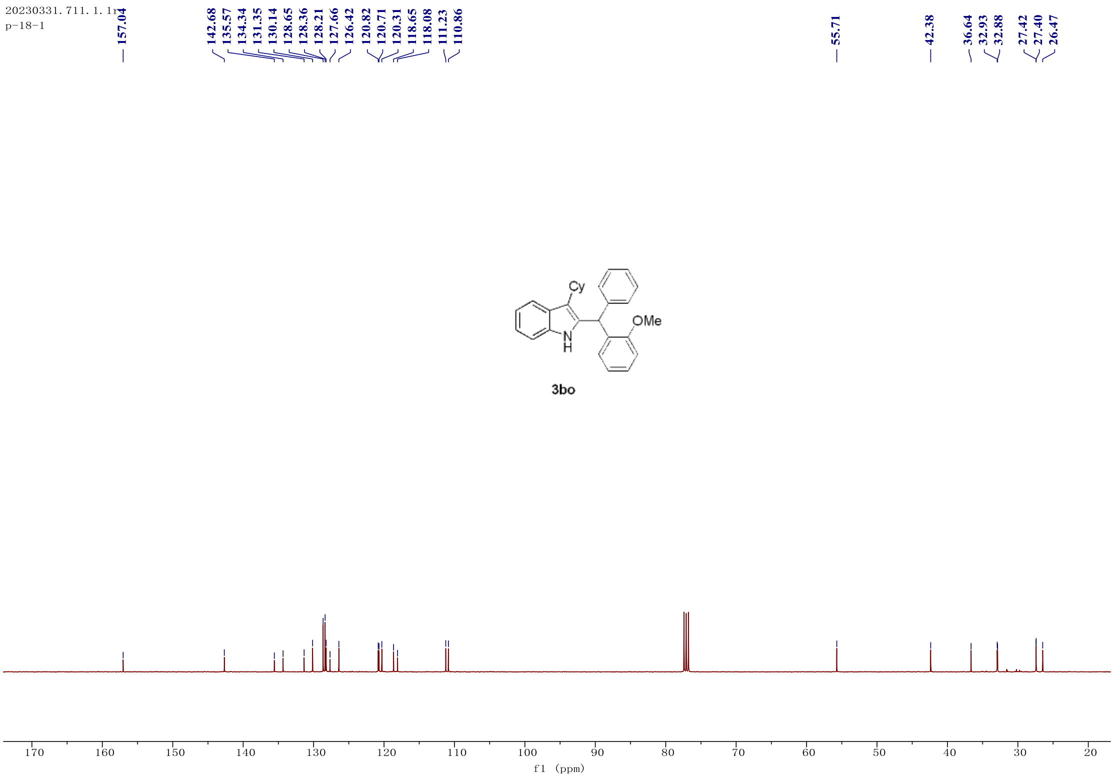
20230331.711.1.1r
p-18-1
157.04
142.68
135.57
134.34
131.35
130.14
128.65
128.36
128.21
127.66
126.42
120.82
120.71
120.31
118.65
118.08
111.23
110.86
55.71
42.38
36.64
32.93
32.88
27.42
27.40
26.47
Cy
OMe
N
H
3bo
f1 (ppm)

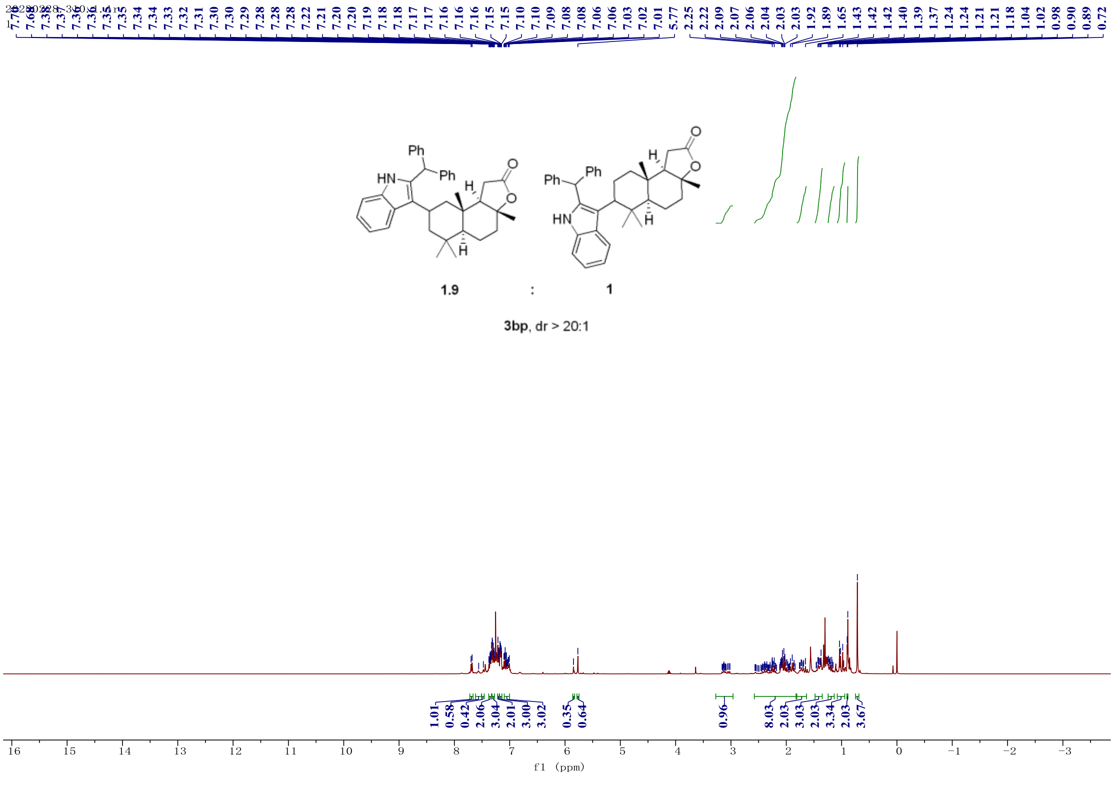
Ph
Ph
HN
H
O
1.9
:
1
3bp, dr > 20:1
1.01
0.58
0.42
2.06
3.04
2.01
3.00
3.02
0.35
0.64
0.96
8.03
2.03
3.03
2.03
3.34
2.03
3.67
f1 (ppm)

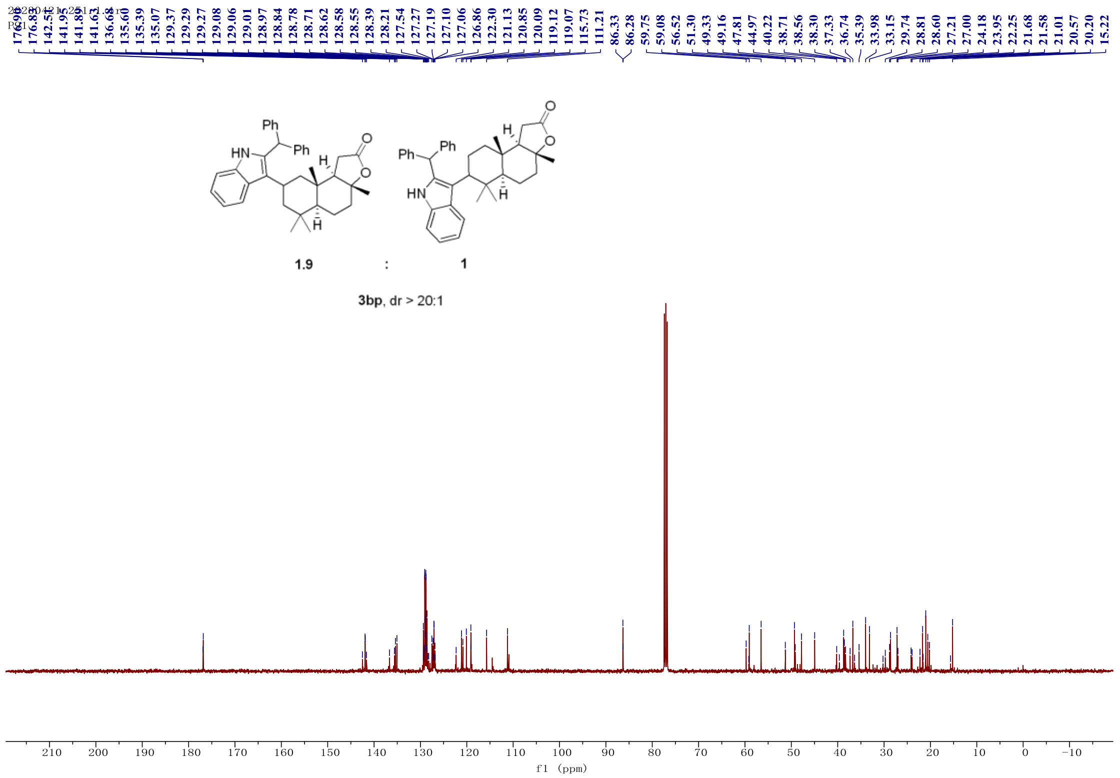
Ph
Ph
HN
H
O
O
H
Ph
Ph
HN
H
O
O
H
1.9
:
1
3bp, dr > 20:1
f1 (ppm)

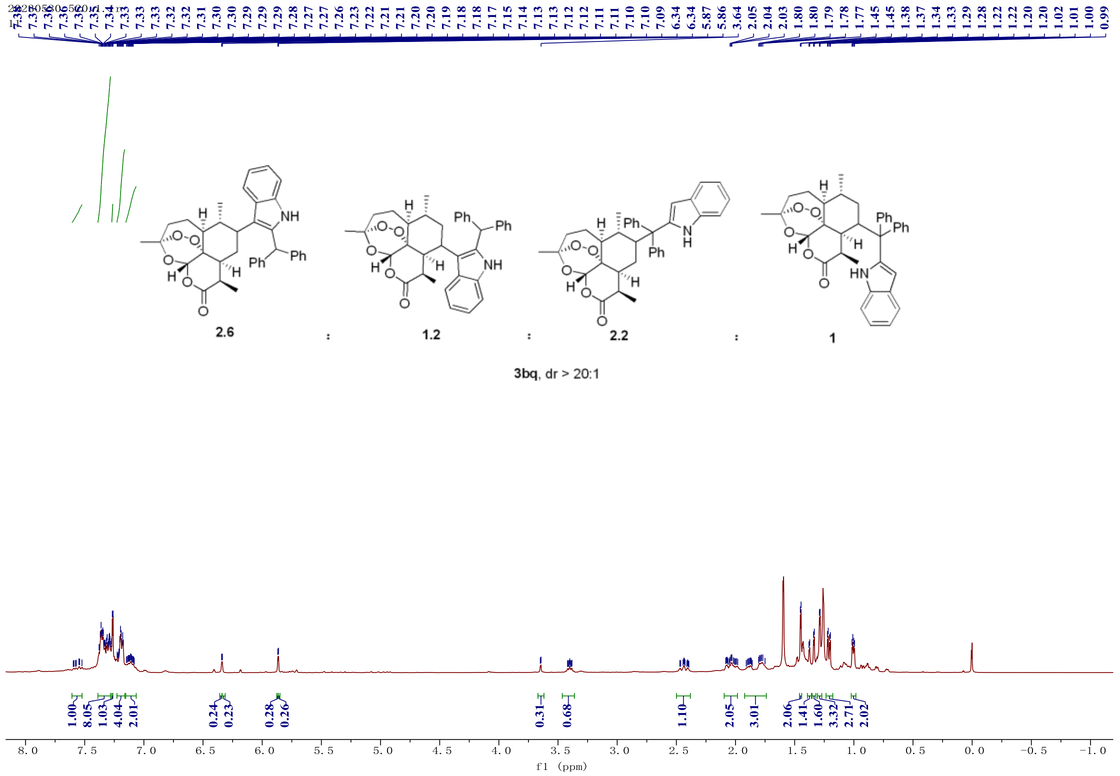
H
NH
Ph
Ph
H
O
O
H
O
O
O
2.6
:
1.2
:
2.2
:
1
3bq, dr > 20:1
f1 (ppm)

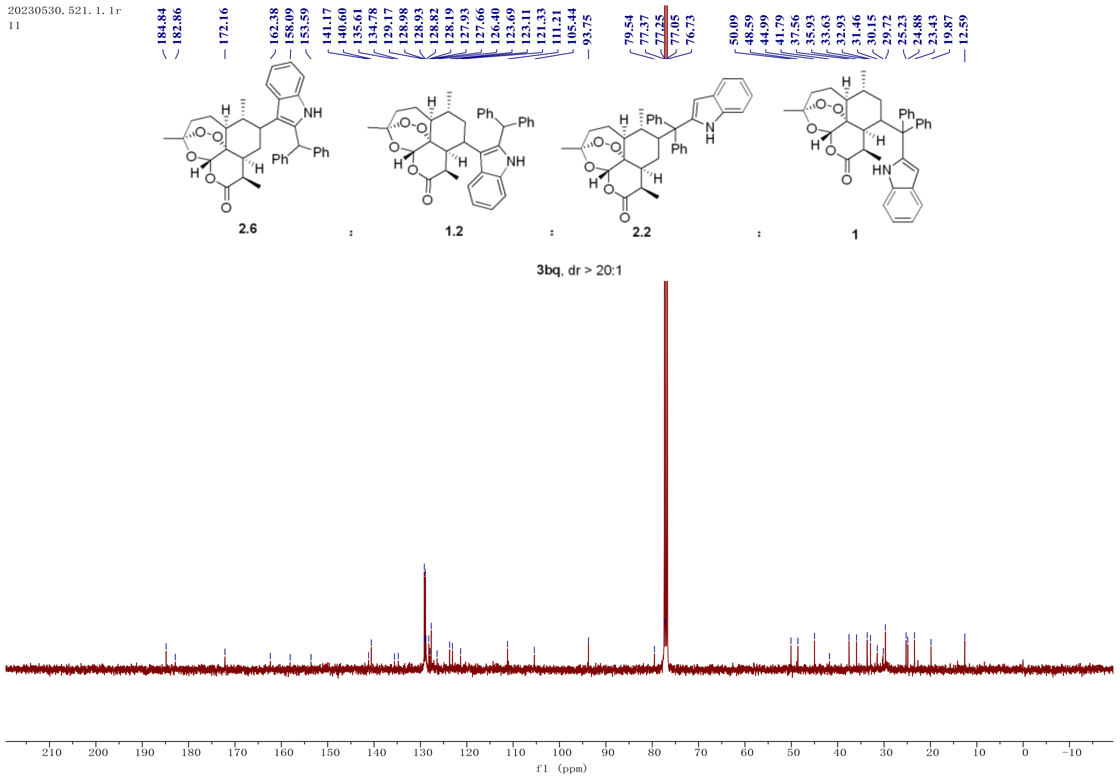

20230530.521.1.1r
11
184.84
182.86
172.16
162.38
158.09
153.59
141.17
140.60
135.61
134.78
129.17
128.98
128.93
128.82
128.19
127.93
127.66
126.40
123.69
123.11
121.33
111.21
105.44
93.75
79.54
77.37
77.25
77.05
76.73
50.09
48.59
44.99
41.79
37.56
35.93
33.63
32.93
31.46
30.15
29.72
25.23
24.88
23.43
19.87
12.59
2.6 : 1.2 : 2.2 : 1
3bq, dr > 20:1
f1 (ppm)

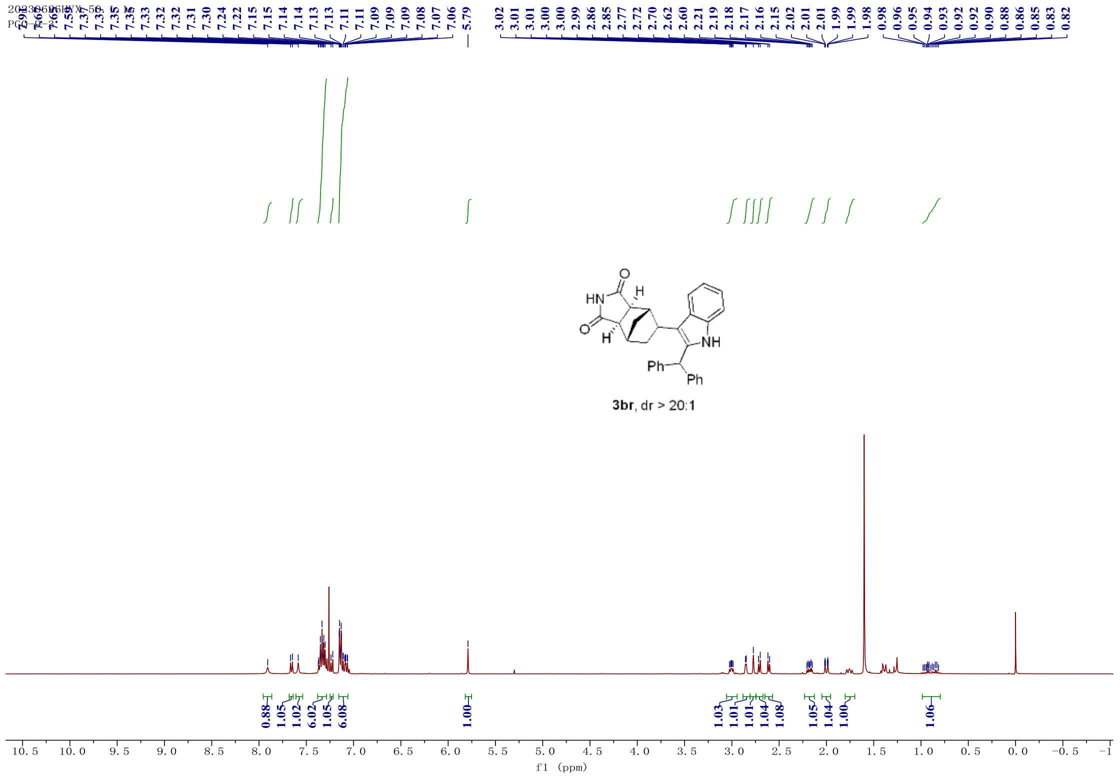

7.37
7.35
7.35
7.33
7.32
7.32
7.31
7.30
7.24
7.22
7.15
7.15
7.14
7.14
7.13
7.13
7.11
7.11
7.09
7.09
7.08
7.07
7.06
5.79
3.02
3.01
3.00
3.00
2.99
2.86
2.77
2.72
2.70
2.62
2.60
2.21
2.19
2.18
2.17
2.16
2.15
2.02
2.01
2.01
1.99
1.98
0.98
0.96
0.95
0.94
0.93
0.92
0.92
0.90
0.88
0.86
0.85
0.83
0.82
3br, dr > 20:1
0.88
1.05
1.02
6.02
1.05
6.08
1.00
1.03
1.01
1.01
1.04
1.08
1.05
1.04
1.00
1.06
f1 (ppm)

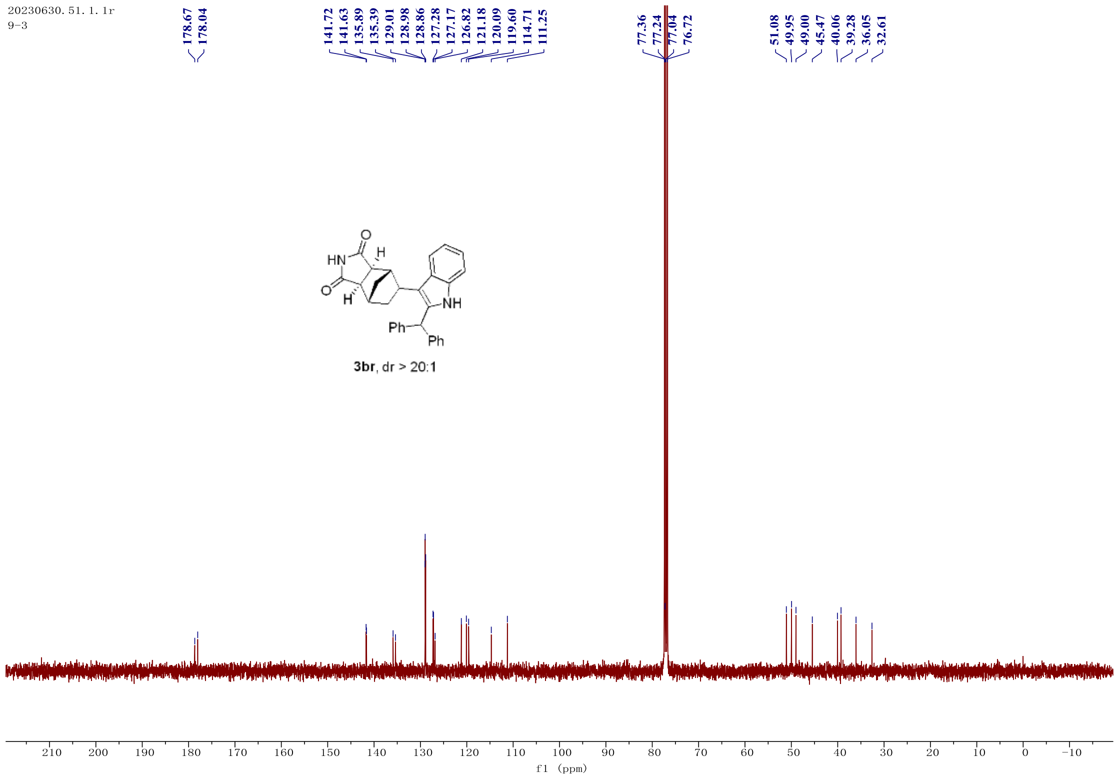
20230630.51.1.1r
9-3
178.67
178.04
141.72
141.63
135.89
135.39
129.01
128.98
128.86
127.28
127.17
126.82
121.18
120.09
119.60
114.71
111.25
77.36
77.24
77.04
76.72
51.08
49.95
49.00
45.47
40.06
39.28
36.05
32.61
HN
O
H
NH
Ph
3br, dr > 20:1
f1 (ppm)

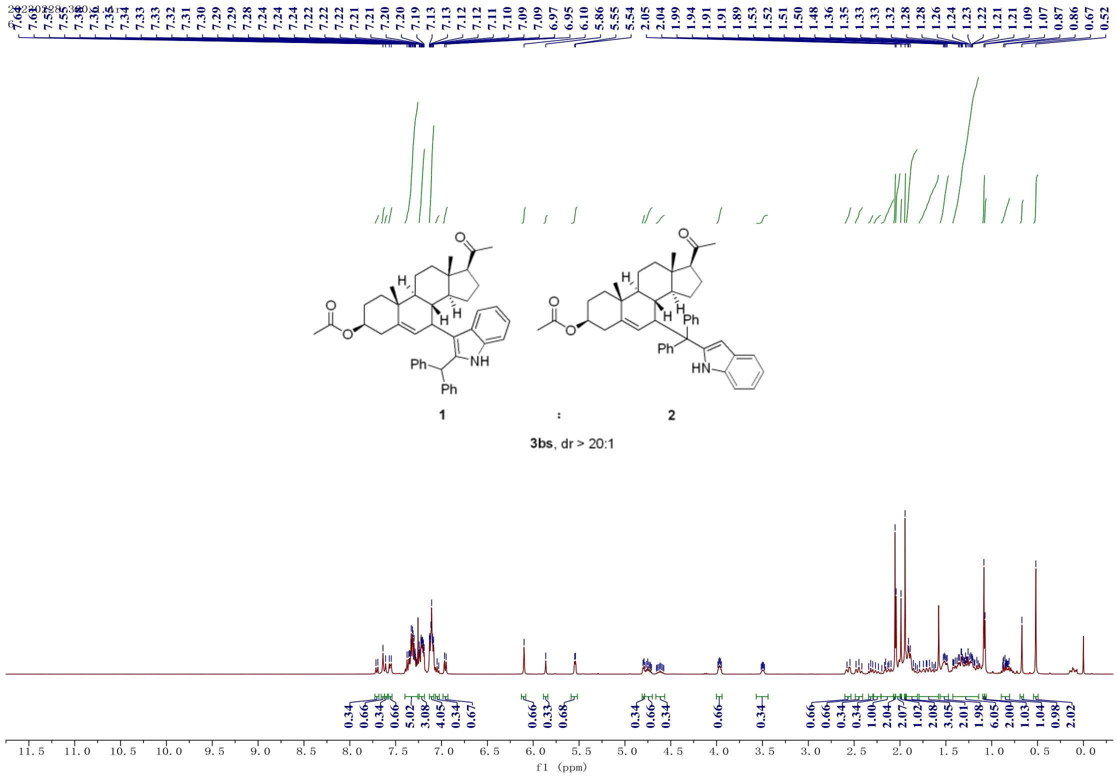
7.63
7.55
7.36
7.35
7.34
7.33
7.33
7.32
7.31
7.30
7.29
7.29
7.28
7.24
7.24
7.24
7.22
7.22
7.21
7.21
7.20
7.20
7.19
7.13
7.13
7.12
7.12
7.11
7.10
7.09
7.09
6.97
6.95
6.10
5.86
5.55
5.54
2.05
2.04
1.99
1.94
1.91
1.89
1.53
1.52
1.51
1.50
1.48
1.36
1.35
1.33
1.33
1.32
1.28
1.28
1.26
1.24
1.23
1.22
1.21
1.21
1.09
1.07
0.87
0.86
0.67
0.52
Ph
NH
HN
1
:
2
3bs, dr > 20:1
0.34
0.66
0.34
0.66
5.02
3.08
4.05
0.34
0.67
0.66
0.33
0.68
0.34
0.66
0.34
0.66
0.34
0.66
0.66
0.34
1.00
2.04
2.07
1.02
2.08
3.05
2.01
1.98
6.05
2.00
1.03
1.04
0.98
2.02
f1 (ppm)

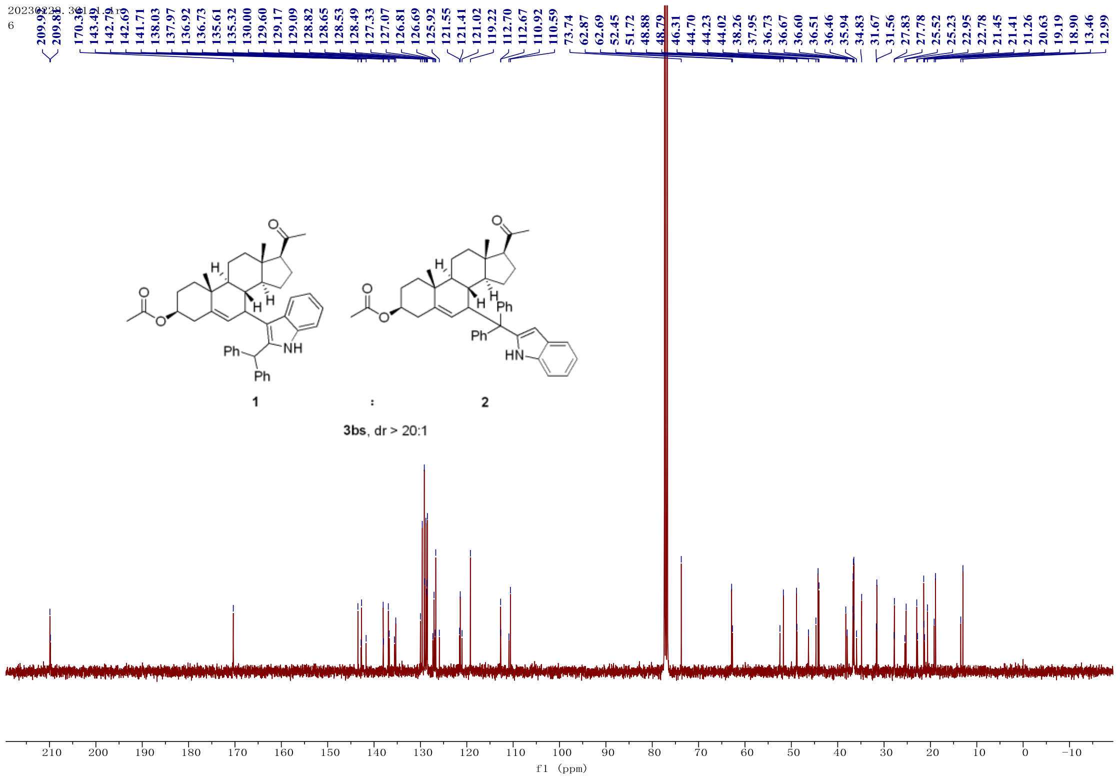
209.92
209.83
170.36
143.42
142.79
142.69
141.71
138.03
137.97
136.92
136.73
135.61
135.32
130.00
129.60
129.17
129.09
128.82
128.65
128.53
128.49
127.33
127.07
126.81
126.69
125.92
121.55
121.41
121.02
119.22
112.70
112.67
110.92
110.59
73.74
62.87
62.69
52.45
51.72
48.88
48.79
46.31
44.70
44.23
44.02
38.26
37.95
36.73
36.67
36.60
36.51
36.46
35.94
34.83
31.67
31.56
27.83
27.78
25.52
25.23
22.95
22.78
21.45
21.41
21.26
20.63
19.19
18.90
13.46
12.99
H
H
H
NH
Ph
Ph
1
:
H
H
H
Ph
Ph
HN
2
3bs, dr > 20:1
f1 (ppm)

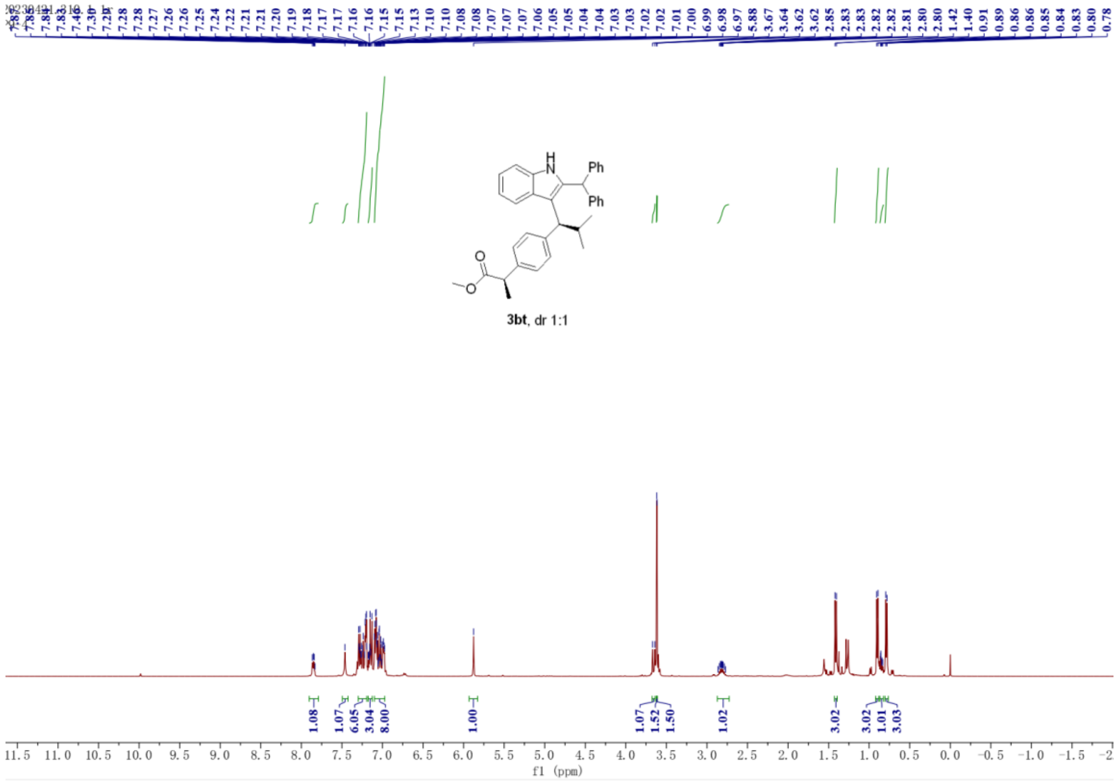
H
N
Ph
Ph
O
O
3bt, dr 1:1
1.08
1.07
6.05
3.04
8.00
1.00
1.07
1.52
1.50
1.02
3.02
3.02
1.01
3.03
f1 (ppm)

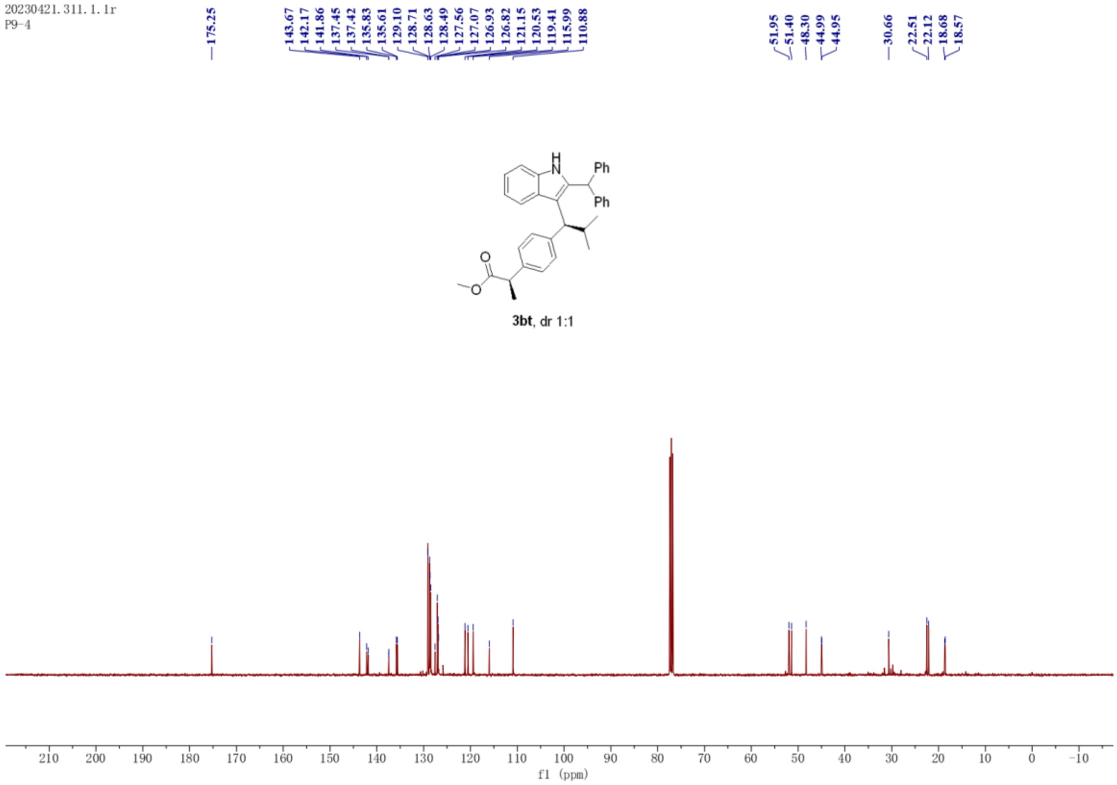
20230421.311.1.1r
P9-4
175.25
143.67
142.17
141.86
137.45
137.42
135.83
135.61
129.10
128.71
128.63
128.49
127.56
127.07
126.93
126.82
121.15
120.53
119.41
115.99
110.88
51.95
51.40
48.30
44.99
44.95
30.66
22.51
22.12
18.68
18.57
H
N
Ph
Ph
O
O
210 200 190 180 170 160 150 140 130 120 110 100 90 80 70 60 50 40 30 20 10 0 -10
f1 (ppm)

3bt, dr 1:1

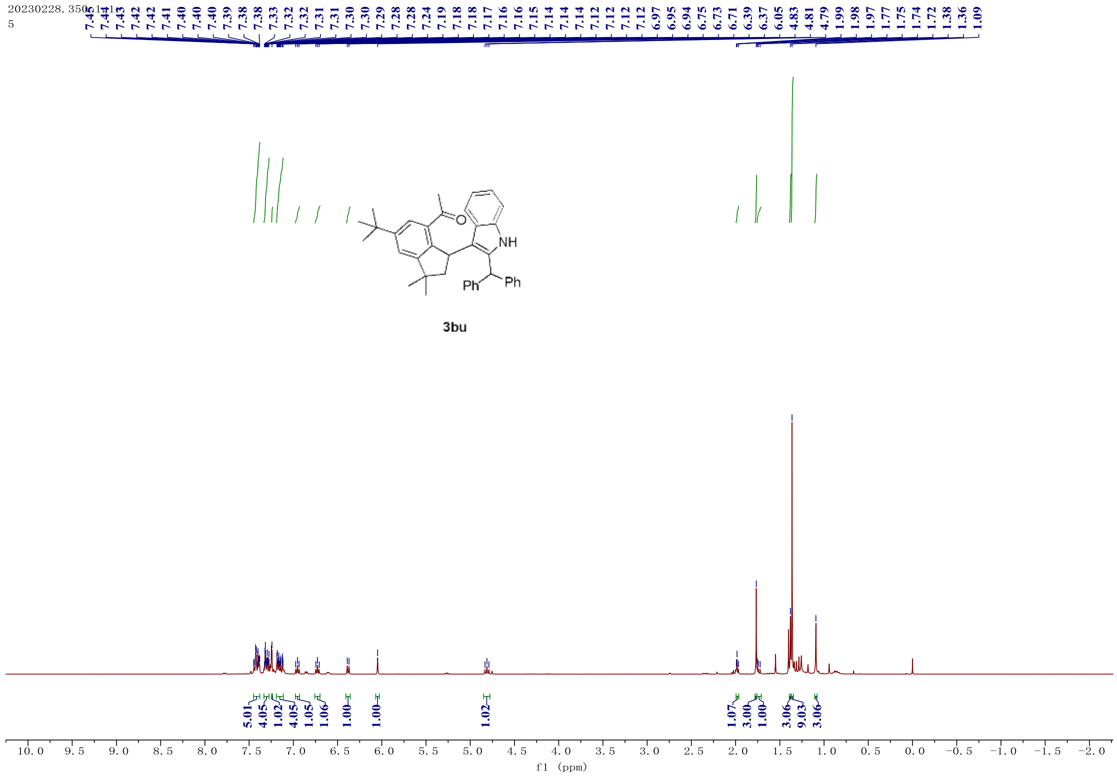
5
7.45
7.44
7.43
7.42
7.41
7.40
7.40
7.39
7.38
7.38
7.33
7.32
7.32
7.31
7.31
7.30
7.29
7.28
7.24
7.19
7.18
7.17
7.16
7.16
7.15
7.14
7.14
7.12
7.12
7.12
6.97
6.95
6.94
6.75
6.73
6.71
6.39
6.37
6.05
4.83
4.81
4.79
1.99
1.98
1.97
1.77
1.75
1.74
1.72
1.38
1.36
1.09
O
NH
Ph
Ph
5.01
4.05
1.02
4.05
1.05
1.06
1.00
1.00
1.02
1.07
3.00
1.00
3.06
9.03
3.06
10.0 9.5 9.0 8.5 8.0 7.5 7.0 6.5 6.0 5.5 5.0 4.5 4.0 3.5 3.0 2.5 2.0 1.5 1.0 0.5 0.0 -0.5 -1.0 -1.5 -2.0
f1 (ppm)

3bu

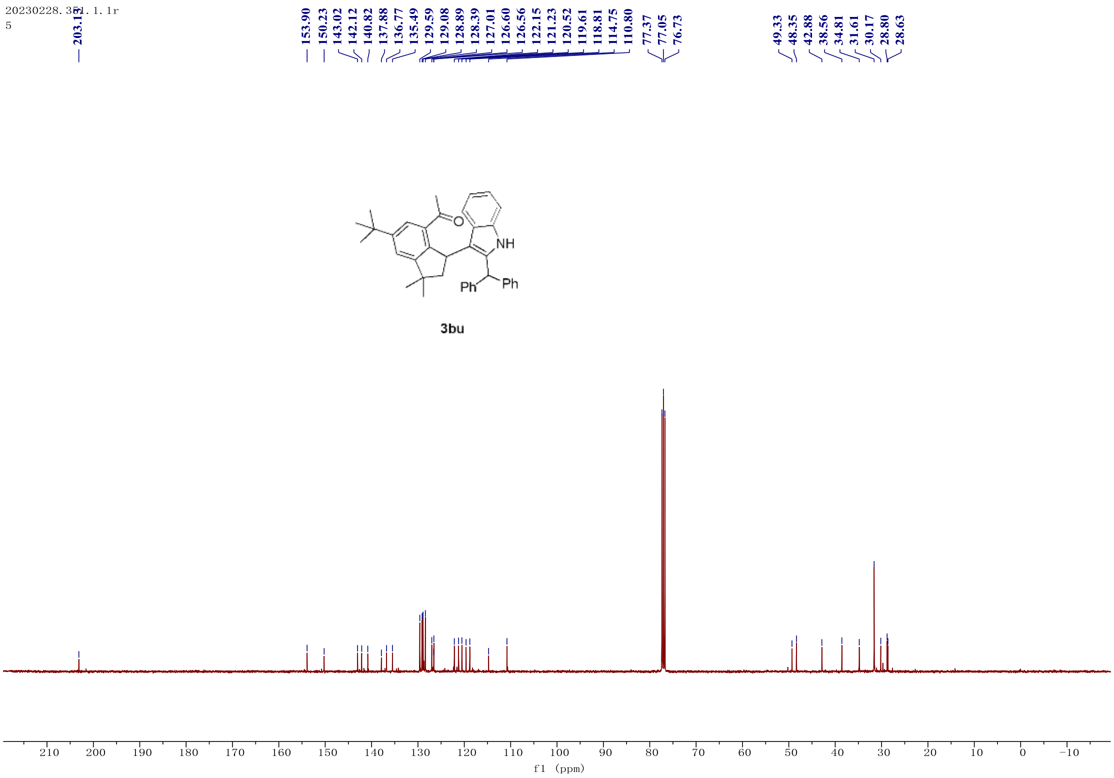
203.11
153.90
150.23
143.02
142.12
140.82
137.88
136.77
135.49
129.59
129.08
128.89
128.39
127.01
126.60
126.56
122.15
121.23
120.52
119.61
118.81
114.75
110.80
77.37
77.05
76.73
49.33
48.35
42.88
38.56
34.81
31.61
30.17
28.80
28.63
NH
Ph
Ph
3bu
210 200 190 180 170 160 150 140 130 120 110 100 90 80 70 60 50 40 30 20 10 0 -10
f1 (ppm)

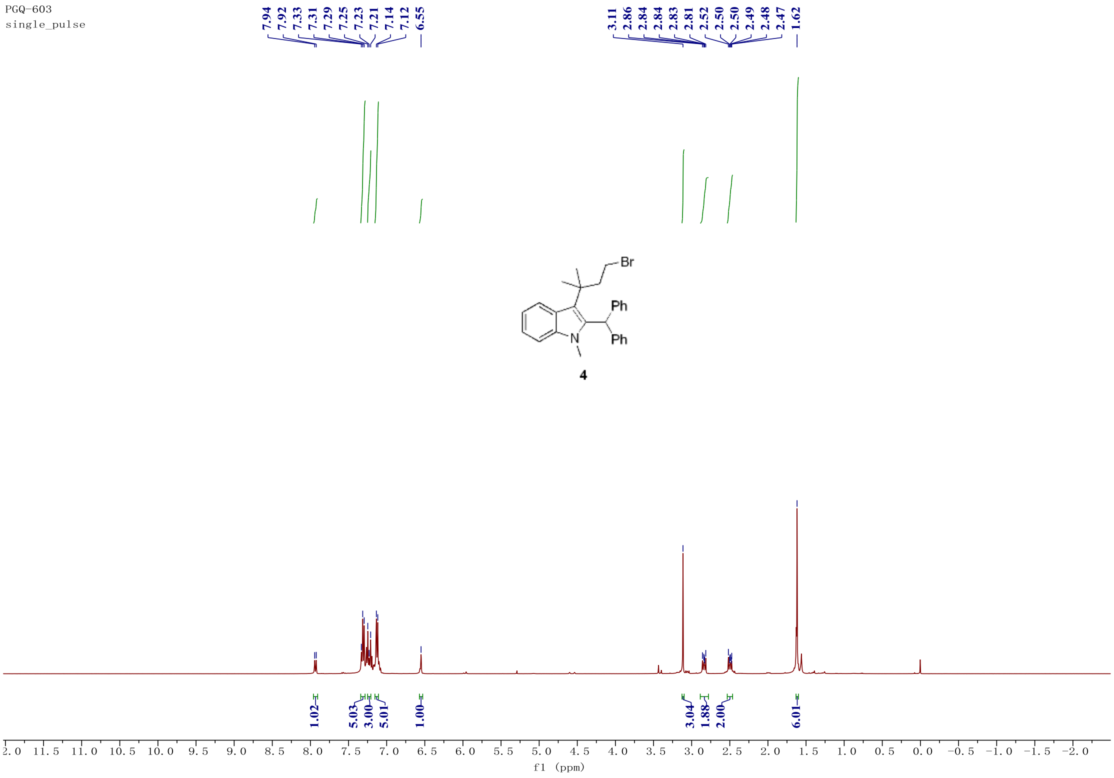
PGQ-603
single_pulse
7.94
7.92
7.33
7.31
7.29
7.25
7.23
7.21
7.14
7.12
6.55
3.11
2.86
2.84
2.84
2.83
2.81
2.52
2.50
2.50
2.49
2.48
2.47
1.62
Br
Ph
Ph
N
4
1.02
5.03
3.00
5.01
1.00
3.04
1.88
2.00
6.01
f1 (ppm)

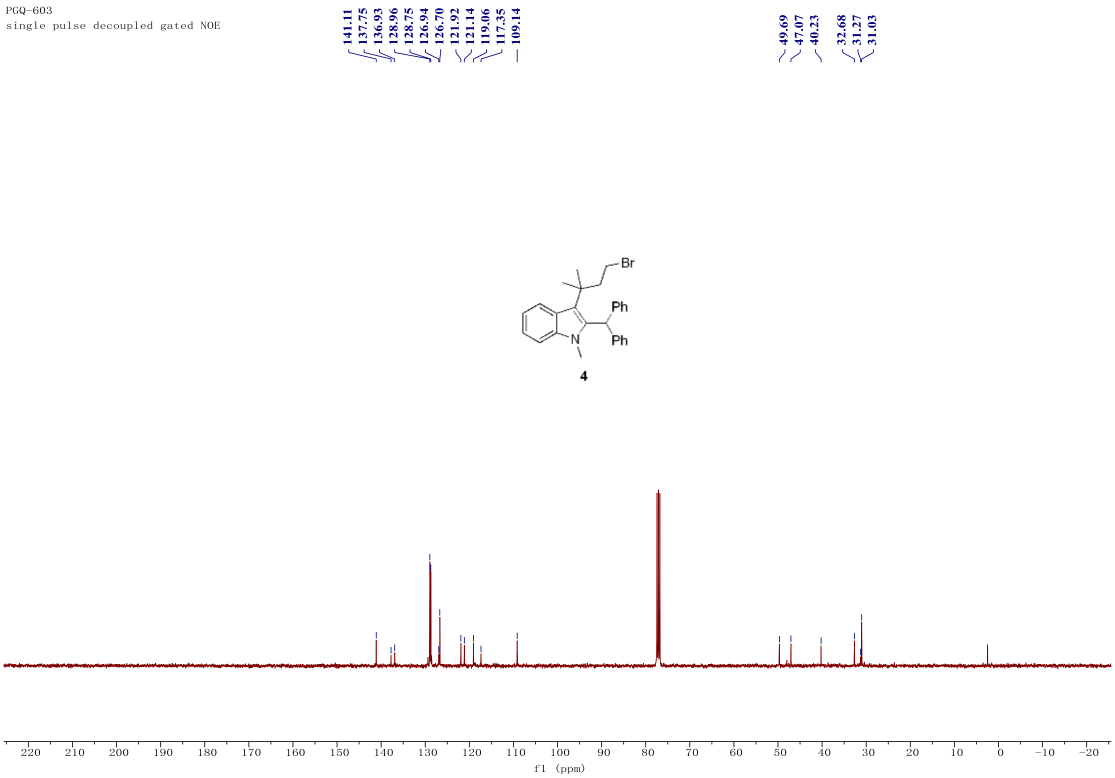
PGQ-603
single pulse decoupled gated NOE
141.11
137.75
136.93
128.96
128.75
126.94
126.70
121.92
121.14
119.06
117.35
109.14
49.69
47.07
40.23
32.68
31.27
31.03
Br
Ph
Ph
N
4
f1 (ppm)

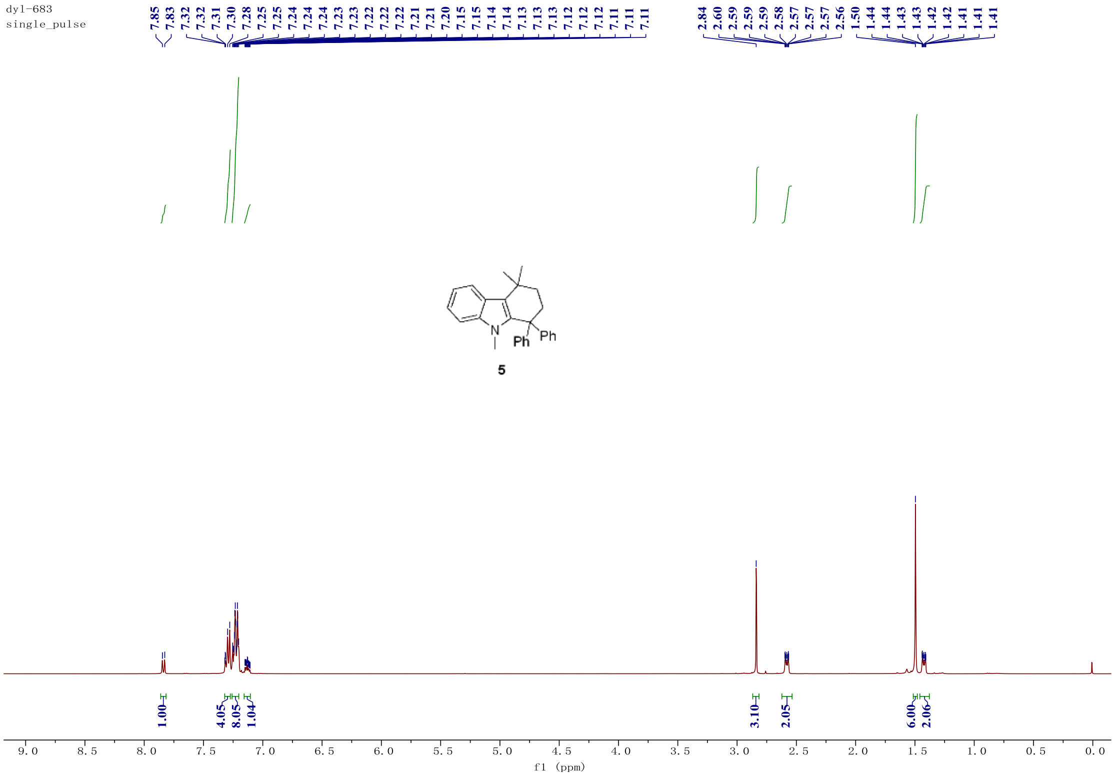
dyl-683
single_pulse
7.85
7.83
7.32
7.31
7.30
7.28
7.25
7.25
7.24
7.24
7.24
7.23
7.23
7.22
7.22
7.22
7.21
7.20
7.15
7.15
7.14
7.14
7.13
7.13
7.12
7.12
7.12
7.11
7.11
7.11
2.84
2.60
2.59
2.59
2.58
2.57
2.57
2.57
2.56
1.50
1.44
1.44
1.43
1.43
1.42
1.42
1.41
1.41
1.41
N
Ph
Ph
5
1.00
4.05
8.05
1.04
3.10
2.05
6.00
2.06
f1 (ppm)

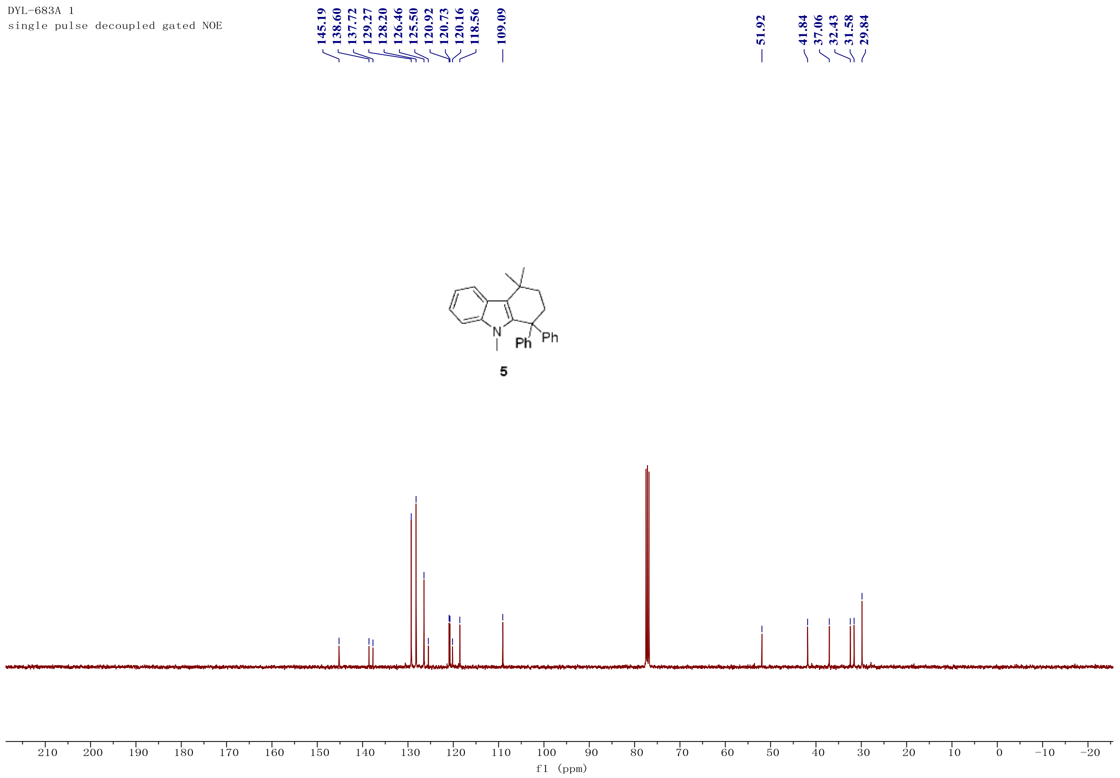
DYL-683A 1
single pulse decoupled gated NOE
145.19
138.60
137.72
129.27
128.20
126.46
125.50
120.92
120.73
120.16
118.56
109.09
51.92
41.84
37.06
32.43
31.58
29.84
Ph
Ph
5
210 200 190 180 170 160 150 140 130 120 110 100 90 80 70 60 50 40 30 20 10 0 -10 -20
f1 (ppm)

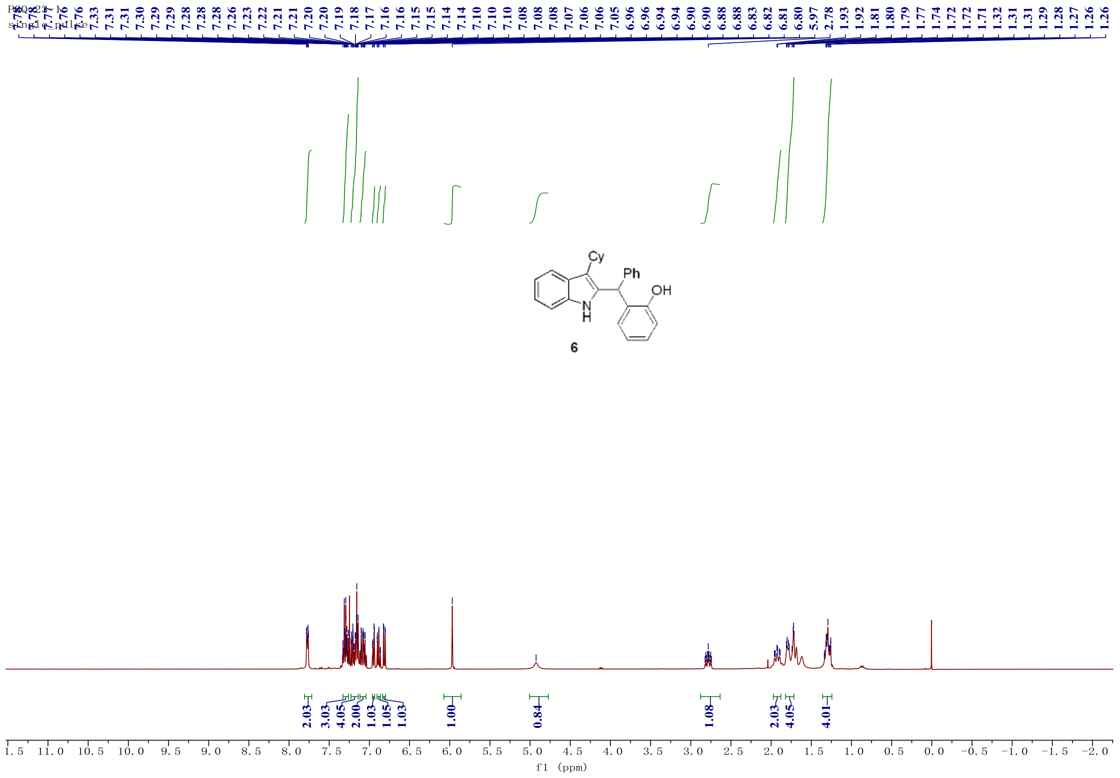
7.33
7.31
7.31
7.30
7.29
7.29
7.28
7.28
7.26
7.23
7.22
7.21
7.21
7.20
7.20
7.19
7.18
7.17
7.16
7.16
7.15
7.15
7.14
7.14
7.10
7.10
7.10
7.10
7.08
7.08
7.07
7.06
7.06
7.05
6.96
6.96
6.94
6.94
6.90
6.90
6.88
6.88
6.83
6.82
6.81
6.80
5.97
2.78
1.93
1.92
1.81
1.80
1.79
1.77
1.74
1.72
1.71
1.32
1.31
1.29
1.28
1.27
1.26
1.26
Cy
Ph
OH
N
H
6
2.03
3.03
4.05
2.00
1.03
1.05
1.03
1.00
0.84
1.08
2.03
4.05
4.01
11.0 10.5 10.0 9.5 9.0 8.5 8.0 7.5 7.0 6.5 6.0 5.5 5.0 4.5 4.0 3.5 3.0 2.5 2.0 1.5 1.0 0.5 0.0 -0.5 -1.0 -1.5 -2.0
f1 (ppm)

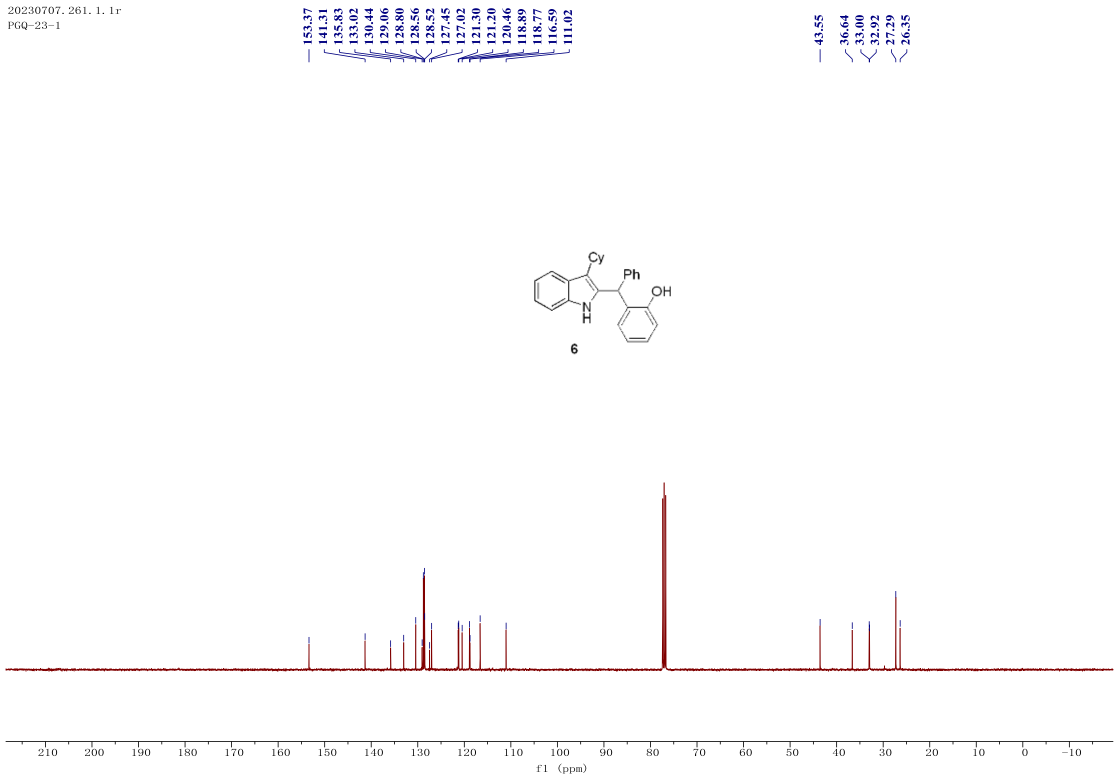
20230707.261.1.1r
PGQ-23-1
153.37
141.31
135.83
133.02
130.44
129.06
128.80
128.56
128.52
127.45
127.02
121.30
121.20
120.46
118.89
118.77
116.59
111.02
43.55
36.64
33.00
32.92
27.29
26.35
Cy
Ph
OH
N
H
6
f1 (ppm)

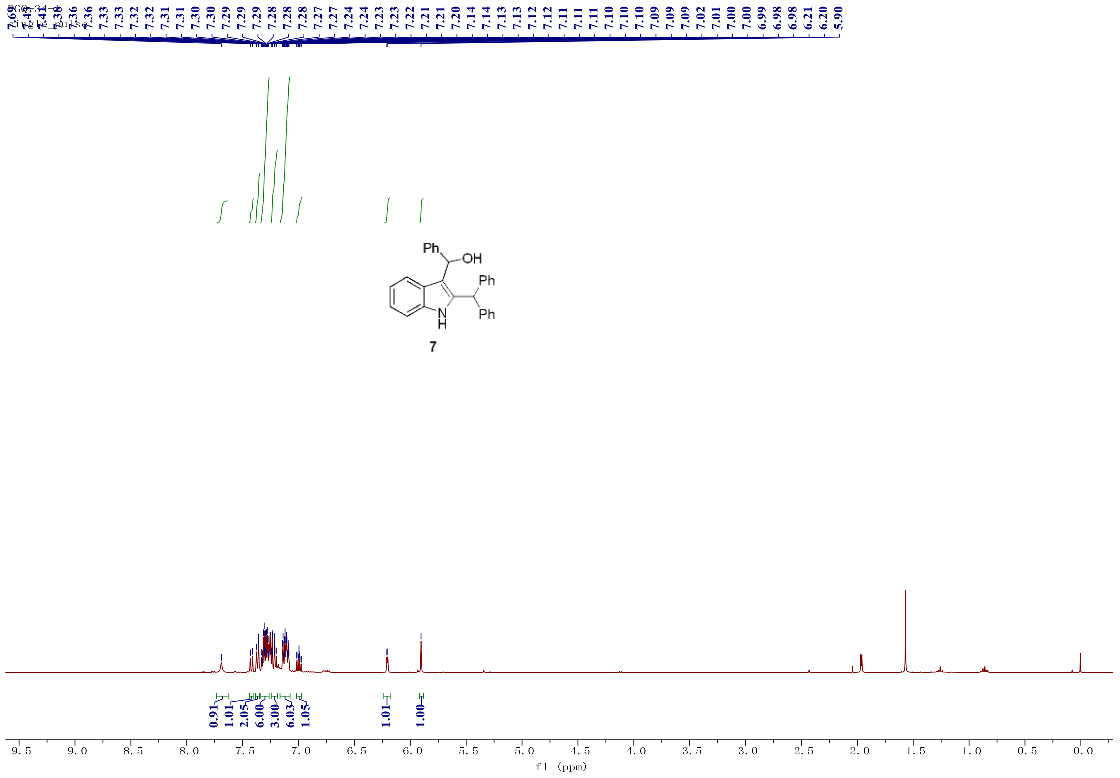
7.36
7.33
7.32
7.32
7.31
7.31
7.30
7.29
7.29
7.28
7.28
7.28
7.27
7.27
7.24
7.23
7.23
7.22
7.21
7.21
7.20
7.14
7.14
7.13
7.12
7.12
7.11
7.11
7.11
7.10
7.10
7.09
7.09
7.09
7.02
7.01
7.00
6.99
6.98
6.98
6.21
6.20
5.90
Ph
OH
Ph
Ph
N
H
7
0.91
1.01
2.05
6.00
3.00
6.03
1.05
1.01
1.00
f1 (ppm)

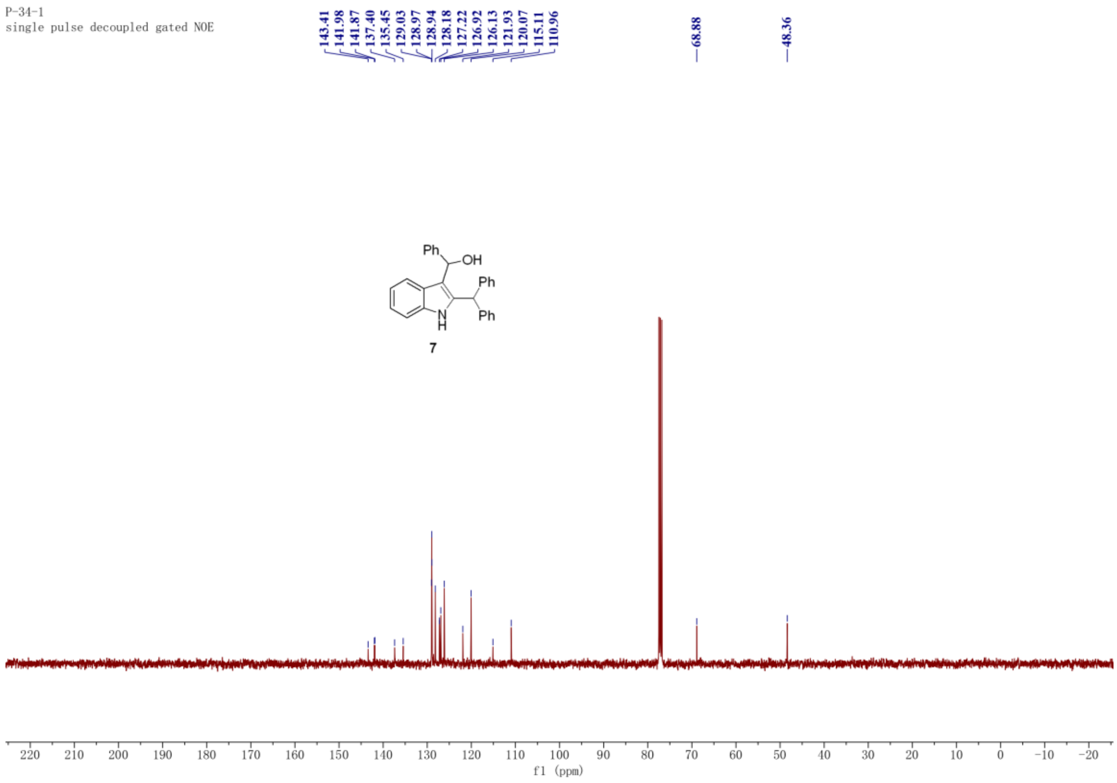
P-34-1
single pulse decoupled gated NOE
143.41
141.98
141.87
137.40
135.45
129.03
128.97
128.94
128.18
127.22
126.92
126.13
121.93
120.07
115.11
110.96
68.88
48.36
Ph
OH
Ph
Ph
N
H
7
f1 (ppm)

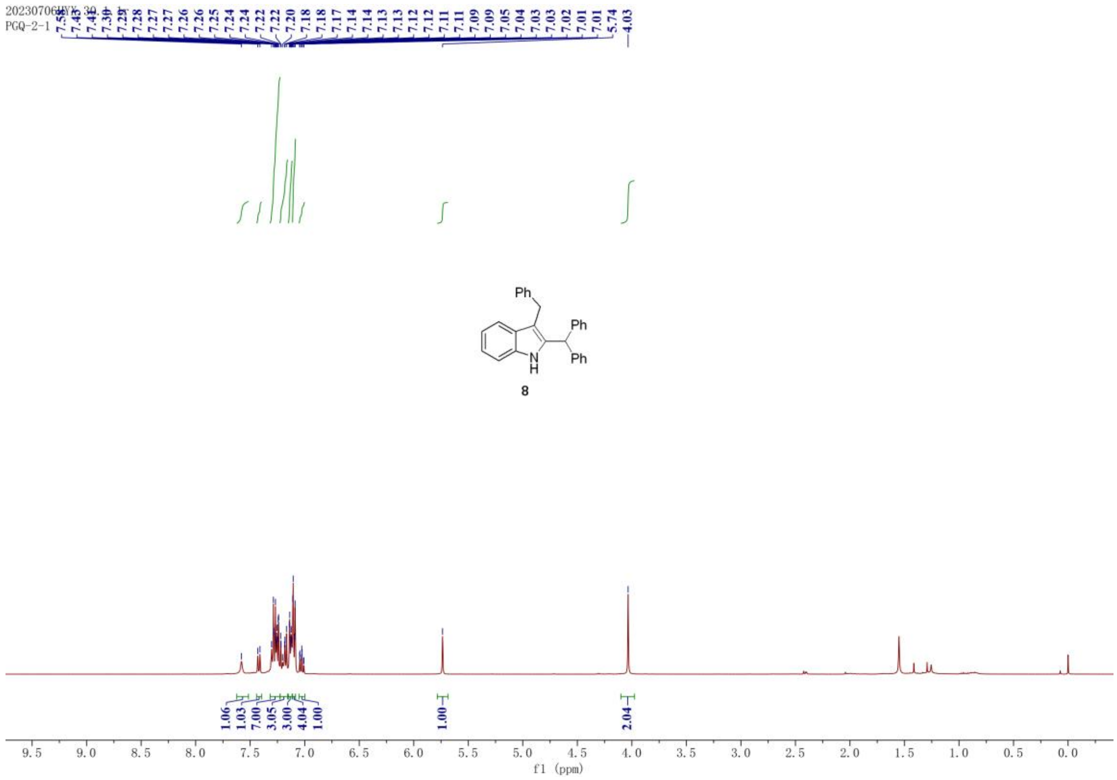
PGQ-2-1
7.58
7.30
7.28
7.27
7.26
7.25
7.24
7.24
7.22
7.22
7.20
7.18
7.17
7.14
7.14
7.13
7.13
7.12
7.12
7.11
7.11
7.09
7.09
7.05
7.04
7.03
7.03
7.02
7.01
7.01
5.74
4.03
Ph
Ph
Ph
N
H
8
1.06
1.03
7.00
3.05
3.00
4.04
1.00
1.00
2.04
f1 (ppm)

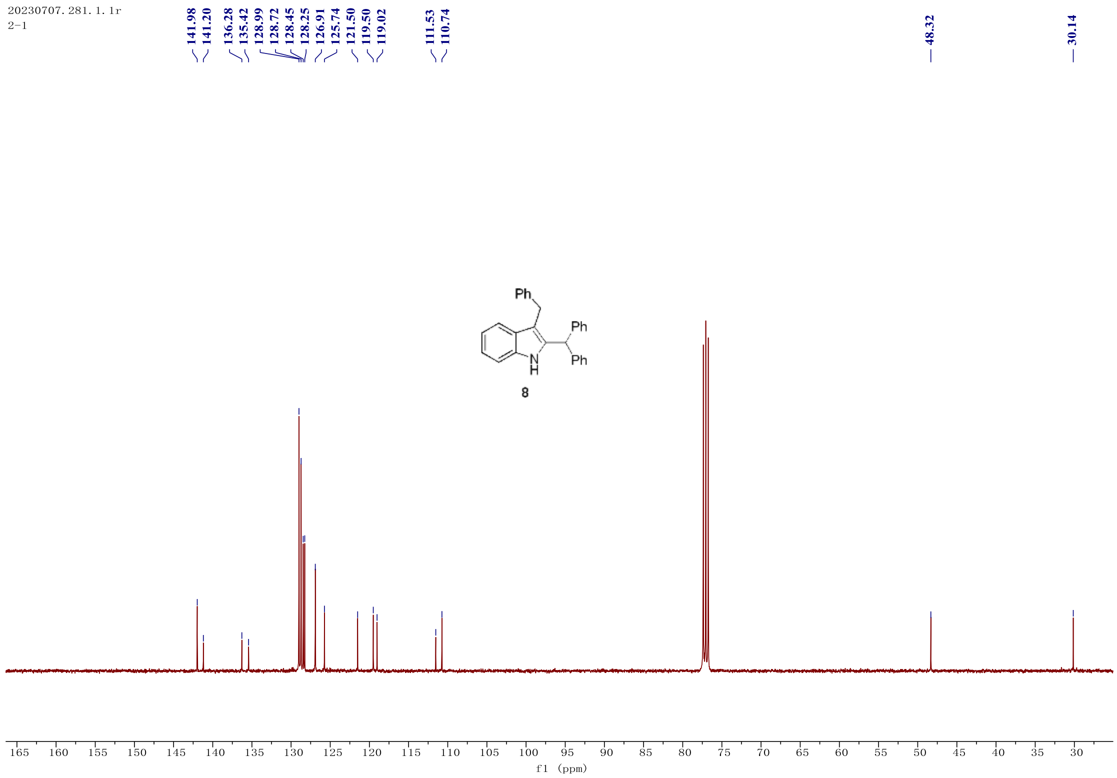
20230707.281.1.1r
2-1
141.98
141.20
136.28
135.42
128.99
128.72
128.45
128.25
126.91
125.74
121.50
119.50
119.02
111.53
110.74
48.32
30.14
Ph
Ph
Ph
N
H
8
f1 (ppm)

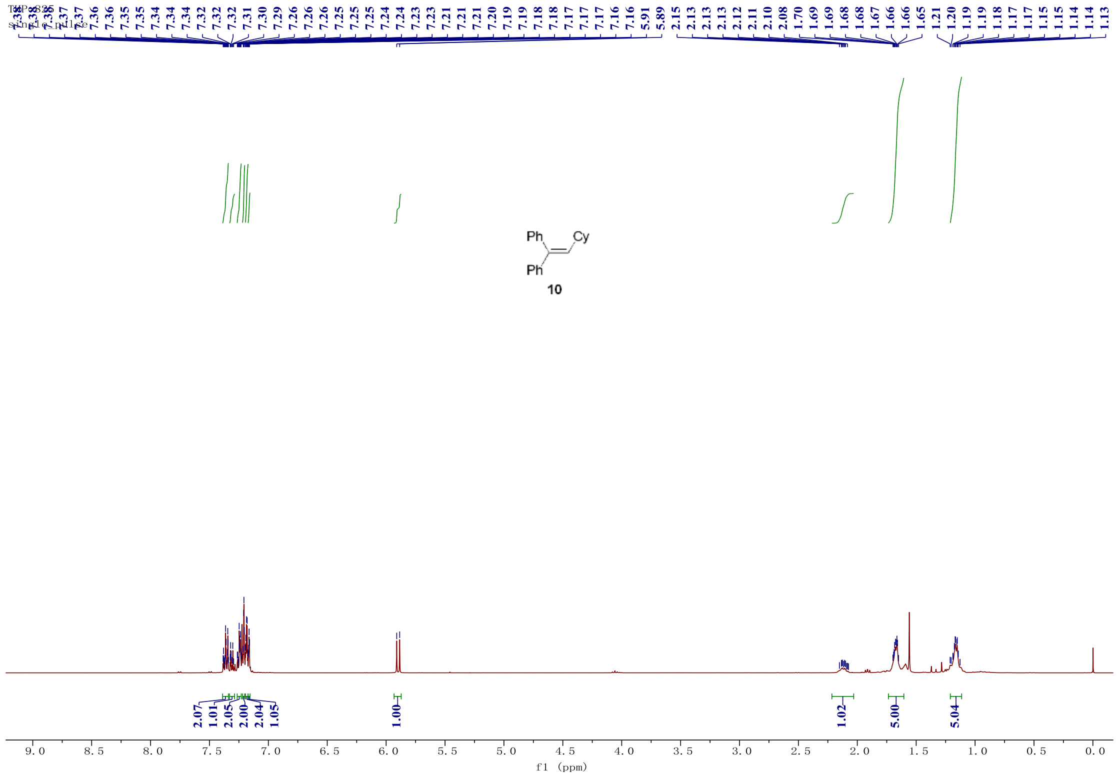
7.38 7.38 7.38 7.37 7.37 7.36 7.36 7.35 7.34 7.34 7.34 7.32 7.32 7.32 7.31 7.30 7.29 7.26 7.26 7.25 7.25 7.25 7.24 7.24 7.23 7.23 7.21 7.21 7.20 7.19 7.19 7.18 7.17 7.17 7.17 7.16 7.16 5.91 5.89 2.15 2.13 2.13 2.12 2.11 2.10 2.08 1.70 1.69 1.68 1.68 1.67 1.66 1.66 1.65 1.21 1.20 1.19 1.19 1.18 1.17 1.17 1.15 1.15 1.14 1.14 1.13
Ph
Cy
Ph
10
2.07
1.01
2.05
2.00
2.04
1.05
1.00
1.02
5.00
5.04
f1 (ppm)

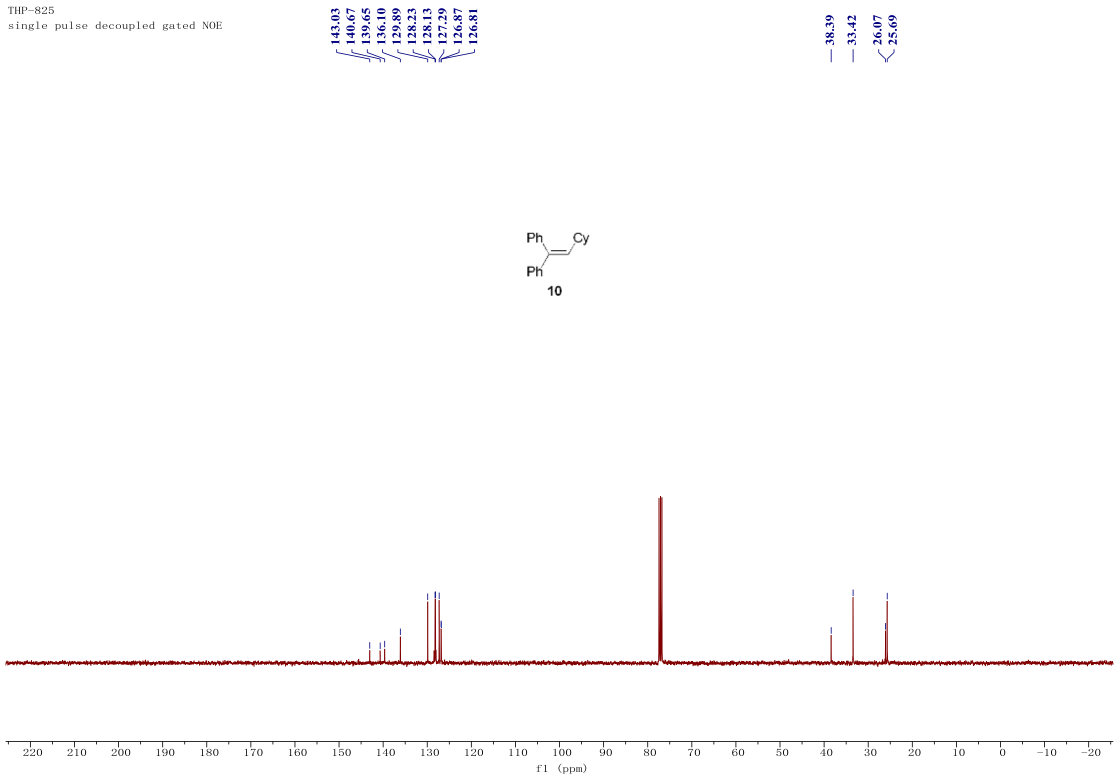
THP-825
single pulse decoupled gated NOE
143.03
140.67
139.65
136.10
129.89
128.23
128.13
127.29
126.87
126.81
38.39
33.42
26.07
25.69
Ph
Cy
Ph
10
220 210 200 190 180 170 160 150 140 130 120 110 100 90 80 70 60 50 40 30 20 10 0 −10 −20
f1 (ppm)
